# The Effectiveness of Mulligan's Techniques in Non‐Specific Neck Pain: A Systematic Review and Meta‐Analysis

**DOI:** 10.1002/pri.70045

**Published:** 2025-05-29

**Authors:** Jordana Barbosa‐Silva, Alexandre Luc, Ana Izabela Sobral de Oliveira‐Souza, Raphael Martins de Abreu, Jakelline Cipriano, Marine de Schaetzen, Laurent Pitance, Susan Armijo‐Olivo

**Affiliations:** ^1^ Physical Therapy Department Federal University of São Carlos São Carlos Brazil; ^2^ Faculty of Business and Social Sciences University of Applied Sciences Osnabrück Germany; ^3^ Neuro Musculo Skeletal Lab Institute of Experimental and Clinical Research Université Catholique de Louvain Brussels Belgium; ^4^ Department of Physiotherapy LUNEX University International University of Health Differdange Luxembourg; ^5^ LUNEX ASBL Luxembourg Health & Sport Sciences Research Institute Differdange Luxembourg; ^6^ University of Pernambuco Recife Brazil; ^7^ Federal Institute of Education Science and Technology of Alagoas Maceió Brazil; ^8^ Stomatology and Maxillofacial Surgery Department Cliniques Universitaires Saint‐Luc Université Catholique de Louvain Brussels Belgium; ^9^ Department of Physical Therapy Faculty of Rehabilitation Medicine University of Alberta Edmonton Canada; ^10^ Faculty of Medicine and Dentistry University of Alberta Edmonton Canada

**Keywords:** musculoskeletal manipulations, neck pain, physical therapy modalities, randomized controlled trials, systematic review

## Abstract

**Background and Purpose:**

Mulligan's techniques, such as Sustained Natural Apophyseal Glides (SNAGs) and Natural Apophyseal Glides (NAGs), are commonly applied by physiotherapists when treating patients with non‐specific neck pain (NP). However, there has been no comprehensive synthesis of their effects in NP. This review aimed to assess the effectiveness of Mulligan's techniques in reducing pain, improving disability, and enhancing cervical range of motion (CROM) in adults with acute, subacute, or chronic NP.

**Methods:**

A systematic review with meta‐analysis was conducted on randomized controlled trials (RCTs) comparing Mulligan's techniques with other interventions in adults with NP. Two reviewers independently conducted study selection, data extraction, and risk of bias (RoB) assessment. Meta‐analyses were performed when clinical homogeneity was present; otherwise, a narrative synthesis was used. Certainty of evidence was rated using the Grading of Recommendations, Assessment, Development and Evaluations (GRADE) approach.

**Results:**

Thirty‐three studies were included. For acute and mixed (acute/subacute/chronic) NP, Mulligan's techniques were no more effective than other interventions for pain reduction, disability improvement, or CROM enhancement. However, in patients with chronic or uncertain chronicity NP, SNAGs combined with other interventions demonstrated superior outcomes—both statistically and sometimes clinically—compared to certain treatments like exercises and muscle‐energy techniques, for reducing pain and disability and improving CROM. The certainty of evidence was rated very low.

**Discussion:**

Mulligan's techniques appear to be safe, simple, and potentially beneficial for managing mixed or chronic NP when combined with other interventions, presenting results that may be comparable or occasionally superior to other standard techniques.

**Implications for Physiotherapy Practice:**

Physiotherapists may consider incorporating Mulligan's techniques, especially SNAGs, within broader NP treatment strategies, as they offer a feasible, low‐risk option for improving patient outcomes, particularly for chronic NP cases when used alongside other therapies.

## 1 Introduction

Neck pain is within the top five most burdensome conditions in the world (Kim et al. 2018). The global prevalence of NP is 4.8% (Vos et al. 2012). Up to 70% of the general population suffer from NP at least once in their lifetime, with a high percentage (between 50% and 80%) of people suffering from neck pain on a recurrent basis. Prevalence increases with age, is higher in women than in men (56% vs. 44%), and more often affects people with lower levels of education (Blanpied et al. 2017). Recent reports estimate an increase in the prevalence of neck pain up to 32.5%, which includes around 269 million people globally affected in 2050 (GBD 2021 Neck Pain Collaborators 2024).

Mechanical neck dysfunction is the most common type of neck pain, commonly called non‐specific neck pain (NSNP), which has been defined as “neck pain occurring in the absence of trauma, signs or symptoms of major structural pathology, neurological signs, or specific pathology” (Blanpied et al. 2017). Current evidence shows that manual therapy (MT) (i.e., mobilization and manipulation) and exercises, including range of movement (ROM), strengthening, and endurance exercises (Côté et al. 2016; Gross et al. 2015), have a beneficial effect to manage acute NSNP and chronic NSNP. However, there is still controversies about the combination of different strategies while treating patients with neck pain, as previous evidences suggested that the combination of MT and exercise is not superior to the prescription of exercises alone (Fredin and Loras, 2017), while some authors showed that combining MT with exercise is better than MT or exercise alone (Hidalgo et al. 2017).

Among conservative strategies used to manage neck pain, Mulligan's techniques combine passive techniques (glides) with active mobilization of the patient. They are also characterized by self‐exercise treatments. These techniques are known as “Mobilizations With Movement” (MWM). Those applied to the spine are called Sustained Natural Apophyseal Glides (SNAGs) or Natural Apophyseal Glides (NAGs). They are frequently used by physiotherapists to reduce pain and restore full ROM when treating patients with NSNP (Hearn and Rivett, 2002). Cervical SNAGs allow normal pain‐free function by mobilizing the zygapophyseal joints while patients are performing their limited or painful neck movement (Hing et al. 2020).

Recently, six systematic reviews were published and demonstrated overall significant therapeutic effects of the Mulligan's techniques to decrease pain and disability and increase ROM in different musculoskeletal conditions, such as low back pain (Pourahmadi et al. 2018), conditions affecting peripheral joints (Stathopoulos et al. 2019; Weerasekara et al. 2020; Westad et al. 2019), shoulder disorders (Satpute et al. 2022) and pain and function in individuals with cervicogenic headache (Cardoso et al. 2022). However, none of the previous reviews focused on patients with NSNP. In the last decade, there has been an increasing number of published studies regarding Mulligan's techniques focusing on NSNP. Current clinical studies on Mulligan's interventions show mixed results, high heterogeneity and insufficient high‐quality evidence to confirm its effectiveness (Pourahmadi et al. 2018; Satpute et al. 2022; Stathopoulos et al. 2019; Westad et al. 2019). However, to the best of our knowledge, no study has systematically reviewed the effectiveness of the Mulligan's techniques applied to the cervical spine to treat acute NSNP, subacute, and chronic NSNP. Therefore, the aims of our systematic review are: (1) to provide a comprehensive overview of the current literature concerning Mulligan's techniques, applied alone or combined with other techniques, to manage adult patients with acute, subacute, and/or chronic NSNP, and (2) to analyze the effectiveness of Mulligan's techniques to treat acute, subacute, and chronic NSNP, compared with other therapies on neck pain intensity, disability, and neck range of motion (ROM).

## 2 Methods

The reporting of this systematic review was based on the PRISMA guidelines (Page et al. 2021). The protocol was registered in PROSPERO *(ID CRD42018111788).*

### 2.1 Study Selection

#### 2.1.1 Inclusion and Exclusion Criteria

Trials were selected if they included adults (≥ 18 years old) with diagnosis of acute (less than 30 days), subacute (> 30 days and < 90 days), or chronic (non‐specific) neck pain (> 90 days) NSNP, and had a randomized controlled design for interventions comparing Mulligan's techniques (SNAGs, self‐SNAGs, NAGs, and MWMs) applied alone or in combination with other techniques, and compared with no treatment, placebo, minimal treatment, or to any other treatment, whether conservative or non‐conservative, including but not limited to opioids, nonsteroidal anti‐inflammatory analgesics, surgery, therapeutic exercises, or other manual therapy techniques. Techniques were defined as followed (Hing et al. 2020):

– **MWM**: Mobilization With Movement (MWM) is a manual therapy technique that integrates passive joint mobilization by the therapist with active movement performed by the patient. This method aims to immediately reduce pain and restore normal movement by applying a sustained, pain‐free accessory glide to the joint while the patient actively moves through the impaired range of motion.
– **SNAGs**: The application of an MWM in the spine is referred to as a SNAG. This manual therapy technique applied to the spine involves the combination of a sustained passive accessory glide (or joint mobilization) applied in the plane of the facet joints by the physiotherapist to the spine (specific motion segment) with active movement from the patient. SNAGs can be applied centrally on the spinous process or laterally on the articular pillar.
– **NAGs**: Painless oscillatory mid‐to end‐range mobilization applied in the plane of the facet joints on the spinous process or articular pillar applied between C2 and C7.
– **Reverse NAGS**: Painless oscillatory mid‐to end‐range mobilization is applied in the plane of the facet joints on the spinous process or articular pillar. This technique can be applied between C6 and the upper thoracic spine.

Trials were excluded if they included participants with rheumatoid arthritis, spondylitis ankylosing, previous surgery in the cervical region, or other serious pathology that can lead to neck pain (e.g., fracture, tumor, traumatic injury). The main outcome of interest was pain intensity. Secondary outcomes of interest were cervical range of motion (CROM) and neck disability.

#### 2.1.2 Data Sources and Searches

This review is part of a large project analyzing the effectiveness of Mulligan's techniques in cervical spine musculoskeletal disorders, including acute, subacute, and chronic NSNP, cervicogenic dizziness, cervicogenic headache, cervical radiculopathy, and cervical spondylosis. The last update of the databases search was performed on April 15, 2024, on the following databases: MEDLINE, EMBASE, Cochrane Library and Best Evidence, CINAHL, and ISI Web of Science. The manual search was performed in September 2024. No limits were applied on the databases in relation to publication date, language, or publication status. Keywords used included Mulligan's techniques (“SNAG(s)”, “self‐SNAG(s)”, “NAG(s)”) and cervical disorders (“neck pain”, “cervicogenic dizziness”, “cervicogenic headache”), selected with the help of a librarian who is specialized in health science databases, and experts in orthopedic manual therapy. Supporting Information S1: Appendix 1 displays the search strategies used for each database. The search was complemented by looking for new studies on the list of references of the included studies and by manual‐searching. One of the authors is an expert in Mulligan's techniques and was consulted for ongoing trials in this field.

#### 2.1.3 Data Screening

Titles and abstracts and then full texts of potentially relevant studies were screened for inclusion by two independent reviewers using Covidence software (http://www.covidence.org). Disagreements regarding selection of studies were resolved by consensus. If no consensus was obtained, a third reviewer made a final decision.

#### 2.1.4 Data Extraction

Data extraction was performed by one reviewer and verified by a second reviewer, using an Excel data extraction form previously pilot‐tested. Data extracted from each trial, including qualitative and quantitative data, included the study characteristics, population studied, Mulligan interventions, comparison treatments and their details, primary and secondary outcomes, results, and authors' conclusion. Results from all follow‐ups were extracted and are presented in tables below. Data extracted for primary and secondary outcomes included statistical tests and their associated *p*‐value.

#### 2.1.5 Risk of Bias Assessment

The risk of bias assessment of the selected studies was performed by two independent reviewers using a compiled set of items based on the 7 tools used to evaluate the risk of bias in complex physical therapy trials (Armijo‐Olivo et al. 2008). In addition, the Cochrane risk of bias (RoB) tool was used to comprehensively assess the trials (Higgins and Altman, 2008). We used previously developed specific decision rules, as described elsewhere (Armijo‐Olivo et al. 2014). Studies were classified as having low, high RoB, or some concerns. Criteria to determine the overall risk of bias have been used previously (Armijo‐Olivo et al. 2014; Hartling et al. 2012).

#### 2.1.6 Data Analysis

The synthesis was structured around the type of NSNP (acute, subacute, and chronic), Mulligan techniques (SNAGs, NAGs), comparators, and outcomes (pain intensity, CROM, disability). In case of high clinical (participants, interventions, and outcomes), methodological (outcome measurement tools, risk of bias), or statistical (*I* ^2^ statistic superior to 80%) heterogeneity, studies were not pooled as recommended by the Cochrane collaboration. Therefore, a narrative synthesis was used (Deeks et al. 2023). For comparisons including at least two studies and low heterogeneity (*I* ^2^ statistic < 80%), meta‐analyses were performed, using Revman Review Manager (RevMan) version 5.0 (The Nordic Cochrane Center, The Cochrane Collaboration, Copenhagen, Denmark, 2008). Results are reported using mean differences (MD) or standardized mean differences (SMD), and their 95% confidence interval (95%CI). SMD were used if the outcome of interest was assessed using different scales (i.e. tools). To perform the data analysis, the effect of the interventions was computed in RevMan after the extraction of the data from pre‐ and post‐intervention measures.

Effect sizes (ES) (determined by the SMD) for respective comparisons and outcomes were interpreted using Cohen's d criteria (0.2, 0.5, and 0.8 were considered as small, moderate, and large effect sizes, respectively (Cohen, 1988)). Forest plots were provided using RevMan. Statistical significance was interpreted following previous recommendations (strong, good, moderate, and weak evidence against the null) (Sterne and Smith, 2001). Authors from seven studies were contacted in order to clarify numbers and doubts. Only one author confirmed that two included manuscripts analyzed the same population (Izquierdo Perez et al. 2014; Lopez‐Lopez et al., 2015), while the others did not reply our e‐mails to confirm data reported in the manuscripts (Gautam et al. 2014; Keyur et al. 2016; Manzoor et al. 2021; Ozlu and Sahin, 2024; Usama et al. 2022; Vijayan et al. 2022).

#### 2.1.7 Certainty of Evidence

The Grading of Recommendations, Assessment, Development and Evaluations (GRADE) approach, as described by Guyatt et al. (Guyatt et al. 2011), was used to determine the certainty of the overall evidence. Risk of bias, imprecision, inconsistency of results, indirectness of evidence, and publication bias can decrease the certainty of the evidence (Guyatt et al. 2011). Conversely, certainty can be increased for example in case of large effects or dose‐response associations. The overall certainty of evidence was classified as high, moderate, low, or very low (Guyatt et al. 2011). The SMD for each comparison was presented along with GRADE, after considering the significant variability in the tools used to assess the outcomes. Thus, this measure was used to present a clearer understanding of the ES for the respective comparisons and outcomes, which would not be achievable with MD measurements alone (although MDs were reported in the rest of the tables for completeness).

#### 2.1.8 Clinical Significance of the Results

Considering the outcomes, the clinical significance of all outcomes included in the present review were reported based on the cut‐offs found in the literature. Those cut‐offs are presented in Supporting Information S2: Appendix 2.

## 3 Results

### 3.1 Study Selection

**FIGURE 1:**
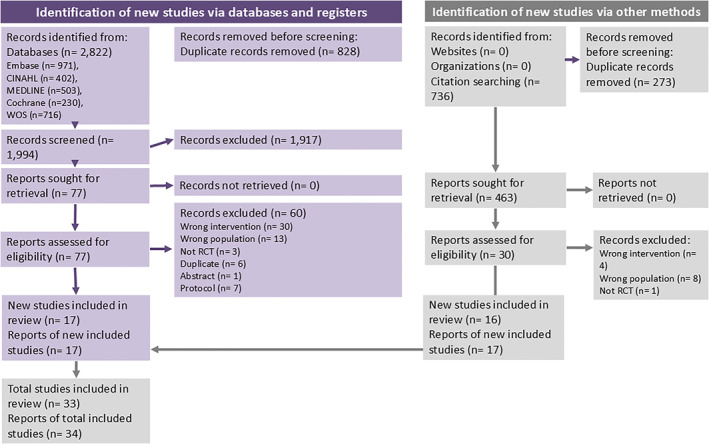
PRISMA flowchart.

Figure 1 (PRISMA flowchart) shows details of the selection process. During our first search, a total of 2822 potentially relevant studies were found but 828 were duplicates and removed, leaving 1994 records to be screened by titles and abstracts. From these, seventy‐eight studies were assessed in full and 17 studies (reported in 17 articles) were included. When conducting our manual search, 736 studies were found by citation searching, and from these, 30 studies were read in full. From these, 16 studies (reported in 17 articles) were included. One study was reported by two different manuscripts (Izquierdo Perez et al. 2014; Lopez‐Lopez et al. 2015) and, therefore, we merged both manuscripts into one. Therefore, 33 studies, reported in 34 manuscripts met the eligibility criteria. A list of excluded studies is provided in the Supporting Information [Link], [Link], [Link], [Link], [Link], [Link]: Appendix 3.

Table 1 is a summary of characteristics of included studies. Among studies included, two focused on acute NSNP (Ganesh et al. 2015; Tank et al. 2018), three on subacute and chronic NSNP (Alansari et al. 2021; El‐Sodany et al. 2014; Shelke et al. 2023), one study focused on acute and subacute neck pain (Hussain et al. 2016), one study included a mixed group of participants with acute, subacute, and chronic NSNP (Vijayan et al. 2022), and 18 on chronic NSNP (Abd El‐Azeim and Grase, 2023; Ali et al. 2014; Alshami and AlSadiq, 2021; Buyukturan et al. 2018; Duymaz and Yagci, 2018; Izquierdo Perez et al. 2014; Keyur et al. 2016; Kumar et al. 2011; Lopez‐Lopez et al., 2015; Manzoor et al. 2021; Mohamed and Elrazik, 2020; Morsi et al. 2023; Pal and Misra, 2019; Rezkallah and Abdullah, 2018; Said et al. 2017; Shamsi et al. 2021; Sun et al. 2024; Tachii et al. 2015; Zemadanis, 2018). Eight studies did not report the stage of NSNP (Aggarwal and Verma, 2018; Gautam et al. 2014; Ozlu and Sahin, 2024; Shehri et al. 2018; Sultan et al. 2021; Tanveer et al. 2017; Usama et al. 2022; Vijayan et al. 2022; Waqas et al. 2017).

**TABLE 1:** Description of included studies (*n* = 33).

**Country**	***n* (%)**		**Mulligan technique** **^a^**	***n* (%)** **^a^**
China	1 (3.03)		NAGs	2 (6.06)
Egypt	6 (18.18)		SNAGs	21 (63.63)
Greece	1 (3.03)		Self‐SNAGs	3 (9.09)
India	9 (27.27)		Combination of mulligan techniques	6 (18.18)
Pakistan	7 (21.21)		Others	3 (9.09)
Saudi Arabia	4 (12.12)		Other treatment^a^	*n* (%)^a^
Spain	1 (3.03)		Maitland	6 (18.18)
Turkey	3 (9.09)		Exercises	16 (48.48)
Not reported	1 (3.03)		CT	16 (48.48)
Published date	*n* (%)		HVLA	1 (3.03)
2011	1 (3.03)		Sham/No treatment	*n* (%)
2014	5 (15.15)		Placebo or SHAM mulligan	2 (6.06)
2015	2 (6.06)		Outcomes^a^	*n* (%)
2016	2 (6.06)		Pain	33 (100)
2017	3 (9.09)		ROM	24 (72.72)
2018	7 (21.21)		Neck disability	28 (84.84)
2019	1 (3.03)		Total sample size	*n* (%)
2020	1 (3.03)		< 50	16 (48.48)
2021	5 (15.15)		≥ 50 but < 100	18 (54.54)
2022	2 (6.06)		Diagnostic tool^a^	*n* (%)^a^
2023	3 (9.09)		VAS	20 (60.60)
2024	2 (6.06)		NPRS	14 (42.42)
Study design	*n* (%)		Inclinometer	4 (12.12)
RCT	33 (100%)		Goniometer	12 (36.36)
Diagnosis	*n* (%)		CROM device (no device described)	5 (15.15)
Acute/subacute nonspecific neck pain	4 (12.12)		NDI	26 (78.78)
Acute, subacute, and chronic nonspecific neck pain	3 (9.09)		NPDS	1 (3.03)
Chronic mechanical neck pain	2 (6.06)		CNFDS	1 (3.03)
Chronic nonspecific neck pain	17 (51.51)		Funding	*n* (%)
Mechanical neck pain (no further description)	5 (15.15)		Not reported	16 (48.48)
Work‐related neck pain (no further description)	1 (3.03)		No funding	16 (48.48)
Non‐specific neck pain	2 (6.06)		Government or others	1 (3.03)
Trial registered	*n* (%)		Ethical approval	*n* (%)
Yes	22 (66.66)		Yes	27 (81.81)
Not reported	12 (36.36)		Not reported	7 (21.21)

Abbreviations: CNFDS, Copenhagen Neck Functional Disability Scale; CROM, Cervical Range of Motion; CT, Conventional therapy; HVLA, high‐velocity low‐amplitude; NAGs, natural apophyseal glides; NDI, Neck disability index; NPDS, Neck Pain and Disability Scale; NPRS, Numeric Pain Rating Scale; RCT, Randomized controlled trial; ROM, Range of motion; SNAGs, sustained natural apophyseal glide; VAS, Visual Analog scale.

^a^ numbers do not add up since several studies were included in these categories.

Mulligan's techniques were SNAGs (Abd El‐Azeim and Grase, 2023; Alansari et al. 2021; Ali et al. 2014; Buyukturan et al. 2018; Duymaz and Yagci, 2018; El‐Sodany et al. 2014; Ganesh et al. 2015; Gautam et al. 2014; Izquierdo Perez et al. 2014; Keyur et al. 2016; Lopez‐Lopez et al., 2015; Manzoor et al. 2021; Mohamed and Elrazik, 2020; Morsi et al. 2023; Ozlu and Sahin, 2024; Pal and Misra, 2019; Rezkallah and Abdullah, 2018; Shamsi et al. 2021; Shehri et al. 2018; Shelke et al. 2023; Sultan et al. 2021; Tachii et al. 2015; Tank et al. 2018; Tanveer et al. 2017; Usama et al. 2022; Vijayan et al. 2022; Waqas et al. 2017), self‐SNAGs (Aggarwal and Verma, 2018; Duymaz and Yagci, 2018; Said et al. 2017; Sun et al. 2024; Zemadanis, 2018), NAGs (Buyukturan et al. 2018; Gautam et al. 2014; Hussain et al. 2016; Kumar et al. 2011; Manzoor et al. 2021; Sun et al. 2024; Usama et al. 2022; Waqas et al. 2017), and Mulligan MWMs for the scapula (Alshami and AlSadiq, 2021).

They were compared to passive accessory intervertebral movements (PAIVMS) (Alansari et al. 2021; Ganesh et al. 2015; Izquierdo Perez et al. 2014; Lopez‐Lopez et al., 2015), high velocity low amplitude (HVLA) manipulation techniques (Izquierdo Perez et al. 2014; Lopez‐Lopez et al., 2015), exercises programs (ROM, strengthening, and stretching exercises) (Duymaz and Yagci, 2018; El‐Sodany et al. 2014; Keyur et al. 2016; Mohamed and Elrazik, 2020; Rezkallah and Abdullah, 2018; Shehri et al. 2018), conventional treatment (hot pack, transcutaneous electrical nerve stimulation (TENS), interferential therapy, ultrasound, massage, active or isometric exercises) (Abd El‐Azeim and Grase, 2023; Aggarwal and Verma, 2018; Alshami and AlSadiq, 2021; Buyukturan et al. 2018; Gautam et al. 2014; Mohamed and Elrazik, 2020; Ozlu and Sahin, 2024; Pal and Misra, 2019; Said et al. 2017; Shamsi et al. 2021; Shehri et al. 2018; Sultan et al. 2021; Tachii et al. 2015; Vijayan et al. 2022; Waqas et al. 2017), muscle energy techniques (Manzoor et al. 2021; Tank et al. 2018; Usama et al. 2022), myofascial release (Duymaz and Yagci, 2018; Gautam et al. 2014; Keyur et al. 2016; Morsi et al. 2023; Sultan et al. 2021; Tanveer et al. 2017), neck motor control training using the craniocervical flexion test (CCFT) (Shelke et al. 2023), cervical and cervicothoracic self‐mobilization (Sun et al. 2024), placebo (Kumar et al. 2011) and sham techniques (Zemadanis, 2018). A more detailed description of the studies is presented in Supporting Information S4 Appendix 4. Figure 1 (PRISMA flowchart) shows details of the selection process.

### 3.2 Risk of Bias Assessment

**FIGURE 2:**
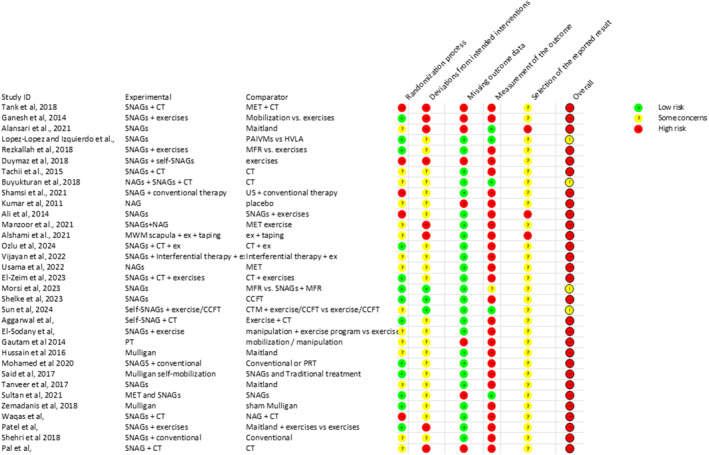
Risk of bias of studies included in the present review, according to Cochrane RoB tool.

Figure 2 presents the risk of bias assessment according to the Cochrane RoB tool. The overall risk of bias for the majority of studies (n = 29) was classified as high, only four studies were classified with some concerns (Buyukturan et al. 2018; Izquierdo Perez et al. 2014; Lopez‐Lopez et al., 2015; Morsi et al. 2023; Sun et al. 2024). None of the studies was classified as low risk of bias.

Only one study reported by two different manuscripts (Izquierdo Perez et al. 2014; Lopez‐Lopez et al., 2015) accomplished 81% of the compiled items analyzed, one study presented 72.5% of accomplished items (Zemadanis, 2018), five studies accomplished around 61%–63% (Buyukturan et al. 2018; Ganesh et al. 2015; Rezkallah and Abdullah, 2018; Shelke et al. 2023; Sun et al. 2024), 45%–51% (Abd El‐Azeim and Grase, 2023; Aggarwal and Verma, 2018; Alansari et al. 2021; Alshami and AlSadiq, 2021; El‐Sodany et al. 2014; Morsi et al. 2023; Said et al. 2017; Shamsi et al. 2021; Sultan et al. 2021) and 31%–36% items (Duymaz and Yagci, 2018; Hussain et al. 2016; Keyur et al. 2016; Kumar et al. 2011; Mohamed and Elrazik, 2020; Tachii et al. 2015; Tanveer et al. 2017; Vijayan et al. 2022), six studies accomplished 24%–30% of items (Ali et al. 2014; Gautam et al. 2014; Pal and Misra, 2019; Shehri et al. 2018; Tank et al. 2018; Waqas et al. 2017), and one study (Manzoor et al. 2021) accomplished only 18% of the items.

Detailed results of the critical appraisal of the included studies are presented in Supporting Information S5: Appendix 5. The certainty of the evidence using GRADE for all comparisons is reported in Supporting Information S6: Appendix 6.

### 3.3 Quantitative Analyses (SMD and Mean Difference (MD) are Presented in Supporting Information S6: Appendix 6)

A summary of the results is presented in a matrix available in Supporting Information S7: Appendix 7. Results were analyzed based on clinical and statistical significance.

#### 3.3.1 Acute NSNP

##### 3.3.1.1 Pain Intensity

A statistical significance difference was found between SNAGS plus conventional therapy versus muscle energy technique and conventional therapy at 2 weeks (large ES, SMD: 0.97 [0.31, 1.63]) favoring the comparison group (see Supporting Information S6: Appendix 6, GRADE 6.1) (Tank et al. 2018). SNAGs were no statistically significantly better than PAIVMs (Ganesh et al. 2015) or an exercise program (Ganesh et al. 2015) at 2 weeks (Ganesh et al. 2015; Tank et al. 2018) to reduce pain (ES ranged between very small to moderate) (Supporting Information S6: Appendix 6, GRADE 6.2 and 6.3).

At 12 weeks (Ganesh et al. 2015), no statistically significant differences between treatments were reported, although small and moderate effect sizes favoring SNAG group compared to PAIVMS and an exercise program were found (see Supporting Information S6: Appendix 6, GRADE 6.4 and 6.5). None of the results was considered clinically significant. All these results had very low level of evidence (Supporting Information S6: Appendix 6; analyses 6.1–6.5).

##### 3.3.1.2 Cervical Range of Motion

Two studies reported that all the interventions (SNAGs, muscle energy techniques, PAIVMs and exercises) were equally effective to significantly improve CROM after 2 weeks (Ganesh et al. 2015; Tank et al. 2018) and 12 weeks (Ganesh et al. 2015) with no significant differences among groups (in general). It was not possible to determine the clinical significance of the treatment for the SNAGs and muscle energy techniques groups based on the MIDs as authors (Tank et al. 2018) did not specify which device they applied during data collection; however, the SMD were in general small. Differences between SNAGs and PAIVMs and exercises were not considered clinically significant based on the interpretation of the ES (small to moderate ES (SMD)) (very low evidence according to GRADE) (see Supporting Information S6: Appendix 6, analyses 6.6 to 6.31).

##### 3.3.1.3 Disability

All groups (SNAGs, muscle energy techniques, PAIVMs, and exercises) showed statistically significant improvement in the neck disability index (NDI) with no statistically significant differences between groups after 2 weeks (Ganesh et al. 2015; Tank et al. 2018) and 12 weeks (Ganesh et al. 2015) (moderate‐weak evidence against the null).

ES varied from small to moderate at 2 and 12 weeks, with very low evidence according to GRADE (see Supporting Information S6: Appendix 6, analyses 6.32–6.36). However, according to MD presented in Supporting Information S6: Appendix 6 and the cut‐off points considered for this study (Supporting Information S2: Appendix 2), changes could be considered clinically significant at 12 weeks, favoring SNAGs versus PAIVMS and exercise (Ganesh et al. 2015).

#### 3.3.2 Acute, Subacute, and Chronic NSNP (Mixed)

Findings comparing SNAGs versus PAIVMs regarding pain intensity and neck disability after 3 weeks (Alansari et al. 2021) showed a small effect, with no significant differences between groups, and a very low certainty in the evidence (see Supporting Information S4: Appendix 4, analyses 4.37 and 4.38). Similarly, both SNAGs and neck motor control using the CCFT seem to improve pain intensity and CROM immediately after one‐single session (Shelke et al. 2023) but a small effect and very low certainty of the evidence were found, based on GRADE (see Supporting Information S4: Appendix 4, analyses 4.39–45). None of the comparisons was considered clinically significant for both outcomes.

Comparisons between SNAGs plus interferential therapy plus isometric neck exercises versus interferential therapy (IFC) plus isometric neck exercises alone resulted in improvement on pain intensity as well as CROM in both groups (Vijayan et al. 2022), with significantly greater effectiveness in the group that received SNAGs in addition to IFC and neck exercises at 2 weeks for pain intensity (large effect; Supporting Information S6: Appendix 6, 6.46) neck flexion, extension, and left rotation movements (CROM, large effect; Supporting Information S6: Appendix 6, 6.47, 6.48, and 6.50). Measurements of right rotation were considered statistically and clinically significant, favouring the comparison group (large effect; Appendix 6.49). Based on MD, measures of CROM were considered clinically relevant, although the certainty of the evidence was considered very low.

The combination of NAGS and CT was better than Maitland applied with CT to improve neck pain and disability at 4 weeks (moderate ES, Supporting Information S6: Appendix 6, 6.52 and 6.54) but no significant results were shown neither for pain nor disability at 2 weeks (Hussain et al. 2016) (moderate ES, Supporting Information S6: Appendix 6, 6.51 and 6.53). One study (El‐Sodany et al. 2014) did not report numerical results but authors concluded that both SNAGs and manipulation were effective in the treatment of subacute/chronic on pain, disability, and CROM; no one was superior to the other. In addition, the combination of SNAG or manipulation with exercise therapy produced greater increase in CROM and greater reduction of pain, compared to exercise alone (Supporting Information S6: Appendix 6, analyses 6.55–66). The certainty of the evidence was considered very low for all comparisons.

#### 3.3.3 Chronic NSNP

The quantitative analysis focused on the effectiveness of Mulligan's techniques on chronic NSPS is presented in Supporting Information S6: Appendix 6 (analyses 6.67–6.222). Only six studies were included in a meta‐analysis as they were comparable regarding the population, intervention, comparator, and follow‐ups. Results from meta‐analysis are presented in Supporting Information S8: Appendix 8.

##### 3.3.3.1 Pain Intensity

Pooled effects of three studies (Buyukturan et al. 2018; Shamsi et al. 2021; Tachii et al. 2015) comparing SNAGs plus CT and exercises versus CT plus exercises presented a small ES at two weeks (three studies, 170 participants, *I* ^2^ = 0%, SMD [95%CI] = −0.30 [−0.61; 0.00]; *p* = 0.05) (Appendix 8,Figure 8.1.1). However, when adding a recent study to this analysis (Abd El‐Azeim and Grase, 2023), a large ES between these two interventions (SNAGs plus CT and exercises vs. CT plus exercises) at two‐four weeks was found (four studies, 260 participants, *I* ^2^ = 97%, SMD [95%CI] = −1.52 [−3.38; 0.35]; *p* = 0.11) (Appendix 8, Figure 8.1.2), however, there was a high heterogeneity especially driven by this recent study (Abd El‐Azeim and Grase, 2023). No significant differences between groups on pain intensity were found but analyses were considered potentially clinically relevant according to SMD (large ES; Appendix 6, analysis 6.70).

According to the pooled estimate of two studies (Duymaz and Yagci, 2018; Rezkallah and Abdullah, 2018), when SNAGs were associated with exercises, SNAGs had a superior clinically relevant effect than exercises alone but no statistical difference was found (large ES [Supporting Information S6: Appendix 6, analysis 6.75]; Appendix 8, Figure 8.2; two studies, high RoB, 87 participants, *I* ^2^ = 32%, SMD [95%CI] = −2.38 [−3.07, −1.69]; *p* = < 0.00001).

At 3–4 weeks, groups that received only Mulligan techniques seem to be favored (moderate/large ES) compared to those receiving a different manual therapy or a sham therapy (SNAGs plus NAGs vs. muscle energy techniques alone (Manzoor et al. 2021), moderate ES (Supporting Information S6: Appendix 6, analysis 6.73); SNAG plus NAG and self‐SNAG versus sham (Zemadanis, 2018) (large ES, Supporting Information S6: Appendix 6; analyses 6.74, 6.81). Findings and ES were similar when Mulligan's techniques were combined with different approaches (self‐SNAGs and CT vs. CT (Supporting Information S6: Appendix 6; analysis 6.79); SNAGs and CT versus CT (Supporting Information S6: Appendix 6; analysis 6.80) (Said et al. 2017)). At 8 weeks, Mulligan techniques' combined with different approaches also presented moderate/large ES (SNAGs plus CT plus exercises vs. CT plus exercises plus positional release therapy (Supporting Information S6: Appendix 6; analysis 6.85) (Mohamed and Elrazik, 2020); SNAGs plus myofascial release versus SNAGs (Supporting Information S6: Appendix 6, analysis 6.87; SNAG plus myofascial release vs. myofascial release (Appendix 6, 6.88)).

Comparisons between Mulligan's techniques versus HVLA, MWM, myofascial release and myofascial release plus exercises did not show any significant differences between groups (Supporting Information S6: Appendix 6, analyses 6.68, 6.71–72, 6.76–6.78, 6.84, 6.86, 6.89–6.92), except for a comparison between SNAGs vs PAIVM after a single session that showed a statistically and clinically significant differene, favouring the comparison group (Appendix 6, analysis 6.69). However, it is worth noting that although not statistically significant differences were obtained, HVLA technique tended to be superior to Mulligan techniques right after the treatment (reaching large ES) (Supporting Information S6: Appendix 6, analysis 6.68). In addition, cervicothoracic mobilization plus exercise, including motor control of the neck through the CCF training was significantly better than self‐SNAGs plus exercise (large ES) at 6 weeks (Supporting Information S6: Appendix 6, analysis 6.83).

Two studies (Ali et al. 2014; Kumar et al. 2011) did not report numerical results on pain intensity but authors concluded that NAGs were superior to a placebo (Kumar et al. 2011) (Supporting Information S6: Appendix 6, analysis 6.67) and SNAGs associated with exercises were superior to SNAGs alone (Ali et al. 2014) (Supporting Information S6: Appendix 6, analysis 6.82). Both studies reported a statistically significant difference between the groups.

All these results had very low evidence based on GRADE (Supporting Information S6: Appendix6 ; analyses 6.67–6.92).

##### 3.3.3.2 CROM

Different movements of CROM were assessed, including flexion, extension, rotation, and lateral flexion. However, for some movements, authors did not specify which side was evaluated (i.e., right or left) (Duymaz and Yagci, 2018; Izquierdo Perez et al. 2014; Lopez‐Lopez et al., 2015; Manzoor et al. 2021), thus, it was unclear in which direction results were significant. Therefore, we are presenting results according to specific movements below only for those studies with clear data.

## 4 Neck Flexion (Supporting Information S6: Appendix 6, Analyses 6.94–6.114)

Most of the comparisons did not show a clear superiority among interventions for improving neck flexion. Some of the highlights for these comparisons will be reported in more detail below.

Only one comparison favored the application of Mulligan's techniques alone (statistically and clinically superiority favoring the intervention group) when compared to a different manual therapy technique (Manzoor et al. 2021) (large ES, Supporting Information S6: Appendix 6, analysis 6.97). Most of the comparisons favored the application of Mulligan's techniques combined with additional therapies.

When pooling the results of two studies (Buyukturan et al. 2018; Shamsi et al. 2021), statistical and clinically differences were found, favouring the group that received SNAGs, conventional therapy and exercises versus a group that received conventional therapy and exercises only (large ES [Appendix 6, analysis 6.96]) (high heterogeneity, results are presented for completeness and should be interpreted with cautions; Appendix 8, Figure 8.3; two studies, 140 participants, *I* ^2^ = 94%, SMD [95%CI] = 1.62 (–0.01, 3.25); *p* = 0.05); Moreover, in a second pooled analysis with two studies (Duymaz and Yagci, 2018; Rezkallah and Abdullah, 2018), SNAGs plus exercises was found to be better than exercises alone to increase neck flexion at short term (large ES [Supporting Information S6: Appendix 6, analysis 6.100]), statistically and clinically significant differences (Appendix 8, Figure 8.4) (two studies, 87 participants, *I* ^2^ = 80%, SMD [95%CI] = 1.73 (0.59, 2.87); *p* = 0.003). At 8 weeks, SNAG applied in combination with CT and exercises were superior to positional release therapy plus CT and exercises (Mohamed and Elrazik, 2020) (statistically and clinically significant) (large ES, Supporting Information S6: Appendix 6, analysis, 6.109). At 12 weeks, SNAG plus exercises were superior than exercises alone (Duymaz and Yagci, 2018) (statistically and clinically significant, large ES [Supporting Information S6: Appendix 6, analysis 6.112b])).

The rest of the comparisons did not show a clear superiority between treatments (Supporting Information S6: Appendix 6, analyses 6.94–96, analyses 6.98–99, 6.101–108, 6.110–111, 6.113–114).

## 5 Neck Extension (Supporting Information S6: Appendix 6, Analyses 6.115–6.135)

Similar to the previous section, most of the comparisons did not show a clear superiority among interventions for improving neck extension (20 comparisons). To enhance the completeness of our findings, we reported the meta‐analysis with pooled effects of two studies comparing SNAGs plus exercises versus exercises alone (Supporting Information S6: Appendix 6, analyses 6.117 and 6.119; Supporting Information S8: Appendix 8, Figure 8.5 and 8.6), however, results presented high heterogeneity, and the interpretation of the results should be done with caution.

Nonetheless, SNAG plus NAG demonstrated a superior effect than muscle energy techniques (Manzoor et al. 2021) (large ES, statistically and potentially clinically relevant [Supporting Information S6: Appendix 6, analysis 6.120]) to improve neck extension at 3 weeks. At 6 weeks, a large ES (statistically and clinically relevant) favoring self‐SNAGs plus exercise/CCFT versus exercise/CCFT alone (Supporting Information S6: Appendix 6, analysis 6.126) was found. Similarly, at 8 weeks, SNAG applied in combination with CT and exercises were superior to positional release therapy plus CT and exercises (Mohamed and Elrazik, 2020) (large ES, statistically and potentially clinically relevant; Supporting Information S6: Appendix 6, analysis, 6.130). At 12 weeks, SNAGs plus exercise showed superiority to increase neck extension versus exercises alone (Duymaz and Yagci, 2018) (large ES, statistically and potentially clinically relevant [Supporting Information S6: Appendix 6, analysis 6.133]). The rest of the comparisons did not show a clear superiority between treatments (Supporting Information S6: Appendix 6, analyses 6.116, 6.118–6.119, 6.121–6.125, 6.127–6.129, 6.131–132, 6.134–6.136). Clinically relevance favouring the group that received Mulligan techniques was observed in absence of statistical differences when comparing a) SNAGs vs. HVLA (Appendix 6, analysis 6.115), b) SNAGs plus CT plus exercises vs. CT and exercises (Appendix 6, analysis 6.117) and c) SNAGs plus exercises vs. exercises (Appendix 6, analysis 6.119). All analyses were classified as having very low certainty of evidence.

## 6 Neck Lateral Flexion (Supporting Information S6: Appendix 6, Analyses 6.136–6.165)

From almost 30 comparisons looking at neck lateral flexion, only a bit more than a third showed differences among groups. These results will be highlighted below.

Immediately after a single session, SNAGs seemed to be better than mobilization to improve lateral flexion (large ES representing a potential clinically significant result ; Supporting Information S6: Appendix 6, analysis 6.137) (Izquierdo Perez et al. 2014; Lopez‐Lopez et al., 2015). Similarly, at 12 weeks, SNAGs combined with exercises were superior to exercises applied alone (Duymaz and Yagci, 2018) (large ES representing a potential clinically significant result, statistical significance; Supporting Information S6: Appendix 6, analysis 6.143).

In a pooled analysis of two studies (Buyukturan et al. 2018; Shamsi et al. 2021), SNAGs plus CT and exercises were found to be superior than CT plus exercises alone to increase neck lateral flexion (right and left) at short‐term (Supporting Information S8: Appendix 8, Figure 8.7 and 8.8). SMDs were high, but MDs were lower than the cut‐off of CROM provided by the literature (Supporting Information S2: Appendix 2), so these results were considered statistically but only potentially clinically significant, with large ES [Supporting Information S6: Appendix 6; analyses 6.146 and 6.157]) (left lateral flexion, two studies, 140 participants, *I* ^2^ = 0%, SMD [95%CI] = 1.09 ([0.73; 1.45]; *p* < 0.00001; right lateral flexion, two studies, 170 participants, *I* ^2^ = 0%, and SMD [95%CI] = 0.90 [0.55; 1.25]; *p* < 0.00001; *I* ^2^ = 0%).

A substantial improvement of neck lateral flexion favoring SNAGs plus exercises versus exercises alone was found (Rezkallah and Abdullah, 2018) (large ES [Supporting Information S6: Appendix 6; analyses 6.149 and 6.160]), SNAGs plus exercises versus myofascial release plus exercise (Rezkallah and Abdullah, 2018) (large and moderate ES respectively [Supporting Information S6: Appendix 6; analyses 6.150 and 6.161]), SNAGs plus myofascial release versus SNAGs alone (Morsi et al. 2023) (large ES at 8 weeks, Supporting Information S6: Appendix 6; analyses 6.154 and 6.165) and SNAGs plus myofascial release versus myofascial release alone (large ES at 8 weeks, Supporting Information S6: Appendix 6; analyses 6.155 and 6.166) (Morsi et al. 2023), SNAGs combined with CT and exercises versus positional release therapy plus CT and exercises (Mohamed and Elrazik, 2020) (large ES, Supporting Information S6: Appendix 6, analyses 6.156 and 6.167), which means that most of the subjects receiving Mulligan techniques achieved greater lateral flexion than other interventions, especially when combined with other interventions.

For right lateral flexion, a comparison between self‐SNAGs plus exercise/CCFT versus cervicothoracic mobilization plus exercise/CCFT showed a large ES at 6 weeks (Sun et al. 2024) (Supporting Information S6: Appendix 6; analysis 6.162), favoring the comparison group.

The rest of the comparisons did not show a clear superiority. All these results had very low evidence based on GRADE (Supporting Information S6: Appendix 6, analyses 6.136; 6.138–6.142; 6.144–6.145; 6.147–6.148; 6.151–6.153; 6.158–6.159, 6.163–6.164).

## 7 Neck Rotation (Supporting Information S6: Appendix 6, Analyses 6.168–6.197)

Almost 30 comparisons evaluated the effect of different interventions on neck rotation. From these, only a few comparisons demonstrated a difference between groups (either statistical or clinical). Significant and potentially clinically relevant differences based on SMD were found between SNAGs plus CT and exercises versus CT and exercises alone (Buyukturan et al. 2018; Shamsi et al. 2021) pooling the results of two studies (large ES [Supporting Information S6: Appendix 6, analyses 6.176 and 6.187] (Supporting Information S8: Appendix 8, Figure 8.9 and 8.10, respectively), (two studies, 140 participants, *I* ^2^ = 0%, SMD [95%CI] = 1.84 [1.11; 2.57]; *p* < 0.00001; *I* ^2^ = 66% for left side and SMD [95%CI] = 1.51 [1.13; 1.89]; *p* < 0.00001; *I* ^2^ = 0% for right side).

Large ES were found for both left and right rotation by comparing SNAGs plus exercises versus exercises alone (Rezkallah and Abdullah, 2018) (potentially clinically relevant based on large ES [Supporting Information S6: Appendix 6, analyses 6.179 and 6.190];) and ESs for SNAG plus myofascial release versus myofascial release alone (Supporting Information S6: Appendix 6, analyses 6.184 and 6.196, respectively) (Morsi et al. 2023). At 8 weeks, SNAG applied in combination with CT and exercises were statistically superior and potentially clinically relevant to positional release therapy plus CT and exercises on neck rotation (Mohamed and Elrazik, 2020) (large ES, Supporting Information S6: Appendix 6, analyses, 6.186 and 6.197; right and left rotation, respectively). At 12 weeks (Duymaz and Yagci, 2018), the comparison between SNAGs plus exercises versus exercises alone showed a large ES (potentially clinically relevant), favoring the combined group that included SNAGs (Supporting Information S6: Appendix 6, analysis 6.173). Clinically significance (according to MD and the cut‐of points presented in Appendix 2) was observed in absence of statistical differences and small SMD when comparing SNAGs vs. mobilization (PAIVMs), favouring the Mulligan group (Appendix 6, analysis 6.175).

Left rotation showed a significantly and potentially clilnically improvement when comparing a) self‐SNAGs plus exercise/CCFT versus cervicothoracic mobilization plus exercise/CCFT at 6 weeks (Sun et al. 2024) (large ES, Supporting Information S6: Appendix 6, analysis 6.181); and b) SNAGs alone versus SNAGs plus MFR at 8 weeks (Supporting Information S6: Appendix 6, analyses 6.184 and 6.185) (Morsi et al. 2023), favoring the group that received Mulligan techniques combined with other therapies. Similarly, right rotation was improved significantly when comparing SNAGs plus CT and exercises vs. CT and exercises (large ES, Appendix 6, analysis 6.187), SNAGs plus exercises vs exercises alone (large ES, Appendix 6, analysis 6.190), and self‐SNAGs plus exercise/CCFT versus exercise/CCFT (Sun et al. 2024), favoring the group that received Mulligan's techniques combined with different treatments (large ES, Supporting Information S6: Appendix 6, analyses 6.193 and 6.196).

Finally, NAGs were superior to a sham intervention for all directions except right lateral flexion (statistically significant differences reported only qualitatively, not quantitatively; one study; Supporting Information S6: Appendix 6, analysis 6.93) (Kumar et al. 2011).

The rest of the comparisons did not show a clear superiority of Mulligan techniques when‐compared with other interventions for neck rotation (Supporting Information S6: Appendix 6, analyses 6.168–6.172, 6.174, 6.177–178, 6.180, 6.182–6.183, 6.188–6.189, 6.191–192, 6.194–6.195). All these results had very low evidence based on GRADE.

### 7.1 Disability

About 25 comparisons looked at neck disability. From these, only 11 demonstrated a difference between treatments. In a meta‐analysis of three studies, a moderate effect was found, favoring SNAGs associated with CT and exercises versus CT alone plus exercises (Supporting Information S8: Appendix 8, Figure 8.11.1) (three studies, 170 participants, *I* ^2^ = 0%, SMD [95%CI] = −0.68 [−0.99, −0.37]; *p* < 0.0001). When adding a recent study, a pooled effect of four studies, similar results were obtained (SNAGs associated with CT and exercises were superior to CT alone plus exercises with a large ES at 2–4 weeks (Supporting Information S6: Appendix 6; analysis 68.200; Supporting Information S8: Appendix 8, Figure 8.11.2); (four studies, 260 participants, *I* ^2^ = 95%, SMD [95%CI] = −1.35 [−2.63, −0.06]; *p* < 0.04) (Abd El‐Azeim and Grase, 2023; Buyukturan et al. 2018; Shamsi et al. 2021; Tachii et al. 2015), however, there was a high heterogeneity (*I* ^2^ = 95%), especially driven by this recent study (Abd El‐Azeim and Grase, 2023).

Pooled results of two studies comparing SNAGs associated with exercises were superior to exercises alone (Duymaz and Yagci, 2018; Rezkallah and Abdullah, 2018) (two studies, 87 participants, *I* ^2^ = 0%, MD [95%CI] = −8.65 [−9.57, −7.73]; *p* < 0.001) (statistically and clinically significant differences, with a large ES [Supporting Information S6: Appendix 6, analysis 6.203; Supporting Information S8: Appendix 8, Figure 8.12]).

Comparisons from single studies also showed large ES, favoring the intervention groups, especially including the addition of Mulligan techniques to other treatment strategies: (a) SNAGs plus NAGs were superior to muscle energy techniques post‐isometric relaxation on reducing NDI scores (statistically and clinically significant differences) (Manzoor et al. 2021) (Supporting Information S6: Appendix 6, analysis 6.201); (b) the combination of SNAG with NAG and self‐SNAGs were superior than sham Mulligan techniques at 3 and 4 weeks, respectively (Supporting Information S6: Appendix 6, analyses 6.202 and 6.211) (Zemadanis, 2018); (c) SNAGs associated with exercises were found to be superior to myofascial release associated with exercises (large ES, potentially clinically significant based on the ES; Supporting Information S6: Appendix 6; analysis 6.206) (Rezkallah and Abdullah, 2018); (d) self‐SNAGs plus CT and SNAGs plus CT presented a large ES at 4 weeks, favoring the Mulligan’ groups when compared to CT applied alone (Supporting Information S6: Appendix 6, analyses 6.209–210) (Said et al. 2017); (e) SNAGs applied in combination with CT and exercises were better than positional release therapy and CT plus exercises, favoring the Mulligan group (Mohamed and Elrazik, 2020) (Supporting Information S6: Appendix 6, analysis 6.215); (f) SNAGs plus myofascial release versus SNAGs alone (Morsi et al. 2023) (large ES at 8 weeks, Supporting Information S6: Appendix 6; analysis 6.217) and SNAGs plus myofascial release versus myofascial release alone (large ES at 8 weeks, Supporting Information S6: Appendix 6; analysis 6.218) (Morsi et al. 2023), favored the groups with combined interventions containing Mulligan's techniques; (g) self‐SNAGs, SNAGs plus NAGs versus sham, favouring the Mulligan group (large ES at 8 weeks, Appendix 6; analysis 6.211) (Zemadanis 2018).

All these results had very low evidence based on GRADE (Supporting Information S6: Appendix 6).

#### 7.1.1 Non‐Specified Chronicity (Unclear)

The quantitative analysis of eight studies that focused on the effectiveness of Mulligan's techniques on individuals without specifying the chronicity of NSPS is presented below. Approximately, 45 different comparisons were investigated by these studies. Relevant results are discussed below. Details of the rest of the comparisons are provided in Supporting Information S6: Appendix 6, analyses 6.223–6.269.

### 7.2 Pain Intensity

Most of the comparisons that included combined therapies in addition to Mulligan techniques were found to be superior than other techniques.

In order to increase the completeness of our findings, we reported a meta‐analysis with two pooled studies showed a large ES favoring the Mulligan's group when comparing SNAGs and CT versus CT alone (Aggarwal and Verma, 2018; Ozlu and Sahin, 2024) (two studies, 78 participants, *I* ^2^ = 0%, MD [95%CI] = −1.76 [−4.07, 0.54]; *p* = 0.13) (not statistically but potentially clinically significant considering the large ES [Appendix 6, analysis 6.223; Appendix 8, Figure 8.13), however, results presented high heterogeneity, and the interpretation of the results should be done with caution.

Pain intensity was statistically reduced at 2 weeks (large ES favoring Mulligan's group) when comparing SNAG plus CT versus NAG plus CT (Waqas et al. 2017) (Supporting Information S6: Appendix 6, analysis 6.224). At 4 weeks, Mulligan's group (NAGs) was significantly superior than muscle energy technique (result was also considered clinically significant) (Usama et al. 2022) (Supporting Information S6: Appendix 6, analysis 6.226), SNAGs plus CT versus CT (Supporting Information S6: Appendix 6, analysis 6.227) (Aggarwal and Verma, 2018); SNAG with CT and exercises versus Maitland combined CT and exercises (Shehri et al. 2018) (Appendix 6, analysis 6.228); SNAG combined with neck exercises vs. a) Maitland and exercises (Tanveer et al. 2017), and b) exercises alone (Tanveer et al. 2017) (Supporting Information S6: Appendix 6, analyses 6.229–230); and self SNAG combined with CT versus CT (Aggarwal and Verma, 2018) (Supporting Information S6: Appendix 6, analysis 6.231). At 8 weeks, the combination of SNAGs, muscle energy technique, and CT was superior than SNAGs and CT, with a large ES favoring the group that received both manual therapies associated with CT (Sultan et al. 2021) (Supporting Information S6: Appendix 6, analysis 6.232).

### 7.3 CROM

Regarding CROM, superiority between interventions was not clearly demonstrated. Only few comparisons reported a significant difference. For instance, SNAGs plus CT versus CT at 2 weeks (Ozlu and Sahin, 2024) showed a clinically and statistical improvement favoring the Mulligan's group when analyzing flexion (Supporting Information S6: Appendix 6, analysis 6.233).

Pooled results for extension did not show statistical differences (high heterogeneity, results are presented for completeness and should be interpret with cautions; Supporting Information S8: Appendix 8, Figure 8.14, two studies, 78 participants, *I* ^2^ = 86%, MD [95%CI] = 3.95 [−9.73, 17.63], *p* = 0.57)) (Supporting Information S6: Appendix 6, analysis 6.237) or left lateral flexion (high heterogeneity, results are presented for completeness and should be interpret with cautions; Supporting Information S8: Appendix 8, Figure 8.15, two studies, 78 participants, *I* ^2^ = 87%, MD [95%CI] = 5.50 [–3.51, 14.52], *p* = 0.23)) (Supporting Information S6: Appendix 6, analysis 6.243).

Pooled results for right lateral flexion showed no statistical differences between groups (Supporting Information S6: Appendix 6, analysis 6.248) (Supporting Information S8: Appendix 8, Figure 8.16, two studies, 78 participants, *I* ^2^ = 78%, MD [95%CI] = 3.97 [−3.22, 11.17]. *p* = 0.28)) (Aggarwal and Verma, 2018; Ozlu and Sahin, 2024). Similar, no statistical differences were found for left rotation (high heterogeneity, results are presented for completeness and should be interpret with caution; Appendix 8, Figure 8.17, two studies, 78 participants, *I* ^2^ = 97%, MD [95%CI] = 15.10 [–3.71, 33.91], *p* = 0.12)) (Appendix 6, analysis 6.254) and right rotation in the same comparison group (high heterogeneity, results are presented for completeness and should be interpret with cautions; Supporting Information S8: Appendix 8, Figure 8.18, two studies, 78 participants, *I* ^2^ = 95%, MD [95%CI] = 12.87 [−2.07, 27.81]. *p* = 0.09)) (Appendix 6, analysis 6.259).

At 4 weeks, clinically and statistical differences for flexion and extension were shown when comparing NAGs versus muscle energy technique, favoring the Mulligan's technique (Usama et al. 2022) (Supporting Information S6: Appendix 6, analyses 6.234, 6.239). Similarly, results favoring the Mulligan's techniques were found when comparing SNAGs plus NAGs, CT, and exercises versus CT combined with exercises for left lateral flexion (Supporting Information S6: Appendix 6, analysis 6.246) (Gautam et al. 2014). Flexion, extension, right and left lateral flexion, and right and left rotation were significantly improved in subjects who received Mulligan's techniques plus muscle relaxation technique and CT, compared with a group that received only SNAGs and CT at 8 weeks (Sultan et al. 2021) (Appendix 6, analyses 6.236, 6.242, 6.247, 6.252, 6.257, 6.262).

### 7.4 Disability

Only a few comparisons looking at disability were carried out in this set of studies.

The pooled results of two studies showed no statistical differences between SNAG plus CT and CT alone (Aggarwal and Verma 2018; Ozlu and Sahin 2024) (high heterogeneity, results are presented for completeness and should be interpret with cautions; Supporting Information S8: Appendix 8, Figure 8.19, two studies, 78 participants, *I* ^2^ = 97%, MD [95%CI] = −13.05 [−29.47, 3.38], *p* < 0.12)) (Supporting Information S6: Appendix 6, analysis 6.263).

At short‐term, Mulligan’ techniques were statistically and clinically different than muscle energy techniques (Usama et al. 2022)) to improve neck disability (large ES, Supporting Information S6: Appendix 6, analysis 6.265). The comparison between SNAGs plus exercise versus Maitland and exercise at 4 weeks showed a large ES favoring Mulligan's techniques (Tanveer et al. 2017) (Supporting Information S6: Appendix 6, analysis 6.267).

All comparisons had very low evidence based on GRADE (Supporting Information S6: Appendix 6).

### 7.5 Adverse Events

Adverse events were reported in only three studies (Ganesh et al. 2015; Izquierdo Perez et al. 2014; Shelke et al. 2023). Izquierdo et al. (Izquierdo Perez et al. 2014) and Shelke et al. (Shelke et al. 2023) reported no adverse events with any of the interventions. Ganesh et al. (Ganesh et al. 2015) reported that none of the mobilization group participants had major side effects except local muscle and joint soreness, which rarely led to even short‐term impairment in functional status.

## 8 Discussion

Findings from the present systematic review highlight that a) in acute NSNP, SNAGs combined with CT or exercise, are not superior than exercise or other manual therapies in reducing pain, disability, or improving CROM; (b) in subacute/chronic NSNP, SNAGs alone offer no additional benefits compared to exercise or manual therapies. However, in subjects with mixed chronicity of NSNP (subjects with acute, subacute or chronic in the same sample), SNAGs combined with exercise and interferential therapy show better results for CROM; (c) combining SNAGs with CT, exercise, and other manual techniques (NAGs, myofascial release) is more effective than prescribing any of these therapies alone in chronic NSNP; (d) For unspecified chronicity, SNAGs with CT are better than performing CT alone, while NAGs are better than muscle energy techniques.

According to our findings, the effectiveness of Mulligan's techniques was associated with several interventions (i.e., exercises, CT, different manual therapies), which highlights the limited impact of Mulligan mobilization alone on improving pain, CROM, and disability. These results align with existing clinical guidelines which recommend a combination of approaches to treat individuals with NSNP, based on the chronicity of the condition. For acute and subacute neck pain, manual therapy and exercise therapy are generally advised, while for chronic NSNP, a multimodal approach is recommended, combining manual therapy, exercise therapy, medication, dry needling, or laser therapy (Blanpied et al. 2017; Côté et al. 2016). Moreover, the literature also suggests that manual therapy should be applied in combination with exercises in order to improve pain and patients' satisfaction (Gross et al. 2002). Therefore, results from the present review support the combination of Mulligan's techniques with different types of treatments, in accordance with previous guidelines, for individuals with different NSNP chronicity.

One hypothesis for the absence of superiority of Mulligan's techniques might be the heterogeneity of parameters for interventions and comparators reported by primary studies, which might have an influence on the effect of different interventions. Another hypothesis might be the patients' clinical presentation. It is known that manipulations might not succeed in cases where there might be a high level of psychosocial factors involved in the pain mechanism and when nociplastic pain is present (Baeske et al. 2020). However, it was not possible to establish an association between covariables and the main findings from studies that were included in the present review, as there was a lack of reporting of the association between covariables and the main results, as well as poor reporting of the outcomes.

We believe that the low quality of the methodology and risk of biases of the included studies might have contributed to the lack of clear findings. Our findings are similar to the previous literature that included patients with cervicogenic headache (Cardoso et al. 2022) and low back pain (Pourahmadi et al. 2018). We also found a low percentage of items accomplished in the compiled list of items and RoB tool when assessing the trials' RoB. Therefore, results from studies should be interpreted with caution, considering the lack of information of important criteria of a RCT that includes appropriated randomization method, concealment of allocation, blinding appropriateness and others (please see Supporting Information S5: Appendix 5) which could result in an inaccurate estimation of the treatment effect (Savovic et al. 2012). Other aspects that should be considered while interpreting the results is the report of compliance and adherence to the treatment, and the use of appropriate statistical analysis to interpret the results (i.e., intention‐to‐treat analysis); that was poorly reported by the authors. Therefore, it is unclear if subjects adequately followed the protocol of treatment, specially during the analysis of the follow‐ups.

An important strength of this systematic review is the rigorous methodological sequence followed to complete the study, which included the preparation and registration of a protocol for the review, and a systematic search on the most accessed databases. The selection, data extraction and RoB assessment of the studies was performed by two independent researchers. Moreover, the present review only included RCTs that analyzed the effectiveness of Mulligan's techniques compared to other interventions, which provide the highest level of evidence in terms of primary studies of interventions.

The present review has some limitations. The first one is related to the diversity of studies found in the literature, which has impacted the meta‐analysis of the estimates. However, it was possible to include a heterogeneous population (i.e., female and male, age range, and nationality) in our review; a fact that contributes to the external validity of our results. Another limitation might be related with the quality of the report of the results. Some authors did not report the estimates using numerical values (Ali et al. 2014; Kumar et al. 2011), which limited our analysis of clinical significance and pooling the results into a single estimation. Although some authors were contacted for more details, they did not answer our emails. It is already pointed out by the literature that results from studies that applied manual therapy techniques are generally incomplete and poorly described (Núñez‐Cortés et al. 2022). Only reporting the narrative synthesis of the results is also a limitation as the information is incomplete and makes the assessment of the validity of its findings difficult (Campbell et al. 2019).

In addition, the absence of high‐quality studies and evidence limited our ability to make strong conclusions, which highlights the need for well‐designed RCTs concerning NSNP, especially the main domains that were used to downgrade the certainty in the evidence (Supporting Information S6: Appendix 6) (risk of bias, level of heterogeneity (inconsistency), and imprecisions surrounding the effect estimates). However, the present review has a similarity with previous studies that analyzed Mulligan's techniques: the high/moderate risk of bias of primary studies and the limited number of trials included in the review.

### 8.1 Implications on Physiotherapy Practice

This systematic review demonstrates that the combination of Mulligan's techniques with other interventions, particularly CT, exercise, and manual techniques, is more effective for individuals with chronic or mixed chronicity NSNP (acute, subacute, and chronic) when comparing those modalities alone or in absence of the Mulligan's technique. This highlights the importance of integrating SNAGs in combination with different therapies for optimal patient outcomes, rather than relying on SNAGs alone or other modalities alone. For patients with acute NSNP, Mulligan's techniques applied in combination with CT and/or exercises do not show superior effects when compared to CT, other manual techniques (PAIVMs) and/or exercises alone.

Our study gives clinicians an extra string to their bow when treating subjects with NSNP. Depending on the preferences and expectations of their patients, clinicians can use Mulligan's techniques to improve CROM and/or reduce pain intensity and associated disability, in addition to other interventions. The use of self‐Mulligan's techniques can also be applied as it meets the recommendations of combining manual techniques with self‐exercises.

Moreover, we specified the clinical significance of the comparisons (magnitude of ES), exploring more than the statistical analysis reported by primary studies (which is based only on *p* values). However, clinical significance was observed in a few comparisons for outcomes. This result should be interpreted with attention as clinicians working with patients with NSNP should follow the concept of evidence‐based practice, by choosing any of these techniques that fit the patient preferences and expectations in order to result in a clinically significant change in the health status of the patient (Nilsagård et al. 2019). Nonetheless, clinicians and researchers can focus on comparisons that were classified as clinically relevant and can be successfully implemented in clinical practice. Thus, readers are encouraged to incorporate these outcomes in their future clinical studies to measure the effectiveness Mulligan's interventions, taking into consideration not only statistical significance but also clinical relevance.

Evidence about the effectiveness of Mulligan's techniques for treating patients with NSNP is limited and with very low certainty of evidence. Caution should be drawn in order to prescribe these specific techniques to reduce pain and improve CROM or neck disability in patients with NSNP. Our findings might result in a more meticulous approach for this population, as results from these techniques are very heterogeneous, with low to very‐low certainty in the evidence. Future studies should be performed with better methodological standards to increase the certainty in the evidence of the effectiveness of Mulligan's techniques in patients with NSNP. Researchers should also investigate the efficacy of Mulligan's techniques according to the clinical presentation of the patient with NSNP and other factors that might be related to pain (e.g., psychosocial factors, nociplastic pain), as they may influence the efficacy of the intervention (Cardoso et al. 2022). The identification of patients who best respond to these techniques would be a desirable next step.

## Conflicts of Interest

The authors declare no conflicts of interest.

## Supporting information

Supporting Information S1

Supporting Information S2

Supporting Information S3

Supporting Information S4

Supporting Information S5

Supporting Information S6

Supporting Information S7

Supporting Information S8

## Data Availability

The authors have nothing to report.
