# Supplementary material for: The Effectiveness of Mulligan's Techniques in Non‐Specific Neck Pain: A Systematic Review and Meta‐Analysis
Source: Physiother Res Int. 2025 May 29;30(3):e70045. doi: 10.1002/pri.70045 (PMC12121345; doi:10.1002/pri.70045)
Supplement: Supplementary file 1 — Supporting Information S1 [file PRI-30-e70045-s006.docx]

**Appendix 1. Databases search strategy.**

**Medline, 15 April 2024**

1 cervicogenic headache*.mp. 685

2 cervical headache*.mp. 59

3 exp Cervical Vertebrae/ 42547

4 exp Neck/ 32445

5 exp Headache/ 29941

6 cephalalgia.mp. 559

7 exp Dizziness/ 6044

8 cervical dizziness.mp. 11

9 exp Neck Pain/ 7977

10 3 or 4 73538

11 5 or 6 30280

12 10 and 11 735

13 7 and 10 100

14 1 or 2 or 8 or 9 or 12 or 13 9191

15 mulligan.tw,kf. 203

16 (Mobilization with Movement or mobilisation with movement).mp. 132

17 Natural Apophyseal Glides.mp. 25

18 Sustained Natural Apophyseal Glides.mp. 25

19 ((NAGS or SNAGS or MWMs) and (manual technique* or manipulation* or mobilization or mobilisation or exercise*)).mp. 33

20 manual therap*.mp. or exp Musculoskeletal Manipulations/ 19754

21 15 or 16 or 17 or 18 or 19 or 20 19952

22 14 and 21 957

23 randomized controlled trial.mp. or exp Randomized Controlled Trial/ 603996

24 exp Clinical trial/ or randomized.tw. or placebo.tw. or dt.fs. or randomly.tw. or trial.tw. or groups.tw. 5522509

25 23 or 24 5523128

26 22 and 25 501

27 (neckache or neckache or cervicodynia or cervicalgia).mp. 243

28 ((pain or ache) adj3 (neck or cervical or cervicothoracic or cervicogenic)).mp. 18738

29((chronic pain or intractable pain or musculoskeletal pain or non?cancer pain or non?malignant pain or non? neoplastic pain or myofascial pain or myofascial trigger point*) adj8 (neck or cervical or cervicothoracic or cervicogenic)).mp. 1294

30 neck pain.mp. or exp Neck Pain/ 14774

31 (Headache adj4 neck).mp. [mp=title, abstract, original title, name of substance word, subject heading word, floating sub-heading word, keyword heading word, organism supplementary concept word, protocol supplementary concept word, rare disease supplementary concept word, unique identifier, synonyms] 1410

32 (Headache adj4 cervical spine).mp. [mp=title, abstract, original title, name of substance word, subject heading word, floating subheading word, keyword heading word, organism supplementary concept word, protocol supplementary concept word, rare disease supplementary concept word, unique identifier, synonyms] 45

33 (Headache adj4 (neckache or neck ache or cervicodynia or cervicalgia)).mp. 32

34 (Dizziness adj4 neck).mp. [mp=title, abstract, original title, name of substance word, subject heading word, floating sub-heading word, keyword heading word, organism supplementary concept word, protocol supplementary concept word, rare disease supplementary concept word, unique identifier, synonyms] 186

35 (Dizziness adj4 cervical spine).mp. [mp=title, abstract, original title, name of substance word, subject heading word, floating subheading word, keyword heading word, organism supplementary concept word, protocol supplementary concept word, rare disease supplementary concept word, unique identifier, synonyms] 10

36 (Dizziness adj4 (neckache or neck ache or cervicodynia or cervicalgia)).mp. 1

37 14 or 27 or 28 or 29 or 30 or 31 or 32 or 33 or 34 or 35 or 36 20608

38 21 and 25 and 37 650

39 limit 38 to yr= « 2020 » - Current 119

40 limit 39 to (humans and randomized controlled trial) 44

**Embase, 15 April 2024**

1 cervical headache.mp. or exp cervicogenic headache/ 1574

2 cervical dizziness.mp. 19

3 cervical vertebrae.mp. or exp cervical spine/ 42365

4 headache*.mp. or exp headache/ 314921

5 cephalalgia.mp. 1646

6 exp neck pain/ 27630

7 exp dizziness/ 88636

8 4 or 5 315112

9 3 and 8 1891

10 3 and 7 342

11 1 or 2 or 6 or 9 or 10 30401

12 mulligan.mp. 328

13 (Mobilization with Movement or mobilisation with movement).mp. [mp=title, abstract, heading word, drug trade name, original title, device manufacturer, drug manufacturer, device trade name, keyword heading word, floating subheading word, candidate term word] 158

14 Natural Apophyseal Glides.mp. 32

15 Sustained Natural Apophyseal Glides.mp. 32

16 ((NAGS or SNAGS or MWMs) and (manual technique* or manipulation* or mobilization or mobilisation or exercise*)).mp. 40

17 manual therapy.mp. or exp manipulative medicine/ 40933

18 12 or 13 or 14 or 15 or 16 or 17 41242

19 randomized controlled trial.mp. or exp Randomized Controlled Trial/ 946444

20 exp Clinical trial/ or randomized.tw. or placebo.tw. or dt.fs. or randomly.tw. or trial.tw. or groups.tw. 8388624

21 19 or 20 8438689

22 11 and 18 and 21 1175

23 (neckache or neck ache or cervicodynia or cervicalgia).mp. 414

24 ((pain or ache) adj3 (neck or cervical or cervicothoracic or cervicogenic)).mp. 36039

25 ((chronic pain or intractable pain or musculoskeletal pain or non?cancer pain or non?malignant pain or non? neoplastic pain or myofascial pain or myofascial trigger point*) adj8 (neck or cervical or cervicothoracic or cervicogenic)).mp. 2958

26 neck pain.mp. or exp Neck Pain/

27 (Headache adj4 neck).mp. [mp=title, abstract, heading word, drug trade name, original title, device manufacturer, drug manufacturer, device trade name, keyword heading word, floating subheading word, candidate term word] 2317

28 (Headache adj4 cervical spine).mp. [mp=title, abstract, heading word, drug trade name, original title, device manufacturer, drug manufacturer, device trade name, keyword heading word, floating subheading word, candidate term word] 70

29 (Headache adj4 (neckache or neck ache or cervicodynia or cervicalgia)).mp. 50

30 (Dizziness adj4 neck).mp. [mp=title, abstract, heading word, drug trade name, original title, device manufacturer, drug manufacturer, device trade name, keyword heading word, floating subheading word, candidate term word] 285

31 (Dizziness adj4 cervical spine).mp. [mp=title, abstract, heading word, drug trade name, original title, device manufacturer, drug manufacturer, device trade name, keyword heading word, floating subheading word, candidate term word] 16

32 (Dizziness adj4 (neckache or neck ache or cervicodynia or cervicalgia)).mp. 3

33 23 or 24 or 25 or 26 or 27 or 28 or 29 or 30 or 31 or 32 37845

34 4 or 5 or 33 344007

35 11 or 33 40233

36 18 and 21 and 35 1371

37 limit 36 to yr="2020 - Current" 206

38 limit 37 to (human and randomized controlled trial and yr="2020 -Current") 100

39 limit 38 to yr="2022 -Current") 128

**CINAHL, 15 April 2024**

S1 TX cervicogenic headache

S2 TX cervicogenic headache OR cervical headache

S3 TX cervicogenic headache OR cervical headache*

S4 TX cervicogenic headache* OR TX cervical headache* OR TX cervical dizziness

S5 TX cervical vertebrae AND neck

S6 headache* OR cephalalgia

S7 dizziness

S8 S5 AND S7

S9 S5 AND S6

S10 S4 OR S8 OR S9

S11 neck pain

S12 S10 OR S11

S13 Mulligan

S14 Mobilization with Movement or mobilisation with movement

S15 Natural Apophyseal Glides

S16 Sustained Natural Apophyseal Glides

S17 ((NAGS or SNAGS or MWMs) and (manual technique* or manipulation* or mobilization or mobilisation or exercise*))

S18 (MH "Manual Therapy+")

S19 S13 OR S14 OR S15 OR S16 OR S17 OR S18

S20 randomized controlled trials OR randomised controlled trial

S21 exp Clinical trial/ or randomized.tw. or placebo.tw. or dt.fs. or randomly.tw. or trial.tw. or groups.tw.

S22 S20 OR S21

S23 S12 AND S19 AND S22

S24 neckache OR neck ache OR cervicodynia OR cervicalgia

S25 ((pain OR ache) N3 (neck OR cervical OR cervicothoracic OR cervicogenic))

S26 ((chronic pain OR intractable pain OR musculoskeletal pain OR non?cancer pain OR non?malignant pain OR non?neoplastic pain OR myofascial pain OR myofascial trigger point*) N8 (neck OR cervical OR cervicothoracic OR cervicogenic))

S27 Headache N4 neck

S28 Headache N4 cervical spine

S29 Headache N4 (neckache OR neck ache OR cervicodynia OR cervicalgia)

S30 Dizziness N4 neck

S31 Dizziness N4 cervical spine

S32 Dizziness N4 (neckache OR neck ache OR cervicodynia OR cervicalgia)

S33 S24 OR S25 OR S26 OR S27 OR S28 OR S29 OR S30 OR S31 OR S32

S34 S19 AND S22 AND S33

S35 (S24 OR S25 OR S26 OR S27 OR S28 OR S29 OR S30 OR S31 OR S32) AND (S19 AND S22 AND S33) (Limiters - Published Date: 20200501- 20221231)

S36 (S24 OR S25 OR S26 OR S27 OR S28 OR S29 OR S30 OR S31 OR S32) AND (S19 AND S22 AND S33) (Limiters - Published Date: 20200501- 20221231)

S37 S 19 AND S22 AND S33 (Limiters - Published Date: 20200501- 20221231)

S38 S 19 AND S22 AND S33 (Limiters - Published Date: 20220401- 20240431) 75

**Web of Science, 15 April 2024**

#1 TS=(cervicogenic headache) 1358

#2 TS=(cervical headache) 1358

#3 TS=(cervical vertebrae) 63885

#4 TS=(cervical dizziness) 1409

#5 TS=(dizziness) 41835

#6 TS=(Neck) 747519

#7 #3 OR #6 793575

#8 TS=(Headache) 179014

#9 TS=(Cephalalgia) 1193

#10 TS=(Neck pain) 54314

#11 #8 OR #9 179371

#12 7 and 5 1828

#13 7 and 11 10252

#14 TS=(Neackache OR neck ache OR cervicodynia OR cervicalgia) 1943

#15 TS=((((pain OR ache) NEAR/3 (neck OR cervical OR cervicothoracic OR cervicogenic))) 32475

#16 TS=((((chronic pain OR intractable pain OR musculoskeletal pain OR non?cancer pain OR non?malignant pain OR non?neoplastic pain OR myofascial pain OR myofascial trigger point*NEAR/8 neck OR cervical OR cervicothoracic)))) 764812

#17 TS=((Headache NEAR/4 cervical spine) 2095

#18 TS=(Headache NEAR/4 neck) 119

#19 TS=((Headache NEAR/4 neckache OR neck ache OR cervicodynia OR cervicalgia)) 1919

#20 TS=(Dizziness NEAR/4 neck) 363

#21 TS=(Dizziness NEAR/4 cervical spine) 19

#22 TS=(Dizziness NEAR/4 neckache OR neck ache OR cervicodynia OR cervicalgia) 1898

#23 #17 OR #18 OR #19 OR #20 OR #21 OR #22 776350

#24 #1 OR #2 OR #12 OR #13 OR #23 779744

#25 TS=(Mulligan) 971

#26 TS=(Natural Apophyseal Glides) 65

#27 TS=(Sustained Natural Apophyseal Glides) 53

#28 TS=(manual therap*) 73585

#29 TS=((nags OR snags OR mwms) AND (manual technique OR manipulation OR mobilization OR mobilisation OR exercise)) 357

#30 #29 OR #28 OR #27 OR #26 OR #25 74756

#31 TS=( randomised controlled trial) 687958

#32 TS=( randomised controlled trial) 687958

#33 TS=(controlled trial) 1265617

#34 #33 OR #32 OR #31 1265617

#35 #24 AND #30 AND #34 1569

#36 #35 (limit by 2020-05-27 to 2022-04-25) 308

#37 #35 and Humans 209

#38 #35 (limit by 2022-04-01 to 2024-04-15) 225

**Cochrane, 15 April 2024**

#1 cervicogenic headache 281

#2 cervical headache 929

#3 cervical vertebrae 1536

#4 cervical dizziness 297

#5 dizziness 15506

#6 Neck 30269

#7 #3 OR #6 31077

#8 Headache 36193

#9 Cephalalgia 1372

#10 Neck pain 9109

#11 #8 OR #9 36560

#12 7 and 5 645

#13 7 and 11 1518

#14 Neackache OR neck ache OR cervicodynia OR cervicalgia 148

#15 (((pain OR ache) NEAR/3 (neck OR cervical OR cervicothoracic OR cervicogenic))) 5586

#16 (((chronic pain OR intractable pain OR musculoskeletal pain OR non?cancer pain OR non?malignant pain OR non?neoplastic pain OR myofascial pain OR myofascial trigger point*NEAR/8 neck OR cervical OR cervicothoracic ))) 64338

#17 (Headache NEAR/4 neck) 347

#18 (Headache NEAR/4 cervical spine) 43

#19 (Headache NEAR/4 neckache OR neck ache OR cervicodynia OR cervicalgia) 148

#20 (Dizziness NEAR/4 neck) 62

#21 (Dizziness NEAR/4 cervical spine) 13

#22 (Dizziness NEAR/4 neckache OR neck ache OR cervicodynia OR cervicalgia) 145

#23 #17 OR #18 OR #19 OR #20 OR #21 OR #22 549

#24 #1 OR #2 OR #12 OR #13 OR #23 2486

#25 Mulligan 621

#26 Natural Apophyseal Glides 52

#27 Sustained Natural Apophyseal Glides 49

#28 manual therap* 14642

#29 ((nags OR snags OR mwms) AND (manual technique OR manipulation OR mobilization OR mobilisation OR exercise)) 47

#30 randomized controlled trial 1119441

#31 randomised controlled trial 1119441

#32 controlled trial 1375670

#33 #30 OR 31 OR #32 1738195

#34 #25 OR #26 OR #27 OR #28 OR #29 15180

#35 #24 AND #33 AND #34 297

#36 #35 (limited from May 2020 to Apr 2022) 54

#37 #35 (limited from April 2022 to Apr 2024) 57
