# Supplementary material for: The Effectiveness of Mulligan's Techniques in Non‐Specific Neck Pain: A Systematic Review and Meta‐Analysis
Source: Physiother Res Int. 2025 May 29;30(3):e70045. doi: 10.1002/pri.70045 (PMC12121345; doi:10.1002/pri.70045)
Supplement: Supplementary file 2 — Supporting Information S2 [file PRI-30-e70045-s008.docx]

**Appendix 2. Cut-off points to determine the clinical significance of outcomes included in the present review.**

| Outcomes | Clinical significance of results |
| --- | --- |
| Pain | |
| VAS | 2.5 points^1^ |
| NPRS |  |
| CROM | |
| Goniometer | |
| Extension^2^  Flexion^2^  Left lateral flexion^2^  Right lateral flexion^2^  Left rotation^2^  Right rotation^3^ | 5.6°  5.8°  4.2°  3.7°  4.1°  4.8°^3^ |
| CROM device | |
| Extension^4^ | 3.0° |
| Flexion^4^ | 4.1° |
| Left lateral flexion^4^ | 3.9° |
| Right lateral flexion^4^ | 2.5° |
| Left rotation^4^ | 2.9° |
| Right rotation^4^ | 3.3° |
| Inclinometer |  |
| Extension^5^ | 4.0º |
| Flexion^5^ | 6.0º |
| Left lateral flexion^5^ | 5.0° |
| Right lateral flexion^5^ | 3.0° |
| Left rotation^5^ | 5.0° |
| Right rotation^5^ | 10° |
| Disability | |
| NDI^1^ | 3.5 points (out of 50) |

CROM: cervical range of motion; NDI: neck disability index; NPRS: numerical pain rating scale; VAS: visual analogue scale

**References**:

1. Pool JJ, Ostelo RW, Hoving JL, Bouter LM, de Vet HC. Minimal clinically important change of the Neck Disability Index and the Numerical Rating Scale for patients with neck pain. *Spine*. Dec 15 2007;32(26):3047-51. doi:10.1097/BRS.0b013e31815cf75b

2. Armijo-Olivo S, de Castro-Carletti EM, Calixtre LB, de Oliveira-Souza AIS, Mohamad N, Fuentes J. Understanding Clinical Significance in Rehabilitation: A Primer for Researchers and Clinicians. *Am J Phys Med Rehabil*. Jan 1 2022;101(1):64-77. doi:10.1097/PHM.0000000000001799

3. Piva SR, Erhard RE, Childs JD, Browder DA. Inter-tester reliability of passive intervertebral and active movements of the cervical spine. *Man Ther*. Nov 2006;11(4):321-30. doi:10.1016/j.math.2005.09.001

4. Fletcher JP, Bandy WD. Intrarater reliability of CROM measurement of cervical spine active range of motion in persons with and without neck pain. *J Orthop Sports Phys Ther*. Oct 2008;38(10):640-5. doi:10.2519/jospt.2008.2680

5. Jorgensen R, Ris I, Juhl C, Falla D, Juul-Kristensen B. Responsiveness of clinical tests for people with neck pain. *BMC Musculoskelet Disord*. Dec 28 2017;18(1):548. doi:10.1186/s12891-017-1918-1
