# Supplementary material for: The Effectiveness of Mulligan's Techniques in Non‐Specific Neck Pain: A Systematic Review and Meta‐Analysis
Source: Physiother Res Int. 2025 May 29;30(3):e70045. doi: 10.1002/pri.70045 (PMC12121345; doi:10.1002/pri.70045)
Supplement: Supplementary file 3 — Supporting Information S3 [file PRI-30-e70045-s007.docx]

**Appendix 3. List of excluded studies.**

| Year | Title | Authors | Reason of exclusion |
| --- | --- | --- | --- |
| 2006 | Manual therapy, physical therapy, or continued care by a general practitioner for patients with neck pain - A randomized, controlled trial | Hoving, J. L.; Koes, B. W.; de Vet, H. C. W.; van der Windt, Dawm; Assendelft, W. J. J.; van Mameren, H.; Deville, Wljm; Pool, J. J. M.; Scholten, Rjpm; Bouter, L. M. | Wrong intervention |
| 2007 | Stretching exercises vs manual therapy in treatment of chronic neck pain: a randomized, controlled cross-over trial | Ylinen, J.; Kautiainen, H.; Wirén, K.; Häkkinen, A. | Wrong intervention |
| 2007 | Effect of manual therapy and stretching on neck muscle strength and mobility in chronic neck pain | Hakkinen, A.; Salo, P.; Tarvainen, U.; Wiren, K.; Ylinen, J. | Wrong intervention |
| 2008 | The effectiveness of manual physical therapy and exercise for mechanical neck pain: a randomized clinical trial | Walker, M. J.; Boyles, R. E.; Young, B. A.; Strunce, J. B.; Garber, M. B.; Whitman, J. M.; Deyle, G.; Wainner, R. S. | Wrong intervention |
| 2008 | Relation between changes in posteroanterior stiffness and active range of movement of the cervical spine following manual therapy treatment | Tuttle, N.; Barrett, R.; Laakso, L. | Wrong intervention |
| 2011 | Primary care randomized clinical trial: Manual therapy effectiveness in comparison with TENS in patients with neck pain | Escortell-Mayor, E.; Riesgo-Fuertes, R.; Garrido-Elustondo, S.; Asunsolo-del Barco, A.; Diaz-Pulido, B.; Blanco-Diaz, M.; Bejerano-Alvarez, E. | Wrong intervention |
| 2012 | Upper Cervical and Upper Thoracic Thrust Manipulation Versus Nonthrust Mobilization in Patients With Mechanical Neck Pain: A Multicenter Randomized Clinical Trial | Dunning, James; Cleland, Joshua | Wrong intervention |
| 2013 | Short-Term Combined Effects of Thoracic Spine Thrust Manipulation and Cervical Spine Nonthrust Manipulation in Individuals With Mechanical Neck Pain: A Randomized Clinical Trial | Masaracchio, Michael; Cleland, Joshua; Hellman, Madeleine; Hagins, Marshall | Wrong intervention |
| 2013 | The Effects of Thoracic Manipulation Versus Mobilization for Chronic Neck Pain: a Randomized Controlled Trial Pilot Study | Thavatchai, Suvarnnato; Rungthip, Puntumetakul; Kaber, David; Boucaut, Rose; Yodchai, Boonphakob; Preeda, Arayawichanon; Uraiwan, Chatchawan | Wrong intervention |
| 2014 | A comparison of two non-thrust mobilization techniques applied to the C7 segment in patients with restricted and painful cervical rotation | Creighton, Doug; Gruca, Mark; Marsh, Douglas; Murphy, Nancy | Wrong intervention |
| 2014 | Ist eine (Technik) besser als die andere? Eine randomisierte klinische Studie ber Manuelle Therapie bei Patienten mit chronischem Nackenschmerz [German] | Schomacher, Jochen | Wrong intervention |
| 2015 | Manual Therapy, Therapeutic Patient Education, and Therapeutic Exercise, an Effective Multimodal Treatment of Nonspecific Chronic Neck Pain: A Randomized Controlled Trial | Beltran-Alacreu, H.; Lopez-de-Uralde-Villanueva, I.; Fernandez-Carnero, J.; La Touche, R. | Wrong intervention |
| 2016 | Physiotherapy reduced recurrent headaches linked to neck pain and dysfunction in older adults | Gavrilescu, Gabriel | Not RCT |
| 2016 | Myofascial Release Therapy in the Treatment of Occupational Mechanical Neck Pain: A Randomized Parallel Group Study | Rodriguez-Fuentes, I.; De Toro, F. J.; Rodriguez-Fuentes, G.; de Oliveira, I. M.; Meijide-Failde, R.; Fuentes-Boquete, I. M. | Wrong intervention |
| 2016 | Myofascial Release Therapy in the Treatment of Occupational Mechanical Neck Pain: A Randomized Parallel Group Study | Rodriguez-Fuentes, I.; De Toro, F. J.; Rodriguez-Fuentes, G.; de Oliveira, I. M.; Meijide-Failde, R.; Fuentes-Boquete, I. M. | Duplicate |
| 2016 | Three combinations of manual therapy techniques within naprapathy in the treatment of neck and/or back pain: a randomized controlled trial | Paanalahti, K.; Holm, L. W.; Nordin, M.; Hoijer, J.; Lyander, J.; Asker, M.; Skillgate, E. | Wrong intervention |
| 2016 | A Comparison of the Effects of Stabilization Exercises Plus Manual Therapy to Those of Stabilization Exercises Alone in Patients With Nonspecific Mechanical Neck Pain: A Randomized Clinical Trial | Celenay, S. T.; Akbayrak, T.; Kaya, D. O. | Wrong intervention |
| 2017 | The effect of normalizing the sagittal cervical configuration on dizziness, neck pain, and cervicocephalic kinesthetic sensibility: a 1-year randomized controlled study | Moustafa, I. M.; Diab, A. A.; Harrison, D. E. | Wrong intervention |
| 2017 | Manual therapy compared with physical therapy in patients with non-specific neck pain: A randomized controlled trial | Groeneweg, R.; van Assen, L.; Kropman, H.; Leopold, H.; Mulder, J.; Smits-Engelsman, B. C. M.; Ostelo, R. W. J. G.; Oostendorp, R. A. B.; van Tulder, M. W. | Wrong intervention |
| 2017 | Additional Effect of Static Ultrasound and Diadynamic Currents on Myofascial Trigger Points in a Manual Therapy Program for Patients With Chronic Neck Pain...A Randomized Clinical Trial | Dibai-Filho, Almir Vieira; De Oliveira, Alessandra Kelly; Girasol, Carlos Eduardo; Cancio Dias, Fabiana Rodrigues; De Jesus Guirro, Rinaldo Roberto | Wrong intervention |
| 2018 | The effectiveness of thoracic manipulation combined with conventional therapy for mechanical neck pain | Muralidharan CK, Selvi P, Kalaivani T, Nandhakumar R, Sivakumar C, Yatheendra Kumar G | Not RCT |
| 2018 | Seeing the site of treatment improves habitual pain but not cervical joint position sense immediately after manual therapy in chronic neck pain patients | Beinert, K.; Lutz, B.; Zieglgansberger, W.; Diers, M. | Wrong intervention |
| 2018 | Immediate effects of cervical mobilisations on global perceived effect, movement associated pain and neck kinematics in patients with non-specific neck pain. A double blind placebo randomised controlled trial | Lascurain-Aguirrebena, I.; Newham, D. J.; Casado-Zumeta, X.; Lertxundi, A.; Critchley, D. J. | Wrong intervention |
| 2019 | Short- or Long-Term Treatment of Spinal Disability in Older Adults With Manipulation and Exercise | Michele Maiers, Jan Hartvigsen, Roni Evans, Kristine Westrom, Qi Wang, Craig Schulz, Brent Leininger, and Gert Bronfort | Wrong intervention |
| 2019 | Endocrine response after cervical manipulation and mobilization in people with chronic mechanical neck pain: a randomized controlled trial | Antonio VALERA-CALERO, Enrique LLUCH GIRBÉS, Tomás GALLEGO-IZQUIERDO, Anneleen MALFLIET, Daniel PECOS-MARTÍN | Wrong intervention |
| 2019 | Is a combined programme of manual therapy and exercise more effective thant usual care in patients with non-specific chronic neck pain? | Lucia Domingues , Fernando Manuel Pimentel-Santos, Eduardo Brazete Cruz, Ana Cristina Sousa, Ana Santos, Ana Cordovil, Anabela Correia, Laura Sa Torres, Antonio Silva, Pedro Soares Branco and Jaime Cunha Branco | Wrong intervention |
| 2019 | Effectiveness of mobilisation of the upper cervical region and craniocervical flexor training on orofacial pain, mandibular function and headache in women with TMD. A randomised, controlled trial | Letícia Bojikian CALIXTRE, Bruno Leonardo da Silva GRÜNINGER, Melina Nevoeiro HAIK, Francisco ALBURQUERQUE-SENDÍN, Ana Beatriz OLIVEIRA1 | Wrong population |
| 2020 | Effectiveness of cervical mobilization and stretching exercise with or without upper thoracic mobilization on non-specific neck pain | Maryam Safdar, Nazia Sarfraz, Iqra Ishaq, Madiha Sabir, Shamim Mahmooda, Anum Bashir | Wrong intervention |
| 2024 | Effects of exercise combined with cervicothoracic spine self-mobilization on chronic non-specific neck pain | Sun et al., | Duplicate |
| 2008 | A comparative study between post isometric relaxation and isometric exercises in non-specific neck pain | Gupta et al., | Wrong intervention |
| 2015 | Short-term effect of sustained natural apophyseal glides on cervical joint position sense, pain and neck disability in patients with chronic neck pain | Tacci et al., | Duplicate |
| 2016 | A Randomized Clinical Trial of Multimodal Therapy and Mulligan’s Concept of Manual Therapy for Patients with Chronic Pain Syndrome Caused by Upper Cervical Spine Disorders | Pu et al., | Wrong population |
| 2008 | The effectiveness of self snags over conventional physiotherapy management in  chronic neck pain among computer professionals | Chhababa et al, | Not RCT |
| 2023 | Sustained Natural Apophyseal Glides and Thoracic Posture Correction Technique on Mechanical Neck Pain | https://trialsearch.who.int/Trial2.aspx?TrialID=IRCT20190717044238N5 | Protocol |
| 2023 | Therapeutic and stabilization exercises after manual therapy in patients with non-specific chronic neck pain: A randomised clinical trial | Demir, O.; Atici, E.; Torlak, M. S. | Wrong intervention |
| 2024 | Efficacy of Mulligan on electromyography activation of cervical muscles in mechanical neck pain: randomised experimental trial | Alshaymaa, S. Abd El-Azeim; Mariam, O. Grase | Duplicate |
| 2023 | Comparison of effectiveness of mulligan mobilization technique and cervical stabilization training in patients with chronic neck pain: A single-blinded randomized controlled trial | Hikmet Kocaman, Nazım Tolgahan Yıldız, Mehmet Canlı, Halil Alkan | Protocol |
| 2018 | Immediate and short-term effects of mulligan concept positional sustained natural apophyseal glides on an athletic young-adult population classified with mechanical neck pain: an exploratory investigation | Andrews, D. P.; Odland-Wolf, K. B.; May, J.; Baker, R.; Nasypany, A.; Dinkins, E. M. | Wrong population |
| 2008 | A comparative study between post isometric relaxation and isometric exercises in non-specific neck pain | Gupta, S.; Jaiswal, P.; Chhabra, D. | Wrong intervention |
| 2016 | A Randomized Clinical Trial of Multimodal Therapy and Mulliganâ€™s Concept of Manual Therapy for Patients with Chronic Pain Syndrome Caused by Upper Cervical Spine Disorders | Put, M.; Huber, J.; PieniÄ…Å¼ek, M.; GÄ…dek-Michalska, A.; SzczygieÅ‚, A. | Wrong population |
| 2021 | Clinical outcomes of maitland mobilization in patients with Myofascial Chronic Neck Pain: A randomized controlled trial | Shabbir, M.; Arshad, N.; Naz, A.; Saleem, N. | Wrong intervention |
| 2022 | Effect of mobilization with movement technique in athletes with cervicogenic headache | https://trialsearch.who.int/Trial2.aspx?TrialID=IRCT20230815059155N1 | Protocol |
| 2021 | Sustained Natural Apophyseal Glide and Deep Friction Massage in Patients With Cervicogenic Headache | https://clinicaltrials.gov/show/NCT05404113 | Protocol |
| 2022 | Sustained Neutral Apophyseal Glides Half Rotation Technique and Gong's Mobilizations in Cervicogenic Headache | https://clinicaltrials.gov/show/NCT05434468 | Protocol |
| 2023 | Immediate Effects of Suboccipital Muscle Release Combined With Sustained Natural Apophyseal Glides in Patients With Chronic Tension Type Headache | https://clinicaltrials.gov/show/NCT05883813 | Protocol |
| 2022 | Effectiveness of different physiotherapy interventions in the management of cervicogenic headache: a pilot randomized controlled trial | Rani, M.; Kaur, J. | Wrong population |
| 2020 | Efficacy of sustained natural apophyseal glides mulligan technique on mobility and function in patients with cervical spondylosis: An experimental study | Arul Pragassame, S.; Mohandas Kurup, V. K.; Kour, J. | Wrong population |
| 2017 | Effect of SNAGS Mulligan technique on chronic cervical radiculopathy: A randomized clinical trial | El-Sayed, W.; Mohamed, A. F. E.; El-Monem, G.; Ahmed, H. H. | Wrong population |
| 2021 | Effect of manual therapy with exercise in patients with chronic cervical radiculopathy: a randomized clinical trial | Alshami, A. M.; Bamhair, D. A. | Wrong intervention |
| 2021 | Pragmatic application of manipulation versus mobilization to the upper segments of the cervical spine plus exercise for treatment of cervicogenic headache: a randomized clinical trial | Lerner-Lentz, A.; O'Halloran, B.; Donaldson, M.; Cleland, J. A. | Wrong intervention |
| 2015 | Manual therapy for cervicogenic dizziness: Long-term outcomes of a randomised trial | Reid, S. A.; Callister, R.; Snodgrass, S. J.; Katekar, M. G.; Rivett, D. A. | Wrong population |
| 2016 | Three combinations of manual therapy techniques within naprapathy in the treatment of neck and/or back pain: a randomized controlled trial | Paanalahti, K.; Holm, L. W.; Nordin, M.; Hoijer, J.; Lyander, J.; Asker, M.; Skillgate, E. | Wrong population |
| 2016 | Addition of a Sagittal Cervical Posture Corrective Orthotic Device to a Multimodal Rehabilitation Program Improves Short- and Long-Term Outcomes in Patients With Discogenic Cervical Radiculopathy | Moustafa, I. M.; Diab, A. A.; Taha, S.; Harrison, D. E. | Wrong population |
| 2017 | The effect of normalizing the sagittal cervical configuration on dizziness, neck pain, and cervicocephalic kinesthetic sensibility: a 1-year randomized controlled study | Moustafa, I. M.; Diab, A. A.; Harrison, D. E. | Wrong population |
| 2015 | Mobilization versus manipulations versus sustain apophyseal natural glide techniques and interaction with psychological factors for patients with chronic neck pain: randomized controlled trial | Lopez-Lopez, A.; Alonso Perez, J. L.; Gonzalez Gutierez, J. L.; La Touche, R.; Lerma Lara, S.; Izquierdo, H.; Fernandez-Carnero, J. | Duplicate |
| 2017 | Manual therapy compared with physical therapy in patients with non-specific neck pain: A randomized controlled trial | Groeneweg, R.; van Assen, L.; Kropman, H.; Leopold, H.; Mulder, J.; Smits-Engelsman, B. C. M.; Ostelo, R. W. J. G.; Oostendorp, R. A. B.; van Tulder, M. W. | Wrong Intervention |
| 2016 | Physiotherapy reduced recurrent headaches linked to neck pain and dysfunction in older adults | Gavrilescu, Gabriel | Abstract |
| 2015 | Effectiveness of mobilization therapy and exercises in mechanical neck pain | G anesh, G. S.; Mohanty, P.; Pattnaik, M.; Mishra, C. | Duplicate |
| 2016 | Upper cervical and upper thoracic manipulation versus mobilization and exercise in patients with cervicogenic headache: a multi-center randomized clinical trial | Dunning, James R.; Butts, Raymond; Mourad, Firas; Ian, Young; PeÃ±as, Cesar Fernandez-de-las; Hagins, Marshall; Stanislawski, Thomas; Donley, Jonathan; Buck, Dustin; Hooks, Todd R.; Cleland, Joshua A.; Young, Ian; Fernandez-de-Las PeÃ±as, Cesar | Wrong population |
| 2024 | Additional Effect of Static Ultrasound and Diadynamic Currents on Myofascial Trigger Points in a Manual Therapy Program for Patients With Chronic Neck Pain...A Randomized Clinical Trial | Dibai-Filho, Almir Vieira; De Oliveira, Alessandra Kelly; Girasol, Carlos Eduardo; Cancio Dias, Fabiana Rodrigues; De Jesus Guirro, Rinaldo Roberto | Wrong intervention |
| 2016 | Additional Effects of a Physical Therapy Protocol on Headache Frequency, Pressure Pain Threshold, and Improvement Perception in Patients With Migraine and Associated Neck Pain: A Randomized Controlled Trial | Bevilaqua-Grossi, D.; Goncalves, M. C.; Carvalho, G. F.; Florencio, L. L.; Dach, F.; Speciali, J. G.; Bigal, M. E.; Chaves, T. C. | Wrong population |
| 2015 | Manual Therapy, Therapeutic Patient Education, and Therapeutic Exercise, an Effective Multimodal Treatment of Nonspecific Chronic Neck Pain: A Randomized Controlled Trial | Beltran-Alacreu, H.; Lopez-de-Uralde-Villanueva, I.; Fernandez-Carnero, J.; La Touche, R. | Wrong intervention |
| 2019 | Seeing the site of treatment improves habitual pain but not cervical joint position sense immediately after manual therapy in chronic neck pain patients | Beinert, K.; Lutz, B.; Zieglgansberger, W.; Diers, M. | Wrong intervention |
| 2014 | Mulligan sustained natural apophyseal glides and Maitland mobilisations for cervicogenic dizziness | Lawrence, Dj | Wrong population |
| 2019 | Effectiveness of mobilisation of the upper cervical region and craniocervical flexor training on orofacial pain, mandibular function and headache in women with TMD. A randomised, controlled trial | Calixtre, L. B.; Oliveira, A. B.; de Sena Rosa, L. R.; Armijo-Olivo, S.; Visscher, C. M.; Alburquerque-Sendin, F. | Wrong population |
| 2019 | A study on the effectiveness of manual mulligan traction compared to intermittent electrical traction in patients with cervical spondylosis | Arul Pragassame, S.; Mohandas Kurup, V. K.; Sivashanmugam, V. | Wrong population |
| 2021 | Pragmatic application of manipulation versus mobilization to the upper segments of the cervical spine plus exercise for treatment of cervicogenic headache: a randomized clinical trial | Lerner-Lentz, A.; O'Halloran, B.; Donaldson, M.; Cleland, J. A. | Wrong population |
| 2023 | The effectiveness of thoracic manipulation combined with conventional therapy for mechanical neck pain | Muralidharan, C. K.; Selvi, P.; Kalaivani, T.; Nandhakumar, R.; Sivakumar, C.; Yatheendra Kumar, G. | Not RCT |
| 2023 | Comparison of effectÄ±veness of mulligan mobilization technique and cervical stabilization training in patients with chronic neck pain: A single-blinded randomized controlled trial | ÅžekerÃ¶z, Serbay | Protocol |
| 2022 | Comparative Effects of Mulligan's Mobilization, Spinal Manipulation, and Conventional Massage Therapy in Cervicogenic Headache-A Prospective, Randomized, Controlled Trial | Nambi, G.; Alghadier, M.; Ebrahim, E. E.; Vellaiyan, A.; Tedla, J. S.; Reddy, R. S.; Kakaraparthi, V. N.; Aldhafian, O. R.; Alshahrani, N. N.; Saleh, A. K. | Wrong population |
| 2023 | Clinical efficacy of the mulligan maneuver for cervicogenic headache: a randomized controlled trial | Jin, X.; Du, H. G.; Kong, N.; Shen, J. L.; Chen, W. J. | Wrong population |
| 2018 | Is mulligan's sustained natural apophyseal glides (Snags) or muscle energy technique is effective in the non-surgical management of cervicogenic headache? a two-group pretest-posttest randomized controlled trial | Veena Kirthika, S.; Padmanabhan, K.; Sudhakar, S.; Vijaya Kumar, M. | Wrong population |
| 2021 | *Postural and clinical outcomes of sustained natural apophyseal glides treatment in cervicogenic dizziness patients: A randomised controlled trial* | Micarelli, A.; Viziano, A.; Granito, I.; Carlino, P.; Micarelli, R. X.; Augimeri, I.; Alessandrini, M. | Wrong population |
