# Supplementary material for: The Effectiveness of Mulligan's Techniques in Non‐Specific Neck Pain: A Systematic Review and Meta‐Analysis
Source: Physiother Res Int. 2025 May 29;30(3):e70045. doi: 10.1002/pri.70045 (PMC12121345; doi:10.1002/pri.70045)
Supplement: Supplementary file 4 — Supporting Information S4 [file PRI-30-e70045-s001.docx]

**Appendix 7.** Characteristics of the studies included in the present systematic review.

| **Study characteristics** | **Interventions characteristics** | **Treatment features**  **Follow-up** | **Outcomes of interest** (measurement tool) | **Results descriptions** | **Conclusion** |
| --- | --- | --- | --- | --- | --- |
| **First Author, Year:**  **Study 1A***  Lopez-Lopez et al, 2015(Lopez-Lopez et al., 2015)  **Country:** Spain  **Design:** RCT  **Setting:** Valleaguado Primary Health Care Centre in Coslada  **Sample size:** 48  **Age range:** 18-65 years  **Gender M/F:** 6/42  **Diagnosis:** Chronic nonspecific neck pain  **Diagnosis description:** Posterior neck pain from the superior nuchal line to the first thoracic spinous process for more than 12 weeks without radicular symptoms radiating to the head, trunk, and/or the upper limbs  **Funding:** None | **Main therapy:** SNAGs vs. Mobilization; SNAGs vs. HVLA  **Intervention (n = 17):**  SNAGs: applied to hypomobile and symptomatic intervertebral level; 3 x 10 repetitions.  **Comparator1 (n = 16):** Mobilizations: unilateral posteroanterior grade III passive oscillations at 2 Hz on hypomobile and symptomatic cervical vertebrae; 3 x 2 min with 1 min rest between sessions  **Comparitor2 (n = 15):**  HVLA: high-velocity, low-amplitude manipulations at hypomobile zygapophyseal joints in the cervical spine | Treatment features for all groups: 1 session  Session duration: NR  Follow-up: None | **Pain intensity** (VAS at rest and during active movements in flexion/  extension, rotation, and lateral flexion)  **Cervical ROM** (CROM device) | Pain intensity at rest: Significant two-way treatment x time interaction (F(2, 24)=3.69, P=0.040, ηp^2^=0.23) and greater effectiveness (Cohen’s d) of manipulation (4.53) over mobilization (2.87), and of both over SNAG (0.45)  Pain intensity in flexion/extension: Significant three-way treatment x anxiety x time interaction (F (2,24) =6.65, P=0.005, ηp^2^=0.36)  Cervical ROM: No technique seemed statistically superior over the others (p > 0.05) | The authors only reported the within-comparisons for each group but did not compare groups between them.  All manual therapy techniques studied produced decreased pain during active movement, increased cervical range of motion, and local hypoalgesic effects. However, only the manipulation and mobilization groups experienced pain relief at rest. Under high anxiety levels, a better outcome is expected after mobilization intervention. Under low anxiety levels, a better prognosis is expected after manipulation and SNAG technique intervention. |
| **First Author, Year:**  **Study 1B***  Izquierdo et al, 2014(Izquierdo Perez et al., 2014)  **Country:** Spain  **Design:** RCT  **Setting:** Valleaguado Primary Health Care Centre in Coslada  **Sample size:** 61  **Age range:** 20-65 years  **Gender M/F:** 35/26  **Diagnosis:** Chronic nonspecific neck pain  **Diagnosis description:** Posterior neck pain from the superior nuchal line to the first thoracic spinous process for more than 12 weeks without radicular symptoms radiating to the head, trunk, and/or the upper limbs  **Funding:** NR | **Main therapy:** SNAGs vs. Mobilization; SNAGs vs. HVLA  **Intervention (n = 21):**  SNAGs: on the hypomobile and painful intervertebral level; 3 x 10 repetitions  **Comparator1 (n = 21):** Mobilizations: unilateral posteroanterior oscillatory pressure at 2 Hz on hypomobile cervical vertebrae; 3 x 2 min with 1 min rest between mobilizations  **Comparator2 (n = 19):**  HVLA in the most limited movement, lateral flexion, or rotation | Treatment features for all groups: 2 weeks**,** 4 sessions  Session duration: NR  Follow-up: At 1-, 2- and 3-months post-treatment | **Pain intensity** (VAS)  **Neck disability** (NDI)  **Cervical ROM** (CROM device) | Pain intensity (at rest): Significant effect for time (F = 10.38, P < 0.0001, Partial eta = 0.54) but not for group x time interaction (F = 0.12, P < 0.83, Partial eta = 0.06). In general, it decreased for all groups  Neck disability: No differences between groups from pre-treatment to the 3^rd^ month. Significant effect for time (F = 33.56, P < 0.0001, Partial eta = 0.37) but not for group x time interaction (F = 1.03, P = 0.41, Partial eta = 0.03)  Cervical ROM: Significant effect for time for all movements (p < 0.0001) but no group x time interactions were identified for any movement (P > 0.05) except extension (Extension F = 1.95, P = 0.02, Partial eta = 0.06). The HVLA group had greater increases in extension (P < 0.01) than the Mobilizations and SNAGs (P > 0.05) groups in all the follow-up months. Mobilization increased extension movement immediately (P < 0.01) but not during the follow-up (P > 0.05). SNAG increased only during the one-month follow-up (P < 0.01) | The results conclude that there is no long-term difference in pain, disability, and cervical range of motion between the application of HVLA, mobilizations, and SNAGs for patients with chronic neck pain. Any one of the three techniques is as effective as the others. |
| **First Author, Year:** Tachii et al, 2015(Tachii et al., 2015)  **Country:** NR  **Design:** RCT  **Setting:** NR  **Sample size:** 30  **Age range:** 25-50 years  **Gender M/F:** NR  **Diagnosis:** Chronic nonspecific neck pain  **Diagnosis description:** Neck pain for more than 3 months  **Funding:** self-financed | **Main therapy:** SNAGs + CT vs. CT  **Intervention (n = 15):**  SNAGs: gliding force on the spinous process of the vertebra above the suspected lesion; 3 x 6-10 repetitions, plus 15 min hot pack, plus neck isometric exercises in flexion, extension, rotation, and side bending at 80% maximum strength; 15 x 10 sec  **Comparator (n = 15):**  CT: 15 min hot pack plus neck isometric exercises in flexion, extension, rotation, and side bending at 80% of maximum strength; 15 x 10 sec | Treatment features for all groups: 2 weeks**,** 3 sessions/week  Session duration: NR  Follow-up: None | **Pain intensity** (NPRS)  **Neck disability** (NDI) | Pain intensity: No statistically significant difference post-treatment (p > 0.05) between SNAGs group (MD = 2.21 + 0.89) and CT group (MD = 1.93 + 1.37)  Neck disability: No statistically significant difference post-treatment (p > 0.05) between SNAGs group (MD = 7.07 + 4.42) and CT group (MD = 5.72 + 5.15) | SNAGs mobilization demonstrates some improvement in pain and neck disability in patients with chronic neck pain. |
| **First Author, Year:** Buyukturan et al, 2018(Buyukturan et al., 2018)  **Country:** Turkey  **Design:** RCT  **Setting:** Physical therapy and Rehabilitation Center of Ahi Evran University  **Sample size:** 40  **Age range:** > 65 years  **Gender M/F:** NR  **Diagnosis:** Chronic nonspecific neck pain  **Diagnosis description:** Neck pain for at least 3 months having no neurological, rheumatological, or musculoskeletal problems  **Funding:** NR | **Main therapy:** NAGs + SNAGs + CT vs. CT  **Intervention (n = 21):** Mulligan Mobilization: NAGs on C2 to C7; 6 repetitions plus SNAGs; 6 times/session, plus heat, TENS, ultrasound, massage on cervical and thoracic regions, and cervical ROM and posture exercises  **Comparator (n = 19):**  CT: Heat, TENS, US, massage on cervical and thoracic regions, cervical ROM and posture exercises | Treatment features for all groups: 2 weeks**,** 5 sessions/week  Session duration: NR  Follow-up: None | **Pain intensity** (VAS)  **Neck disability** (NDI)  **Cervical ROM** (goniometer) | Pain intensity at rest: Similar improvement (p = 0.862) in the Mulligan group (Delta median [IQR] = -4 [-6 to -2]) and CT group (Delta median [IQR] = -3 [-6 to -3])  Pain intensity during activity: Similar improvement (p = 0.083) in the Mulligan group (Delta median [IQR] = -6 [-6 to -3]) and CT group (Delta median [IQR] = -5 [-5 to -4])  Neck disability: Similar improvement (p = 0.335) in the Mulligan group (Delta median [IQR] = -13 [-14 to -7]) and CT group (Delta median [IQR] = -10 [-12 to -8])  Cervical ROM: Similar improvement for Mulligan group compared to CT group for left lateral flexion (Delta median [IQR] = 6 [5.4-8.2] vs. 5 [3.5-6.8]; p = 0.089), right rotation (Delta median [IQR] = 7 [5.6-8.3] vs. 3 [2.7-4.7]; p = 0.527), and left rotation (Delta median [IQR] = 13 [10.5-15.6] vs. 3 [2.9-4.5]; p = 0.354)  Higher improvement for Mulligan group compared to CT group for flexion (Delta median [IQR] = 10.2 [8.3-12.4] vs. 6.4 [4.2-6.9]; p < 0.001), extension (Delta median [IQR] = 8.4 [5.8-9.7] vs. 5.3 [3.7-6.4]; p < 0.001), and right lateral flexion (Delta median [IQR] = 9 [8.01-11.2] vs. 6 [4.4-7.1]; p = 0.004) | Applying NAGs and SNAGs in older adults with neck pain positively affects pain, ROM, and functional level. |
| **First Author, Year:** Kumar et al, 2011(Kumar et al., 2011)  **Country:** India  **Design:** RCT  **Setting:** Guru Nanak Dev University  **Sample size:** 100  **Age range:** > 30 years  **Gender M/F:** NR  **Diagnosis:** Chronic nonspecific neck pain  **Diagnosis description:** Local spinal pain and/or joint stiffness between C3-C7 with no radiating pain in the upper limbs  **Funding:** NR | **Main therapy:** NAGs vs. placebo  **Intervention (n = 25):**  NAGs: 3 x 30 sec (1-2 movements/second), hot packs, active and isometrics strengthening exercises, and home exercises  **Comparator1 (n = 25):**  Day 1 to 6: NAGs (3 x 30 sec, 1-2 movements/second)  Day 7 to 12: placebo, hot packs (12min), active and isometrics strengthening exercises, and home exercises  **Comparator2 (n = 25):**  Day 1 to 6: placebo  Day 7 to 12: NAGs (30 sec), hot packs, active and isometrics strengthening exercices, and home exercises  **Comparator3 (n = 25):**  Placebo plus hot packs, active and isometrics strengthening exercises, and home exercises | Treatment features for all groups: 12 days  Session duration: NR  Follow-up: Day 42 (30 days after treatment) | **Pain intensity** (VAS)  **Cervical ROM** (dual inclinometer) | Pain intensity: Significant difference between the groups treated with NAGs and sham intervention for flexion (F_3,95_ = 5.45; p < 0.01), extension (F_3,95_ = 6.83; p < 0.01), left rotation (F_3,95_ = 6.59; p < 0.01), and right rotation (F_3,95_ = 6.15; p < 0.01) in favor of the NAGs groups (between pre-treatment and the average of the post-treatment and follow-up observations)  Cervical ROM: Significant difference between the groups treated with NAGs and sham intervention for flexion (F_3,95_ = 4.50; p < 0.01), extension (F_3,95_ = 15.41; p < 0.01), left side flexion (F_3,95_ = 6.16; p < 0.01) in favor of the NAGs groups (between pre-treatment and the average of the post-treatment and follow-up observations), but not for right side flexion (F_3,95_ = 1.57; p > 0.05) | The results show NAGs effectively improve ROM and reduce pain at the end range in patients with cervical pain and stiffness. |
| **First Author, Year:** Duymaz et al, 2018(Duymaz & Yagci, 2018)  **Country:** Turkey  **Design:** RCT  **Setting:** NR  **Sample size:** 40  **Age range:** 25-50 years  **Gender M/F:** 5/35  **Diagnosis:** Chronic nonspecific neck pain  **Diagnosis description:** Mechanical neck pain characterized by spasms of cervical muscles caused by trauma or posture dysfunction  **Funding:** None | **Main therapy:** SNAGs + self-SNAGs + Exercises vs. Exercises  **Interventions (n = 20):**  SNAGs (3 x 10 repetitions), self-SNAGs (3 x 10 repetitions, 3 times/day), and home exercise program (see below)  **Comparator (n = 20):**  Home exercise program: ROM and stretching exercises; 3 x 10 repetitions, 3 times/day | Treatment features for all groups: 2 weeks**,** 5 sessions/week  Session duration: NR  Follow-up: 1- and 3-months post-treatment | **Cervical ROM** (goniometer)  **Pain intensity** (VAS)  **Neck disability** (NDI) | Cervical ROM: Statistically significant difference (p = 0.0001) at 3 months post-treatment between Mulligan group and control group for flexion (MD = 19.9 + 8.86 vs. 0.1 + 2.04), extension (MD = 11.85 + 3.67 vs. -0.65 + 1.59), lateral flexion (MD = 9.55 + 4.91 vs. -0.4 + 2.11), and rotation (MD = 12.25 + 5.42 vs. 0.45 + 2.79), in favor of Mulligan group  Pain intensity: Favorable change was reported in the Mulligan Mobilization group (MD = -5.81 + 1.44; p = 0.0001) and the control group (MD = -1 + 1.7; p < 0.05) after treatment.  Statistically significant difference (p = 0.0001) at 3 months post-treatment between Mulligan group (MD = -5.65 + 2.02) and control group (MD = -2.25 + 2.05), in favor of Mulligan group  Neck disability: Favorable change was reported in the Mulligan Mobilization group (MD = -12.1 + 4.5; p = 0.0001) and the control group (MD = -2 + 5.12; p < 0.05) after treatment.  Statistically significant difference (p = 0.0001) at 3 months post-treatment between Mulligan group (MD = -11.15 + 6.25) and control group (MD = -0.5 + 1), in favor of Mulligan group | The results of this study showed that the Mulligan Mobilization treatment program has positive effects on pain, ROM, and disability in participants with mechanical neck pain. |
| **First Author, Year:** Ali et al, 2014(Ali et al., 2014)  **Country:** Pakistan  **Design:** RCT  **Setting:** Outpatient Department of Physiotherapy and Rehabilitation, Khyber Teaching Hospital Peshawar  **Sample size:** 102  **Age range:** NR  **Gender M/F:** NR  **Diagnosis:** Chronic nonspecific neck pain  **Diagnosis description:** NR  **Funding:** NR | **Main therapy:** SNAGs + exercises vs. SNAGs  **Interventions (n = 51):**  SNAGs plus IETP  **Comparator (n = 51):** SNAGs | Treatment features for all groups: 6 weeks, 4 sessions/week  Session duration: NR  Follow-up: None | **Neck disability** (NDI)  **Pain intensity** (VAS) | Neck disability: Group A demonstrated better improvement in NDI (MD = -26; p = 0.003) than Group SNAGs (MD = -12; p = 0.264)  Pain intensity: Group SNAGs plus IETP improved better VAS (MD = -5; p = 0.013) than group SNAGs (MD = -4; p = 0.147) | The authors reported only within-group comparisons, without inter-group analysis. SNAGs followed by exercises reduce pain and improve function more effectively than SNAGs alone. |
| **First Author, Year:** Rezkallah et al, 2018(Rezkallah & Abdullah, 2018)  **Country:** Egypt  **Design:** RCT  **Setting:** Outpatient clinic of the School of Physical Therapy, Cairo University  **Sample size:** 70  **Age range:** 25-45 years  **Gender M/F:** 30/40  **Diagnosis:** Chronic nonspecific neck pain  **Diagnosis description:** Nonspecific neck pain with or without associated neurological symptoms, with a duration of at least 3 weeks and of not more than 6 months  **Funding:** NR | **Main therapy:** SNAGs + exercises vs. MFR; SNAGs + exercises vs. exercises  **Intervention (n = 25):**  SNAGs: anterosuperior accessory glides through the spinous process or articular pillar of the vertebra above the site of the problem (3 x 6-10 repetitions) plus isometric exercise (4 x 15 repetitions), stretching (2-3 x 30 sec), straightening (5 x 3-5 sec) 5 times/week for all  **Comparator1 (n = 23):**  MFR: trigger point pressure release for 30 sec; stretching for 1 min plus isometric exercise (4 x 15 repetitions), stretching (2-3 x 30 sec), straightening (5 x 3-5 sec) 5 times/week for all  **Comparator2 (n = 22):** Exercises (same as above) | Treatment features for all groups: 4 weeks**,** 3 sessions/week  Session duration: NR  Follow-up: None | **Cervical ROM** (goniometer)  **Neck disability** (NDI)  **Pain intensity** (VAS) | Cervical ROM: All groups showed significant improvement in neck ROM (p<0.0001); however, the SNAGs group reported the highest percentage increase in neck ROM (16% for flexion, 36.6% for extension, 24.5% for right side bending, 26.1% for left side bending, 12.1% for right rotation, and 12.7% for left rotation) among the 3 groups  Neck disability: All groups showed significant improvement in NDI (p<0.0001); however, the SNAGs group reported the highest percentage decrease in NDI scores (69%) among the 3 groups  Pain intensity: All groups showed significant improvement in pain (p<0.0001); however, the SNAGs group reported the highest percentage decrease in VAS scores (65.2%) among the 3 groups  **SNAGs + exercises** are statistically superior to exercises only for all outcomes (p<0.0001)  **MFR + exercises** are statistically superior to exercises only for ROM (p<0.001) and NDI (p<0.0001) but not for VAS (p=0.26)  **SNAGs + exercises** are statistically superior to MFR + exercises only for NDI (p=0.002) | The combination of SNAGs or MFR with exercises provides short-term benefits for pain, ROM, and function in nonspecific neck pain. Based on this study, therapists may consider including SNAGs mobilization or MFR with exercises in care plans for individuals with chronic nonspecific neck pain. |
| **First Author, Year:** Tank et al, 2018(Tank et al., 2018)    **Country:** India  **Design:** RCT  **Setting:** Various outpatient physiotherapy departments  **Sample size:** 40  **Age range:** 18-45 years  **Gender M/F:** NR  **Diagnosis:** Acute nonspecific neck pain  **Diagnosis description:** Mechanical neck pain for less than 3 months  **Funding:** NR | **Main therapy:** SNAGs + CT vs. MET + CT  **Intervention (n = 20):**  SNAGs: gliding force applied to spinous process or articular pillar during flexion, extension, rotation, or lateral flexion; 6-10 repetitions) plus CT (moist heat pack + isometric neck exercises 10-15 x 10 sec)  **Comparator (n = 20):**  MET: side bending and rotation: isometric contraction 5 sec plus relaxation 5 sec; 2-3 times plus CT (moist heat pack + isometric neck exercises 10-15 x 10 sec) | Treatment features for all groups: 2 weeks**,** 6 days/week  Session duration: NR  Follow-up: None | **Pain intensity** (VAS)  **Neck disability** (NDI)  **Cervical ROM** (NR) | Pain intensity: No significant difference between the Mulligan group (Mean = 4.3 + 0.4) and MET group (Mean = 3.73 + 0.71) (p = 0.16)  Neck disability: No significant difference between the Mulligan group (Mean = 18.52 + 6.08) and MET group (Mean = 18.05 + 7.4) (p = 0.571)  Cervical ROM: No significant difference between the Mulligan group and MET group, for any movement (p > 0.125) | Muscle energy technique and Mulligan SNAGs are equally effective in reducing pain and disability and increasing ROM. |
| **First Author, Year:** Ganesh et al, 2014(Ganesh et al., 2015)  **Country:** India  **Design:** RCT  **Setting:** NR  **Sample size:** 60  **Age range:** NR  **Gender M/F:** 38/22  **Diagnosis:** Acute nonspecific neck pain  **Diagnosis description:** Insidious onset of neck pain for less than 12 weeks, reduced ROM in extension, side flexion, and rotation, and neck symptoms reproduced during passive accessory movements  **Funding:** NR | **Main therapy:** SNAGs + exercises vs. Mobilization; SNAGs + exercises vs. exercises  **Intervention (n = 20):**  SNAGs: gliding force on the spinous process or articular pillar of the upper vertebra in the implicated functional-spinal unit; 6 repetitions, plus exercises (cervical and scapular muscle stretching, deep neck flexor strengthening, isometric exercises for extensors, side flexors, and rotators, anti-gravity strengthening for rhomboids and trapezius, and cervical ROM exercises; 1 x 10 repetitions with 6-sec hold and 10-sec rest between repetitions) plus home exercises (instruction to continue strengthening and stretching at home for four weeks)  **Comparator1 (n = 20):**  Maitland mobilizations: posteroanterior oscillatory pressure at a rate of 2-3 oscillations per second and a frequency of 3-4 mobilizations during 30 sec, on the facet or the spinous process of the hypomobile vertebras plus exercises (same as above) plus home exercises (same as above)  **Comparator2 (n = 20):** Exercises (same as above) plus home exercises (same as above) | Treatment features for all groups: 2 weeks**,** 5 sessions/week  Session duration: NR  Follow-up: 12 weeks post-treatment | **Pain intensity** (VAS)  **Cervical ROM** (goniometer)  **Neck disability** (NDI) | All groups showed improvement over time compared to baseline for each outcome (p < 0.01)  No significant differences between groups, for all outcomes (p > 0.056)  No significant group x time interaction effects across groups in improving outcomes (p > 0.199)  The effect sizes between the groups were small (0.2) revealing minimal clinically detectable difference between the mobilization and exercise groups after intervention and at follow-up | The results of this study suggest that supervised exercises are as effective as mobilization and exercises combined in reducing neck pain and improving ROM and related disability among participants with acute neck pain. |
| **First Author, Year:** Manzoor et al, 2021(Manzoor et al., 2021)  **Country:** Pakistan  **Design:** RCT  **Setting:** Physiotherapy Department of Mayo Hospital  **Sample size:** 56  **Age range:** 20-40 years  **Gender M/F:** NR  **Diagnosis:** Chronic nonspecific neck pain  **Diagnosis description:** Mechanical neck pain  **Funding:** None | **Main therapy:** SNAGs and NAGs vs MET post-isometric relaxation  **Intervention (n = 28):**  MMT: oscillatory moves with <6 repeats  **Comparator (n = 28):**  MET: applied to the sternocleidomastoid and upper trapezius muscles by holding the movement for 5-10 seconds and maintaining the stretch for 30 sec. Each maneuver was repeated 3-5 times per treatment session | Treatment features for all groups: 3 weeks, 2 sessions/week  Session duration: NR  Follow-up: None | **Pain intensity** (VAS)  **Neck disability** (NDI)  **Cervical ROM** (goniometer) | Pain intensity: Improved significantly more in Mulligan group (MD = -4.86 + 1.07) than in MET group (MD = -3.6 + 1.15) (p = 0.03)  Neck disability: Improved significantly more in the Mulligan group than in the MET group (p < 0.01)  Cervical ROM: Only ROM in extension improved significantly more in the Mulligan group (MD = 17.75 + 8.55) than in the MET group (MD = 13.43 + 7.15) (p = 0.001) | MMT significantly affected pain intensity and functional neck status. For ROM, only extension was significantly improved by MMT. |
| **First Author, Year:** Shamsi et al, 2021(Shamsi et al., 2021)  **Country:** Saudi Arabia  **Design:** RCT  **Setting:** Raj Nursing Home  **Sample size:** 100  **Age range:** 20-45 years  **Gender M/F:** NR  **Diagnosis:** Chronic nonspecific neck pain  **Diagnosis description:** Localized pain or stiffness in the spine or both combined between C3 and C7 without upper‑limb radiculopathy, and Pain reported on VAS score ˃3/10 in the neck region for more than 3 months  **Funding:** None | **Main therapy:** SNAGs + CT vs US + CT  **Intervention (n = 50):**  SNAGs (3x6 repetitions) plus CT (moist hot pack for 15 min, neck stretching - upper trapezius, neck rotators; 30s holding, 30 sec relaxing for 3 times both sides, and strengthening exercises - isometric for neck extensors, flexors, rotators, and neck side bending muscles; 10 x 10-sec hold per session)  **Comparator (n = 50):**  US: 1 MHz of frequency and 1.0 W/cm² of intensity and on continuous mode for 10 min, on bilateral trapezius trigger points, in a circular motion) plus CT (same as above) | Treatment features for all groups: 2 weeks, 3 sessions/week  Session duration: NR  Follow-up: None | **Pain intensity** (VAS)  **Neck disability** (NDI)  **Cervical ROM** (goniometer) | Pain intensity: Statistically significant difference between groups with a lower VAS in the Mulligan group (MD = -6.04 + 0.88) than in the US group (MD = -5.66 + 1.08) (p = 0.032)  Neck disability: Statistically significant difference between groups with a lower NDI in the Mulligan group (MD = -18.76 + 4.95) than in the US group (MD = -12.94 + 6.91) (p < 0.001)  Cervical ROM: Statistically significant difference between groups with a higher cervical ROM in Mulligan group than in US group in neck flexion (MD = -21.26 + 3.71 vs. 12.54 + 3.81), extension (MD = 19.58 + 4.01 vs. 12.28 + 2.86), right side flexion (MD = 14.42 + 2.78 vs. 12.58 + 3.66), left side flexion (MD = 14.66 + 2.75 vs. 12.52 + 3.24), right rotation (MD = 21.74 + 5.74 vs. 12.02 + 7.21), and left rotation (MD = 23.06 + 4.93 vs. 13.46 + 6.22) (p < 0.001 for each movement) | The SNAGs group showed better improvements in the intensity of pain, neck disability, and ROM compared to the US group in patients with mechanical neck pain, as assessed through the VAS, NDI, and goniometry. |
| **First Author, Year:** Alshami et al, 2021(Alshami & AlSadiq, 2021)  **Country:** Saudi Arabia  **Design:** RCT  **Setting:** Hospital (Department of physical therapy)  **Sample size:** 40  **Age range:** 25-50 years  **Gender M/F:** 9/31  **Diagnosis:** Chronic nonspecific neck pain  **Diagnosis description:** Neck pain for 3 months or longer before the study, and scored 5 or more on the NDI  **Funding:** None | **Main therapy:** MWM + neck and scapulothoracic exercises + taping vs. Neck and scapulothoracic exercises + taping  **Intervention (n = 20):**  MWM for scapula (neck movements with corrective gliding force to reposition the scapula to the optimal position; 3 x 6-10 repetitions) plus neck and scapulothoracic exercises (cervical and scapular retraction, deep neck flexor strengthening, active ROM exercises of the neck in all directions; 10 x 10 sec, 5x/day) plus taping (I-shaped elastic tape applied to the belly of the upper trapezius)  **Comparator (n = 20):**  Neck and scapulothoracic exercises (same as above) plus taping (same as above) | Treatment features for all groups: 2-3 weeks, 2-3 sessions/ week  Session duration: 30-60 minutes  Follow-up: None | **Pain intensity** (VAS)  **Cervical ROM** (dual inclinometers)  **Neck disability** (NDI) | Pain intensity: No statistical difference between Mulligan group (MD = -3.1 + 1.5) and comparison group (MD = -1.8 + 1.95) at the end of the treatment (p = 0.068)  Cervical ROM: No statistical difference between the Mulligan group and the comparison group at the end of the treatment for flexion (MD = 0.5 + 8.32 vs. 3.4 + 9.49), extension (MD = 9.1 + 11.05 vs. 13 + 9.22), right rotation (MD = 6.2 + 7.87 vs. 9.2 + 9.22), left rotation (MD = 3.5 + 7.7 vs. 6.3 + 11.44), right side bending (MD = 5.3 + 6.37 vs. 6.1 + 7.28), and left side bending (MD = 4.7 + 6.45 vs. 7.3 + 8.97) (p > 0.280)  Neck disability: No statistical difference between Mulligan group (MD = -9.6 + 10.3) and comparison group (MD = -12.3 + 11.75) at the end of the treatment (p = 0.933) | Pain and disability improved similarly in patients with chronic neck pain accompanied by scapular dyskinesis in both the MWM and comparison groups. The addition of scapulothoracic MWM did not enhance the outcomes of the exercise and corrective tape treatment regimen during the 3-week period. |
| **First Author, Year:** Alansari et al, 202(Alansari et al., 2021)  **Country:** Saudi Arabia  **Design:** RCT  **Setting:** Rehabilitative Clinic, King Abdulaziz Hospital and East Jeddah General Hospital  **Sample size:** 44  **Age range:** 25-45 years  **Gender M/F:** 3/41  **Diagnosis:** Subacute and chronic nonspecific neck pain  **Diagnosis description:** Neck pain for more than one week without radicular symptoms that extended to the posterior aspect of the neck from the superior nuchal line to the first thoracic vertebra  **Funding:** None | **Main therapy:** SNAGs vs. Maitland mobilizations  **Intervention (n = 22):**  SNAG: gliding force on the spinous process or facet joint of the superior vertebra (3 x 10 repetitions)  **Comparator (n = 22):**  Maitland mobilizations: passive spinal segmental mobilization with low-velocity, small or large amplitude oscillations applied to target cervical vertebrae; 2 min of oscillatory pressure, repeated 3 times with 1 min rest | Treatment features for all groups: 3 weeks, 2 sessions/ week  Session duration: NR  Follow-up: None | **Pain intensity** (NPRS)  **Neck disability** (NDI) | Pain intensity: No statistical difference between Mulligan group (MD = -4.5 + 1.7) and Maitland group (MD = -3.73 + 2.15) (p = 0.884)  Neck disability: No statistical difference between Mulligan group (MD = -22.19 + 12.96) and Maitland group (MD = -15.81 + 13.99) (p = 0.589) | In patients with nonspecific neck pain, both Maitland and Mulligan mobilization techniques positively impact neck pain, functional disability, and selected psychological variables, with no significant differences between them. |
| **First Author, Year:** Vijayan et al, 2022(Vijayan et al., 2022)  **Country:** India  **Design:** RCT  **Setting:** Outpatient Department of Physical Medicine and Rehabilitation at Rajah Muthiah Medical College and Hospital (RMMCH), Annamalai University  **Sample size:** 20  **Age range:** 20-40 years  **Gender M/F:** 9/11  **Diagnosis:** Acute, subacute, and chronic nonspecific neck pain  **Diagnosis description:** Primary complaint of neck pain along with restricted movement for more than a week  **Funding:** None | **Main therapy:** SNAGs + Interferential therapy + Isometric neck exercises vs. Interferential therapy + Isometric neck exercises  **Intervention (n = 10):**  SNAGs: 6 repetitions of 2 sets/session for cervical movements, three sessions/week for two weeks (15-20 min/session with rest).  Interferential therapy: Bipolar method over the para-cervical region for 10 min at 80-100 Hz, intensity per comfort, single session for 5 consecutive days in the first week.  Isometric neck exercises: Neck flexors, extensors, and rotators; 5-sec hold × 10 repetitions each, daily for 3 days/week over 2 weeks  **Comparator (n = 10):** Interferential therapy plus isometric neck exercises | Treatment features for all groups: 2 weeks, 2 sessions/ week  Session duration: NR  Follow-up: None | **Pain intensity** (NPRS)  **Cervical ROM** (goniometer) | Pain intensity: Both groups showed a significant reduction in NPRS score (p-value=0.004, p-value=0.005), with significantly greater effectiveness in the intervention treatment group.  CROM: Both groups increased cervical range of flexion (p-value=0.001, p-value=0.001), cervical extension (p-value=0.004, p-value=0.001), and rotation movements (p-value <0.05), respectively, with the intervention treatment group demonstrating significantly greater effectiveness | Short-term Mulligan SNAGs combined with conventional physiotherapy were found to be more effective in reducing pain and improving cervical active range of motion in patients with nonspecific neck pain. |
| **First Author, Year:** Usama Jamil et al, 2022(Usama et al., 2022)  **Country:** Pakistan  **Design:** RCT  **Setting:** Physiotherapy ward of Mayo Hospital Lahore  **Sample size:** 45  **Age range:** NR  **Gender M/F:** 25/20  **Diagnosis:** Mechanical neck pain (no further precision regarding the chronicity)  **Diagnosis description:** Mechanical cervical pain starts at the back of the head and moves down the neck to the lower cervical spine and shoulders. Neck discomfort, limited range of motion, and stiffness are common indications of mechanical neck pain  **Funding:** NR | **Main therapy:** NAGs vs. MET (post-isometric relaxation)  **Intervention (n = 22):**  NAGs for neck region. PT stands in a stride stance at the side, facing posteriorly, with the hip blocking the seated patient’s shoulder. The patient’s neck is slightly flexed without rotation or side bending. The PT cradles the head with the forearm (top arm) and chest, while the other hand supports the back of the head/neck. The middle phalanx of the fifth finger (top arm) is hooked under the spinous process, with the thenar eminence of the bottom hand positioned obliquely under the fifth finger to mobilize parallel to the facet plane. After taking up the slack, the PT oscillates (2-3 times/sec) from mid to end range by pushing through the top hand toward the patient’s gaze. This is done for 5-10 seconds, followed by a reassessment  **Comparator (n = 23):**  MET for upper trapezius, elevator scapulae, and sternocleidomastoid | Treatment features for all groups: 4 weeks, 4 sessions/week  Session duration: NR  Follow-up: None | **Pain intensity** (NPRS)  **Neck disability** (NDI)  **Cervical ROM** (NR) | Pain intensity: Both groups showed a reduction in pain intensity after treatment  Neck disability: Both groups showed a reduction in neck disability after the treatment  CROM: Both groups showed an increase in the CROM after the treatment | Both MET and NAGs effectively reduce neck pain, enhance range of motion, and decrease functional disability. However, Mulligan Mobilization offers additional benefits, with greater pain reduction (NPRS) and improved functional ability (NDI). Thus, Mulligan Mobilization is a highly effective therapeutic option for patients with mechanical neck pain. |
| **First Author, Year:** El-Azeim et al, 2023(Abd El-Azeim & Grase, 2023)  **Country: Egypt**  **Design:** RCT  **Setting:** Electromyography Laboratory at the College of Physiotherapy  **Sample size:** 90  **Age range:** 18-30  **Gender M/F:** 46/44  **Diagnosis:** Non-specific neck pain in the last 3 months  **Diagnosis description:** Pain at the posterior or posterior lateral aspect of the neck in the last 3 months  **Funding:** None | **Main therapy:** SNAGS + conventional therapy vs. conventional therapy  **Intervention (n = 45):**  SNAGs: limited flexion or extension and limited rotation or side bending. SNAGs were applied as three sets; each set contained 10 repetitions in every session. CT: hot pack for 15–20 min at the back of the neck plus isometric strengthening exercises for all cervical muscles for 30 sec and repeated three times in every session plus an active range of motion exercise for neck and shoulder, chin in, and scapular retraction exercise  **Comparator (n = 45):**  CT: hot pack for 15–20 min at the back of the neck plus isometric strengthening exercises for all cervical muscles for 30 sec and repeat 3 times in every session plus an active range of motion exercise for neck and shoulder, chin in, and scapular retraction exercise | Treatment features for all groups: 4 weeks, 3 sessions/week  Session duration: NR  Follow-up: None | **Pain intensity** (VAS)  **Neck disability** (NDI) | Pain intensity: There were no significant statistical differences between groups (p=0.81)  Neck disability: There were no significant statistical differences between groups (p=0.76) | The outcome results of this trial reported improvement in muscle activity, pain, and function in both groups with more benefits in the group that received Mulligan SNAGs. |
| **First Author, Year:** Morsi et al, 2023(Morsi et al., 2023)  **Country:** Egypt  **Design:** RCT  **Setting:** Clinic of physiotherapy at Misr university for science and technology  **Sample size:** 54  **Age range:** 20-45  **Gender M/F:** 22/32  **Diagnosis:** Chronic nonspecific neck pain  **Diagnosis description:** Patients experiencing continuous neck pain that has no clear organic or pathologic origin  **Funding:** None | **Main therapy:** SNAGs vs. MFR vs. SNAGs + MFR  **Intervention (n = 18):**  SNAGs: the therapist applied an anterosuperior accessory glide across the superior spinous process or facet joint of the affected movement segment using the medial edge of one thumb's distal phalanx in conjunction with the pad of the other thumb at a 45-degree angle. The therapist's other fingers were placed on the side of the neck in an elevating position. The therapist elevated the spinous process in a direction consistent with the plane of the treated facet joints (toward the ears). The patient then actively performed the painful movement while the therapist guided the vertebra throughout the movement and resisted it when returning to neutral. This was done in 3 sets of 10 repetitions, with the force delivered perpendicular to the joint plane  **Comparator1 (n = 19):**  MRF: a description of the therapy was not reported  **Comparator2 (n = 17):** Combination of intervention + comparator 1 | Treatment features for all groups: 4 weeks, 3 sessions/week  Session duration: NR  Follow-up: None | **Pain intensity** (NPRS)  **Neck disability** (NDI)  **Cervical ROM** (goniometer) | Pain intensity: There was no clear disparity in VAS, flexion, and extension between groups post-treatment (p > 0.05).  Neck disability: There was a decrease in NDI in SNAGs+ MFR (p < 0.05) compared to both groups. There was no clear variance in NDI between group SNAGs vs MFR (p > 0.05).  CROM: There was an increase in the right and left bending ROM (p < 0.01) compared with group intervention and MFR (p < 0.05); increase in the right and left bending ROM of group SNAGs + MFR compared with the other two groups (p < 0.01); increase in right and left rotation ROM of group SNAGs + MFR compared with MFR (p < 0.05).  There was no clear disparity right and left rotation between group SNAGs and SNAGs + MFR (p > 0.05).  No marked disparity has been noticed in right and left bending and right and left rotation ROM between group SNAGs and MFR (p>0.05). | For individuals with CNSNP, the combined effect of SNAGs and MRF showed improvement over almost all the variables, which  are as follows in pain sensitivity and functional  ability, and finally, the cervical rotation and bending ROMs, suggesting applying the  combined intervention in treatment programs of  cases of CNSNP as a desirable choice. |
| **First Author, Year:** Shelke et al, 2023(Shelke et al., 2023)  **Country:** India  **Design:** RCT  **Setting:** Not reported  **Sample size:** 26  **Age range:** 18-45  **Gender M/F:** 10/16  **Diagnosis:** Acute/subacute nonspecific neck pain  **Diagnosis description:** Patients with a complaint of non-specific mechanical NP as per the defined criteria, referred to the physiotherapy clinic with the duration  of the present episode of neck pain less than three weeks  **Funding:** None | **Main therapy:** SNAGs vs. CCFT  **Intervention (n = 13):**  The therapist applied a sustained passive accessory movement along the zygapophyseal joint  plane (45º) with the participant simultaneously performing the physiological movement (comparable sign). The therapist sustained the glide throughout the movement and released it upon the return of the patient to the starting position. The dosage was decided pragmatically depending upon the symptom of the patient with 3 sets of  6–10 repetitions with a 1-min break in between each set.  **Comparator (n = 13):**  participants lay in a crook lying position with the neck in neutral. An air-filled pressure sensor was inflated to 20 mmHg and placed under the occiput. After relaxing, participants performed gentle nodding head movements. A low-load CCF exercise was executed at pressure levels determined by the CCFT, aiming for the highest comfortable pressure for 10 seconds across 10 repetitions. Each session included three sets of 10 repetitions (10-second holds) with 10-second rest pauses, focusing on correct movement without engaging superficial muscles | Treatment features for all groups: 1 day, 1 session  Session duration: NR  Follow-up: None | **Pain intensity** (NPRS)  **Cervical ROM** (inclinometer) | Pain intensity: A significant interaction between group and time was observed for the exercise group and the Mulligan mobilization group for pain intensity immediately after the intervention with no statistically significant difference between groups.  CROM: CROM improved in all directions for both groups post-intervention except bilateral rotation ranges for the Mulligan mobilization group. There was no statistically significant difference for ROM between groups. | Patients with MNP who received either active CCF exercises or Mulligan mobilization experienced similar reductions in pain intensity, increased CROM, and improved performance in the CCF test after intervention. |
| **First Author, Year:** Sun et al, 2024(Sun et al., 2024)  **Country:** China  **Design:** RCT  **Setting:** Sports Rehabilitation Laboratory of the Capital University of Physical Education  **Sample size:** 30  **Age range:** NR  **Gender M/F:** NR  **Diagnosis:** Chronic nonspecific neck pain  **Diagnosis description:** Among people with chronic neck pain, those with unknown etiology and symptoms lasting more than 12 weeks are referred to as CNSNP  **Funding:** Yes (Beijing Universities high Quality Undergraduate courses; Research on Teaching reform of "Sports Rehabilitation Therapy Technology" based on virtual simulation Rehabilitation Technology; 2023 Connotation Development-Scientific research-Science and Technology strong  school support Program-Study on the intervention effect and brain mechanism of motor cognition dual task  training on functional ankle instability) | **Main therapy:** Self-SNAGs + exercise/CCFT vs. Cervicothoracic mobilization + exercise/CCFT vs. exercise/CCFT  **Intervention (n = 10):**  Self-mobilization: (1) cervical spine rotation, (2) cervical extension. The exercise program for the deep neck flexor: strength and endurance of deep neck flexor with pressure biofeedback from 20 mmHg for 20 sec, rest for 10 sec, and increase 2 mm Hg each time until 30 mmHg; the program was designed as 3 sets/ week for the first 3 weeks and 5 sets/week for the last three weeks.  Exercise program for scapular stability: the subject lay in the prone position with the head  outside the bed and the arms extended to form the W, Y, and T shapes. In the first three weeks, two sets of each exercise, 15 repetitions  per set, 3 times a week. In the following three weeks, do each exercise 30 times per set.  **Comparator1 (n = 10):** Thoracic spine mobilization: (1) stretching of the thoracic spine using a foam roller (maintained for 5–10 sec, and the procedure was repeated for the next segment of the thoracic spine); (2) lateral rotation of the thoracic spine; and (3) prayer stretching with the foam roller. Deep neck flexor exercise under pressure biofeedback and  scapular stabilization.  **Comparator2 (n = 10):**  Deep neck flexor exercise under pressure biofeedback and  scapular stabilization | Treatment features for all groups: 6 weeks, 3 sessions/week  Session duration: 40 minutes  Follow-up: None | **Pain intensity** (VAS)  **Neck disability** (NDI)  **Cervical ROM** (goniometer) | Pain intensity, neck disability, cervical ROM: All three training programs increased cervical ROM, reduced pain, and improved neck function (P < 0.05). The exercise combined with self-mobilization in 2 groups compared with the exercise training group had better improvement in ROM of extension, lateral flexion, rotation, and quality of life (P < 0.05). Compared with exercise alone and exercise combined with cervical self-mobilization training, the exercise combined with cervicothoracic self-mobilization training was the best in improving ROM of right lateral flexion (exercise training group vs ECCTM: P < 0.01, d = 1.61, ECCM vs ECCTM: P < 0.05,  d = 1.14) and pain (exercise training group vs ECCTM: P < 0.05, d = 1.34, ECCM vs ECCTM: P < 0.05, d = 1.23). | Training targeting deep flexor muscles and shoulder stability enhances neck endurance, strength, and coordination. Self-mobilization improves cervical lateral flexion and rotation, reduces neck-related disability, and enhances quality of life. Combining exercises with cervicothoracic self-mobilization is effective in managing neck pain. |
| **First Author, Year:** Ozlu et al, 2024(Ozlu & Sahin, 2024)  **Country:** **Turkey**  **Design:** RCT  **Setting:** University  **Sample size:** 40  **Age range:** 25-66  **Gender M/F:** 10/16  **Diagnosis:** Mechanical neck pain (no further precision)  **Diagnosis description:** MNP is a non-radicular origin of local musculoskeletal system pain. It is characterized by spasms in cervical muscles caused by postural disorder and trauma  **Funding:** None | **Main therapy:** Mulligan + CT vs CT  **Intervention (n = 20):**  SNAGs by giving 15–20 s rest between 3 sets of 4–5 repetitions and CT  **Comparator (n = 20):**  CT: US (8 min, 1.5 w/cm2, pulsed), hot pack, TENS  (20 min, 60–120 Hz frequency), Interferential Current (20 min, 100 rpm) and exercises (postural and stretching, 3 sets of 10 repetitions) | Treatment features for all groups: 2 weeks, 5 sessions/week  Session duration: NR  Follow-up: None | **Pain intensity** (VAS)  **Neck disability** (NPDS)  **Cervical ROM** (goniometer) | Pain intensity, disability, CROM: Both groups were found to have statistically significant differences in VAS, CROM of the neck, and NPDS in pre- and post-treatment (p *<* 0.05). There were significant differences between groups and the intervention group was found to be better than the conventional physiotherapy group (p *<* 0.05) (VAS resting and activity scores, ROM of the neck, NPDS scores). |  |
| **First Author, Year:** Aggarwal et al, 2018(Aggarwal & Verma, 2018)  **Country:** India  **Design:** RCT  **Setting:**  Physiotherapy outpatient department  **Sample size:** 38  **Age range:** NR  **Gender M/F:** 20/18  **Diagnosis:** Work‑related neck pain (WRNP)  **Diagnosis description:** NR  **Funding:** None | **Main therapy:** Self‑SNAG mobilization + CT vs CT  **Intervention (n = 19): S**elf‑SNAGS for cervical extension and rotation of cervical (5 sessions/week/2 weeks). The selvage on one side of the towel is hooked under the spinous process. The ends of the towel on the same selvage side were firmly held, and pull was exerted toward the treatment plane in the direction of movement. Before performing self‑SNAGs, the hot pack was placed for 15 min duration around the cervical region. Ergonomic advice was incorporated into their treatment regime  **Comparator (n = 19):** Cervical isometric exercises for neck flexors, extensors, and rotators (5 sessions/week/2 weeks). Before the commencement of exercise, the patients were instructed to apply a hot pack for 15 min around the cervical region. Ergonomic advice was incorporated into their treatment regime | Treatment features for all groups: 5 sessions/week  Session duration: NR  Follow-up: 4 weeks after randomization (2 weeks after the end of the treatment) | **Pain intensity** (VAS)  **Cervical ROM** (goniometer)  **Neck disability** (NDI) | Pain intensity: Both groups improved and presented a statistically significant difference after treatment. A significant difference between groups at follow-up (4 weeks) favoring Mulligan's techniques, but not post-treatment (2 weeks)  Cervical ROM:  Extension: Significant differences within-group for Mulligan’s; no statistical differences between groups  Left lateral flexion: No statistical differences within groups, no statistical differences between groups  Right lateral flexion: No statistical differences within groups, no statistical differences between groups  Left rotation: Significant differences within the group for Mulligan’s; no statistical differences between groups  Right rotation: Significant differences within the group for Mulligan’s; no statistical differences between groups at post-treatment (2 weeks) but statistical differences between at follow-up (4 weeks), favoring Mulligan  Neck disability: Both groups improved and presented a statistically significant difference after treatment. Significant difference at post-treatment (2 weeks) favoring Mulligan's techniques; results to follow-up were not reported | Two‑week self‑SNAGs have the sufficient potential to decrease neck pain and disability and increase mobility among NP with WRNP than the conventional physiotherapy. |
| **First Author, Year:** El-Sodany et al, 2014(El-Sodany et al., 2014)  **Country:** Egypt  **Design:** RCT  **Setting:**  NR  **Sample size:** 49  **Age range:** NR  **Gender M/F:** NR  **Diagnosis:** Subacute/chronic nonspecific neck pain  **Diagnosis description:** Neck pain lasting more than 1 month  **Funding:** NR | **Main therapy:** SNAGs + exercise vs manipulation + exercise program vs exercise  **Intervention (n = 18):**  The therapist applied an anterosuperior accessory glide using reinforced thumbs on the superior spinous process or articular pillar of the involved vertebra. The thumb was angled at 45 degrees, and the glide followed the treatment plane toward the eyeball. At the end range, the movement was held for several seconds, with the patient applying overpressure. The procedure was repeated in sets of 5-10. Unilateral SNAGs were used for one-sided lesions and bilateral SNAGs for bilateral lesions  **Comparator1 (n = 15):** Manipulation: high-velocity, low-amplitude spinal manipulative thrust  **Comparator2 (n = 16):** Exercises: isometric exercises, stretching exercises, and postural exercises | Treatment features for all groups: 2 sessions/week  Session duration: NR  Follow-up: 1 month | **Pain intensity** (VAS)  **Cervical ROM** (CROM device)  **Neck disability** (NDI) | Pain intensity: Analysis of VAS showed a significant improvement in the VAS in all groups and this effect was kept during follow-up. Mann–Whitney U-test revealed that the greatest improvement in VAS occurred in the SNAGs and manipulation groups, whereas the least improvement was observed in the exercise group  Cervical ROM: ANOVA with repeated measures showed a significant increase in all ROM movements in all groups, and the effect of treatment was maintained during the follow-up as shown by the post-hoc test. The post hoc test showed no significant difference between the SNAGs group and the manipulation group, whereas there was a significant difference between the exercise group and both the SNAGs and manipulation groups in all ROM movements  Neck disability: NDI showed a significant improvement in the NDI in all groups and this effect was kept during follow-up. Mann–Whitney U-test revealed that the greatest improvement in NDI occurred in the SNAGs and manipulation groups, whereas the least improvement was observed in the exercise group | Both SNAGs mobilization and manipulation were effective in the treatment of cervical spine disorders, where no one was superior to the other as they yielded approximately the same results. In addition, the combination of mobilization or manipulation with exercise therapy produced a greater increase in CROM and a greater reduction of pain, which in turn caused an improvement of function in patients with chronic cervical disorders both after treatment and at short-term follow-up. |
| **First Author, Year:** Waqas et al, 2017(Waqas et al., 2017)  **Country:** Pakistan  **Design:** RCT  **Setting:** Physiotherapy Department National Hospital  **Sample size:** 50  **Age range:** NR  **Gender M/F:** 31/19  **Diagnosis:** Mechanical neck pain  **Diagnosis description:** MNP from both genders age 50  **Funding:** None | **Main therapy:** SNAGs + exercise + SWD vs NAG + exercise + short wave diathermy  **Intervention (n = 25):**  SNAGS associated with neck strengthening exercises (10 repetitions twice in a day) and SWD in continuous mode  **Comparator (n = 25):**  NAGs associated with neck strengthening exercises (10 repetitions twice a day) and SWD in continuous mode | Treatment features for all groups: 3 sessions/week  Session duration: Not reported  Follow-up: None | **Pain intensity** (NPRS) | Pain intensity: Statistically significant difference between the mean values of NPRS pre and post-treatment (p<0.001) in terms of pain reduction, favoring the group that received SNAGs plus exercise and SWD | SNAGS is a more effective treatment approach than NAGS in subjects with mechanical neck pain. |
| **First Author, Year:** Patel et al, 2016(Keyur et al., 2016)  **Country:** India  **Design:** RCT  **Setting:** NR  **Sample size:** 63  **Age range:** 30-50  **Gender M/F:** NR  **Diagnosis:** Chronic nonspecific neck pain  **Diagnosis description:** Nonspecific chronic neck pain (>3 months)  **Funding:** NR | **Main therapy:** Maitland + exercises vs SNAGs + exercises vs exercises  **Intervention (n = 21):** Maitland: the physiotherapist palpates the neck to find the 3 most dysfunctional joints and then performs passive joint mobilization on those joints. During passive joint mobilization, the therapist uses their thumbs to rhythmically apply pressure to a vertebra, typically in a posterior-to-anterior direction. The procedure was performed three times for 30 sec on dysfunctional joints. After mobilization, the subject had to perform the same exercises as the conventional group  **Comparator1 (n = 21):**  SNAG: in a seated position, each participant was instructed to move their head in the direction that elicited their symptoms. As the participant moved their head, the physiotherapist gently glided the painful vertebra anteriorly and sustained the glide throughout the movement. During this glide application, the participant was required to remain symptom-free and instructed to stop if any pain occurred. This movement was repeated 10 times. After mobilization, the subject performed the same exercises as the conventional group  **Comparator2 (n = 21):** Exercises: isometrics of the neck (2 sets x 10 reps); Scapula stabilizer exercises (2 sets X 10 reps); Deep neck flexor strengthening (2 sets X 10 reps); Active neck movement in all directions (10 reps) | Treatment features for all groups: 5 sessions/week  Session duration: NR  Follow-up: None | **Pain intensity** (NPRS)  **Neck disability** (Copenhagen Neck Functional Disability Scale) | Pain intensity: All three groups showed more than 3 points of improvement which suggests meaningful change. No significant difference between Maitland + exercises vs SNAGs + exercises. Scores favoring the Maitland group  Neck disability: Significant differences between Maitland and SNAGs, favoring the Maitland group (p<0.01, mean difference 2.57) | Subjects were treated with SNAGs mobilization, and conventional treatment in three groups, respectively. All three exercises are statistically significant in reducing the subject symptoms. But Maitland mobilization is significant in reducing the subject symptoms when it is compared with conventional therapy and SNAGs mobilization. |
| **First Author, Year:** Pal et al, 2019(Pal & Misra, 2019)  **Country:** India  **Design:** RCT  **Setting:** Out-patient Department of Physiotherapy, Sri Aurobindo Institute of Medical Sciences, Indore Department of Physiotherapy, IIMS Indore & Department of Physiotherapy, BHRC Indore  **Sample size:** 100  **Age range:** 20-50  **Gender M/F:** NR  **Diagnosis:** Chronic nonspecific neck pain  **Diagnosis description:** Mechanical neck  pain lasting more than 3 months.  **Funding:** NR | **Main therapy:** SNAG + CT vs CT  **Intervention (n = 50):**  SNAGs Mobilization: 5 to 10 sets of 3-5 repetitions plus CT  **Comparator (n = 50):**  CT: moist heat pack for 10 minutes, followed by cervical and upper quadrant muscle stretching (2 to 4 repetitions with a 15-30 second hold). Dynamic isometric exercises using a resistance band (2 to 3 sets of 10-15 repetitions) and postural advice | Treatment features for all groups: 6 weeks  Session duration: NR  Follow-up: None | **Pain status** (Not described)  **Active range of motion (AROM)** (no device described)  **Neck disability index** (NDI) | Pain status: Overall, the pain status was found to be different after administration of exercises in groups  AROM: At post-intervention, the mean difference in flexion and extension angles among computer professionals between groups SNAGs and CT were statistically highly significant. (p<0.001), favoring the Mulligan group  Neck disability: Both groups improved. However, a better Disability index was experienced by computer professionals of group SNAGs + CT than by computer professionals of group CT. | The group treated with the SNAG approach had significant improvement in ROM of cervical joint, pain, and disability due to mechanical neck pain and mobility deficit than those treated with conventional physiotherapy alone. |
| **First Author, Year:** Said et al, 2017(Said et al., 2017)  **Country:** Egypt  **Design:** RCT  **Setting:** Faculty of Physical Therapy, Cairo University  **Sample size:** 87  **Age range:** 20-35  **Gender M/F:** NR  **Diagnosis:** Chronic mechanical neck pain  **Diagnosis description:** NR  **Funding:** Self-funded | **Main therapy:** Self-SNAGs + CT vs SNAGs + CT vs CT  **Intervention (n = 29):**  Self-SNAGs plus CT: the towel was positioned on the posterior arch of C5 and drawn horizontally forward across the face. The purpose of the towel is for cervical flexion at C5-C6. The subject applied pressure on the towel and flexed his neck forward, sustaining end range for 3 sec. The treating patient assisted with the positioning of the towel and applied end-range overpressure in bending. Subjects were then asked each session to perform 3 sets of 10 repetitions  **Comparator1 (n = 29):**  SNAGs plus CT: the patient is seated with back support while the therapist applies an anterosuperior glide to the affected spinous process using the thumb at a 45° angle, stabilizing the neck simultaneously. The patient performs active flexion as the spinous process moves upward. This is repeated for three sets of 10 repetitions per session  **Comparator2 (n = 29):**  CT**:** a tungsten filament lamp with inert gas is placed above the patient, who lies prone with the neck uncovered. The distance is adjusted for comfort, and the treatment lasts 15 min. For TENS, electrodes are placed, and current intensity is adjusted to patient tolerance (comfortable, not painful) at 4-8 Hz. Each session lasts 20 minutes, for 12 sessions on alternate days | Treatment features for all groups: 3 sessions/week  Session duration: TENS: 20 minutes  Follow-up: None | **Pain intensity** (VAS - 10cm)  **Neck disability** (NDI) | Pain intensity: The mean values for the VAS decreased significantly after treatment compared with pre-treatment in Group 1(p<0.001, CI:(2.87-3.529), Group 2 (p<0.001, CI:(2.77-3.429) and Group 3 (p<0.001, CI:(0.921-1.579)  Neck disability: The mean values for the NDI decreased significantly after treatment compared with pre-treatment in Group 1(p<0.001, CI:(40.78-50.216), Group2(p<0.001, CI:(40.58-50.01 over Group 3(p=0.16, CI:(1.366-8.06). There was no significant difference in mean values of group 3 pre and post-treatment | It can be reasoned that both Mulligan self-mobilization and Mulligan SNAGs techniques have a similar effect in JPE, VAS, and NDI favoring traditional in chronic mechanical neck pain patients. |
| **First Author, Year:** Sultan et al, 2021(Sultan et al., 2021)  **Country:** Pakistan  **Design:** RCT  **Setting:** Kulsum International Hospital  **Sample size:** 54  **Age range:** 20-45  **Gender M/F:** 41/19  **Diagnosis:** Mechanical neck pain  **Diagnosis description:** NR  **Funding:** None | **Main therapy:** SNAGs + MET + CT vs SNAGs + CT  **Intervention (n = 27):**  MET: the subject was asked to sit in a relaxed position, then the end range of cervical muscles was achieved, and the subject was asked to contract the muscle voluntarily to 20% of total strength. The contraction was held for 6-10 seconds and then released. There were 4 repetitions in each session. SNAGs were performed at the cervical spine in a sitting position with 6-10 repetitions in two sets each in one session with 10 minutes of rest between both sets plus CT  **Comparator (n = 27):**  CT: Inferential current for 15 minutes and postural education | Treatment features for all groups: 3 sessions/week  Session duration: NR  Follow-up: None | **Pain intensity** (NPRS 0-10)  **Cervical ROM** (Inclinometer | Pain intensity: Statistically significant reduction in both groups and a statistically significant difference between groups in favor of the experimental group  Cervical ROM: Statistically significant increases in both groups (for each direction), and statistically significant differences between groups in favor of the experimental group (for each direction) | SNAGs combined with Muscle Energy Techniques (MET) improve the range of motion and effectively reduce pain in individuals with mechanical neck pain compared to conventional treatments and SNAGs alone. This approach is recommended as it helps patients regain their restricted range of motion and continue their daily activities with minimal hindrance. |
| **First Author, Year:** Tanveer et al, 2017(Tanveer et al., 2017)  **Country:** Pakistan  **Design:** RCT  **Setting:** Chaudhary Muhammad Akram, Teaching and Research Hospital, Lahore and Suriya Azeem Teaching Hospital, Lahore  **Sample size:** 75  **Age range:** 20-40  **Gender M/F:** 41/34  **Diagnosis:** Non-specific neck pain  **Diagnosis description:** NR  **Funding:** NR | **Main therapy:** SNAG + CT vs Maitland + CT vs CT  **Intervention (n = 25):**  SNAGs plus CT: The experienced physiotherapist provided the sustained glide while the patients meanwhile performed active movement on command of the physiotherapist with 3 sets of 10 repetitions for 20 min plus CT  **Comparator1 (n = 25):** Maitland plus CT: posteroanterior glide at grade I or II applied where the pain occurred before the motion barrier and grades III and IV here motion barrier was encountered before pain. This oscillatory mobilization was performed at a rate of 2-3 oscillations per second and a frequency of 3-4 mobilization of the joint lasting approximately 30 sec each. The rest time between each mobilization was 1 minute plus CT  **Comparator2 (n = 25):**  CT: Stabilization exercises, stretching exercises, and posture training | Treatment features for all groups: 4 weeks  Session duration: NR  Follow-up: None | **Pain intensity** (NPRS)  **Neck disability** (NDI) | Pain intensity: The one-way ANOVA test compared mean of three groups and p-value after treatment was p = 0.000. The difference among pain intensity showed more improvement in group 1 received SNAGs (3.52) than group 2 received Maitland mobilization (2.36), and group 3 received conventional treatment (2.04)  Neck disability: The one-way ANOVA test compared the mean of three groups and the p-value after treatment was p = 0.000. The distinction among NDI scores indicated more change in group 3 got conventional treatment (12.96) than in group 1 (18.44) and group 2 (8.68) | The SNAGs mobilization is more effective in the management of non-specific neck pain and conventional treatment improved functional status of neck than Maitland mobilization. |
| **First Author, Year:** Zemadanis, 2018(Zemadanis, 2018)  **Country:** Greece  **Design:** RCT  **Setting:** Experimental Physiology Laboratory of Medical School Faculty at National and Kapodistrian University of Athens  **Sample size:** 40  **Age range:** 20-55  **Gender M/F:** 12/28  **Diagnosis:** Chronic mechanical neck pain  **Diagnosis description:** Reproducible non-specific neck pain with a primary location between the supra nuchal line and the first thoracic spinous process, lasting more than 3 months  **Funding:** NR | **Main therapy:** NAGs + SNAGs + self-SNAGs vs SHAM Mulligan  **Intervention (n = 20):**  NAGs: passive mid-to-end range oscillatory mobilizations applied anterior-cranially at 2-3 Hz, targeting restricted cervical facet joints, with three sets of three repetitions.  SNAGs: a combination of sustained zygapophyseal glide by the therapist and active, pain-free movements (rotation, flexion, etc.) by the patient, with overpressure applied at the end range. Dosage: 6 repetitions in 3 sets for each painful direction.  Self-SNAGs: Patients use a towel to guide mobilization of the affected cervical segment while performing pain-free active movements and applying overpressure at the end range. Dosage: 3 repetitions of 3 sets, with 2 practice trials  **Comparator (n = 20):**  SHAM Mulligan: the control group followed the same parameters as the experimental but without the therapist's mobilization force or direction. Participants were taught a SHAM self-SNAG, applying 3 sec of sustained pressure on the painful or restricted cervical segment without moving the head, using the same dosage as the experimental group | Treatment features for all groups: 3 sessions/week/  with one day rest between sessions  Session duration: NR  Follow-up: 4 weeks post-intervention | **Pain intensity** (NPRS)  **Neck disability** (NDI) | Pain intensity: Simple main effect analysis of Group on NPRS was significant, with F values of pain F(1, 38)=53.8, (p=0.000). Simple main effect analysis of time factor on NPRS was significant, with F values of pain F(2,76)=42.39, (p=0.000)  Neck disability: Simple main effect analysis of Group on NDI was significant, with F values of functionality F(1,38)=41.15, (p=0.000). Simple main effect analysis of time factor on NDI was significant, with F values of functionality F(2,76)=35.59, (p=0.000) | The current study confirmed the research hypothesis that significant clinical improvements in pain and functionality scores occur after the application of the Mulligan Concept therapy protocol in patients with chronic mechanical neck pain, both in the short and mid-term timeframes. |
| **First Author, Year:** Gautam et al, 2014(Gautam et al., 2014)  **Country:** India  **Design:** RCT  **Setting:** NR  **Sample size:** 30  **Age range:** 20-45  **Gender M/F:** NR  **Diagnosis:** Mechanical neck pain  **Diagnosis description:** Patient with a primary complaint of nonspecific neck pain. Pain and Stiffness for ≥2 weeks  **Funding:** NR | **Main therapy:** SNAGs+ NAGs + CT vs Maitland + CT vs CT  **Intervention (n = 10):**  CT plus mulligan mobilization (NAGS, SNAGS). NAGS were given with 2-3 hertz (for less than 6 repetitions) and SNAGS for 6 repetitions in 3 sets.  **Comparator1 (n = 10):**  CT plus Maitland grade 2 oscillatory movements for 60 sec with 2-3 hertz.  **Comparator2 (n = 10):**  Active exercises: 10 pain-free repetitions in all directions. Isometrics: maximum contraction held for 5-10 sec for flexors, extensors, side flexors, and rotators. Moist hot packs: applied for 15 min in a sitting position on the cervical region | Treatment features for all groups: 4 sessions/week  Session duration: NR  Follow-up: None | **Pain intensity** (NPRS)  **ROM** (Goniometer)  **Neck disability index** (NDI) | Pain intensity:  Control group: NPRS between 0 to 30 (p=.013)  Maitland group: NPRS between 0 to 15 (p=.001)  Mulligan group: NPRS between 0 to 15 (p=.004), NPRS between 0 to 30 (p=.013), NPRS between 15 to 30 days (p=.011)  ROM:  Control group: flexion between 0 to 15 days (p=.03) Extension between 0 to 30 days (p=.019), Extension between 15 to 30(p=.037), left rotation between 0 to15 (p=.037)  Maitland group: Flexion between 0 to 30 days (p=.000), flexion between 15 to 30 days (p=.008), rt. rotation between 0 to 30 days (p=.010), rt. rotation between 15 to 30 days (p=.010), lt. rotation between 0 to 30 days (p=.024), lt. side flexion between 0 to 15 (p=.024), lt. side flexion between 15 to 30 (p=.003)  Mulligan group: Flexion between 0 to 30 (p=.000), flexion between 15 to 30 (p=.000), extension between 0 to 30(p=.000), extension between 15 to 30 (p=.002), rt. Rotation between 15 to 30 (p=.003), rt. Rotation between 0 to 30 (p=.000), lt. Rotation between 0 to 30 (p=.001), rt side flexion between 0 to 15 (p=.008), rt. Side flexion between 0 to 30 (p=.003), lt. Side flexion between 0 to 15 (p=.000), lt. Side flexion between 0 to 30 (p=.003)  Neck disability:  Maitland group: NDI between 0 to 15 (p=.003), NDI between 15 to 30 (p=.007)  Mulligan group: NDI between 0 to 15 (p.000), NDI between 0 to 30 (p=.000), NDI between 15 to 30 (p=.012) | Mulligan mobilization is better than Maitland mobilization in improving Pain, ROM and disability |
| **First Author, Year:** Hussain et al, 2016(Hussain et al., 2016)  **Country:** Pakistan  **Design:** RCT  **Setting:** Physiotherapy Department Shalamar Hospital, Lahore  **Sample size:** 50  **Age range:** 18-45  **Gender M/F:** NR  **Diagnosis:** Acute/subacute nonspecific neck pain  **Diagnosis description:** Patients have a chief illness of NSNP. The neck pain occurrence should be less than three months span followed by a minimum of thirty days that were pain-free  **Funding:** None | **Main therapy:** NAGs + CT vs Maitland + CT  **Intervention (n = 25):**  NAGS group: 2 – 3 hertz for less than 6 repetitions in 3 sets plus CT (Ultrasonic Therapy (ITO US – 100) in Continuous mode, frequency 1MHz, intensity 1.0 W/cm2, with 70% for 5 min and short-wave diathermy (ENRAF NONIUS Curaplus 970) in co-planer, continuous mode, frequency 27.12 MHz, wavelength 11 meters, for 15 min)  **Comparator (n = 25):**  Grade 1 and Grade II Maitland mobilization: traditional therapy plus oscillatory movements of Maitland grade I, II that were given with 2 – 3 hertz for a total of 60 sec plus CT (US and short-wave diathermy) | Treatment features for all groups: 4 sessions/week  Session duration: NR  Follow-up:  After 2nd and 4th week | **Pain intensity** (NPRS)  **Neck disability** (NDI) | Pain intensity: Group NAGS showed a mean pain score on NPRS before treatment of 4.80 (SD = 2.43242), while the group Maitland showed a mean score on NPRS before treatment of 4.64 (SD = 2.30723). After four weeks of treatment group A showed a mean score on NPRS of 0.76 (SD = 1.09087)  Neck disability: Group NAGS showed a mean pain score on NDI before treatment of 17.32(SD = 7.88310), while the group Maitland showed a mean score on NDI before treatment of 18.00 (SD = 8.38650). After four weeks of treatment group A showed a mean score of neck pain disability index of 3.92 (SD = 3.34066) | Mulligan natural apophyseal glide mobilization technique for the cure of nonspecific neck pain has been confirmed to be more helpful in aiding pain and fixing neck impairment in patients having nonspecific neck pain than Grade I & II Maitland mobilization |
| **First Author, Year:** Mohamed et al, 2020(Mohamed & Elrazik, 2020)  **Country:** Egypt  **Design:** RCT  **Setting:** Hospital of October 6 University  **Sample size:** 120  **Age range:** 20-40  **Gender M/F:** 50/70  **Diagnosis:** Chronic nonspecific neck pain  **Diagnosis description:** Chronic mechanical neck dysfunction (Neck pain lasting more than 3 months)  **Funding: NR** | **Main therapy: SNAGs + CT vs Positional Release + CT vs CT**  **Intervention (n = 40):**  CT plus SNAGs: the patient sits on a low-back chair with the cervical spine vertical, while the therapist, standing behind, observes the patient's reactions using a mirror.  A posteroanterior glide is gently applied to the C3-7 spinous process or articular pillar using the therapist’s thumbs. The patient actively moves the neck toward the symptomatic direction, applying overpressure at the end of the restricted range (flexion, extension, rotation, side bending). This is repeated six times in three sets  **Comparator1 (n = 40):**  CT plus Positional Release: the patient, in a supine position, had the upper trapezius trigger point identified. The therapist applied thumb pressure while laterally flexing the head toward the affected side and abducting the shoulder to 90 degrees for fine-tuning. The pressure was maintained for 90 sec to release tension, then the head was passively returned to neutral  **Comparator2 (n = 40):**  CT: Infrared radiation for 15 min with the patient seated, followed by strengthening exercises for neck flexors, extensors, and side benders (10 repetitions, 3 sets), and isometric exercises (10 repetitions, 3 sets, 6-sec holds) | Treatment features for all groups: 3 sessions/week  Session duration: NR  Follow-up: None | **Pain intensity** (NPRS)  **ROM** (goniometer)  **Neck disability index** (NDI) | Pain intensity: There was a significant decrease in NPRS of the three groups post-treatment compared with that of pretreatment (p < 0.001). There was a significant decrease in NPRS of group B compared with that of group A and C post-treatment (p < 0.001) and a significant decrease in NPRS of group C compared with that of group A post-treatment (p < 0.001)  ROM: There was a significant increase in neck ROM post treatment compared with that pretreatment in the groups A, B and C (p < 0.001). Group B showed the highest percent of improvement and was followed by group C, while group A showed the lowest percent of improvement. There was a significant increase in neck ROM of group B compared with that of group A and C post-treatment (p < 0.001) and a significant increase in neck ROM of group C compared with that of group A post-treatment (p <0.001).  Neck disability: There was a significant decrease in NDI of the three groups post-treatment compared with that of pretreatment (p < 0.001). There was a significant decrease in NDI of group B compared with that of group A and C post-treatment (p < 0.001) and a significant decrease in NDI of group C compared with that of group A post-treatment (p < 0.001) | Adding SNAGs to conventional therapy is more effective in improving cervical range of motion, decreasing pain, and reducing functional disability in patients with chronic mechanical neck dysfunction compared to conventional therapy alone or conventional therapy combined with positional release techniques. |
| **First Author, Year:** Shehri et al, 2018(Shehri et al., 2018)  **Country:** Saudi Arabia  **Design:** RCT  **Setting:** Physical Therapy Department of Prince Sultan Military Medical City, Riyadh, Saudi Arabia  **Sample size:** 50  **Age range:** 30-50  **Gender M/F:** NR  **Diagnosis:** Non-specific neck pain  **Diagnosis description:** Patient with primary complaint of non-specific neck pain. Pain and Stiffness for ≥2 weeks  **Funding:** NR | **Main therapy:** SNAGs + CT vs Maitland + CT  **Intervention (n = 25):**  SNAG plus CT: The participant, seated, moves their head in the direction that provokes symptoms while the physiotherapist applies an anterior glide to the painful vertebra, maintaining it symptom-free. The participant stops if any pain occurs. This movement is repeated 10 times plus active, isometrics exercises, moist hot packs  **Comparator (n = 25):** Maitland’s Mobilization plus conventional therapy (Active, Isometrics exercises, moist hot packs): Thumbs are positioned at the facet of the hypomobile cervical vertebra, applying unilateral posteroanterior (PA) oscillatory pressure using Grade II and III Maitland techniques at 2 Hz for 2 min, repeated three times with 1-min rest intervals plus active, isometrics exercises, moist hot packs | Treatment features for all groups: 3 sessions/week  Session duration: NR  Follow-up: None | **Pain intensity** (VAS)  **ROM** (Goniometer)  **Neck disability index** (NDI) | Pain intensity: Both groups had clinically significant differences in pre Rx to Post RX values as p values for group SNAGs and Maitland were p=0.06 and p=0.005 respectively  Cervical ROM: Both groups had significant differences in pre Rx to Post RX p=0.000  Neck disability: Both groups had significant differences in pre Rx to Post RX p=0.05 (SNAGs), p=0.005 (Maitland) | Both mobilization techniques are clinically significant in reducing the subject's symptoms. However, Maitland mobilization is statistically significant in reducing the subject's symptoms compared to Mulligan SNAGs mobilization. |

*The results from Study 1A and 1B were not merged despite the fact that the sample was the same in both studies. Due to the way of reporting, it was difficult to combine the information; although they were treated as one study.

ANOVA: Analysis of variance;

CCF: Cranio-cervical flexion;

CCFT: Cranio-cervical flexion test;

CNSNP: Chronic non-specific neck pain;

CROM: Cervical Range of Motion;

CT: Conventional treatment;

HVLA: High velocity and low amplitude;

IETP: Isometric Exercise Training Program;

IQR: inter quartile range;

JPE: Joint position error;

SNAGs: Sustained Natural Apophyseal Glide;

NAG: Natural Apophyseal Glides;

NPRS: Numeric Pain Rating Scale;

NDI: Neck Disability Index Questionnaire;

NP: nursing professionals;

NR: Not reported;

MD: Medial deviation;

MET: Muscle energy Technique;

MFR: Myofascial release;

MMT: Mulligan mobilisation techniques;

MWM: Mobilisation with movement;

RCT: Randomized controlled trial;

ROM: Range of Motion;

SWD: Short wave diathermy;

TENS: Transcutaneous electrical nerve stimulation;

US: Ultrasound therapy;

VAS: Visual Analogue scale;

WRNP: Work related neck pain

References:

Abd El-Azeim, A. S., & Grase, M. O. (2023). Efficacy of Mulligan on electromyography activation of cervical muscles in mechanical neck pain: randomized experimental trial. *Physiotherapy Quarterly*, *31*(4). <https://doi.org/https://dx.doi.org/10.5114/pq.2023.117224>

Aggarwal, S., & Verma, M. (2018). Efficacy of Mulligan’s self-sustained natural apophyseal glides in decreasing pain, disability, and improving neck mobility among the nursing professional suffering from work-related neck pain. *Arch Med Health Sci*, *6*(1), 48-53.

Alansari, S. M., Youssef, E. F., & Shanb, A. A. (2021). Efficacy of manual therapy on psychological status and pain in patients with neck pain. A randomized clinical trial. *Saudi Med J*, *42*(1), 82-90. <https://doi.org/10.15537/smj.2021.1.25589>

Ali, A., Shakil-Ur-Rehman, S., & Sibtain, F. (2014). The efficacy of Sustained Natural Apophyseal Glides with and without Isometric Exercise Training in Non-specific Neck Pain. *Pakistan journal of medical sciences*, *30*(4), 872-874.

Alshami, A. M., & AlSadiq, A. I. (2021). Outcomes of scapulothoracic mobilisation in patients with neck pain and scapular dyskinesis: A randomised clinical trial. *J Taibah Univ Med Sci*, *16*(4), 540-549. <https://doi.org/10.1016/j.jtumed.2021.03.006>

Buyukturan, O., Buyukturan, B., Sas, S., Kararti, C., & Ceylan, I. (2018). The Effect of Mulligan Mobilization Technique in Older Adults with Neck Pain: A Randomized Controlled, Double-Blind Study [Randomized Controlled Trial]. *Pain Research & Management*, *2018*, 2856375. <https://doi.org/https://dx.doi.org/10.1155/2018/2856375>

Duymaz, T., & Yagci, N. (2018). Effectiveness of the mulligan mobilization technique in mechanical neck pain. *Journal of Clinical and Analytical Medicine*, *9*(4), 304-309. <https://doi.org/http://dx.doi.org/10.4328/JCAM.5715>

El-Sodany, A. M., Alayat, M. S. M., & Zafer, A. M. I. (2014). Sustained natural apophyseal glides mobilization versus manipulation in the treatment of cervical spine disorders: a randomized controlled trial. *International Journal*, *2*(6), 274-280.

Ganesh, G. S., Mohanty, P., Pattnaik, M., & Mishra, C. (2015). Effectiveness of mobilization therapy and exercises in mechanical neck pain [Comparative Study

Randomized Controlled Trial]. *Physiotherapy Theory & Practice*, *31*(2), 99-106. <https://doi.org/https://dx.doi.org/10.3109/09593985.2014.963904>

Gautam, R., Dhamija, J. K., Puri, A., Trivedi, P., Sathiyavani, D., & Nambi, G. (2014). Comparison of Maitland and Mulligan mobilization in improving neck pain, ROM and disability. *Int J Physiother Res*, *2*(3), 482-487.

Hussain, S. I., Ahmad, A., Amjad, F., Shafi, T., & Shahid, H. A. (2016). Effectiveness of natural apophyseal glides versus grade I and II Maitland mobilization in Non-specific neck pain. *Annals of King Edward Medical University Lahore Pakistan*, *22*, 23-29.

Izquierdo Perez, H., Alonso Perez, J. L., Gil Martinez, A., La Touche, R., Lerma-Lara, S., Commeaux Gonzalez, N., Arribas Perez, H., Bishop, M. D., & Fernandez-Carnero, J. (2014). Is one better than another?: A randomized clinical trial of manual therapy for patients with chronic neck pain. *Man Ther*, *19*(3), 215-221. <https://doi.org/10.1016/j.math.2013.12.002>

Keyur, M. P., Balaganapathy, M., & Hinal, M. P. (2016). Effect of Maitland mobilization versus mulligan (Snags) mobilization on head repositioning accuracy (Hra), pain and functional disability in patients with chronic neck pain‑A randomized controlled clinical trial. *Int J Curr Res*, *8*, 31144‑31149.

Kumar, D., Sandhu, J. S., & Broota, A. (2011). Efficacy of mulligan concept (NAGs) on pain at available end range in cervical spine: A randomised controlled trial. *Indian J. Physiotherap. Occup. Ther. Indian Journal of Physiotherapy and Occupational Therapy*, *5*(1), 154-158.

Lopez-Lopez, A., Alonso Perez, J. L., Gonzalez Gutierez, J. L., La Touche, R., Lerma Lara, S., Izquierdo, H., & Fernandez-Carnero, J. (2015). Mobilization versus manipulations versus sustain apophyseal natural glide techniques and interaction with psychological factors for patients with chronic neck pain: randomized controlled trial. *European journal of physical & rehabilitation medicine.*, *51*(2), 121-132.

Manzoor, A., Anwar, N., Khalid, K., Haider, R., Saghir, M., & Javed, M. A. (2021). Comparison of effectiveness of muscle energy technique with Mulligan mobilization in patients with non-specific neck pain. *J Pak Med Assoc*, *71*(6), 1532-1524. <https://doi.org/10.47391/JPMA.981>

Mohamed, E. E., & Elrazik, R. K. A. (2020). Sustained natural apophyseal glides versus positional release therapy in the treatment of chronic mechanical neck dysfunction [Article]. *International Journal of Human Movement and Sports Sciences*, *8*(6), 384-394. <https://doi.org/10.13189/saj.2020.080610>

Morsi, A. A., Al-Kabalawy, M. A., Aneis, Y. M., Hamza, M. S., & Atta, H. K. (2023). Effect of Sustained Natural Apophyseal Glides and Myofascial Release on Chronic Nonspecific Neck Pain: Randomized Controlled Trial. *Journal of Population Therapeutics and Clinical Pharmacology*, *30(8)*, e390-e404. <https://doi.org/https://dx.doi.org/10.47750/jptcp.2023.30.08.042>

Ozlu, O., & Sahin, M. (2024). The effect of mulligan mobilization technique application in addition to conventional physiotherapy on pain and joint range of motion in people with neck pain. *Journal of Bodywork and Movement Therapies*, *39*, 225-230. <https://doi.org/https://dx.doi.org/10.1016/j.jbmt.2024.02.009>

Pal, A., & Misra, A. (2019). EFFECTIVENESS OF SNAG MOBILIZATION ON COMPUTER PROFESSIONALS WITH MECHANICAL NECK PAIN AND MOBILITY DEFICIT. *International Journal of Physiotherapy and Research*, *7*(2), 3022-3027. <https://doi.org/https://dx.doi.org/10.16965/ijpr.2019.104>

Rezkallah, S. S., & Abdullah, G. A. (2018). Comparison between sustained natural apophyseal glides (SNAG’s) and myofascial release techniques combined with exercises in non specific neck pain. *Physiotherapy Practice & Research*, *39*(2), 135-145. <https://doi.org/10.3233/PPR-180116>

Said, M. S., Ali, O. I., Elazm, S. N. A., & Abdelraoof, N. A. (2017). Mulligan self mobilization versus Mulligan snags on cervical position sense. *International Journal of Physiotherapy*, *4*(2), 93-100.

Shamsi, S., Alyazedi, F., Abdelkader, S., Khan, S., & Akhtar, A. (2021). Efficacy of sustained natural apophyseal glides in the management of mechanical neck pain: A randomized clinical trial. *Indian Journal of Medical Specialities*, *12*(4). <https://doi.org/10.4103/injms.injms_30_21>

Shehri, A. A., Khan, S., Shami, S., & Almureef, S. S. (2018). COMPARATIVE STUDY OF MULLIGAN (SNAGS) AND MAITLAND MOBILIZATION IN NECK PAIN. *European Journal of Physical Education and Sport Science*, *5*(1), 19-29. <https://doi.org/doi>: 10.5281/zenodo.1481977

Shelke, A., Prabhu, B. A., Balthillaya, M. G., Kumaran, S. D., & Raja, G. P. (2023). Immediate effect of craniocervical flexion exercise and Mulligan mobilisation in patients with mechanical neck pain - A randomised clinical trial. *Hong Kong Physiotherapy Journal*, *43(2)*, 137-147. <https://doi.org/https://dx.doi.org/10.1142/S1013702523500154>

Sultan, N., Khushnood, K., Altaf, S., Awan, M. M. A., Qureshi, S., & Mehmood, R. (2021). Muscle Energy Technique Augmented with Sustained Natural Apophyseal Glides; An Effective Way to Improve Mechanical Neck Pain and Range of Motion: A Randomized Control Trial [Article]. *Journal of Islamic International Medical College*, *16*(2), 96-100. <https://www.scopus.com/inward/record.uri?eid=2-s2.0-85150527555&partnerID=40&md5=e40920e5fc54e272b271b3389a9bb3fd>

Sun, X., Chai, L., Huang, Q., Zhou, H., & Liu, H. (2024). Effects of exercise combined with cervicothoracic spine self-mobilization on chronic non-specific neck pain. *Scientific reports*, *14(1)*, 5298. <https://doi.org/https://dx.doi.org/10.1038/s41598-024-55181-8>

Tachii, R., sen, s., & Arfath, U. (2015). Short term effect of sustained apohuseal glides on cervical joint position sense, pain, and neck disability in patients with chronic neck pain *International Journal of Therapies and Rehabilitation Research*, *4*(4), 244.

Tank, K., Choksi, P., & Makwana, P. (2018). To study the effect of muscle energy technique versus Mulligan SNAGs on pain, range of motion, and functional disability for individuals with mechanical neck pain: A comparative study. *International Journal of Physiotherapy and Research*, *6*(1), 2582-2587.

Tanveer, F., Afzal, M., Adeel, S., Shahid, S., & Masood, M. (2017). Comparison of sustained natural apophyseal glides and maitland manual therapy in non-specific neck pain on numeric pain rating scale and neck disability index. *Annals of King Edward Medical University*, *23*.

Usama, J., Iram, A., Sania, M., Saddiqa, Q., Hafiz Muhammad Uzair, A., Aliza, T., Arifa, M., & Mudassar, I. (2022). Comparative Effect of Muscle Energy Techniques and Mulligan Mobilization on Pain & Range of Motion in patients with Mechanical Neck Pain. In (pp. 195-199): CrossLinks International Publishers.

Vijayan, K., Sivaraman, A., Kumaresan, P., & Palani, J. (2022). Short-term Effect of Mulligan SNAGs on Pain Intensity, Cervical Range of Motion and Craniovertebral Angle in Patients with Non Specific Neck Pain: A Quasi-experimental Study. *Journal of Clinical and Diagnostic Research*, *16(7)*, YC05-YC08. <https://doi.org/https://dx.doi.org/10.7860/JCDR/2022/55962.16547>

Waqas, S., Shah, S. H. A., Zafar, U., & Akhtar, M. F. (2017). Comparison of Mulligan Sustained Natural Apophyseal Glides Versus Mulligan Natural Apophyseal Glides in Mechanical Neck Pain. . *Annals of King Edward Medical University*, *23*. <https://doi.org/https://doi.org/10.21649/akemu.v23i3.2007>

Zemadanis, K. (2018). The short and mid-term effects of Mulligan concept in patients with chronic mechanical neck pain. *J Nov Physiother Rehabil*, *2*(2), 022-021.
