# Supplementary material for: The Effectiveness of Mulligan's Techniques in Non‐Specific Neck Pain: A Systematic Review and Meta‐Analysis
Source: Physiother Res Int. 2025 May 29;30(3):e70045. doi: 10.1002/pri.70045 (PMC12121345; doi:10.1002/pri.70045)
Supplement: Supplementary file 5 — Supporting Information S5 [file PRI-30-e70045-s003.docx]

**Appendix 5. Quality assessment of included studies (n=33) according to the compiled set of items.**

**Commentary about the table below:**

All the studies were described as randomized. but only 18 studies (54.5%) described an appropriate randomization, and 15 studies (45.5%) employed a concealed allocation. Four studies (12.1%) used a double-blinded design. and only five (15.2%) used an appropriate method of blinding. The assessor was blinded in seven studies (21.2%) and none of the studies blinded the therapist. When analyzing issues regarding intervention. All the studies except four (87.9%) described the treatment protocol appropriately for the main intervention group and for the control or comparison group. Eight studies (24.2%) used a control group, and two studies (6.1%) used a placebo group. Testing participants’ compliance and adherence to the intervention was only met by five (15.2%) studies. The report of adverse effects was another issue that was not met by many studies (only five studies (15.2%) reported adverse effects). 15 studies (45.5%) reported withdrawals and dropouts, but only seven studies (21.2%) explained the reasons. 14 studies (42.4%) used intention-to-treat analysis to prevent bias in data analysis. In all studies. the outcomes were relevant and objective. There were a limited number of studies that described the outcome’s validity (10 studies (30.3%)), reliability (13 studies (39.4%)), and responsiveness (four studies (12.1%)). Concerning statistical issues, the sample size was adequate in ten studies (30.3%). The statistical analysis was appropriated in all studies except two studies (6.1%). Only four studies (12.1%) reported an evaluation of the clinical significance of their results. Since 34 studies were included in this review, in order to make the data presentation clearer, we divided the quality of assessment results into two tables (Table A3.A and Table A3.B).

Table A5.A Quality assessment of included studies according to the compiled items.

| **Items** | **Lopez-Lopez et al. 2015; and Izquierdo-Perez et al.. 2014**  **^1.2^** | **Tachiiet al.. 2015**  **^3^** | **Buyukturan et al.. 2018**  **^4^** | **Kumar et al.. 2011^5^** | **Duymaz et al.. 2018^6^** | **Ali et al.. 2014^7^** | **Rezkallah et al.. 2018^8^** | **Tank et al.. 2018^9^** | **Ganesh et al.. 2014^10^** | **Manzoor et al.. 2021^11^** | **Shami et al.. 2021^12^** | **Alshami et al.. 2021^13^** | **Alansari et al.. 2021^14^** | **Vijayan et al. 2022^15^** | **Usama Jamil et al. 2022^16^** | **El-Azeim et al. 2023^17^** | **Morsi et al. 2023^18^** |
| --- | --- | --- | --- | --- | --- | --- | --- | --- | --- | --- | --- | --- | --- | --- | --- | --- | --- |
| Inclusion and exclusion criteria clearly defined consensus | Yes | Yes | Yes | Yes | No | No | Yes | Yes | Yes | Yes | Yes | Yes | Yes | Yes | No | Yes | Yes |
| Study described as randomized consensus | Yes | Yes | Yes | Yes | Yes | Yes | Yes | Yes | Yes | Yes | Yes | Yes | Yes | Yes | Yes | Yes | Yes |
| Method of randomization described and appropriate consensus | Yes | Unclear | Yes | Unclear | No | Unclear | Yes | Unclear | Yes | Unclear | Yes | Yes | Unclear | No | Unclear | Yes | Yes |
| Method of randomization concealed Consensus | Yes | Unclear | Unclear | Unclear | No | Unclear | Yes | Unclear | Yes | Unclear | No | No | Unclear | Unclear | Unclear | Yes | Yes |
| Baseline comparability regarding the most important prognostic indicators consensus | Yes | Unclear | Yes | Unclear | Yes | Unclear | Yes | Unclear | Unclear | Unclear | Yes | Yes | Yes | Unclear | Yes | Yes | Yes |
| Study described as double-blind consensus | Yes | No | Yes | Yes | No | No | No | Unclear | No | No | No | No | No | No | No | No | No |
| Method of blinding appropriate consensus | Yes | Unclear | Yes | Unclear | No | Unclear | Unclear | Unclear | Unclear | No | Unclear | Unclear | Unclear | NA | Unclear | Unclear | Yes |
| Blinding investigator consensus | Unclear | Unclear | Yes | Unclear | Unclear | Unclear | Unclear | Unclear | Unclear | Unclear | Unclear | No | Unclear | No | Unclear | Unclear | Unclear |
| Blinding of assessors consensus | Yes | Unclear | Yes | Unclear | Unclear | Unclear | Unclear | Unclear | Unclear | Unclear | Unclear | Unclear | No | No | Unclear | Yes | Yes |
| Blinding of participants consensus | Yes | Unclear | Yes | Unclear | Unclear | Unclear | Unclear | Unclear | Unclear | No | No | Unclear | No | No | Unclear | Unclear | Unclear |
| Blinding of therapist consensus | No | No | No | No | Unclear | No | No | No | No | No | No | No | No | No | No | Unclear | No |
| Blinding of outcomes analysis (statistician) consensus | Unclear | Unclear | Unclear | Unclear | Unclear | Unclear | Unclear | Unclear | Unclear | Unclear | Unclear | Unclear | Unclear | No | Unclear | Unclear | Unclear |
| Treatment protocol adequately described for treatment group consensus | Yes | Yes | Yes | Yes | Unclear | Unclear | Yes | Yes | Yes | Unclear | Yes | Yes | Yes | Yes | Yes | Yes | Yes |
| Treatment protocol adequately described for control or comparision group consensus | Yes | Yes | Yes | Yes | Unclear | Unclear | Yes | Yes | Yes | Unclear | Yes | Yes | Yes | Yes | Yes | Yes | No |
| Control group adequate consensus | Yes | No | Yes | Yes | No | No | No | No | Yes | NA | NA | No | No | Yes | Yes | Yes | No |
| Placebo group adequate consensus | No | NA | No | Yes | NA | NA | NA | No | NA | NA | Unclear | No | No | No | No | No | No |
| Co-interventions avoided or comparable consensus | Unclear | Unclear | Unclear | Unclear | Unclear | Unclear | Unclear | Unclear | Unclear | Unclear | No | Unclear | Unclear | Unclear | Unclear | Unclear | Unclear |
| Co-interventions reported for each group separately consensus | No | No | No | No | No | No | No | No | No | Unclear | Unclear | Unclear | Unclear | No | No | No | No |
| Testing of subject compliance to treatment protocol consensus | Yes | Unclear | Unclear | Unclear | Yes | Unclear | Unclear | Unclear | Yes | Unclear | Unclear | Unclear | Yes | Unclear | Unclear | Unclear | Unclear |
| Compliance acceptable in all group consensus | Yes | Unclear | Unclear | Unclear | Unclear | Unclear | Unclear | Unclear | Yes | Unclear | Yes | Unclear | Yes | Unclear | Unclear | Yes | Yes |
| Description of withdraws and dropouts consensus | Yes | Yes | Yes | No | No | NA | Yes | Unclear | Yes | No | Yes | Yes | Yes | NA | No | NA | NA |
| Withdrawal/dropouts rate described and acceptable consensus | Yes | Yes | Yes | Unclear | Unclear | Yes | Yes | Unclear | No | No | Yes | Yes | Yes | NA | Unclear | NA | Yes |
| Reasons for dropouts consensus | Unclear | No | No | No | No | NA | NA | No | Unclear | No | No | No | Yes | NA | No | NA | NA |
| Adverse effects described consensus | Yes | No | No | No | No | No | No | No | Yes | No | Yes | No | No | No | No | No | No |
| Short term follow measurement performed consensus | Yes | Yes | Yes | Yes | Yes | Yes | Yes | Yes | Yes | No | No | Yes | Yes | Unclear | No | No | No |
| Long term follow measurement performed consensus | No | NA | No | No | NA | NA | No | No | Yes | No | Yes | No | No | Unclear | No | No | No |
| The timing of the outcome assessment was comparable in all groups consensus | Yes | Yes | Unclear | Yes | Yes | Yes | Yes | Yes | Yes | Yes | Unclear | Yes | Yes | Yes | Yes | Yes | Yes |
| Description of outcome measures consensus | Yes | Yes | Yes | Yes | Yes | Unclear | Yes | No | Yes | Unclear | Yes | Yes | Yes | Yes | No | Yes | Yes |
| Relevant outcomes were used consensus | Yes | Yes | Yes | Yes | Yes | Yes | Yes | Yes | Yes | Yes | No | Yes | Yes | Unclear | Unclear | Yes | Yes |
| Validity reported for the main outcome measure consensus | Yes | No | No | No | No | No | Yes | No | Yes | No | No | Yes | Unclear | No | No | Yes | Yes |
| Responsiveness reported for the main outcome measure consensus | Yes | No | No | No | No | No | No | No | No | No | No | No | No | No | No | No | No |
| Reliability reported for the main outcome measure consensus | Yes | No | No | No | No | No | Yes | No | Yes | No | Yes | Yes | Unclear | No | No | Unclear | Yes |
| Use of objective outcome measures consensus | Yes | Yes | Yes | Yes | Yes | Yes | Yes | Yes | Yes | Yes | Yes | Yes | Yes | Yes | Yes | Yes | Yes |
| Descriptive measures identified and reported for the primary outcome consensus | Yes | Yes | Yes | No | Yes | No | Yes | Yes | Yes | Yes | Yes | Yes | Yes | Yes | Yes | Yes | Yes |
| Appropriate statistical analysis used consensus | Yes | Yes | Yes | Yes | Yes | Unclear | Yes | Yes | Yes | Yes | Yes | Yes | Yes | Yes | Unclear | Yes | Yes |
| Sample size calculation prior to initiation of the study consensus | Yes | No | Yes | No | No | No | Yes | No | Yes | No | Yes | Yes | Yes | Unclear | Unclear | Yes | Yes |
| Adequate sample size consensus | Yes | Unclear | Yes | Unclear | Unclear | Unclear | Yes | Unclear | No | Unclear | No | Yes | Unclear | Yes | Yes | Yes | Yes |
| Sample size described for each group consensus | Yes | No | Yes | No | Yes | Yes | Yes | Yes | Yes | Unclear | Yes | Yes | Yes | Yes | Unclear | Yes | Yes |
| Intention to treat analysis used consensus | Yes | No | Yes | Unclear | Unclear | Yes | Yes | Unclear | Unclear | Unclear | No | Yes | Unclear | No | No | Yes | No |
| Clinical significance reported consensus | Yes | No | No | No | No | No | Yes | No | Yes | No | No | No | No | No | No | Yes | No |
| **Number of items accomplished/total of applicable items** | **34** | **14** | **25** | **15** | **12** | **9** | **25** | **12** | **26** | **7** | **18** | **21** | **19** | **12** | **9** | **22** | **22** |
| **% of items accomplished** | **85** | **35** | **62.5** | **37.5** | **30** | **22.5** | **62.5** | **30** | **65** | **17.5** | **45** | **52.5** | **47.5** | **30** | **22.5** | **55** | **55** |

Table A5.B Quality assessment of included studies according to the compiled items (continuation).

| **Items** | **Shelke et al. 2023^19^** | **Sun et al. 2024^20^** | **Ozlu et al. 2024^21^** | **Aggarwal et al 2018^22^** | **El-Sodany et al 2014^23^** | **Gautam et al. 2014^24^** | **Hussain et al 2016^25^** | **Mohamed et al 2020^26^** | **Said et al 2017^27^** | **Sultan et al 2021^28^** | **Tanveer et al 2017^29^** | **Zemadani et al 2018^30^** | **Waqas et al 2017^31^** | **Keyur et al 2016^32^** | **Abduhllah Shehri et al 2018^33^** | **Pal et al 2019^34^** | **How many studies 'YES'** | **% 'yes'** |
| --- | --- | --- | --- | --- | --- | --- | --- | --- | --- | --- | --- | --- | --- | --- | --- | --- | --- | --- |
| Inclusion and exclusion criteria clearly defined consensus | Yes | Yes | Yes | Unclear | Yes | Yes | Yes | Yes | Unclear | Yes | Yes | Yes | Unclear | Yes | Yes | Yes | **27** | **81.8** |
| Study described as randomized consensus | Yes | Yes | Yes | Yes | Yes | Yes | Yes | Yes | Yes | Yes | Yes | Yes | Yes | Yes | Yes | Yes | **33** | **100.0** |
| Method of randomization described and appropriate consensus | Yes | Yes | Yes | Yes | Yes | No | Yes | Unclear | Yes | No | Yes | Yes | No | Yes | No | No | **18** | **54.5** |
| Method of randomization concealed Consensus | Yes | Yes | Yes | Yes | Unclear | No | Yes | Yes | Yes | Yes | No | Yes | No | Yes | No | No | **15** | **45.5** |
| Baseline comparability regarding the most important prognostic indicators consensus | Yes | Yes | Yes | Yes | Yes | Unclear | Yes | Yes | Yes | Yes | Yes | Yes | Yes | Unclear | Unclear | Unclear | **21** | **63.6** |
| Study described as double-blind consensus | No | No | No | No | No | No | No | No | No | No | No | Yes | No | No | No | No | **4** | **12.1** |
| Method of blinding appropriate consensus | Yes | Unclear | No | Unclear | Unclear | Unclear | Unclear | No | No | Yes | No | No | No | Unclear | No | No | **5** | **15.2** |
| Blinding investigator consensus | No | No | No | Unclear | Unclear | Unclear | Unclear | Unclear | Unclear | Unclear | Unclear | Unclear | Unclear | Unclear | Unclear | Unclear | **1** | **3.0** |
| Blinding of assessors’ consensus | Yes | No | No | No | Unclear | No | No | No | Unclear | Yes | No | Yes | No | No | No | No | **7** | **21.2** |
| Blinding of participants consensus | No | Yes | Unclear | No | No | No | No | No | No | Yes | No | Yes | No | No | No | No | **5** | **15.2** |
| Blinding of therapist consensus | No | No | No | No | No | No | No | No | No | No | No | No | No | No | No | No | **0** | **0.0** |
| Blinding of outcomes analysis (statistician) consensus | Unclear | No | Unclear | Unclear | Unclear | Unclear | Unclear | Unclear | Unclear | Unclear | Unclear | Unclear | Unclear | Unclear | Unclear | Unclear | **0** | **0.0** |
| Treatment protocol adequately described for treatment group consensus | Yes | Yes | Yes | Yes | Yes | Yes | Yes | Yes | Yes | Yes | Yes | Yes | No | Yes | Yes | Yes | **29** | **87.9** |
| Treatment protocol adequately described for control or comparison group consensus | Yes | Yes | Yes | Yes | Yes | Yes | Yes | Yes | Yes | Yes | Yes | Yes | No | Yes | Yes | Yes | **28** | **84.8** |
| Control group adequate consensus | No | Yes | No | No | No | No | No | No | No | No | No | NA | No | No | No | No | **8** | **24.2** |
| Placebo group adequate consensus | No | No | No | No | No | No | No | No | No | No | No | Yes | No | No | No | No | **2** | **6.1** |
| Co-interventions avoided or comparable consensus | Unclear | Unclear | Unclear | Yes | Unclear | Unclear | Unclear | Unclear | Unclear | No | No | Unclear | Yes | Unclear | No | No | **2** | **6.1** |
| Co-interventions reported for each group separately consensus | Unclear | Yes | Unclear | No | No | No | No | No | No | No | No | No | Yes | No | No | No | **2** | **6.1** |
| Testing of subject compliance to treatment protocol consensus | Unclear | Unclear | Unclear | No | No | No | No | No | No | No | No | Yes | No | No | No | No | **5** | **15.2** |
| Compliance acceptable in all group consensus | Yes | Yes | Yes | Unclear | Unclear | Unclear | Unclear | Unclear | Unclear | Unclear | Unclear | Yes | Unclear | Unclear | Unclear | Unclear | **10** | **30.3** |
| Description of withdraws and dropouts consensus | Yes | Yes | Yes | Yes | No | No | No | No | Yes | Yes | No | Yes | No | No | No | No | **15** | **45.5** |
| Withdrawal/dropouts rate described and acceptable consensus | Yes | Yes | Yes | Yes | Unclear | Unclear | No | Unclear | Yes | Yes | Unclear | Yes | Unclear | No | No | No | **16** | **48.5** |
| Reasons for dropouts consensus | Yes | Yes | Yes | Yes | No | No | No | No | Yes | No | No | Yes | No | No | No | No | **7** | **21.2** |
| Adverse effects described consensus | Yes | No | No | No | No | No | No | No | No | No | No | Yes | No | No | No | No | **5** | **15.2** |
| Short term follow measurement performed consensus | No | No | No | Yes | Yes | Yes | Yes | Yes | Yes | Yes | Yes | Yes | Yes | Yes | Yes | Yes | **24** | **72.7** |
| Long term follow measurement performed consensus | No | No | No | No | No | No | No | No | No | No | No | No | No | No | No | Yes | **3** | **9.1** |
| The timing of the outcome assessment was comparable in all groups consensus | Yes | Yes | Yes | Yes | Yes | Yes | Yes | Yes | Yes | Yes | Yes | Yes | Yes | Yes | Yes | Yes | **31** | **93.9** |
| Description of outcome measures consensus | Yes | Yes | Yes | No | Yes | Yes | Unclear | Yes | Yes | Yes | No | Yes | No | Yes | No | No | **23** | **69.7** |
| Relevant outcomes were used consensus | Yes | Yes | Yes | Yes | Yes | Yes | Yes | Yes | Yes | Yes | Yes | Yes | Yes | Yes | Yes | Yes | **30** | **90.9** |
| Validity reported for the main outcome measure consensus | No | No | No | No | Yes | No | No | Yes | Yes | No | No | Yes | No | No | No | No | **10** | **30.3** |
| Responsiveness reported for the main outcome measure consensus | No | No | No | No | Yes | No | No | Yes | No | No | No | Yes | No | No | No | No | **4** | **12.1** |
| Reliability reported for the main outcome measure consensus | Yes | Yes | No | No | Yes | No | No | Yes | Yes | Yes | No | Yes | No | No | No | No | **13** | **39.4** |
| Use of objective outcome measures consensus | Yes | Yes | Yes | Yes | Yes | Yes | Yes | Yes | Yes | Yes | Yes | Yes | Yes | Yes | Yes | Yes | **33** | **100.0** |
| Descriptive measures identified and reported for the primary outcome consensus | Yes | Yes | Yes | Yes | Yes | Unclear | Yes | Yes | Yes | Yes | Yes | Yes | Yes | Yes | Yes | Yes | **30** | **90.9** |
| Appropriate statistical analysis used consensus | Yes | Yes | Yes | Yes | Yes | Yes | Yes | Yes | Yes | Yes | Yes | Yes | Yes | Yes | Yes | Yes | **31** | **93.9** |
| Sample size calculation prior to initiation of the study consensus | Yes | Yes | Unclear | No | Yes | No | No | Yes | Yes | No | Yes | No | No | No | No | No | **15** | **45.5** |
| Adequate sample size consensus | Yes | Yes | Yes | Unclear | Yes | Unclear | Unclear | Unclear | Yes | Unclear | Unclear | Unclear | Unclear | Unclear | Unclear | Unclear | **10** | **30.3** |
| Sample size described for each group consensus | Yes | Yes | Unclear | Yes | Yes | No | Yes | Yes | Yes | Yes | Yes | Yes | Yes | Yes | Yes | Yes | **29** | **87.9** |
| Intention to treat analysis used consensus | No | No | No | Yes | No | No | Yes | No | Yes | No | Unclear | Yes | Unclear | No | No | No | **14** | **42.4** |
| Clinical significance reported consensus | No | No | No | No | No | No | No | No | No | No | No | No | No | No | No | No | **4** | **12.1** |
| **Number of items accomplished/total of applicable items** | **25** | **25** | **18** | **18** | **19** | **10** | **15** | **18** | **22** | **20** | **14** | **29** | **11** | **14** | **11** | **12** |  |  |
| **% of items accomplished** | **62.5** | **62.5** | **45** | **45** | **47.5** | **25** | **37.5** | **37.5** | **55** | **50** | **35** | **72.50** | **27.5** | **35** | **27.5** | **30** |  |  |

References:

1. Izquierdo Perez H. Alonso Perez JL. Gil Martinez A. et al. Is one better than another?: A randomized clinical trial of manual therapy for patients with chronic neck pain. *Man Ther*. Jun 2014;19(3):215-21. doi:10.1016/j.math.2013.12.002

2. Lopez-Lopez A. Alonso Perez JL. Gonzalez Gutierez JL. et al. Mobilization versus manipulations versus sustain apophyseal natural glide techniques and interaction with psychological factors for patients with chronic neck pain: randomized controlled trial. Randomized Controlled Trial. *Eur J Phys Rehabil Med*. Apr 2015;51(2):121-32.

3. Tachii R. sen s. Arfath U. Short term effect of sustained apohuseal glides on cervical joint position sense. pain. and neck disability in patients with chronic neck pain *International Journal of Therapies and Rehabilitation Research*. 2015;4(4):244.

4. Buyukturan O. Buyukturan B. Sas S. Kararti C. Ceylan I. The Effect of Mulligan Mobilization Technique in Older Adults with Neck Pain: A Randomized Controlled. Double-Blind Study. Randomized Controlled Trial. *Pain Res Manag*. 2018;2018:2856375. doi:<https://dx.doi.org/10.1155/2018/2856375>

5. Kumar D. Sandhu JS. Broota A. Efficacy of mulligan concept (NAGs) on pain at available end range in cervical spine: A randomised controlled trial. *Indian J Physiotherap Occup Ther Indian Journal of Physiotherapy and Occupational Therapy*. 2011;5(1):154-158.

6. Duymaz T. Yagci N. Effectiveness of the mulligan mobilization technique in mechanical neck pain. *Journal of Clinical and Analytical Medicine*. July 2018;9(4):304-309. doi:<http://dx.doi.org/10.4328/JCAM.5715>

7. Ali A. Shakil-Ur-Rehman S. Sibtain F. The efficacy of Sustained Natural Apophyseal Glides with and without Isometric Exercise Training in Non-specific Neck Pain. *Pakistan journal of medical sciences*. 2014;30(4):872-4.

8. Rezkallah SS. Abdullah GA. Comparison between sustained natural apophyseal glides (SNAG’s) and myofascial release techniques combined with exercises in non specific neck pain. *Physiotherapy Practice & Research*. 2018;39(2):135-145. doi:10.3233/PPR-180116

9. Tank K. Choksi P. Makwana P. To study the effect of muscle energy technique versus Mulligan SNAGs on pain. range of motion. and functional disability for individuals with mechanical neck pain: A comparative study. *International Journal of Physiotherapy and Research*. 2018;6(1):2582-2587.

10. Ganesh GS. Mohanty P. Pattnaik M. Mishra C. Effectiveness of mobilization therapy and exercises in mechanical neck pain. Comparative Study

Randomized Controlled Trial. *Physiother*. Feb 2015;31(2):99-106. doi:<https://dx.doi.org/10.3109/09593985.2014.963904>

11. Manzoor A. Anwar N. Khalid K. Haider R. Saghir M. Javed MA. Comparison of effectiveness of muscle energy technique with Mulligan mobilization in patients with non-specific neck pain. *J Pak Med Assoc*. Jun 2021;71(6):1532-1524. doi:10.47391/JPMA.981

12. Shamsi S. Alyazedi F. Abdelkader S. Khan S. Akhtar A. Efficacy of sustained natural apophyseal glides in the management of mechanical neck pain: A randomized clinical trial. *Indian Journal of Medical Specialities*. 2021;12(4)doi:10.4103/injms.injms_30_21

13. Alshami AM. AlSadiq AI. Outcomes of scapulothoracic mobilisation in patients with neck pain and scapular dyskinesis: A randomised clinical trial. *J Taibah Univ Med Sci*. Aug 2021;16(4):540-549. doi:10.1016/j.jtumed.2021.03.006

14. Alansari SM. Youssef EF. Shanb AA. Efficacy of manual therapy on psychological status and pain in patients with neck pain. A randomized clinical trial. *Saudi Med J*. Jan 2021;42(1):82-90. doi:10.15537/smj.2021.1.25589

15. Vijayan K. Sivaraman A. Kumaresan P. Palani J. Short-term Effect of Mulligan SNAGs on Pain Intensity. Cervical Range of Motion and Craniovertebral Angle in Patients with Non Specific Neck Pain: A Quasi-experimental Study. *Journal of Clinical and Diagnostic Research*. July 2022;16(7):YC05-YC08. doi:<https://dx.doi.org/10.7860/JCDR/2022/55962.16547>

16. Usama J. Iram A. Sania M. et al. Comparative Effect of Muscle Energy Techniques and Mulligan Mobilization on Pain & Range of Motion in patients with Mechanical Neck Pain. CrossLinks International Publishers; 2022. p. 195-199.

17. Abd El-Azeim AS. Grase MO. Efficacy of Mulligan on electromyography activation of cervical muscles in mechanical neck pain: randomized experimental trial. *Physiotherapy Quarterly*. 2023;31(4)doi:<https://dx.doi.org/10.5114/pq.2023.117224>

18. Morsi AA. Al-Kabalawy MA. Aneis YM. Hamza MS. Atta HK. Effect of Sustained Natural Apophyseal Glides and Myofascial Release on Chronic Nonspecific Neck Pain: Randomized Controlled Trial. *Journal of Population Therapeutics and Clinical Pharmacology*. 18 Apr 2023;30(8):e390-e404. doi:<https://dx.doi.org/10.47750/jptcp.2023.30.08.042>

19. Shelke A. Prabhu BA. Balthillaya MG. Kumaran SD. Raja GP. Immediate effect of craniocervical flexion exercise and Mulligan mobilisation in patients with mechanical neck pain - A randomised clinical trial. *Hong Kong Physiotherapy Journal*. 01 Dec 2023;43(2):137-147. doi:<https://dx.doi.org/10.1142/S1013702523500154>

20. Sun X. Chai L. Huang Q. Zhou H. Liu H. Effects of exercise combined with cervicothoracic spine self-mobilization on chronic non-specific neck pain. *Scientific reports*. 04 Mar 2024;14(1):5298. doi:<https://dx.doi.org/10.1038/s41598-024-55181-8>

21. Ozlu O. Sahin M. The effect of mulligan mobilization technique application in addition to conventional physiotherapy on pain and joint range of motion in people with neck pain. *Journal of Bodywork and Movement Therapies*. July 2024;39:225-230. doi:<https://dx.doi.org/10.1016/j.jbmt.2024.02.009>

22. Aggarwal S. Verma M. Efficacy of Mulligan’s self-sustained natural apophyseal glides in decreasing pain. disability. and improving neck mobility among the nursing professional suffering from work-related neck pain. *Arch Med Health Sci*. 2018;6(1):48-53.

23. El-Sodany AM. Alayat MSM. Zafer AMI. Sustained natural apophyseal glides mobilization versus manipulation in the treatment of cervical spine disorders: a randomized controlled trial. *International Journal*. 2014;2(6):274-280.

24. Gautam R. Dhamija JK. Puri A. Trivedi P. Sathiyavani D. Nambi G. Comparison of Maitland and Mulligan mobilization in improving neck pain. ROM and disability. *Int J Physiother Res*. 2014;2(3):482-487.

25. Hussain SI. Ahmad A. Amjad F. Shafi T. Shahid HA. Effectiveness of natural apophyseal glides versus grade I and II Maitland mobilization in Non-specific neck pain. *Annals of King Edward Medical University Lahore Pakistan*. 2016;22:23-29.

26. Mohamed EE. Elrazik RKA. Sustained natural apophyseal glides versus positional release therapy in the treatment of chronic mechanical neck dysfunction. Article. *International Journal of Human Movement and Sports Sciences*. 2020;8(6):384-394. doi:10.13189/saj.2020.080610

27. Said MS. Ali OI. Elazm SNA. Abdelraoof NA. Mulligan self mobilization versus Mulligan snags on cervical position sense. *International Journal of Physiotherapy*. 2017;4(2):93-100.

28. Sultan N. Khushnood K. Altaf S. Awan MMA. Qureshi S. Mehmood R. Muscle Energy Technique Augmented with Sustained Natural Apophyseal Glides; An Effective Way to Improve Mechanical Neck Pain and Range of Motion: A Randomized Control Trial. Article. *Journal of Islamic International Medical College*. 2021;16(2):96-100.

29. Tanveer F. Afzal M. Adeel S. Shahid S. Masood M. Comparison of sustained natural apophyseal glides and maitland manual therapy in non-specific neck pain on numeric pain rating scale and neck disability index. *Annals of King Edward Medical University*. 2017;23

30. Zemadanis K. The short and mid-term effects of Mulligan concept in patients with chronic mechanical neck pain. *J Nov Physiother Rehabil*. 2018;2(2):022-021.

31. Waqas S. Shah SHA. Zafar U. Akhtar MF. Comparison of Mulligan Sustained Natural Apophyseal Glides Versus Mulligan Natural Apophyseal Glides in Mechanical Neck Pain. . *Annals of King Edward Medical University*. 2017;23(3)doi:<https://doi.org/10.21649/akemu.v23i3.2007>

32. Keyur MP. Balaganapathy M. Hinal MP. Effect of Maitland mobilization versus mulligan (Snags) mobilization on head repositioning accuracy (Hra). pain and functional disability in patients with chronic neck pain‑A randomized controlled clinical trial. *Int J Curr Res*. 2016;8:31144‑9.

33. Shehri AA. Khan S. Shami S. Almureef SS. COMPARATIVE STUDY OF MULLIGAN (SNAGS) AND MAITLAND MOBILIZATION IN NECK PAIN. *European Journal of Physical Education and Sport Science*. 2018;5(1):19-29. doi:doi: 10.5281/zenodo.1481977

34. Pal A. Misra A. EFFECTIVENESS OF SNAG MOBILIZATION ON COMPUTER PROFESSIONALS WITH MECHANICAL NECK PAIN AND MOBILITY DEFICIT. *International Journal of Physiotherapy and Research*. 2019;7(2):3022-27. doi:<https://dx.doi.org/10.16965/ijpr.2019.104>
