# Supplementary material for: The Effectiveness of Mulligan's Techniques in Non‐Specific Neck Pain: A Systematic Review and Meta‐Analysis
Source: Physiother Res Int. 2025 May 29;30(3):e70045. doi: 10.1002/pri.70045 (PMC12121345; doi:10.1002/pri.70045)
Supplement: Supplementary file 6 — Supporting Information S6 [file PRI-30-e70045-s005.docx]

**Appendix 6. Assessment of the certainty of evidence according to GRADE**

| Comparison Nº | | Comparison | | Outcome | Time-point | Number of studies (design) | Risk of bias | Inconsistency | Indirectness | Imprecision | Other considerations | Intervention  (n) | Comparator (n) | Effect Estimate  (SMD) | MD (95%CI) | Favor | Certainty |
| --- | --- | --- | --- | --- | --- | --- | --- | --- | --- | --- | --- | --- | --- | --- | --- | --- | --- |
|  |  | | **Acute neck pain** | | | | | | | | | | | | | | |
|  |  | | **Pain Intensity** | | | | | | | | | | | | | | |
| 6.1 | | SNAGs + CT + exercises vs. MET + CT + exercises | | Pain Intensity | 2 weeks | 1 RCT  (Tank et al., 2018) | very serious^a^ | NA | not serious | very serious^b,c^ | none | 20 | 20 | 0.97 (0.31, 1.63) | 0.57 (0.21, 0.93) | MET + CT + ex | ⨁◯◯◯ Very low |
| 6.2 | | SNAG + exercises vs. mobilization (PAIVMs) and exercises | | Pain intensity | 2 weeks | 1 RCT  (Ganesh et al., 2015) | very serious^a^ | NA | not serious | very serious^b,c^ | none | 22 | 24 | -0.45 (-1.03, 0.14) | -0.60 (-1.37, 0.17) | Significant improvement in both groups, no statistical differences | ⨁◯◯◯ Very low |
| 6.3 | | SNAG + exercises vs. exercises | | Pain intensity | 2 weeks | 1 RCT  (Ganesh et al., 2015) | very serious^a^ | NA | not serious | very serious^b,c^ | none | 22 | 20 | -0.09 (-0.69, 0.52) | -0.10 (-0.80, 0.60) | Significant improvement in both groups, no statistical differences | ⨁◯◯◯ Very low |
| 6.4 | | SNAG + exercises vs. exercises | | Pain intensity | 12 weeks | 1 RCT  (Ganesh et al., 2015) | very serious^a^ | NA | not serious | very serious^b,c^ | none | 20 | 20 | 0.32 (-0.30, 0.95) | 0.30 (-0.26, 0.86) | Significant improvement in both groups, no statistical differences | ⨁◯◯◯ Very low |
| 6.5 | | SNAG + exercises vs. mobilization (PAIVMs) and exercises | | Pain intensity | 12 weeks | 1 RCT  (Ganesh et al., 2015) | very serious^a^ | NA | not serious | very serious^b,c^ | none | 20 | 20 | -0.59 (-1.23, 0.04) | -0.70 (-1.42, 0.02) | Significant improvement in both groups, no statistical differences | ⨁◯◯◯ Very low |
|  |  | | **Cervical Range of Motion** | | | | | | | | | | | | | | |
| 6.6 | | SNAGs vs. MET + CT | | Flexion | 2 weeks | 1 RCT  (Tank et al., 2018) | very serious^a^ | NA | not serious | very serious^b,c^ | none | 20 | 20 | 0.09 (-0.53, 0.71) | 0.46 (-2.54, 3.46) | Significant improvement in both groups, no statistical differences | ⨁◯◯◯ Very low |
| 6.7 | | SNAG + exercises vs. mobilization (PAIVMs) and exercises | | Extension | 2 weeks | 1 RCT  (Ganesh et al., 2015) | very serious^a^ | NA | not serious | very serious^b,c^ | none | 22 | 24 | -0.49 (-1.08, 0.10) | -3.00 (-6.47, 0.47) | Significant improvement in both groups, no statistical differences | ⨁◯◯◯ Very low |
| 6.8 | | SNAG + exercises vs. exercises | | Extension | 2 weeks | 1 RCT  (Ganesh et al., 2015) | very serious^a^ | NA | not serious | very serious^b,c^ | none | 22 | 20 | 0 (-0.58, 0.58) | 0 (-3.18, 3.18) | Significant improvement in both groups, no statistical differences | ⨁◯◯◯ Very low |
| 6.9 | | SNAGs vs. MET + CT | | Extension | 2 weeks | 1 RCT  (Tank et al., 2018) | very serious^a^ | NA | not serious | very serious^b,c^ | none | 20 | 20 | 0.20 (-0.42, 0.82) | 0.84 (-1.67, 3.35) | Significant improvement in both groups, no statistical differences | ⨁◯◯◯ Very low |
| 6.10 | | SNAG + exercises vs. mobilization (PAIVMs) and exercises | | Extension | 12 weeks | 1 RCT  (Ganesh et al., 2015) | very serious^a^ | NA | not serious | very serious^b,c^ | none | 20 | 20 | -0.53 (-1.16, 0.10) | -3.00 (-6.42, 0.42) | Significant improvement in both groups, no statistical differences | ⨁◯◯◯ Very low |
| 6.11 | | SNAG + exercises vs. exercises | | Extension | 12 weeks | 1 RCT  (Ganesh et al., 2015) | very serious^a^ | NA | not serious | very serious^b,c^ | none | 20 | 20 | -0.49 (-1.12, 0.14) | 0 (-3.42, 3.42) | Significant improvement in both groups, no statistical differences | ⨁◯◯◯ Very low |
| 6.12 | | SNAG + exercises vs. mobilization (PAIVMs) and exercises | | Left lateral flexion | 2 weeks | 1 RCT  (Ganesh et al., 2015) | very serious^a^ | NA | not serious | very serious^b,c^ | none | 22 | 24 | -0.39 (-0.98, 0.19) | 2.00 (-2.96, 6.95) | Significant improvement in both groups, no statistical differences | ⨁◯◯◯ Very low |
| 6.13 | | SNAG + exercises vs. exercises | | Left lateral flexion | 2 weeks | 1 RCT  (Ganesh et al., 2015) | very serious^a^ | NA | not serious | very serious^b,c^ | none | 22 | 20 | -0.26 (-0.84, 0.32) | -2.00 (-6.34, 2.34) | Significant improvement in both groups, no statistical differences | ⨁◯◯◯ Very low |
| 6.14 | | SNAGs vs. MET + CT | | Left lateral-flexion | 2 weeks | 1 RCT  (Tank et al., 2018) | very serious^a^ | NA | not serious | very serious^b,c^ | none | 20 | 20 | 0.35 (-0.27, 0.98) | 1.22 (-0.88, 3.32) | Significant improvement in both groups, no statistical differences | ⨁◯◯◯ Very low |
| 6.15 | | SNAG + exercises vs. mobilization (PAIVMs) and exercises | | Left lateral flexion | 12 weeks | 1 RCT  (Ganesh et al., 2015) | very serious^a^ | NA | not serious | very serious^b,c^ | none | 20 | 20 | 0.25 (-0.38, 0.87) | 2.00 (-2.96, 6.96) | Significant improvement in both groups, no statistical differences | ⨁◯◯◯ Very low |
| 6.16 | | SNAG + exercises vs. exercises | | Left lateral flexion | 12 weeks | 1 RCT  (Ganesh et al., 2015) | very serious^a^ | NA | not serious | very serious^b,c^ | none | 20 | 20 | 0 (-0.62, 0.62) | 0 (-4.96, 4.96) | Significant improvement in both groups, no statistical differences | ⨁◯◯◯ Very low |
| 6.17 | | SNAG + exercises vs. mobilization (PAIVMs) and exercises | | Right lateral flexion | 2 weeks | 1 RCT  (Ganesh et al., 2015) | very serious^a^ | NA | not serious | very serious^b,c^ | none | 22 | 24 | 0 (-0.58, 0.58) | 0 (-4.34, 4.34) | Significant improvement in both groups, no statistical differences | ⨁◯◯◯ Very low |
| 6.18 | | SNAG + exercises vs. exercises | | Right lateral flexion | 2 weeks | 1 RCT  (Ganesh et al., 2015) | very serious^a^ | NA | not serious | very serious^b,c^ | none | 22 | 20 | -0.14 (-0.72, 0.44) | -1.00 (-5.03, 3.05) | Significant improvement in both groups, no statistical differences | ⨁◯◯◯ Very low |
| 6.19 | | SNAGs vs. MET + CT | | Right lateral-flexion | 2 weeks | 1 RCT  (Tank et al., 2018) | very serious^a^ | NA | not serious | very serious^b,c^ | none | 20 | 20 | 0.43 (-0.19, 1.06) | 1.55 (-0.62, 3.72) | Significant improvement in both groups, no statistical differences | ⨁◯◯◯ Very low |
| 6.20 | | SNAG + exercises vs. mobilization (PAIVMs) and exercises | | Right lateral flexion | 12 weeks | 1 RCT  (Ganesh et al., 2015) | very serious^a^ | NA | not serious | very serious^b,c^ | none | 20 | 20 | 0 (-0.62, 0.62) | 0 (-4.66, 4.66) | Significant improvement in both groups, no statistical differences | ⨁◯◯◯ Very low |
| 6.21 | | SNAG + exercises vs. exercises | | Right lateral flexion | 12 weeks | 1 RCT  (Ganesh et al., 2015) | very serious^a^ | NA | not serious | very serious^b,c^ | none | 20 | 20 | 0 (-0.62, 0.62) | 0 (-5.28, 5.28) | Significant improvement in both groups, no statistical differences | ⨁◯◯◯ Very low |
| 6.22 | | SNAG + exercises vs. mobilization (PAIVMs) and exercises | | Left rotation | 2 weeks | 1 RCT  (Ganesh et al., 2015) | very serious^a^ | NA | not serious | very serious^b,c^ | none | 22 | 24 | -0.26 (-0.84, 0.32) | 2.00 (-6.34, 2.34) | Significant improvement in both groups, no statistical differences | ⨁◯◯◯ Very low |
| 6.23 | | SNAG + exercises vs. exercises | | Left rotation | 2 weeks | 1 RCT  (Ganesh et al., 2015) | very serious^a^ | NA | not serious | very serious^b,c^ | none | 22 | 20 | -0.14 (-0.72, 0.44) | -1.00 (-5.03, 3.05) | Significant improvement in both groups, no statistical differences | ⨁◯◯◯ Very low |
| 6.24 | | SNAGs vs. MET + CT | | Left rotation | 2 weeks | 1 RCT  (Tank et al., 2018) | very serious^a^ | NA | not serious | very serious^b,c^ | none | 20 | 20 | -0.43 (-1.05, 0.20) | -2.11 (-5.12,0.90) | Significant improvement in both groups, no statistical differences | ⨁◯◯◯ Very low |
| 6.25 | | SNAG + exercises vs. mobilization (PAIVMs) and exercises | | Left rotation | 12 weeks | 1 RCT  (Ganesh et al., 2015) | very serious^a^ | NA | not serious | very serious^b,c^ | none | 20 | 20 | 0.14 (-0.48, 0.76) | 1.00 (-3.34, 5.34) | Significant improvement in both groups, no statistical differences | ⨁◯◯◯ Very low |
| 6.26 | | SNAG + exercises vs. exercises | | Left rotation | 12 weeks | 1 RCT  (Ganesh et al., 2015) | very serious^a^ | NA | not serious | very serious^b,c^ | none | 20 | 20 | 0 (-0.62, 0.62) | 0 (-4.34, 4.34) | Significant improvement in both groups, no statistical differences | ⨁◯◯◯ Very low |
| 6.27 | | SNAG + exercises vs. mobilization (PAIVMs) and exercises | | Right rotation | 2 weeks | 1 RCT  (Ganesh et al., 2015) | very serious^a^ | NA | not serious | very serious^b,c^ | none | 22 | 24 | -0.37 (-0.99, 0.26) | -1.00 (-5.91, 3.91) | Significant improvement in both groups, no statistical differences | ⨁◯◯◯ Very low |
| 6.28 | | SNAG + exercises vs. exercises | | Right rotation | 2 weeks | 1 RCT  (Ganesh et al., 2015) | very serious^a^ | NA | not serious | very serious^b,c^ | none | 22 | 20 | 0 (-0.58, 0.58) | 0 (-4.34, 4.34) | Significant improvement in both groups, no statistical differences | ⨁◯◯◯ Very low |
| 6.29 | | SNAGs vs. MET + CT | | Right rotation | 2 weeks | 1 RCT  (Tank et al., 2018) | very serious^a^ | NA | not serious | very serious^b,c^ | none | 20 | 20 | 0.22 (-0.41, 0.84) | 1.11 (-2.02, 4.24) | Significant improvement in both groups, no statistical differences | ⨁◯◯◯ Very low |
| 6.30 | | SNAG + exercises vs. mobilization (PAIVMs) and exercises | | Right rotation | 12 weeks | 1 RCT  (Ganesh et al., 2015) | very serious^a^ | NA | not serious | very serious^b,c^ | none | 20 | 20 | -0.37 (-0.99, 0.26) | -3.00 (-7.96, 1.96) | Significant improvement in both groups, no statistical differences | ⨁◯◯◯ Very low |
| 6.31 | | SNAG + exercises vs. exercises | | Right rotation | 12 weeks | 1 RCT  (Ganesh et al., 2015) | very serious^a^ | NA | not serious | very serious^b,c^ | none | 20 | 20 | -0.26 (-0.88, 0.36) | -2.00 (-6.66, 2.66) | Significant improvement in both groups, no statistical differences | ⨁◯◯◯ Very low |
|  |  | | **Disability** | | | | | | | | | | | | | | |
| 6.32 | | SNAG + exercises vs. mobilization (PAIVMs) and exercises | | Disability | 2 weeks | 1 RCT  (Ganesh et al., 2015) | very serious^a^ | NA | not serious | very serious^b,c^ | none | 22 | 24 | -0.21 (-0.79, 0.37) | -2.30 (-8.44, 3.84) | Significant improvement in both groups, no statistical differences | ⨁◯◯◯ Very low |
| 6.33 | | SNAG + exercises vs. exercises | | Disability | 2 weeks | 1 RCT  (Ganesh et al., 2015) | very serious^a^ | NA | not serious | very serious^b,c^ | none | 22 | 20 | 0.58 (-0.02, 1.17) | 4.70 (0.15, 9.25) | Significant improvement in both groups, no statistical differences | ⨁◯◯◯ Very low |
| 6.34 | | SNAGs vs. MET + CT | | Disability | 2 weeks | 1 RCT  (Tank et al., 2018) | very serious^a^ | NA | not serious | very serious^b,c^ | none | 20 | 20 | 0.07 (-0.55, 0.69) | 0.47 (-3.73, 4.67) | Significant improvement in both groups, no statistical differences | ⨁◯◯◯ Very low |
| 6.35 | | SNAG + exercises vs. mobilization (PAIVMs) and exercises | | Disability | 12 weeks | 1 RCT  (Ganesh et al., 2015) | very serious^a^ | NA | not serious | very serious^b,c^ | none | 20 | 20 | -0.47 (-1.10, 0.16) | -3.80 (-8.72, 1.12) | Significant improvement in both groups, no statistical differences | ⨁◯◯◯ Very low |
| 6.36 | | SNAG + exercises vs. exercises | | Disability | 12 weeks | 1 RCT  (Ganesh et al., 2015) | very serious^a^ | NA | not serious | very serious^b,c^ | none | 20 | 20 | 0.59 (-0.05, 1.22) | 2.70 (-0.08, 5.48) | Significant improvement in both groups, no statistical differences | ⨁◯◯◯ Very low |
|  |  | | **Acute, subacute, and chronic neck pain** | | | | | | | | | | | | | | |
| 6.37 | | SNAGs vs. mobilization (PAIVMs) | | Pain intensity | 3 weeks | 1 RCT  (Alansari et al., 2021) | very serious^a^ | NA | not serious | very serious^b^ | none | 22 | 22 | -0.04 (-0.63, 0.55) | -0.09 (-1.31, 1.13) | Significant improvement in both groups, no statistical differences | ⨁◯◯◯ Very low |
| 6.38 | | SNAGs vs. mobilization (PAIVMs) | | Disability | 3 weeks | 1 RCT  (Alansari et al., 2021) | very serious^a^ | NA | not serious | very serious^b^ | none | 22 | 22 | -0.16 (-0.75, 0.43) | 2.11 (-5.46, 9.68) | Significant improvement in both groups, no statistical differences | ⨁◯◯◯ Very low |
| 6.39 | | SNAGs vs. CCFT | | Pain intensity | Right after treatment | 1 RCT  (Shelke et al., 2023)h | very serious^a^ | NA | not serious | very serious^b^ | none | 13 | 13 | -0.19 (-0.96, 0.58) | -0.33 (-1.61, 0.95) | Significant improvement in both groups, no statistical differences | ⨁◯◯◯ Very low |
| 6.40 | | SNAGs vs. CCFT | | Flexion | Right after treatment | 1 RCT  (Shelke et al., 2023) | very serious^a^ | NA | not serious | very serious^b^ | none | 13 | 13 | 0.20 (-0.57, 0.97) | 2.33 (-6.34, 11.0) | Significant improvement in both groups, no statistical differences | ⨁◯◯◯ Very low |
| 6.41 | | SNAGs vs. CCFT | | Extension | Right after treatment | 1 RCT  (Shelke et al., 2023) | very serious^a^ | NA | not serious | very serious^b^ | none | 13 | 13 | 0.47 (-0.31, 1.25) | 5.33 (-3.18, 13.84) | Significant improvement in both groups, no statistical differences | ⨁◯◯◯ Very low |
| 6.42 | | SNAGs vs. CCFT | | Left side flexion | Right after treatment | 1 RCT  (Shelke et al., 2023) | very serious^a^ | NA | not serious | very serious^b^ | none | 13 | 13 | -0.26 (-1.03, 0.51) | -2.33 (-9.04, 4.38) | Significant improvement in both groups, no statistical differences | ⨁◯◯◯ Very low |
| 6.43 | | SNAGs vs. CCFT | | Right side flexion | Right after treatment | 1 RCT  (Shelke et al., 2023) | very serious^a^ | NA | not serious | very serious^b^ | none | 13 | 13 | -0.07 (-.84, 0.70) | -0.66 (-7.53, 6.21) | Significant improvement in both groups, no statistical differences | ⨁◯◯◯ Very low |
| 6.44 | | SNAGs vs. CCFT | | Left rotation | Right after treatment | 1 RCT  (Shelke et al., 2023) | very serious^a^ | NA | not serious | very serious^b^ | none | 13 | 13 | -0.27 (-1.04, 0.50) | -4.67 (-17.50, 8.16) | Significant improvement in both groups, no statistical differences | ⨁◯◯◯ Very low |
| 6.45 | | SNAGs vs. CCFT | | Right rotation | Right after treatment | 1 RCT  (Shelke et al., 2023) | very serious^a^ | NA | not serious | very serious^b^ | none | 13 | 13 | 0 | 0 (-8.51, 8.51) | Significant improvement in both groups, no statistical differences | ⨁◯◯◯ Very low |
| 6.46 | | SNAGs + Interferential therapy + Isometric neck exercises vs. Interferential therapy + Isometric neck exercises | | Pain intensity | 2 weeks | 1 RCT  (Vijayan et al., 2022) | very serious^a^ | NA | not serious | very serious^b^ | none | 10 | 10 | -0.86 (-1.79, 0.06) | -1.00 (-1.97, -0.03) | Significant improvement in both groups but significantly greater effect in the Mulligan group  (SNAGs + Interferential therapy + Isometric neck exercises) | ⨁◯◯◯ Very low |
| 6.47 | | SNAGs + Interferential therapy + Isometric neck exercises vs. Interferential therapy + Isometric neck exercises | | Flexion | 2 weeks | 1 RCT  (Vijayan et al., 2022) | very serious^a^ | NA | not serious | very serious^b^ | none | 10 | 10 | 1.02 (0.08, 1.97) | 7.50 (1.33, 13.67) | SNAGs + Interferential therapy + Isometric neck exercises | ⨁◯◯◯ Very low |
| 6.48 | | SNAGs + Interferential therapy + Isometric neck exercises vs. Interferential therapy + Isometric neck exercises | | Extension | 2 weeks | 1 RCT  (Vijayan et al., 2022) | very serious^a^ | NA | not serious | very serious^b^ | none | 10 | 10 | 1.72 (0.66, 2.78) | 10 (5.13, 14.87) | SNAGs + Interferential therapy + Isometric neck exercises | ⨁◯◯◯ Very low |
| 6.49 | | SNAGs + Interferential therapy + Isometric neck exercises vs. Interferential therapy + Isometric neck exercises | | Right rotation | 2 weeks | 1 RCT  (Vijayan et al., 2022) | very serious^a^ | NA | not serious | very serious^b^ | none | 10 | 10 | -1.18 (-2.15, -0.21) | -8.00 (-13.70, -2.30) | Interferential therapy + Isometric neck exercises | ⨁◯◯◯ Very low |
| 6.50 | | SNAGs + Interferential therapy + Isometric neck exercises vs. Interferential therapy + Isometric neck exercises | | Left rotation | 2 weeks | 1 RCT  (Vijayan et al., 2022) | very serious^a^ | NA | not serious | very serious^b^ | none | 10 | 10 | 0.91 (-0.02, 1.84) | 5 (0.38, 9.62) | Significant improvement in both groups but significantly greater effect in the Mulligan group | ⨁◯◯◯ Very low |
| 6.51 | | NAGS + CT vs Maitland + CT | | Pain | 2 weeks | 1 RCT  (Hussain et al., 2016) | very serious^a^ | NA | not serious | very serious^b^ | none | 25 | 25 | -0.46 (-1.02, 0.10) | -0.88 (-1.93, 0.17) | No significant differences | ⨁◯◯◯ Very low |
| 6.52 | | NAGS + CT vs Maitland + CT | | Pain | 4 weeks | 1 RCT  (Hussain et al., 2016) | very serious^a^ | NA | not serious | very serious^b^ | none | 25 | 25 | -0.56 (-1.13, 0.01) | -1.32 (-2.61, -0.03) | NAGS + CT | ⨁◯◯◯ Very low |
| 6.53 | | NAGS + CT vs Maitland + CT | | Disability | 2 weeks | 1 RCT  (Hussain et al., 2016) | very serious^a^ | NA | not serious | very serious^b^ | none | 25 | 25 | -0.46 (-1.02, 0.10) | -3.20 (-7.00, 0.60) | No significant differences | ⨁◯◯◯ Very low |
| 6.54 | | NAGS + CT vs Maitland + CT | | Disability | 4 weeks | 1 RCT  (Hussain et al., 2016) | very serious^a^ | NA | not serious | very serious^b^ | none | 25 | 25 | -0.68 (-1.26, -0.11) | -3.44 (-6.18, -0.70) | NAGS + CT | ⨁◯◯◯ Very low |
| 6.55 | | SNAGs + exercise vs manipulation + exercise program | | Pain | 6 weeks | 1 RCT  (El-Sodany et al., 2014) | very serious^a^ | NA | not serious | very serious^b^ | none | 18 | 15 | NR | NR | Significant improvement in both groups, impossible to identify the effects, no numerical data available | ⨁◯◯◯ Very low |
| 6.56 | | SNAGs + exercise vs manipulation + exercise program | | Cervical range of motion | 6 weeks | 1 RCT  (El-Sodany et al., 2014) | very serious^a^ | NA | not serious | very serious^b^ | none | 18 | 15 | NR | NR | Significant improvement in both groups, no statistical differences. No numerical data available. | ⨁◯◯◯ Very low |
| 6.57 | | SNAGs + exercise vs manipulatio + exercise program | | Disability | 6 weeks | 1 RCT  (El-Sodany et al., 2014) | very serious^a^ | NA | not serious | very serious^b^ | none | 18 | 15 | NR | NR | Significant improvement in both groups, impossible to identify the effects, no numerical data available | ⨁◯◯◯ Very low |
| 6.58 | | SNAGs + exercise vs vs exercise | | Pain | 6 weeks | 1 RCT  (El-Sodany et al., 2014) | very serious^a^ | NA | not serious | very serious^b^ | none | 18 | 16 | NR | NR | Significant improvement in both groups, impossible to identify the effects, no numerical data available | ⨁◯◯◯ Very low |
| 6.59 | | SNAGs + exercise vs vs exercise | | Cervical range of motion | 6 weeks | 1 RCT  (El-Sodany et al., 2014) | very serious^a^ | NA | not serious | very serious^b^ | none | 18 | 15 | NR | NR | Significant differences, unclear to identify the effects, no numerical data available | ⨁◯◯◯ Very low |
| 6.60 | | SNAGs + exercise vs vs exercise | | Disability | 6 weeks | 1 RCT  (El-Sodany et al., 2014) | very serious^a^ | NA | not serious | very serious^b^ | none | 18 | 16 | NR | NR | Significant improvement in both groups, impossible to identify the effects, no numerical data available | ⨁◯◯◯ Very low |
| 6.61 | | SNAGs + exercise vs manipulation + exercise program | | Pain | 10 weeks | 1 RCT  (El-Sodany et al., 2014) | very serious^a^ | NA | not serious | very serious^b^ | none | 18 | 16 | NR | NR | Significant improvement in both groups, impossible to identify the effects, no numerical data available | ⨁◯◯◯ Very low |
| 6.62 | | SNAGs + exercise vs manipulation + exercise program | | Cervical range of motion | 10 weeks | 1 RCT  (El-Sodany et al., 2014) | very serious^a^ | NA | not serious | very serious^b^ | none | 18 | 16 | NR | NR | Significant improvement in both groups, no statistical differences | ⨁◯◯◯ Very low |
| 6.63 | | SNAGs + exercise vs manipulation + exercise program | | Disability | 10 weeks | 1 RCT  (El-Sodany et al., 2014) | very serious^a^ | NA | not serious | very serious^b^ | none | 18 | 16 | NR | NR | Significant improvement in both groups, impossible to identify the effects, no numerical data available | ⨁◯◯◯ Very low |
| 6.64 | | SNAGs + exercise vs vs exercise | | Pain | 10 weeks | 1 RCT  (El-Sodany et al., 2014) | very serious^a^ | NA | not serious | very serious^b^ | none | 18 | 16 | NR | NR | Significant improvement in both groups, impossible to identify the effects, no numerical data available | ⨁◯◯◯ Very low |
| 6.65 | | SNAGs + exercise vs vs exercise | | Cervical range of motion | 10 weeks | 1 RCT  (El-Sodany et al., 2014) | very serious^a^ | NA | not serious | very serious^b^ | none | 18 | 16 | NR | NR | Significant improvement in both groups, significant different between groups but impossible to identify the effects, no numerical data available | ⨁◯◯◯ Very low |
| 6.66 | | SNAGs + exercise vs vs exercise | | Disability | 10 weeks | 1 RCT  (El-Sodany et al., 2014) | very serious^a^ | NA | not serious | very serious^b^ | none | 18 | 16 | NR | NR | Significant improvement in both groups, impossible to identify the effects, no numerical data available | ⨁◯◯◯ Very low |
|  | **Chronic neck pain** | | | | | | | | | | | | | | | | |
|  |  |  |  |  |  |  |  |  |  |  |  |  |  |  |  |  |  |
| Pain intensity | | | | |  |  |  |  |  |  |  |  |  |  |  |  |  |
| 6.67 | | NAGs vs. placebo | | Pain | Unclear | 1 RCT  (Kumar et al., 2011) | very serious^a^ | NA | not serious | very serious^b^ | none | 75 | 25 | NR | NR | NAG | ⨁◯◯◯ Very low |
| 6.68 | | SNAGs vs. HVLA | | Pain | Right after treatment | 1 RCT^j^  (Izquierdo Perez et al., 2014; Lopez-Lopez et al., 2015) | serious^d^ | NA | not serious | very serious^b,c^ | none | 38 | 34 | 2.17 (-1.66, 5.99) | 1.34 (-0.07, 2.76) | Significant improvement in both groups, no statistical differences. Large effect size favoring HVLA | ⨁◯◯◯ Very low |
| 6.69 | | SNAGs vs. mobilization (PAIVMs) | | Pain | Right after treatment | 1 RCT^j^  (Izquierdo Perez et al., 2014; Lopez-Lopez et al., 2015) | serious^d^ | NA | not serious | very serious^b^ | none | 38 | 37 | 2.65 (-1.96, 7.26) | 1.53 (0.18, 2.88) | SNAGs | ⨁◯◯◯ Very low |
| 6.70 | | SNAGs + CT + exercises vs. CT + exercises | | Pain | 2 weeks | 3 RCTs  (Buyukturan et al., 2018; Shamsi et al., 2021; Tachii et al., 2015) | very serious^h^ | very serious ^f^ | not serious | very serious^b^ | none | 86 | 84 | -0.90 (-0.61, 0.00) | -0.30 (-0.56, -0.03) | SNAGs + CT + ex | ⨁◯◯◯ Very low |
|  |  | SNAGs + CT + exercises vs. CT + exercises  ***sensitivy analisys** | | Pain | 2/4 weeks | 4 RCTs  (Abd El-Azeim & Grase, 2023; Buyukturan et al., 2018; Shamsi et al., 2021; Tachii et al., 2015) | very serious^h^ | very serious ^f^ | not serious | very serious^b^ | none | 131 | 129 | -1.52 (-3.38, 0.35) | -1.28 (-3.70, 1.13) | Significant improvement in both groups, no statistical differences but potentially clinically significant, large ES favouring the intervention group (SNAGs + CT + exercises) | ⨁◯◯◯ Very low |
| 6.71 | | MWM for the scapula vs. CT + taping | | Pain | 2 weeks | 1 RCT  (Alshami & AlSadiq, 2021) | very serious^a^ | NA | not serious | very serious^b^ | none | 20 | 20 | 0 (-0.62, 0.62) | 0 (-1.21, 1.21) | Significant improvement in both groups, no statistical differences | ⨁◯◯◯ Very low |
| 6.72 | | MWM for the scapula vs. CT + taping | | Pain | 3 weeks | 1 RCT  (Alshami & AlSadiq, 2021) | very serious^a^ | NA | not serious | very serious^b^ | none | 20 | 20 | -0.29 (-0.92, 0.33) | -0.60 (-1.67, 0.47) | Significant improvement in both groups, no statistical differences | ⨁◯◯◯ Very low |
| 6.73 | | SNAGs + NAGs vs. MET | | Pain | 3 weeks | 1 RCT  (Manzoor et al., 2021) | very serious^a^ | NA | not serious | very serious^b^ | none | 28 | 28 | -0.59 (-1.13, -0.06) | -0.65 (-1.22, -0.08) | SNAGs + NAGs | ⨁◯◯◯ Very low |
| 6.74 | | SNAG+NAG+self-SNAG vs. Sham | | Pain | 3 weeks | 1 RCT  (Zemadanis, 2018) | very serious^a^ | NA | not serious | very serious^b^ | none | 20 | 20 | -2.66 (-3.53, -1.78) | -2.55 (-3.13, -1.97) | SNAG+NAG+self-SNAG | ⨁◯◯◯ Very low |
| 6.75 | | SNAGs + exercises vs. exercises | | Pain | 2-4 weeks | 2 RCTs  (Duymaz & Yagci, 2018; Rezkallah & Abdullah, 2018) | very serious^a^ | serious^g^ | not serious | very serious^b,c^ | none | 45 | 42 | -2.38 (-3.07, -1.69) | -22.59 (-62.62, 17.44) | Significant improvement in both groups, no statistical differences, potentially clinically relevant with large ES favouring the intervention group (SNAGs + exercises) | ⨁◯◯◯ Very low |
| 6.76 | | SNAGs + exercises vs. MFR + exercises | | Pain | 4 weeks | 1 RCT  (Rezkallah & Abdullah, 2018) | very serious^a^ | NA | not serious | very serious^b^ | none | 25 | 23 | 0.48 (-1.05, 0.10) | -0.54 (-1.17, 0.09) | Significant improvement in both groups, no statistical differences | ⨁◯◯◯ Very low |
| 6.77 | | SNAGs vs. HVLA | | Pain | 4 weeks | 1 RCT  (Izquierdo Perez et al., 2014) | serious^d^ | NA | not serious | very serious^b,c^ | none | 21 | 19 | 0.12 (-0.50, 0.74) | 0.20 (-0.80, 1.20) | Significant improvement in both groups, no statistical differences | ⨁◯◯◯ Very low |
| 6.78 | | SNAGs vs. mobilization (PAIVMs) | | Pain | 4 weeks | 1 RCT  (Izquierdo Perez et al., 2014) | serious^d^ | NA | not serious | very serious^b,c^ | none | 21 | 21 | 0.32 (-0.29, 0.93) | 0.50 (-0.44, 1.44) | Significant improvement in both groups, no statistical differences | ⨁◯◯◯ Very low |
| 6.79 | | Self-SNAGs + CT vs. CT | | Pain | 4 weeks | 1 RCT  (Said et al., 2017) | very serious^a^ | NA | not serious | very serious^b^ | none | 29 | 29 | -1.90 (-2.53, -1.27) | -1.90 (-2.41, -1.39) | Self-SNAGs + CT | ⨁◯◯◯ Very low |
| 6.80 | | SNAGs + CT vs. CT | | Pain | 4 weeks | 1 RCT  (Said et al., 2017) | very serious^a^ | NA | not serious | very serious^b^ | none | 29 | 29 | -1.90 (-2.53, -1.27) | -1.90 (-2.41, -1.39) | SNAGs + CT | ⨁◯◯◯ Very low |
| 6.81 | | SNAG+NAG+self-SNAG x Sham | | Pain | 4 weeks | 1 RCT  (Zemadanis, 2018) | very serious^a^ | NA | not serious | very serious^b^ | none | 20 | 20 | -2.85 (-3.76, -1.95) | -2.40 (-2.91, -1.89) | SNAG+NAG+self-SNAG | ⨁◯◯◯ Very low |
| 6.82 | | SNAGs vs. SNAGs + exercise | | Pain | 6 weeks | 1 RCT  (Ali et al., 2014) | very serious^a^ | NA | not serious | very serious^b^ | none | 51 | 51 | NR | NR | SNAGs + ex | ⨁◯◯◯ Very low |
| 6.83 | | Self-SNAGs + exercise/CCFT vs. Cervicothoracic mobilization + exercise/CCFT | | Pain | 6 weeks | 1 RCT  (Sun et al., 2024) | serious^k^ | NA | not serious | very serious^b^ | none | 10 | 10 | 1.18 (0.21, 2.15) | 0.90 (0.26, 1.54) | Cervicothoracic mobilization + exercise/CCFT | ⨁◯◯◯ Very low |
| 6.84 | | Self-SNAGs + exercise/CCFT vs. exercise/CCFT | | Pain | 6 weeks | 1 RCT  (Sun et al., 2024) | serious^k^ | NA | not serious | very serious^b^ | none | 10 | 10 | -0.15 (-1.03, 0.73) | -0.10 (-0.67, 0.47) | Significant improvement in both groups, no statistical differences | ⨁◯◯◯ Very low |
| 6.85 | | SNAG+CT+ex vs PRT+CT+ex | | Pain | 8 weeks | 1 RCT  (Mohamed & Elrazik, 2020) | very serious^a^ | NA | not serious | very serious^b^ | none | 40 | 40 | -1.53 (-2.04, -1.03) | -1.03 (-1.32, -0.74) | SNAG+CT+ex | ⨁◯◯◯ Very low |
| 6.86 | | SNAGs vs. MFR | | Pain | 8 weeks | 1 RCT  (Morsi et al., 2023) | serious^k^ | NA | not serious | very serious^b^ | none | 18 | 19 | 0.03 (-0.62, 0.67) | 0.04 (-0.94,1.02) | Significant improvement in both groups, no statistical differences | ⨁◯◯◯ Very low |
| 6.87 | | SNAGs vs. SNAGs + MFR | | Pain | 8 weeks | 1 RCT  (Morsi et al., 2023) | serious^k^ | NA | not serious | very serious^b^ | none | 18 | 17 | 0.70 (0.02, 1.39) | 1.05 (0.08, 2.02) | SNAG + MFR | ⨁◯◯◯ Very low |
| 6.88 | | SNAG + MFR vs MFR | | Pain | 8 weeks | 1 RCT  (Morsi et al., 2023) | serious^k^ | NA | not serious | very serious^b^ | none | 17 | 19 | -0.63 (-1.31, 0.04) | 4.58 (3.79, 5.37) | SNAG + MFR | ⨁◯◯◯ Very low |
| 6.89 | | SNAGs vs. HVLA | | Pain | 8 weeks | 1 RCT  (Izquierdo Perez et al., 2014) | serious^d^ | NA | not serious | very serious^b,c^ | none | 18 | 18 | 0.23 (-0.43, 0.88) | 0.40 (-0.73, 1.53) | Significant improvement in both groups, no statistical differences | ⨁◯◯◯ Very low |
| 6.90 | | SNAGs vs. mobilization (PAIVMs) | | Pain | 8 weeks | 1 RCT  (Izquierdo Perez et al., 2014) | serious^d^ | NA | not serious | very serious^b,c^ | none | 18 | 19 | 0.37 (-0.28, 1.03) | 0.60 (-0.36, 1.56) | Significant improvement in both groups, no statistical differences | ⨁◯◯◯ Very low |
| 6.91 | | SNAGs vs. HVLA | | Pain | 12 weeks | 1 RCT  (Izquierdo Perez et al., 2014) | serious^d^ | NA | not serious | very serious^b,c^ | none | 16 | 17 | 0.11 (-0.57, 0.79) | 0.20 (-1.03, 1.43) | Significant improvement in both groups, no statistical differences | ⨁◯◯◯ Very low |
| 6.92 | | SNAGs vs. mobilization (PAIVMs) | | Pain | 12 weeks | 1 RCT  (Izquierdo Perez et al., 2014) | serious^d^ | NA | not serious | very serious^b,c^ | none | 16 | 18 | 0.38 (-0.30, 1.06) | 0.60 (-8.72, 9.92) | Significant improvement in both groups, no statistical differences | ⨁◯◯◯ Very low |
| Range of Motion | | | | | | | | | | | | | | | | | |
| 6.93 | | NAGs vs. placebo | | Cervical range of motion | Unclear | 1 RCT  (Kumar et al., 2011) | very serious^a^ | NA | not serious | very serious^b^ | none | 75 | 25 | NR | NR | Significant improvement in the NAG group, expect for the lateral flexion right | ⨁◯◯◯ Very low |
| 6.94 | | SNAGs vs. HVLA | | Flexion | Right after treatment | 1 RCT^j^  (Izquierdo Perez et al., 2014; Lopez-Lopez et al., 2015) | serious^d^ | NA | not serious | very serious^b,c^ | none | 38 | 34 | 1.02 (-1.37, 3.42) | 8.31 (-11.83, 28.45) | Significant improvement in both groups, no statistical differences. Large ES favouring HVLA | ⨁◯◯◯ Very low |
| 6.95 | | SNAGs vs. mobilization (PAIVMs) | | Flexion | Right after treatment | 1 RCT^j^  (Izquierdo Perez et al., 2014; Lopez-Lopez et al., 2015) | serious^d^ | NA | not serious | very serious^b^ | none | 38 | 37 | -0.37 (-0.83, 0.09) | -2.72 (-6.80, 1.35) | Significant improvement in both groups, no statistical differences | ⨁◯◯◯ Very low |
| 6.96 | | SNAGs + CT + exercises vs. CT + exercises | | Flexion | 2 weeks | 2 RCTs  (Buyukturan et al., 2018; Shamsi et al., 2021) | Serious^i^ | very serious^f^ | not serious | very serious^b^ | none | 71 | 69 | 1.62 (-0.01, 3.25) | 6.69 (0.73, 12.64) | SNAGs + CT + exercises | ⨁◯◯◯ Very low |
| 6.97 | | SNAGs + NAGs vs. MET | | Flexion | 3 weeks | 1 RCT  (Manzoor et al., 2021) | very serious^a^ | NA | not serious | very serious^b,c^ | none | 28 | 28 | 2.30 (1.61, 2.99) | 14.36 (11.41, 17.58) | SNAGs + NAGs | ⨁◯◯◯ Very low |
| 6.98 | | MWM for the scapula vs. CT + taping | | Flexion | 2 weeks | 1 RCT  (Alshami & AlSadiq, 2021) | very serious^a^ | NA | not serious | very serious^b^ | none | 20 | 20 | 0.54 (-0.09, 1.17) | 3.40 (-0.44, 7.24) | Significant improvement in both groups, no statistical differences | ⨁◯◯◯ Very low |
| 6.99 | | MWM for the scapula vs. CT + taping | | Flexion | 3 weeks | 1 RCT  (Alshami & AlSadiq, 2021) | very serious^a^ | NA | not serious | very serious^b^ | none | 20 | 20 | -0.16 (-0.78, 0.46) | -1.10 (-5.29, 3.09) | Significant improvement in both groups, no statistical differences | ⨁◯◯◯ Very low |
| 6.100 | | SNAGs + exercises vs. exercises | | Flexion | 2-4 weeks | 2 RCTs  (Duymaz & Yagci, 2018; Rezkallah & Abdullah, 2018) | very serious^a^ | Not serious | not serious | very serious^b,c^ | none | 45 | 42 | 1.73 (0.59, 2.87) | 5.93 (4.64, 7.22) | SNAG+ex | ⨁◯◯◯ Very low |
| 6.101 | | SNAGs + exercises vs. MFR + exercises | | Flexion | 4 weeks | 1 RCT  (Rezkallah & Abdullah, 2018) | very serious^a^ | NA | not serious | very serious^b^ | none | 25 | 23 | 0.54 (-0.03, 1.12) | -1.80 (-0.07, 3.67) | Significant improvement in both groups, no statistical differences | ⨁◯◯◯ Very low |
| 6.102 | | SNAGs vs. HVLA | | Flexion | 4 weeks | 1 RCT  (Izquierdo Perez et al., 2014) | serious^d^ | NA | not serious | very serious^b,c^ | none | 21 | 19 | 0.25 (-0.38, 0.87) | 3.20 (-4.56, 10.96) | Significant improvement in both groups, no statistical differences | ⨁◯◯◯ Very low |
| 6.103 | | SNAGs vs. mobilization (PAIVMs) | | Flexion | 4 weeks | 1 RCT  (Izquierdo Perez et al., 2014) | serious^d^ | NA | not serious | very serious^b,c^ | none | 21 | 21 | -0.18 (-0.78, 0.43) | -2.50 (-0.91, 5.91) | Significant improvement in both groups, no statistical differences | ⨁◯◯◯ Very low |
| 6.104 | | Self-SNAGs + exercise/CCFT vs. Cervicothoracic mobilization + exercise/CCFT | | Flexion | 6 weeks | 1 RCT  (Sun et al., 2024) | serious^k^ | NA | not serious | very serious^b^ | none | 10 | 10 | -0.61 (-1.51, 0.30) | -2.94 (-7.01, 1.13) | Significant improvement in both groups, no statistical differences | ⨁◯◯◯ Very low |
| 6.105 | | Self-SNAGs + exercise/CCFT vs. exercise/CCFT | | Flexion | 6 weeks | 1 RCT  (Sun et al., 2024) | serious^k^ | NA | not serious | very serious^b^ | none | 10 | 10 | 0.03 (-0.84, 0.91) | 0.20 (-5.02, 5.42) | Significant improvement in both groups, no statistical differences | ⨁◯◯◯ Very low |
| 6.106 | | SNAGs vs. MFR | | Flexion | 8 weeks | 1 RCT  (Morsi et al., 2023) | serious^k^ | NA | not serious | very serious^b^ | none | 18 | 19 | 0.03 (-0.61, 0.68) | 0.08 (-1.42, 1.58) | Significant improvement in both groups, no statistical differences | ⨁◯◯◯ Very low |
| 6.107 | | SNAGs vs. SNAGs + MFR | | Flexion | 8 weeks | 1 RCT  (Morsi et al., 2023) | serious^k^ | NA | not serious | very serious^b^ | none | 18 | 17 | -0.03 (-0.69, 0.63) | -0.09 (-2.00, 1.82) | Significant improvement in both groups, no statistical differences | ⨁◯◯◯ Very low |
| 6.108 | | SNAG + MFR vs. MFR | | Flexion | 8 weeks | 1 RCT  (Morsi et al., 2023) | serious^k^ | NA | not serious | very serious^b^ | none | 17 | 19 | 0.06 (-0.59, 0.72) | 0.17 (-1.61, 1.95) | Significant improvement in both groups, no statistical differences | ⨁◯◯◯ Very low |
| 6.109 | | SNAG+CT+ex vs. PRT+CT+ex | | Flexion | 8 weeks | 1 RCT  (Mohamed & Elrazik, 2020) | very serious^a^ | NA | not serious | very serious^b^ | none | 40 | 40 | 4.20 (3.40, 5.00) | 3.12 (2.80, 3.44) | SNAG+CT+ex | ⨁◯◯◯ Very low |
| 6.110 | | SNAGs vs. HVLA | | Flexion | 8 weeks | 1 RCT  (Izquierdo Perez et al., 2014) | serious^d^ | NA | not serious | very serious^b,c^ | none | 18 | 18 | -0.07 (-0.72,0.59) | -1.10 (-11.42, 9.22) | Significant improvement in both groups, no statistical differences | ⨁◯◯◯ Very low |
| 6.111 | | SNAGs vs. mobilization (PAIVMs) | | Flexion | 8 weeks | 1 RCT  (Izquierdo Perez et al., 2014) | serious^d^ | NA | not serious | very serious^b,c^ | none | 16 | 18 | -0.24 (-0.89, 0.41) | -3.70 (-13.42, 6.02) | Significant improvement in both groups, no statistical differences | ⨁◯◯◯ Very low |
| 6.112 | | SNAGs + exercises vs. Exercises | | Flexion | 12 weeks | 1 RCT  (Duymaz & Yagci, 2018) | very serious^a^ | NA | not serious | very serious^b^ | none | 20 | 20 | -3.02 (-3.95, -2.09) | -19.80 (-23.70, -15.82) | SNAG+ex | ⨁◯◯◯ Very low |
| 6.113 | | SNAGs vs. HVLA | | Flexion | 12 weeks | 1 RCT  (Izquierdo Perez et al., 2014) | serious^e^ | NA | not serious | very serious^b,c^ | none | 16 | 17 | 0.02 (-0.66, 0.70) | 0.30 (-9.19, 9.79) | Significant improvement in both groups, no statistical differences | ⨁◯◯◯ Very low |
| 6.114 | | SNAGs vs. mobilization (PAIVMs) | | Flexion | 12 weeks | 1 RCT  (Izquierdo Perez et al., 2014) | serious^e^ | NA | not serious | very serious^b^ | none | 16 | 18 | -0.17 (-0.85, 0.50) | -2.50 (-12, 7) | Significant improvement in both groups, no statistical differences | ⨁◯◯◯ Very low |
| 6.115 | | SNAGs vs. HVLA | | Extension | Right after treatment | 1 RCT^j^  (Izquierdo Perez et al., 2014; Lopez-Lopez et al., 2015) | serious^d^ | NA | not serious | very serious^b,c^ | none | 38 | 34 | 0.90 (-1.73, 3.54) | 6.62 (-16.94, 30.19) | Significant improvement in both groups. Large ES favouring SNAGs | ⨁◯◯◯ Very low |
| 6.116 | | SNAGs vs. mobilization (PAIVMs) | | Extension | Right after treatment | 1 RCT^j^  (Izquierdo Perez et al., 2014; Lopez-Lopez et al., 2015) | serious^d^ | NA | not serious | very serious^b^ | none | 38 | 37 | -0.14 (-0.59, 0.32) | -1.21 (-5.23, 2.81) | Significant improvement in both groups, no statistical differences | ⨁◯◯◯ Very low |
| 6.117 | | SNAGs + CT + exercises vs. CT + exercises | | Extension | 2 weeks | 2 RCTs  (Buyukturan et al., 2018; Shamsi et al., 2021) | Serious^i^ | very serious^f^ | not serious | very serious^b^ | none | 71 | 69 | 1.43 (-0.71, 3.56) | -5.98 (-1.44, 13.40) | Significant improvement in both groups, no statistical differences. Large ES favouring SNAGs+CT+exercise | ⨁◯◯◯ Very low |
| 6.118 | | MWM for the scapula vs. CT + taping | | Extension | 2 weeks | 1 RCT  (Alshami & AlSadiq, 2021) | very serious^a^ | NA | not serious | very serious^b^ | none | 20 | 20 | 0.26 (-0.36, 0.88) | 2.20 (-2.95, 7.35) | Significant improvement in both groups, no statistical differences | ⨁◯◯◯ Very low |
| 6.119 | | SNAGs + exercises vs. exercises | | Extension | 2-4 weeks | 2 RCTs  (Duymaz & Yagci, 2018; Rezkallah & Abdullah, 2018) | very serious^a^ | very serious^f^ | not serious | very serious^b,c^ | none | 45 | 42 | 2.79 (-0.66, 6.24) | 8.87 (-0.76, 18.49) | Significant improvement in both groups, no statistical differences. Large ES favouring SNAGs +exercises | ⨁◯◯◯ Very low |
| 6.120 | | SNAGs + NAGs vs. MET | | Extension | 3 weeks | 1 RCT  (Manzoor et al., 2021) | very serious^a^ | NA | not serious | very serious^b,c^ | none | 28 | 28 | 0.95 (0.39, 1.50) | 5.75 (2.61, 8.89) | SNAGs + NAGs | ⨁◯◯◯ Very low |
| 6.121 | | MWM for the scapula vs. CT + taping | | Extension | 3 weeks | 1 RCT  (Alshami & AlSadiq, 2021) | very serious^a^ | NA | not serious | very serious^b^ | none | 20 | 20 | 0.13 (-0.49, 0.75) | 1.10 (-4.16, 6.36) | Significant improvement in both groups, no statistical differences | ⨁◯◯◯ Very low |
| 6.122 | | SNAGs + exercises vs. MFR + exercises | | Extension | 4 weeks | 1 RCT  (Rezkallah & Abdullah, 2018) | very serious^a^ | NA | not serious | very serious^b^ | none | 25 | 23 | 0.46 (-0.11, 1.04) | 1.48 (-0.34, 3.30) | Significant improvement in both groups, no statistical differences | ⨁◯◯◯ Very low |
| 6.123 | | SNAGs vs HVLA | | Extension | 4 weeks | 1 RCT  (Izquierdo Perez et al., 2014) | serious^d^ | NA | not serious | very serious^b,c^ | none | 21 | 19 | -0.12 (-0.74, 0.51) | -1.60 (-10.26, 7.06) | Significant improvement in both groups, no statistical differences | ⨁◯◯◯ Very low |
| 6.124 | | SNAGs vs. mobilization (PAIVMs) | | Extension | 4 weeks | 1 RCT  (Izquierdo Perez et al., 2014) | serious^d^ | NA | not serious | very serious^b,c^ | none | 21 | 21 | 0.09 (-0.51, 0.70) | 1.20 (-6.48, 8.88) | Significant improvement in both groups, no statistical differences | ⨁◯◯◯ Very low |
| 6.125 | | Self-SNAGs + exercise/CCFT vs. Cervicothoracic mobilization + exercise/CCFT | | Extension | 6 weeks | 1 RCT  (Sun et al., 2024) | serious^k^ | NA | not serious | very serious^b^ | none | 10 | 10 | 0.11 (-0.77, 0.98) | 0.48 (-3.29, 4.25) | Significant improvement in both groups, no statistical differences | ⨁◯◯◯ Very low |
| 6.126 | | Self-SNAGs + exercise/CCFT vs. exercise/CCFT | | Extension | 6 weeks | 1 RCT  (Sun et al., 2024) | serious^k^ | NA | not serious | very serious^b^ | none | 10 | 10 | 1.40 (0.40, 2.40) | 8.05 (3.22, 12.88) | Self-SNAGs + exercise/CCFT | ⨁◯◯◯ Very low |
| 6.127 | | SNAGs vs. MFR | | Extension | 8 weeks | 1 RCT  (Morsi et al., 2023) | serious^k^ | NA | not serious | very serious^b^ | none | 18 | 19 | 0.49 (-0.17, 1.14) | 1.20 (-0.36, 2.76) | Significant improvement in both groups, no statistical differences | ⨁◯◯◯ Very low |
| 6.128 | | SNAGs vs. SNAGs + MFR | | Extension | 8 weeks | 1 RCT  (Morsi et al., 2023) | serious^k^ | NA | not serious | very serious^b^ | none | 18 | 17 | 0.36 (-0.31, 1.03) | 0.95 (-0.77, 2.67) | Significant improvement in both groups, no statistical differences | ⨁◯◯◯ Very low |
| 6.129 | | SNAG + MFR vs MFR | | Extension | 8 weeks | 1 RCT  (Morsi et al., 2023) | serious^k^ | NA | not serious | very serious^b^ | none | 17 | 19 | 0.10 (-0.55, 0.76) | 0.25 (-1.31, 1.81) | Significant improvement in both groups, no statistical differences | ⨁◯◯◯ Very low |
| 6.130 | | SNAG+CT+ex vs PRT+CT+ex | | Extension | 8 weeks | 1 RCT  (Mohamed & Elrazik, 2020) | very serious^a^ | NA | not serious | very serious^b^ | none | 40 | 40 | 5.61 (4.62, 6.60) | 6.50 (6.00, 7.00) | SNAG+CT+ex | ⨁◯◯◯ Very low |
| 6.131 | | SNAGs vs HVLA | | Extension | 8 weeks | 1 RCT  (Izquierdo Perez et al., 2014) | serious^d^ | NA | not serious | very serious^b,c^ | none | 18 | 18 | -0.03 (-0.68, 0.63) | -0.40 (-9.90, 9.10) | Significant improvement in both groups, no statistical differences | ⨁◯◯◯ Very low |
| 6.132 | | SNAGs vs. mobilization (PAIVMs) | | Extension | 8 weeks | 1 RCT  (Izquierdo Perez et al., 2014) | serious^d^ | NA | not serious | very serious^b,c^ | none | 18 | 19 | 0.22 (-0.42, 0.87) | 3.20 (-5.78, 12.18) | Significant improvement in both groups, no statistical differences | ⨁◯◯◯ Very low |
| 6.133 | | SNAGs + exercises vs. Exercises | | Extension | 12 weeks | 1 RCT  (Duymaz & Yagci, 2018) | very serious^a^ | NA | not serious | very serious^b^ | none | 20 | 20 | -1.17 (-1.85, -0.50) | -12.30 (-18.66, -5.94) | SNAGs + ex | ⨁◯◯◯ Very low |
| 6.134 | | SNAGs vs. HVLA | | Extension | 12 weeks | 1 RCT  (Izquierdo Perez et al., 2014) | serious^e^ | NA | not serious | very serious^b,c^ | none | 16 | 17 | -0.17 (-0.86, 0.51) | -2.70 (-13.03, 7.63) | Significant improvement in both groups, no statistical differences | ⨁◯◯◯ Very low |
| 6.135 | | SNAGs vs. mobilization (PAIVMs) | | Extension | 12 weeks | 1 RCT  (Izquierdo Perez et al., 2014) | Serious^e^ | NA | not serious | very serious^b^ | none | 16 | 18 | -0.03 (-0.70, 0.64) | -0.40 (-9.10, 8.30) | Significant improvement in both groups, no statistical differences | ⨁◯◯◯ Very low |
| 6.136 | | SNAGs vs. HVLA | | Lateral flexion | Right after treatment | 1 RCT^j^  (Izquierdo Perez et al., 2014; Lopez-Lopez et al., 2015) | serious^d^ | NA | not serious | very serious^b,c^ | none | 38 | 34 | 0.11 (-0.35, 0.58) | 0.58 (-0.65, 1.81) | Significant improvement in both groups, no statistical differences | ⨁◯◯◯ Very low |
| 6.137 | | SNAGs vs. mobilization (PAIVMs) | | Lateral flexion | Right after treatment | 1 RCT^j^  (Izquierdo Perez et al., 2014; Lopez-Lopez et al., 2015) | serious^d^ | NA | not serious | very serious^b^ | none | 38 | 37 | -0.95 (-2.20, 0.31) | -2.60 (-3.66, -1.55) | SNAGs | ⨁◯◯◯ Very low |
| 6.138 | | SNAGs + NAGs vs. MET | | Lateral-flexion | 3 weeks | 1 RCT  (Manzoor et al., 2021) | very serious^a^ | NA | not serious | very serious^b,c^ | none | 28 | 28 | 0.31 (-0.21, 0.84) | 2.39 (-1.54, 6.32) | Significant improvement in both groups, no statistical differences | ⨁◯◯◯ Very low |
| 6.139 | | SNAGs vs. HVLA | | Lateral-flexion | 4 weeks | 1 RCT  (Izquierdo Perez et al., 2014) | serious^d^ | NA | not serious | very serious^b,c^ | none | 21 | 19 | -0.01 (-0.63, 0.61) | -0.20 (-11.29, 10.89) | Significant improvement in both groups, no statistical differences | ⨁◯◯◯ Very low |
| 6.140 | | SNAGs vs. mobilization (PAIVMs) | | Lateral-flexion | 4 weeks | 1 RCT  (Izquierdo Perez et al., 2014) | serious^d^ | NA | not serious | very serious^b,c^ | none | 21 | 21 | -0.01 (-0.62, 0.59) | -0.20 (-9.07, 8.67) | Significant improvement in both groups, no statistical differences | ⨁◯◯◯ Very low |
| 6.141 | | SNAGs vs. HVLA | | Lateral-flexion | 8 weeks | 1 RCT  (Izquierdo Perez et al., 2014) | serious^d^ | NA | not serious | very serious^b,c^ | none | 18 | 18 | 0.33 (-0.33, 0.99) | 6.10 (-5.76, 17.95) | Significant improvement in both groups, no statistical differences | ⨁◯◯◯ Very low |
| 6.142 | | SNAGs vs. mobilization (PAIVMs) | | Lateral-flexion | 8 weeks | 1 RCT  (Izquierdo Perez et al., 2014) | serious^d^ | NA | not serious | very serious^b,c^ | none | 18 | 19 | 0.09 (-0.56, 0.73) | 1.60 (-9.93, 13.13) | Significant improvement in both groups, no statistical differences |  |
| 6.143 | | SNAGs + exercises vs. exercises | | Lateral flexion | 12 weeks | 1 RCT  (Duymaz & Yagci, 2018) | very serious^a^ | NA | not serious | very serious^b^ | none | 20 | 20 | 2.37 (1.55, 3.20) | 9.15 (6.81, 11.49) | SNAGs | ⨁◯◯◯ Very low |
| 6.144 | | SNAGs vs. HVLA | | Lateral flexion | 12 weeks | 1 RCT  (Izquierdo Perez et al., 2014) | serious^e^ | NA | not serious | very serious^b,c^ | none | 16 | 17 | 0.03 (-0.65, 0.71) | 0.60 (-12.7, 13.97) | Significant improvement in both groups, no statistical differences | ⨁◯◯◯ Very low |
| 6.145 | | SNAGs vs. mobilization (PAIVMs) | | Lateral flexion | 12 weeks | 1 RCT  (Izquierdo Perez et al., 2014) | Serious^e^ | NA | not serious | very serious^b^ | none | 16 | 18 | -0.19 (-0.86, 0.49) | -3.30 (-14.35, 7.93) | Significant improvement in both groups, no statistical differences | ⨁◯◯◯ Very low |
| 6.146 | | SNAGs + CT + exercises vs. CT + exercises | | Left lateral flexion | 2 weeks | 2 RCTs  (Buyukturan et al., 2018; Shamsi et al., 2021) | Serious^i^ | not serious | not serious | very serious^b^ | none | 71 | 69 | 1.09 (0.73, 1.45) | 4.00 (2.81, 5.18) | SNAGs + CT + ex | ⨁◯◯◯ Very low |
| 6.147 | | MWM for the scapula vs. CT + taping | | Left lateral flexion | 2 weeks | 1 RCT  (Alshami & AlSadiq, 2021) | very serious^a^ | NA | not serious | very serious^b^ | none | 20 | 20 | 0.27 (-0.35, 0.90) | 2.50 (-3.04, 8.04) | Significant improvement in both groups, no statistical differences | ⨁◯◯◯ Very low |
| 6.148 | | MWM for the scapula vs. CT + taping | | Left lateral flexion | 3 weeks | 1 RCT  (Alshami & AlSadiq, 2021) | very serious^a^ | NA | not serious | very serious^b^ | none | 20 | 20 | -0.30 (-0.92, 0.32) | -2.40 (-7.24, 2.44) | Significant improvement in both groups, no statistical differences | ⨁◯◯◯ Very low |
| 6.149 | | SNAGs + exercises vs. exercises | | Left lateral flexion | 4 weeks | 1 RCT  (Rezkallah & Abdullah, 2018) | very serious^a^ | NA | not serious | very serious^b,c^ | none | 23 | 25 | 3.94 (2.93, 4.95) | 5.69 (4.86, 6.52) | SNAGs + ex | ⨁◯◯◯ Very low |
| 6.150 | | SNAGs + exercises vs. MFR + exercises | | Left lateral flexion | 4 weeks | 1 RCT  (Rezkallah & Abdullah, 2018) | very serious^a^ | NA | not serious | very serious^b^ | none | 25 | 23 | 0.85 (0.26, 1.44) | 1.57 (0.52, 2.62) | SNAGs + ex | ⨁◯◯◯ Very low |
| 6.151 | | Self-SNAGs + exercise/CCFT vs. Cervicothoracic mobilization + exercise/CCFT | | Left lateral flexion | 6 weeks | 1 RCT  (Sun et al., 2024) | serious^k^ | NA | not serious | very serious^b^ | none | 10 | 10 | -0.71 (-1.62, 0.20) | -2.25 (-4.92, 0.42) | Significant improvement in both groups, no statistical differences | ⨁◯◯◯ Very low |
| 6.152 | | Self-SNAGs + exercise/CCFT vs. exercise/CCFT | | Left lateral flexion | 6 weeks | 1 RCT  (Sun et al., 2024) | serious^k^ | NA | not serious | very serious^b^ | none | 10 | 10 | 0.50 (-0.39, 1.40) | 2.18 (-1.45, 5.81) | Significant improvement in both groups, no statistical differences | ⨁◯◯◯ Very low |
| 6.153 | | SNAGs vs. MFR | | Left lateral flexion | 8 weeks | 1 RCT  (Morsi et al., 2023) | serious^k^ | NA | not serious | very serious^b^ | none | 18 | 19 | -0.45 (-1.11, 0.20) | -1.28 (-3.05, 0.49) | Significant improvement in both groups, no statistical differences | ⨁◯◯◯ Very low |
| 6.154 | | SNAGs vs. SNAGs + MFR | | Left lateral flexion | 8 weeks | 1 RCT  (Morsi et al., 2023) | serious^k^ | NA | not serious | very serious^b^ | none | 18 | 17 | -2.31 (-2.87, -1.74) | -3.63 (-4.77, -2.49) | SNAGs + MFR | ⨁◯◯◯ Very low |
| 6.155 | | SNAG + MRF vs MFR | | Left lateral flexion | 8 weeks | 1 RCT  (Morsi et al., 2023) | serious^k^ | NA | not serious | very serious^b^ | none | 17 | 19 | 0.83 (0.15, 1.52) | 2.06 (0.53, 3.59) | SNAGs + MFR | ⨁◯◯◯ Very low |
| 6.156 | | SNAG+CT+ex vs PRT+CT+ex | | Left lateral flexion | 8 weeks | 1 RCT  (Mohamed & Elrazik, 2020) | very serious^a^ | NA | not serious | very serious^b^ | none | 40 | 40 | 4.98 (4.08, 5.89) | 5.80 (5.29, 6.31) | SNAG+CT+ex | ⨁◯◯◯ Very low |
| 6.157 | | SNAGs + CT + exercises vs. CT + exercises | | Right lateral flexion | 2 weeks | 2 RCTs  (Buyukturan et al., 2018; Shamsi et al., 2021) | Serious^i^ | serious^g^ | not serious | very serious^b^ | none | 71 | 69 | 0.90 (0.55, 1.25) | 3.91 (1.10, 6.73) | SNAGs + CT + ex | ⨁◯◯◯ Very low |
| 6.158 | | MWM for the scapula vs. CT + taping | | Right lateral flexion | 2 weeks | 1 RCT  **(Alshami & AlSadiq, 2021)** | very serious^a^ | NA | not serious | very serious^b^ | none | 20 | 20 | 0.23 (-0.39, 0.85) | 2.00 (-3.25, 7.25) | Significant improvement in both groups, no statistical differences | ⨁◯◯◯ Very low |
| 6.159 | | MWM for the scapula vs. CT + taping | | Right lateral flexion | 3 weeks | 1 RCT  (Alshami & AlSadiq, 2021) | very serious^a^ | NA | not serious | very serious^b^ | none | 20 | 20 | -0.10 (-0.72, 0.52) | -0.70 (-4.93, 3.53) | Significant improvement in both groups, no statistical differences | ⨁◯◯◯ Very low |
| 6.160 | | SNAGs + exercises vs. exercises | | Right lateral flexion | 4 weeks | 1 RCT  (Rezkallah & Abdullah, 2018) | very serious^a^ | NA | not serious | very serious^b,c^ | none | 23 | 25 | 2.69 (1.88, 3.49) | 3.52 (2.76, 4.28) | SNAGs + ex | ⨁◯◯◯ Very low |
| 6.161 | | SNAGs + exercises vs. MFR + exercises | | Right lateral flexion | 4 weeks | 1 RCT  (Rezkallah & Abdullah, 2018) | very serious^a^ | NA | not serious | very serious^b^ | none | 25 | 23 | 0.59 (0.01, 1.17) | 0.99 (0.02, 1.96) | SNAGs + ex | ⨁◯◯◯ Very low |
| 6.162 | | Self-SNAGs + exercise/CCFT vs. Cervicothoracic mobilization + exercise/CCFT | | Right lateral flexion | 6 weeks | 1 RCT  (Sun et al., 2024) | serious^k^ | NA | not serious | very serious^b^ | none | 10 | 10 | -1.09 (-2.05, -0.14) | -4.80 (-8.49, -1.11) | Cervicothoracic mobilization + exercise/CCFT | ⨁◯◯◯ Very low |
| 6.163 | | Self-SNAGs + exercise/CCFT vs. exercise/CCFT | | Right lateral flexion | 6 weeks | 1 RCT  (Sun et al., 2024) | serious^k^ | NA | not serious | very serious^b^ | none | 10 | 10 | 0.66 (-0.24, 1.57) | 2.74 (-0.73, 6.21) | Significant improvement in both groups, no statistical differences | ⨁◯◯◯ Very low |
| 6.164 | | SNAGs vs. MFR | | Right lateral flexion | 8 weeks | 1 RCT  (Morsi et al., 2023) | serious^k^ | NA | not serious | very serious^b^ | none | 18 | 19 | -0.55 (-1.20, 0.11) | -1.62 (-3.47, 0.23) | Significant improvement in both groups, no statistical differences | ⨁◯◯◯ Very low |
| 6.165 | | SNAGs vs. SNAGs + MFR | | Right lateral flexion | 8 weeks | 1 RCT  (Morsi et al., 2023) | serious^k^ | NA | not serious | very serious^b^ | none | 18 | 17 | -1.91 (-2.73, -1.10) | -4.30 (-5.75, -2.85) | SNAGs + MFR | ⨁◯◯◯ Very low |
| 6.166 | | SNAG + MRF vs MFR | | Right lateral flexion | 8 weeks | 1 RCT  (Morsi et al., 2023) | serious^k^ | NA | not serious | very serious^b^ | none | 17 | 19 | 0.92 (0.23, 1.61) | 2.68 (0.86, 4.50) | SNAGs + MFR | ⨁◯◯◯ Very low |
| 6.167 | | SNAG+CT+ex vs PRT+CT+ex | | Right lateral flexion | 8 weeks | 1 RCT  (Mohamed & Elrazik, 2020) | very serious^a^ | NA | not serious | very serious^b^ | none | 40 | 40 | 3.77 (3.03, 4.51) | 4.47 (3.96, 4.98) | SNAG+CT+ex | ⨁◯◯◯ Very low |
| 6.168 | | SNAGs vs. HVLA | | Rotation | Right after treatment | 1 RCT^j^  (Izquierdo Perez et al., 2014; Lopez-Lopez et al., 2015) | serious^d^ | NA | not serious | very serious^b,c^ | none | 38 | 34 | 0.73 (-1.18, 2.64) | 3.26 (-8.61, 15.13) | Significant improvement in both groups, no statistical differences | ⨁◯◯◯ Very low |
| 6.169 | | SNAGs vs. mobilization (PAIVMs) | | Rotation | Right after treatment | 1 RCT^j^  (Izquierdo Perez et al., 2014; Lopez-Lopez et al., 2015) | serious^d^ | NA | not serious | very serious^b^ | none | 38 | 37 | -0.07 (-0.52, 0.39) | 0.50 (-1.91, 2.90) | Significant improvement in both groups, no statistical differences | ⨁◯◯◯ Very low |
| 6.170 | | SNAGs + NAGs vs. MET | | Rotation | 3 weeks | 1 RCT  (Manzoor et al., 2021) | very serious^a^ | NA | not serious | very serious^b,c^ | none | 28 | 28 | 0.43 (-0.10, 0.96) | 6.50 (-1.31, 14.31) | Significant improvement in both groups, no statistical differences | ⨁◯◯◯ Very low |
| 6.171 | | SNAGs vs, HVLA | | Rotation | 4 weeks | 1 RCT  (Izquierdo Perez et al., 2014) | serious^d^ | NA | not serious | very serious^b,c^ | none | 21 | 19 | 0.06 (-0.56, 0.68) | 1.70 (-17.09, 20.49) | Significant improvement in both groups, no statistical differences | ⨁◯◯◯ Very low |
| 6.172 | | SNAGs vs, mobilization (PAIVMs) | | Rotation | 4 weeks | 1 RCT  (Izquierdo Perez et al., 2014) | serious^d^ | NA | not serious | very serious^b,c^ | none | 21 | 21 | -0.05 (-0.70, 0.61) | -2.00 (-18.07, 14.07) | Significant improvement in both groups, no statistical differences | ⨁◯◯◯ Very low |
| 6.173 | | SNAGs + exercises vs. exercises | | Rotation | 12 weeks | 1 RCT  (Duymaz & Yagci, 2018) | very serious^a^ | NA | not serious | very serious^b^ | none | 20 | 20 | -2.68 (-3.56, -1.81) | -11.80 (-14.47, -9.13) | SNAGs + ex | ⨁◯◯◯ Very low |
| 6.174 | | SNAGs vs. HVLA | | Rotation | 12 weeks | 1 RCT  (Izquierdo Perez et al., 2014) | serious^e^ | NA | not serious | very serious^b,c^ | none | 16 | 17 | 0.03 (-0.65, 0.71) | 0.90 (-18.46, 20.26) | Significant improvement in both groups, no statistical differences | ⨁◯◯◯ Very low |
| 6.175 | | SNAGs vs. mobilization (PAIVMs) | | Rotation | 12 weeks | 1 RCT  (Izquierdo Perez et al., 2014) | Serious^e^ | NA | not serious | very serious^b^ | none | 16 | 18 | -0.26 (-0.94, 0.41) | -6.20 (-21.53, -0.13) | SNAGs | ⨁◯◯◯ Very low |
| 6.176 | | SNAGs + CT + exercises vs. CT + exercises | | Left rotation | 2 weeks | 2 RCTs  (Buyukturan et al., 2018; Shamsi et al., 2021) | Serious^i^ | very serious^f^ | not serious | very serious^b^ | none | 71 | 69 | 1.84 (1.11, 2.57) | 9.64 (4.52, 14.76) | SNAGs + CT + exercises | ⨁◯◯◯ Very low |
| 6.177 | | MWM for the scapula vs. CT + taping | | Left rotation | 2 weeks | 1 RCT  (Alshami & AlSadiq, 2021) | very serious^a^ | NA | not serious | very serious^b^ | none | 20 | 20 | 0.43 (-0.20, 1.06) | 3.10 (-1.25, 7.45) | Significant improvement in both groups, no statistical differences | ⨁◯◯◯ Very low |
| 6.178 | | MWM for the scapula vs. CT + taping | | Left rotation | 3 weeks | 1 RCT  (Alshami & AlSadiq, 2021) | very serious^a^ | NA | not serious | very serious^b^ | none | 20 | 20 | 0.21 (-0.41, 0.83) | 1.90 (-3.57, 7.37) | Significant improvement in both groups, no statistical differences | ⨁◯◯◯ Very low |
| 6.179 | | SNAGs + exercises vs. exercises | | Left rotation | 4 weeks | 1 RCT  (Rezkallah & Abdullah, 2018) | very serious^a^ | NA | not serious | very serious^b^ | none | 23 | 25 | 1.60 (0.95, 2.26) | 3.94 (2.60, 5.28) | SNAGs + ex | ⨁◯◯◯ Very low |
| 6.180 | | SNAGs + exercises vs. MFR + exercises | | Left rotation | 4 weeks | 1 RCT  (Rezkallah & Abdullah, 2018) | very serious^a^ | NA | not serious | very serious^b^ | none | 25 | 23 | -0.23 (-0.80, 0.33) | -0.40 (-1.36, 0.56) | Significant improvement in both groups, no statistical differences | ⨁◯◯◯ Very low |
| 6.181 | | Self-SNAGs + exercise/CCFT vs. Cervicothoracic mobilization + exercise/CCFT | | Left rotation | 6 weeks | 1 RCT  (Sun et al., 2024) | serious^k^ | NA | not serious | very serious^b^ | none | 10 | 10 | -1.01 (-1.96, -0.07) | -5.31 (-9.71, -0.91) | Self-SNAGs + exercise/CCFT | ⨁◯◯◯ Very low |
| 6.182 | | Self-SNAGs + exercise/CCFT vs. exercise/CCFT | | Left rotation | 6 weeks | 1 RCT  (Sun et al., 2024) | serious^k^ | NA | not serious | very serious^b^ | none | 10 | 10 | 0.38 (-0.50, 1.27) | 2.16 (-2.59, 6.91) | Significant improvement in both groups, no statistical differences | ⨁◯◯◯ Very low |
| 6.183 | | SNAGs vs. MFR | | Left rotation | 8 weeks | 1 RCT  (Morsi et al., 2023) | serious^k^ | NA | not serious | very serious^b^ | none | 18 | 19 | 0.39 (-0.27, 1.04) | 1.09 (-0.66, 2.84) | Significant improvement in both groups, no statistical differences | ⨁◯◯◯ Very low |
| 6.184 | | SNAGs vs. SNAGs + MFR | | Left rotation | 8 weeks | 1 RCT  (Morsi et al., 2023) | serious^k^ | NA | not serious | very serious^b^ | none | 18 | 17 | -0.92 (-1.62, -0.22) | -1.99 (-3.41, -0.57) | SNAGs + MFR | ⨁◯◯◯ Very low |
| 6.185 | | SNAG + MRF vs. MFR | | Left rotation | 8 weeks | 1 RCT  (Morsi et al., 2023) | serious^k^ | NA | not serious | very serious^b^ | none | 17 | 19 | 1.01 (0.31, 1.71) | 3.08 (1.16, 5.00) | SNAGs + MFR | ⨁◯◯◯ Very low |
| 6.186 | | SNAG+CT+ex vs PRT+CT+ex | | Left rotation | 8 weeks | 1 RCT  (Mohamed & Elrazik, 2020) | very serious^a^ | NA | not serious | very serious^b^ | none | 40 | 40 | 5.83 (4.80, 6.85) | 7.20 (6.66, 7.74) | SNAG+CT+ex | ⨁◯◯◯ Very low |
| 6.187 | | SNAGs + CT + exercises vs. CT + exercises | | Right rotation | 2 weeks | 2 RCTs  (Buyukturan et al., 2018; Shamsi et al., 2021) | Serious^i^ | serious^g^ | not serious | very serious^b^ | none | 71 | 69 | 1.51 (1.13, 1.89) | 8.70 (6.02, 11.38) | SNAGs + CT + ex | ⨁◯◯◯ Very low |
| 6.188 | | MWM for the scapula vs. CT + taping | | Right rotation | 2 weeks | 1 RCT  (Alshami & AlSadiq, 2021) | very serious^a^ | NA | not serious | very serious^b^ | none | 20 | 20 | 0.09 (-0.53, 0.71) | 0.80 (-4.82, 6.42) | Significant improvement in both groups, no statistical differences | ⨁◯◯◯ Very low |
| 6.189 | | MWM for the scapula vs. CT + taping | | Right rotation | 3 weeks | 1 RCT  (Alshami & AlSadiq, 2021) | very serious^a^ | NA | not serious | very serious^b^ | none | 20 | 20 | 0.35 (-0.27, 0.98) | 2.50 (-1.84, 6.84) | Significant improvement in both groups, no statistical differences | ⨁◯◯◯ Very low |
| 6.190 | | SNAGs + exercises vs. exercises | | Right rotation | 4 weeks | 1 RCT  (Rezkallah & Abdullah, 2018) | very serious^a^ | NA | not serious | very serious^b^ | none | 23 | 25 | 3.40 (2.49, 4.31) | 4.98 (4.15, 5.81) | SNAGs + ex | ⨁◯◯◯ Very low |
| 6.191 | | SNAGs + exercises vs. MFR + exercises | | Right rotation | 4 weeks | 1 RCT  (Rezkallah & Abdullah, 2018) | very serious^a^ | NA | not serious | very serious^b^ | none | 25 | 23 | 0.41 (-0.17, 0.98) | 1.38 (-0.57, 3.33) | Significant improvement in both groups, no statistical differences | ⨁◯◯◯ Very low |
| 6.192 | | Self-SNAGs + exercise/CCFT vs. Cervicothoracic mobilization + exercise/CCFT | | Right rotation | 6 weeks | 1 RCT  (Sun et al., 2024) | serious^k^ | NA | not serious | very serious^b^ | none | 10 | 10 | -0.78 (-1.70, 0.14) | -4.22 (-8.76, 0.32) | Significant improvement in both groups, no statistical differences | ⨁◯◯◯ Very low |
| 6.193 | | Self-SNAGs + exercise/CCFT vs. exercise/CCFT | | Right rotation | 6 weeks | 1 RCT  (Sun et al., 2024) | serious^k^ | NA | not serious | very serious^b^ | none | 10 | 10 | 1.11 (0.15, 2.06) | 3.72 (0.90, 6.54) | Self-SNAGs + exercise/CCFT | ⨁◯◯◯ Very low |
| 6.194 | | SNAGs vs. MFR | | Right rotation | 8 weeks | 1 RCT  (Morsi et al., 2023) | serious^k^ | NA | not serious | very serious^b^ | none | 18 | 19 | 0.61 (-0.05, 1.27) | 2.51 (-0.07, 5.09) | Significant improvement in both groups, no statistical differences | ⨁◯◯◯ Very low |
| 6.195 | | SNAGs vs. SNAGs + MFR | | Right rotation | 8 weeks | 1 RCT  (Morsi et al., 2023) | serious^k^ | NA | not serious | very serious^b^ | none | 18 | 17 | -0.27 (-0.94, 0.39) | -0.81 (-2.73, 1.11) | Significant improvement in both groups, no statistical differences | ⨁◯◯◯ Very low |
| 6.196 | | SNAG + MRF vs. MFR | | Right rotation | 8 weeks | 1 RCT  (Morsi et al., 2023) | serious^k^ | NA | not serious | very serious^b^ | none | 17 | 19 | 0.81 (0.13, 1.49) | 3.31 (0.77, 5.85) | SNAG + MRF | ⨁◯◯◯ Very low |
| 6.197 | | SNAG+CT+ex vs PRT+CT+ex | | Right rotation | 8 weeks | 1 RCT  (Mohamed & Elrazik, 2020) | very serious^a^ | NA | not serious | very serious^b^ | none | 40 | 40 | 6.92 (5.74, 8.10) | 7.45 (6.98, 7.92) | SNAG+CT+ex | ⨁◯◯◯ Very low |
| Disability | | | |  |  |  |  |  |  |  |  |  |  |  |  |  |  |
| 6.198 | | SNAGs vs. HVLA | | Disability | Right after treatment | 1 RCT^j^  (Izquierdo Perez et al., 2014; Lopez-Lopez et al., 2015) | serious^d^ | NA | not serious | very serious^b,c^ | none | 38 | 34 | -0.49 (-0.14, 1.12) | 3.90 (-0.86, 8.66) | HVLA | ⨁◯◯◯ Very low |
| 6.199 | | SNAGs vs. mobilization (PAIVMs) | | Disability | Right after treatment | 1 RCT^j^  (Izquierdo Perez et al., 2014; Lopez-Lopez et al., 2015) | serious^d^ | NA | not serious | very serious^b^ | none | 38 | 37 | 0.25 (-0.36, 0.86) | 2.40 (-3.32, 8.12) | PAIVMs | ⨁◯◯◯ Very low |
| 6.200 | | SNAGs + CT + exercises vs. CT + exercises | | Disability | 2 weeks | 3 RCTs  (Buyukturan et al., 2018; Shamsi et al., 2021; Tachii et al., 2015) | very serious^h^ | not serious ^g^ | not serious | very serious^b^ | none | 86 | 84 | -0.68 (-0.99, -0.37) | -2.58 (-4.30, 0.86) | SNAGs + CT + ex | ⨁◯◯◯ Very low |
|  |  | SNAGs + CT + exercises vs. CT + exercises  ***sensitiviy analisys** | | Disability | 2/4 weeks weeks | 4 RCTs  (Abd El-Azeim & Grase, 2023; Buyukturan et al., 2018; Shamsi et al., 2021; Tachii et al., 2015) | very serious^h^ | not serious ^g^ | not serious | very serious^b^ | none | 131 | 129 | -1.35 (-2.63, -0.06) | -3.75 (-6.61, -0.88) | SNAGs + CT + ex | ⨁◯◯◯ Very low |
| 6.201 | | SNAGs + NAGs vs. MET | | Disability | 3 weeks | 1 RCT  (Manzoor et al., 2021) | very serious^a^ | NA | not serious | very serious^b,c^ | none | 28 | 28 | 1.59 (0.99, 2.20) | 13.39 (9.05, 17.73) | SNAGs + NAG | ⨁◯◯◯ Very low |
| 6.202 | | SNAG+NAG+self-SNAG x Sham | | Disability | 3 weeks | 1 RCT  (Zemadanis, 2018) | very serious^a^ | NA | not serious | very serious^b^ | none | 20 | 20 | -2.25 (-3.06, -1.44) | -10.20 (-12.95, -7.45) | SNAG+NAG+self-SNAG | ⨁◯◯◯ Very low |
| 6.203 | | SNAGs + exercises vs. exercises | | Disability | 2-4 weeks | 2 RCTs  (Duymaz & Yagci, 2018; Rezkallah & Abdullah, 2018) | very serious^a^ | very serious^f^ | not serious | very serious^b^ | none | 45 | 42 | -3.50 (-6.58, -0.43) | -8.65 (-9.57, -7.73) | SNAGs + ex | ⨁◯◯◯ Very low |
| 6.204 | | MWM for the scapula vs. CT + taping | | Disability | 2 weeks | 1 RCT  (Alshami & AlSadiq, 2021) | very serious^a^ | NA | not serious | very serious^b^ | none | 20 | 20 | -0.32 (-0.94, 0.31) | -3.00 (-8.76, 2,76) | Significant improvement in both groups, no statistical differences | ⨁◯◯◯ Very low |
| 6.205 | | MWM for the scapula vs. CT + taping | | Disability | 3 weeks | 1 RCT  (Alshami & AlSadiq, 2021) | very serious^a^ | NA | not serious | very serious^b^ | none | 20 | 20 | 0.10 (-0.52, 0.72) | 1.00 (-4.87, 6.87) | Significant improvement in both groups, no statistical differences | ⨁◯◯◯ Very low |
| 6.206 | | SNAGs + exercises vs. MFR + exercises | | Disability | 4 weeks | 1 RCT  (Rezkallah & Abdullah, 2018) | very serious^a^ | NA | not serious | very serious^b^ | none | 25 | 23 | -1.00 (-1.60, -0.39) | -2.10 (-3.31, -0.89) | SNAGs + ex | ⨁◯◯◯ Very low |
| 6.207 | | SNAGs vs. HVLA | | Disability | 4 week | 1 RCT^J^  (Izquierdo Perez et al., 2014) | serious^e^ | NA | not serious | very serious^b,c^ | none | 21 | 19 | 0.09 (-0.53, 0.71) | 0.70 (-4.05, 5.45) | Significant improvement in both groups, no statistical differences | ⨁◯◯◯ Very low |
| 6.208 | | SNAGs vs. mobilization (PAIVMs) | | Disability | 4 week | 1 RCT^J^  (Izquierdo Perez et al., 2014; Lopez-Lopez et al., 2015) | serious^e^ | NA | not serious | very serious^b,c^ | none | 21 | 21 | 0.04 (-0.56, 0.65) | 0.40 (-5.10, 5.90) | Significant improvement in both groups, no statistical differences | ⨁◯◯◯ Very low |
| 6.209 | | Self-SNAGs + CT vs. CT | | Disability | 4 weeks | 1 RCT  (Said et al., 2017) | very serious^a^ | NA | not serious | very serious^b^ | none | 29 | 29 | -4.46 (-5.44, -3.47) | -41.30 (-46.00, -36.60) | Self-SNAGs + CT | ⨁◯◯◯ Very low |
| 6.210 | | SNAGs + CT vs. CT | | Disability | 4 weeks | 1 RCT  (Said et al., 2017) | very serious^a^ | NA | not serious | very serious^b^ | none | 29 | 29 | -4.33 (-5.39, -3.36) | -40.10 (-44.81, -35.39) | SNAGs + CT | ⨁◯◯◯ Very low |
| 6.211 | | SNAG+NAG+self-SNAG x Sham | | Disability | 4 weeks | 1 RCT  (Zemadanis, 2018) | very serious^a^ | NA | not serious | very serious^b^ | none | 20 | 20 | -2.55 (-3.41, -1.70) | -10.55 (-13.06, -8.04) | SNAG+NAG+self-SNAG | ⨁◯◯◯ Very low |
| 6.212 | | SNAGs vs. SNAGs + exercise | | Disability | 6 weeks | 1 RCT  (Ali et al., 2014) | very serious^a^ | NA | not serious | very serious^b^ | none | 51 | 51 | NR | NR | SNAGs + ex | ⨁◯◯◯ Very low |
| 6.213 | | Self-SNAGs + exercise/CCFT vs. Cervicothoracic mobilization + exercise/CCFT | | Disability | 6 weeks | 1 RCT  (Sun et al., 2024) | serious^k^ | NA | not serious | very serious^b^ | none | 10 | 10 | 0.47 (-0.42, 1.36) | 0.03 (-0.02, 0.08) | Significant improvement in both groups, no statistical differences | ⨁◯◯◯ Very low |
| 6.214 | | Self-SNAGs + exercise/CCFT vs. exercise/CCFT | | Disability | 6 weeks | 1 RCT  (Sun et al., 2024) | serious^k^ | NA | not serious | very serious^b^ | none | 10 | 10 | -0.47 (-1.36, 0.42) | -0.03 (-0.08, 0.02) | Significant improvement in both groups, no statistical differences | ⨁◯◯◯ Very low |
| 6.215 | | SNAG+CT+ex vs PRT+CT+ex | | Disability | 8 weeks | 1 RCT  (Mohamed & Elrazik, 2020) | very serious^a^ | NA | not serious | very serious^b^ | none | 40 | 40 | -2.47 (-3.06, -1.88) | -2.57 (-3.02, -2.12) | SNAGs+CT+ex | ⨁◯◯◯ Very low |
| 6.216 | | SNAGs vs. MFR | | Disability | 8 weeks | 1 RCT  (Morsi et al., 2023) | serious^k^ | NA | not serious | very serious^b^ | none | 18 | 19 | -0.21 (-0.86, 0.44) | -0.47 (-1.90, 0.96) | Significant improvement in both groups, no statistical differences | ⨁◯◯◯ Very low |
| 6.217 | | SNAGs vs. SNAGs + MFR | | Disability | 8 weeks | 1 RCT  (Morsi et al., 2023) | serious^k^ | NA | not serious | very serious^b^ | none | 18 | 17 | 0.82 (0.13, 1.52) | 1.70 (0.38, 3.02) | SNAGs + MFR | ⨁◯◯◯ Very low |
| 6.218 | | SNAG + MFR vs MFR | | Disability | 8 weeks | 1 RCT  (Morsi et al., 2023) | serious^k^ | NA | not serious | very serious^b^ | none | 17 | 19 | -1.20 (-1.92, -0.48) | -2.71 (-3.31, -1.03) | SNAG + MRF | ⨁◯◯◯ Very low |
| 6.219 | | SNAGs vs. HVLA | | Disability | 8 weeks | 1 RCT  (Izquierdo Perez et al., 2014) | serious^d^ | NA | not serious | very serious^b,c^ | none | 18 | 18 | 0.49 (-0.14, 1.12) | 3.90 (-0.86, 8.66) | Significant improvement in both groups, no statistical differences | ⨁◯◯◯ Very low |
| 6.220 | | SNAGs vs. mobilization (PAIVMs) | | Disability | 8 weeks | 1 RCT  (Izquierdo Perez et al., 2014) | serious^d^ | NA | not serious | very serious^b,c^ | none | 18 | 19 | -0.05 (-0.69, 0.59) | -0.50 (-6.79, 5.79) | SNAGs Significant improvement in both groups, no statistical differences | ⨁◯◯◯ Very low |
| 6. 221 | | SNAGs vs. HVLA | | Disability | 12 weeks | 1 RCT^J^  (Izquierdo Perez et al., 2014) | serious^e^ | NA | not serious | very serious^b,c^ | none | 16 | 17 | -0.12 (-0.80, 0.57) | -1.00 (-6.78, 4.78) | Significant improvement in both groups, no statistical differences | ⨁◯◯◯ Very low |
| 6.222 | | SNAGs vs. mobilization (PAIVMs) | | Disability | 12 weeks | 1 RCT^J^  (Izquierdo Perez et al., 2014) | serious^e^ | NA | not serious | very serious^b^ | none | 16 | 18 | 0 (-0.67, 0.67) | 0 (-5.89, 5.89) | Significant improvement in both groups, no statistical differences | ⨁◯◯◯ Very low |
| Unclear (not specified) chronicity | | | | | | |  |  |  |  |  |  |  |  |  |  |  |
|  | **Pain intensity** | | | | | | | | | | | | | | | | |
| 6.223 | | SNAGs + CT vs. CT | | Pain | 2 weeks | 2 RCTs  (Aggarwal & Verma, 2018; Ozlu & Sahin, 2024) | very serious^a^ | not serious | not serious | very serious^b^ | none | 39 | 39 | -1.01 (-2.27, 0.25) | -1.76 (-4.07, 0.54) | SNAG + CT | ⨁◯◯◯ Very low |
| 6.224 | | SNAG + CT vs. NAG +CT | | Pain | 2 weeks | 1 RCT  (Waqas et al., 2017) | very serious^a^ | NA | not serious | very serious^b^ | none | 25 | 25 | -0.96 (-1.55, -0.37) | -0.64 (-1.00, -0.28) | SNAG + CT | ⨁◯◯◯ Very low |
| 6.225 | | Self-SNAG + CT vs. CT | | Pain | 2 weeks | 1 RCT  (Aggarwal & Verma, 2018) | very serious^a^ | NA | not serious | very serious^b^ | none | 19 | 19 | -0.38 (-1.02, 0.26) | -0.60 (-1.59, 0.39) | Significant improvement in both groups, no statistical differences | ⨁◯◯◯ Very low |
| 6.226 | | MET (post-isometric relaxation) vs. NAGs | | Pain | 4 weeks | 1 RCT  (Usama et al., 2022) | very serious^a^ | NA | not serious | very serious^b^ | none | 23 | 22 | -2.57 (-3.38, -1.76) | -1.57 (-1.92, -1.22) | NAGs | ⨁◯◯◯ Very low |
| 6.227 | | SNAGs + CT vs. CT | | Pain | 4 weeks | 1 RCT  (Aggarwal & Verma, 2018) | very serious^a^ | NA | not serious | very serious^b^ | none | 19 | 19 | -0.72 (-1.38, -0.06) | -1.20 (-2.24, -0.16) | SNAG + CT | ⨁◯◯◯ Very low |
| 6.228 | | SNAG + CT + ex vs. Maitland +CT + ex | | Pain | 4 weeks | 1 RCT  (Shehri et al., 2018) | very serious^a^ | NA | not serious | very serious^b^ | none | 25 | 25 | 0.91 (0.33, 1.49) | 0.62 (0.25, 0.99) | Maitland + CT + ex | ⨁◯◯◯ Very low |
| 6.229 | | SNAG + ex vs. Maitland + ex | | Pain | 4 weeks | 1 RCT (Tanveer et al., 2017) | very serious^a^ | NA | not serious | very serious^b^ | none | 25 | 25 | -2.06 (-2.76, -1.37) | -1.64 (-2.07, -1.21) | SNAG + ex | ⨁◯◯◯ Very low |
| 6.230 | | SNAG + ex vs. ex | | Pain | 4 weeks | 1 RCT (Tanveer et al., 2017) | very serious^a^ | NA | not serious | very serious^b^ | none | 25 | 25 | -2.11 (-2.81, -1.41) | -1.60 (-2.01, -1.19) | SNAG + ex | ⨁◯◯◯ Very low |
| 6.231 | | Self-SNAG + CT vs. CT | | Pain | 4 weeks | 1 RCT  (Aggarwal & Verma, 2018) | very serious^a^ | NA | not serious | very serious^b^ | none | 19 | 19 | -0.72 (-1.00, -0.09) | -1.20 (-2.24, -0.16) | Self-SNAG + CT | ⨁◯◯◯ Very low |
| 6.232 | | SNAGs + MET + CT vs SNAGs + CT | | Pain | 8 weeks | 1 RCT  (Sultan et al., 2021) | very serious^a^ | NA | not serious | very serious^b^ | none | 27 | 27 | -1.04 (-1.61, -0.47) | -0.85 (-1.28, -0.42) | SNAGs + MET + CT | ⨁◯◯◯ Very low |
|  | **Range of motion** | | | | | | | | | | | | | | | | |
| 6.233 | | SNAGs + CT vs CT | | Flexion | 2 weeks | 1 RCT  (Ozlu & Sahin, 2024) | very serious^a^ | NA | not serious | very serious^b,c^ | none | 20 | 20 | 15.17 (11.62, 18.73) | 16.80 (16.13, 17.47) | SNAGs + CT | ⨁◯◯◯ Very low |
| 6.234 | | MET (post-isometric relaxation) vs. NAGs | | Flexion | 4 weeks | 1 RCT  (Usama et al., 2022) | very serious^a^ | NA | not serious | very serious^b^ | none | 23 | 22 | 4.79 (3.60, 5.98) | 23.80 (20.94, 26.66) | NAG | ⨁◯◯◯ Very low |
| 6.235 | | SNAGs+NAGs+CT +ex vs CT+ex | | Flexion | 4 weeks | 1 RCT  (Gautam et al., 2014) | very serious^a^ | NA | not serious | very serious^b^ | none | 10 | 10 | 0.75 (-0.17, 1.66) | 8.07 (-1.02, 17.16) | Significant improvement in both groups, no statistical differences | ⨁◯◯◯ Very low |
| 6.236 | | SNAGs + MET + CT vs SNAGs + CT | | Flexion | 8 weeks | 1 RCT  (Sultan et al., 2021) | very serious^a^ | NA | not serious | very serious^b^ | none | 27 | 27 | 3.23 (2.40, 4.06) | 7.88 (6.60, 9.16) | SNAGs + MET + CT | ⨁◯◯◯ Very low |
| 6.237 | | SNAGs + CT vs CT | | Extension | 2 weeks | 2 RCTs  (Aggarwal & Verma, 2018; Ozlu & Sahin, 2024) | very serious^a^ | not serious | not serious | very serious^b^ | none | 39 | 39 | 0.22 (-0.84, 1.29) | 3.95 (-9.73, 17.63) | Significant improvement in both groups, no statistical differences between interventions | ⨁◯◯◯ Very low |
| 6.238 | | Self-SNAG + CT vs. CT | | Extension | 2 weeks | 1 RCT  (Aggarwal & Verma, 2018) | very serious^a^ | NA | not serious | very serious^b^ | none | 19 | 19 | -0.32 (-0.96, 0.32) | -2.50 (-7.37, 2.37) | Significant improvement in both groups, no statistical differences | ⨁◯◯◯ Very low |
| 6.239 | | MET (post-isometric relaxation) vs. NAGs | | Extension | 4 weeks | 1 RCT  (Usama et al., 2022) | very serious^a^ | NA | not serious | very serious^b^ | none | 23 | 22 | 3.87 (2.85, 4.89) | 19.22 (16.40, 22.04) | NAG | ⨁◯◯◯ Very low |
| 6.240 | | Self-SNAG + CT vs. CT | | Extension | 4 weeks | 1 RCT  (Aggarwal & Verma, 2018) | very serious^a^ | NA | not serious | very serious^b^ | none | 19 | 19 | -0.29 (-0.93, 0.35) | -2.20 (-7.00, 2.60) | Significant improvement in both groups, no statistical differences | ⨁◯◯◯ Very low |
| 6.241 | | SNAGs+NAGs+CT +ex vs CT+ex | | Extension | 4 weeks | 1 RCT  (Gautam et al., 2014) | very serious^a^ | NA | not serious | very serious^b^ | none | 10 | 10 | 0.43 (-0.46, 1.31) | 3.85 (-3.74, 11.44) | Significant improvement in both groups, no statistical differences | ⨁◯◯◯ Very low |
| 6.242 | | SNAGs + MET + CT vs SNAGs + CT | | Extension | 8 weeks | 1 RCT  (Sultan et al., 2021) | very serious^a^ | NA | not serious | very serious^b^ | none | 27 | 27 | 2.42 (1.71, 3.14) | 3.34 (2.62, 4.06) | SNAGs + MET + CT | ⨁◯◯◯ Very low |
| 6.243 | | SNAGs + CT vs CT | | Left lateral flexion | 2 weeks | 2 RCTs  (Aggarwal & Verma, 2018; Ozlu & Sahin, 2024) | very serious^a^ | not serious | not serious | very serious^b,c^ | none | 20 | 20 | 0.70 (-0.39, 1.78) | 5.50 (-3.51, 14.52) | Significant improvement in both groups, no statistical differences | ⨁◯◯◯ Very low |
| 6.244 | | Self-SNAG + CT vs. CT | | Left lateral flexion | 2 weeks | 1 RCT  (Aggarwal & Verma, 2018) | very serious^a^ | NA | not serious | very serious^b^ | none | 19 | 19 | 0.15 (-0.49, 0.79) | 1.00 (-3.17, 5.17) | Significant improvement in both groups, no statistical differences | ⨁◯◯◯ Very low |
| 6.245 | | Self-SNAG + CT vs. CT | | Left lateral flexion | 4 weeks | 1 RCT  (Aggarwal & Verma, 2018) | very serious^a^ | NA | not serious | very serious^b^ | none | 19 | 19 | 0.15 (-0.49, 0.78) | 1.00 (-3.26, 5.26) | Significant improvement in both groups, no statistical differences | ⨁◯◯◯ Very low |
| 6.246 | | SNAGs+NAGs+CT +ex vs CT+ex | | Left lateral flexion | 4 weeks | 1 RCT  (Gautam et al., 2014) | very serious^a^ | NA | not serious | very serious^b^ | none | 10 | 10 | 2.39 (1.19, 3.60) | 17.14 (11.12, 23.16) | SNAGs+NAGs+CT +ex | ⨁◯◯◯ Very low |
| 6.247 | | SNAGs + MET + CT vs SNAGs + CT | | Left lateral flexion | 8 weeks | 1 RCT  (Sultan et al., 2021) | very serious^a^ | NA | not serious | very serious^b^ | none | 27 | 27 | 2.28 (1.59, 2.98) | 4.07 (3.13, 5.01) | SNAGs + MET + CT | ⨁◯◯◯ Very low |
| 6.248 | | SNAGs + CT vs CT | | Right lateral flexion | 2 weeks | 2 RCTs  (Aggarwal & Verma, 2018; Ozlu & Sahin, 2024) | very serious^a^ | not serious | not serious | very serious^b^ | none | 39 | 39 | 0.48 (-0.32, 1.29) | 3.97 (-3.22, 11.17) | Significant improvement in both groups, no statistical differences | ⨁◯◯◯ Very low |
| 6.249 | | Self-SNAG + CT vs. CT | | Right lateral flexion | 2 weeks | 1 RCT  (Aggarwal & Verma, 2018) | very serious^a^ | NA | not serious | very serious^b^ | none | 19 | 19 | 0.08 (-0.56, 0.71) | 0.50 (-3.60, 4.60) | Significant improvement in both groups, no statistical differences | ⨁◯◯◯ Very low |
| 6.250 | | Self-SNAG + CT vs. CT | | Right lateral flexion | 4 weeks | 1 RCT  (Aggarwal & Verma, 2018) | very serious^a^ | NA | not serious | very serious^b^ | none | 19 | 19 | 0.13 (-0.50, 0.77) | 0.90 (-3.33, 5.13) | Significant improvement in both groups, no statistical differences | ⨁◯◯◯ Very low |
| 6.251 | | SNAGs+NAGs+CT +ex vs CT+ex | | Right lateral flexion | 4 weeks | 1 RCT  (Gautam et al., 2014) | very serious^a^ | NA | not serious | very serious^b^ | none | 10 | 10 | 0.71 (-0.20,1.63) | 8.35 (-1.46, 18.16) | Significant improvement in both groups, no statistical differences | ⨁◯◯◯ Very low |
| 6.252 | | SNAGs + MET + CT vs SNAGs + CT | | Right lateral flexion | 8 weeks | 1 RCT  (Sultan et al., 2021) | very serious^a^ | NA | not serious | very serious^b^ | none | 27 | 27 | 1.49 (0.88, 2.09) | 3.77 (2.44, 5.10) | SNAGs + MET + CT | ⨁◯◯◯ Very low |
| 6.253 | | Self-SNAG + CT vs. CT | | Left rotation | 2 weeks | 1 RCT  (Aggarwal & Verma, 2018) | very serious^a^ | NA | not serious | very serious^b^ | none | 19 | 19 | 0.55 (-0.10, 1.19) | 5.30 (-0.74, 11.34) | Significant improvement in both groups, no statistical differences | ⨁◯◯◯ Very low |
| 6.254 | | SNAGs + CT vs CT | | Left rotation | 2 weeks | 2 RCTs  (Aggarwal & Verma, 2018; Ozlu & Sahin, 2024) | very serious^a^ | not serious | not serious | very serious^b,c^ | none | 20 | 20 | 3.00 (-1.89, 7.88) | 15.10 (-3.71, 33.91) | Significant improvement in both groups, no statistical differences | ⨁◯◯◯ Very low |
| 6.255 | | Self-SNAG + CT vs. CT | | Left rotation | 4 weeks | 1 RCT  (Aggarwal & Verma, 2018) | very serious^a^ | NA | not serious | very serious^b^ | none | 19 | 19 | 0.58 (-0.07, 1.23) | 5.60 (-0.41, 11.61) | Significant improvement in both groups, no statistical differences | ⨁◯◯◯ Very low |
| 6.256 | | SNAGs+NAGs+CT +ex vs CT+ex | | Left rotation | 4 weeks | 1 RCT  (Gautam et al., 2014) | very serious^a^ | NA | not serious | very serious^b^ | none | 10 | 10 | 0.12 (-0.76, 1.00) | 1.35 (-8.17, 10.87) | Significant improvement in both groups, no statistical differences | ⨁◯◯◯ Very low |
| 6.257 | | SNAGs + MET + CT vs SNAGs + CT | | Left rotation | 8 weeks | 1 RCT  (Sultan et al., 2021) | very serious^a^ | NA | not serious | very serious^b^ | none | 27 | 27 | 2.90 (2.12, 3.67) | 6.04 (4.94, 7.14) | SNAGs + MET + CT | ⨁◯◯◯ Very low |
| 6.258 | | Self-SNAG + CT vs. CT | | Right rotation | 2 weeks | 1 RCT  (Aggarwal & Verma, 2018) | very serious^a^ | NA | not serious | very serious^b^ | none | 19 | 19 | 0.52 (-0.13, 1.17) | 5.00 (-1.00, 11.00) | Significant improvement in both groups, no statistical differences | ⨁◯◯◯ Very low |
| 6.259 | | SNAGs + CT vs CT | | Right rotation | 2 weeks | 2 RCTs  (Aggarwal & Verma, 2018; Ozlu & Sahin, 2024) | very serious^a^ | not serious | not serious | very serious^b^ | none | 39 | 39 | 2.48 (-1.43, 6.40) | 12.87 (-2.07, 27.81) | Significant improvement in both groups, no statistical differences | ⨁◯◯◯ Very low |
| 6.260 | | Self-SNAG + CT vs. CT | | Right rotation | 4 weeks | 1 RCT  (Aggarwal & Verma, 2018) | very serious^a^ | NA | not serious | very serious^b^ | none | 19 | 19 | 0.65 (-0.01, 1.30) | 6.50 (0.25, 12.75) | Significant improvement in both groups, no statistical differences | ⨁◯◯◯ Very low |
| 6.261 | | SNAGs+NAGs+CT +ex vs CT+ex | | Right rotation | 4 weeks | 1 RCT  (Gautam et al., 2014) | very serious^a^ | NA | not serious | very serious^b^ | none | 10 | 10 | 0.84 (-0.09, 1.76) | 7.69 (-0.03, 15.41) | Significant improvement in both groups, no statistical differences | ⨁◯◯◯ Very low |
| 6.262 | | SNAGs + MET + CT vs SNAGs + CT | | Right rotation | 8 weeks | 1 RCT  (Sultan et al., 2021) | very serious^a^ | NA | not serious | very serious^b^ | none | 27 | 27 | 2.27 (1.58, 2.97) | 4.89 (3.76, 6.02) | SNAGs + MET + CT | ⨁◯◯◯ Very low |
|  | **Disability** | | | | | | | | | | | | | | | | |
| 6.263 | | SNAGs + CT vs. CT | | Disability | 2 weeks | 2 RCTs  (Aggarwal & Verma, 2018; Ozlu & Sahin, 2024) | very serious^a^ | not serious | not serious | very serious^b^ | none | 39 | 39 | -6.86 (-19.58, 5.86) | -13.05 (-29.47, 3.38) | Significant improvement in both groups, no statistical differences | ⨁◯◯◯ Very low |
| 6.264 | | Self-SNAG + CT vs. CT | | Disability | 2 weeks | 1 RCT  (Aggarwal & Verma, 2018) | very serious^a^ | NA | not serious | very serious^b^ | none | 19 | 19 | -0.47 (-1.11, 0.18) | -4.40 (-10.28, 1.48) | Significant improvement in both groups, no statistical differences | ⨁◯◯◯ Very low |
| 6.265 | | MET (post-isometric relaxation) vs. NAGs | | Disability | 4 weeks | 1 RCT  (Usama et al., 2022) | very serious^a^ | NA | not serious | very serious^b^ | none | 23 | 22 | -2.55 (-3.36, -1.75) | -7.83 (-9.60, -6.06) | NAG | ⨁◯◯◯ Very low |
| 6.266 | | Self-SNAG + CT vs. CT | | Disability | 4 weeks | 1 RCT  (Aggarwal & Verma, 2018) | very serious^a^ | NA | not serious | very serious^b^ | none | 19 | 19 | NR | NR | - | ⨁◯◯◯ Very low |
| 6.267 | | SNAG + ex vs. Maitland + ex | | Disability | 4 weeks | 1 RCT (Tanveer et al., 2017) | very serious^a^ | NA | not serious | very serious^b^ | none | 25 | 25 | -1.15 (-1.75, -0.55) | -3.24 (-4.78, -1.70) | SNAG + ex | ⨁◯◯◯ Very low |
| 6.268 | | SNAG + ex vs ex | | Disability | 4 weeks | 1 RCT (Tanveer et al., 2017) | very serious^a^ | NA | not serious | very serious^b^ | none | 25 | 25 | 0.21 (-0.34, 0.77) | 0.52 (-0.82, 1.86) | Significant improvement in both groups, no statistical differences | ⨁◯◯◯ Very low |
| 6.269 | | Mulligan+CT+ex vs Maitland +CT+ex | | Disability | 4 weeks | 1 RCT (Shehri et al., 2018) | very serious^a^ | NA | not serious | very serious^b^ | none | 35 | 35 | -0.02 (-0.49, 0.45) | -3.57 (-17.08, 9.94) | Significant improvement in both groups, no statistical differences | ⨁◯◯◯ Very low |

1. High risk of bias assessed by the RoB2;
2. The sample size is considered small (<300);
3. Confidence intervals relatively wide.
4. Although the overall risk of bias of compiled studies is low, some concerns were observed for the deviations from intended interventions and selection of the reported results;
5. Unclear concealment, compliance and adherence, co-interventions not reported, no reasons for dropouts;
6. There is a high inconsistency between studies (I²=>80);
7. Heterogeneity between studies is lower than 80%.
8. Two studies presented a high risk of bias at the RoB2 (Shamsi et al, 2021; Tachii et al., 2018);
9. One study (Buyukturan et al, 2018) presented final score as "some concerns" at the RoB2, while Shamsi et al. (2021) showed a high risk of bias at the RoB2
10. This result comes from one study reported in two manuscripts. The pooled effect from the two reports for this study were combined.
11. Some concerns at RoB

# Mixed population: Acute, subacute and chronic neck pain

CCFT: neck motor control training using the craniocervical flexion test; CI: confidence interval; CT: conventional therapy; ex: exercise; HVLA: high velocity low amplitude; MD: mean difference; MET: muscle energy techniques; MRF: myofascial release; MWM: Mobilisation with movement; NAGs: Natural Apophyseal Glides; PAIVMs: passive accessory intervertebral movements; PRT: positional release therapy; RCT: Randomized controlled trial; SMD: standardized mean difference; NAGs: Sustained Natural Apophyseal Glides

References:

Abd El-Azeim, A. S., & Grase, M. O. (2023). Efficacy of Mulligan on electromyography activation of cervical muscles in mechanical neck pain: randomized experimental trial. *Physiotherapy Quarterly*, *31*(4). <https://doi.org/https://dx.doi.org/10.5114/pq.2023.117224>

Aggarwal, S., & Verma, M. (2018). Efficacy of Mulligan’s self-sustained natural apophyseal glides in decreasing pain, disability, and improving neck mobility among the nursing professional suffering from work-related neck pain. *Arch Med Health Sci*, *6*(1), 48-53.

Alansari, S. M., Youssef, E. F., & Shanb, A. A. (2021). Efficacy of manual therapy on psychological status and pain in patients with neck pain. A randomized clinical trial. *Saudi Med J*, *42*(1), 82-90. <https://doi.org/10.15537/smj.2021.1.25589>

Ali, A., Shakil-Ur-Rehman, S., & Sibtain, F. (2014). The efficacy of Sustained Natural Apophyseal Glides with and without Isometric Exercise Training in Non-specific Neck Pain. *Pakistan journal of medical sciences*, *30*(4), 872-874.

Alshami, A. M., & AlSadiq, A. I. (2021). Outcomes of scapulothoracic mobilisation in patients with neck pain and scapular dyskinesis: A randomised clinical trial. *J Taibah Univ Med Sci*, *16*(4), 540-549. <https://doi.org/10.1016/j.jtumed.2021.03.006>

Buyukturan, O., Buyukturan, B., Sas, S., Kararti, C., & Ceylan, I. (2018). The Effect of Mulligan Mobilization Technique in Older Adults with Neck Pain: A Randomized Controlled, Double-Blind Study [Randomized Controlled Trial]. *Pain Research & Management*, *2018*, 2856375. <https://doi.org/https://dx.doi.org/10.1155/2018/2856375>

Duymaz, T., & Yagci, N. (2018). Effectiveness of the mulligan mobilization technique in mechanical neck pain. *Journal of Clinical and Analytical Medicine*, *9*(4), 304-309. <https://doi.org/http://dx.doi.org/10.4328/JCAM.5715>

El-Sodany, A. M., Alayat, M. S. M., & Zafer, A. M. I. (2014). Sustained natural apophyseal glides mobilization versus manipulation in the treatment of cervical spine disorders: a randomized controlled trial. *International Journal*, *2*(6), 274-280.

Ganesh, G. S., Mohanty, P., Pattnaik, M., & Mishra, C. (2015). Effectiveness of mobilization therapy and exercises in mechanical neck pain [Comparative Study

Randomized Controlled Trial]. *Physiotherapy Theory & Practice*, *31*(2), 99-106. <https://doi.org/https://dx.doi.org/10.3109/09593985.2014.963904>

Gautam, R., Dhamija, J. K., Puri, A., Trivedi, P., Sathiyavani, D., & Nambi, G. (2014). Comparison of Maitland and Mulligan mobilization in improving neck pain, ROM and disability. *Int J Physiother Res*, *2*(3), 482-487.

Hussain, S. I., Ahmad, A., Amjad, F., Shafi, T., & Shahid, H. A. (2016). Effectiveness of natural apophyseal glides versus grade I and II Maitland mobilization in Non-specific neck pain. *Annals of King Edward Medical University Lahore Pakistan*, *22*, 23-29.

Izquierdo Perez, H., Alonso Perez, J. L., Gil Martinez, A., La Touche, R., Lerma-Lara, S., Commeaux Gonzalez, N., Arribas Perez, H., Bishop, M. D., & Fernandez-Carnero, J. (2014). Is one better than another?: A randomized clinical trial of manual therapy for patients with chronic neck pain. *Man Ther*, *19*(3), 215-221. <https://doi.org/10.1016/j.math.2013.12.002>

Kumar, D., Sandhu, J. S., & Broota, A. (2011). Efficacy of mulligan concept (NAGs) on pain at available end range in cervical spine: A randomised controlled trial. *Indian J. Physiotherap. Occup. Ther. Indian Journal of Physiotherapy and Occupational Therapy*, *5*(1), 154-158.

Lopez-Lopez, A., Alonso Perez, J. L., Gonzalez Gutierez, J. L., La Touche, R., Lerma Lara, S., Izquierdo, H., & Fernandez-Carnero, J. (2015). Mobilization versus manipulations versus sustain apophyseal natural glide techniques and interaction with psychological factors for patients with chronic neck pain: randomized controlled trial [Randomized Controlled Trial]. *European journal of physical & rehabilitation medicine.*, *51*(2), 121-132.

Manzoor, A., Anwar, N., Khalid, K., Haider, R., Saghir, M., & Javed, M. A. (2021). Comparison of effectiveness of muscle energy technique with Mulligan mobilization in patients with non-specific neck pain. *J Pak Med Assoc*, *71*(6), 1532-1524. <https://doi.org/10.47391/JPMA.981>

Mohamed, E. E., & Elrazik, R. K. A. (2020). Sustained natural apophyseal glides versus positional release therapy in the treatment of chronic mechanical neck dysfunction [Article]. *International Journal of Human Movement and Sports Sciences*, *8*(6), 384-394. <https://doi.org/10.13189/saj.2020.080610>

Morsi, A. A., Al-Kabalawy, M. A., Aneis, Y. M., Hamza, M. S., & Atta, H. K. (2023). Effect of Sustained Natural Apophyseal Glides and Myofascial Release on Chronic Nonspecific Neck Pain: Randomized Controlled Trial. *Journal of Population Therapeutics and Clinical Pharmacology*, *30(8)*, e390-e404. <https://doi.org/https://dx.doi.org/10.47750/jptcp.2023.30.08.042>

Ozlu, O., & Sahin, M. (2024). The effect of mulligan mobilization technique application in addition to conventional physiotherapy on pain and joint range of motion in people with neck pain. *Journal of Bodywork and Movement Therapies*, *39*, 225-230. <https://doi.org/https://dx.doi.org/10.1016/j.jbmt.2024.02.009>

Rezkallah, S. S., & Abdullah, G. A. (2018). Comparison between sustained natural apophyseal glides (SNAG’s) and myofascial release techniques combined with exercises in non specific neck pain. *Physiotherapy Practice & Research*, *39*(2), 135-145. <https://doi.org/10.3233/PPR-180116>

Said, M. S., Ali, O. I., Elazm, S. N. A., & Abdelraoof, N. A. (2017). Mulligan self mobilization versus Mulligan snags on cervical position sense. *International Journal of Physiotherapy*, *4*(2), 93-100.

Shamsi, S., Alyazedi, F., Abdelkader, S., Khan, S., & Akhtar, A. (2021). Efficacy of sustained natural apophyseal glides in the management of mechanical neck pain: A randomized clinical trial. *Indian Journal of Medical Specialities*, *12*(4). <https://doi.org/10.4103/injms.injms_30_21>

Shehri, A. A., Khan, S., Shami, S., & Almureef, S. S. (2018). COMPARATIVE STUDY OF MULLIGAN (SNAGS) AND MAITLAND MOBILIZATION IN NECK PAIN. *European Journal of Physical Education and Sport Science*, *5*(1), 19-29. <https://doi.org/doi>: 10.5281/zenodo.1481977

Shelke, A., Prabhu, B. A., Balthillaya, M. G., Kumaran, S. D., & Raja, G. P. (2023). Immediate effect of craniocervical flexion exercise and Mulligan mobilisation in patients with mechanical neck pain - A randomised clinical trial. *Hong Kong Physiotherapy Journal*, *43(2)*, 137-147. <https://doi.org/https://dx.doi.org/10.1142/S1013702523500154>

Sultan, N., Khushnood, K., Altaf, S., Awan, M. M. A., Qureshi, S., & Mehmood, R. (2021). Muscle Energy Technique Augmented with Sustained Natural Apophyseal Glides; An Effective Way to Improve Mechanical Neck Pain and Range of Motion: A Randomized Control Trial [Article]. *Journal of Islamic International Medical College*, *16*(2), 96-100. <https://www.scopus.com/inward/record.uri?eid=2-s2.0-85150527555&partnerID=40&md5=e40920e5fc54e272b271b3389a9bb3fd>

Sun, X., Chai, L., Huang, Q., Zhou, H., & Liu, H. (2024). Effects of exercise combined with cervicothoracic spine self-mobilization on chronic non-specific neck pain. *Scientific reports*, *14(1)*, 5298. <https://doi.org/https://dx.doi.org/10.1038/s41598-024-55181-8>

Tachii, R., sen, s., & Arfath, U. (2015). Short term effect of sustained apohuseal glides on cervical joint position sense, pain, and neck disability in patients with chronic neck pain *International Journal of Therapies and Rehabilitation Research*, *4*(4), 244.

Tank, K., Choksi, P., & Makwana, P. (2018). To study the effect of muscle energy technique versus Mulligan SNAGs on pain, range of motion, and functional disability for individuals with mechanical neck pain: A comparative study. *International Journal of Physiotherapy and Research*, *6*(1), 2582-2587.

Tanveer, F., Afzal, M., Adeel, S., Shahid, S., & Masood, M. (2017). Comparison of sustained natural apophyseal glides and maitland manual therapy in non-specific neck pain on numeric pain rating scale and neck disability index. *Annals of King Edward Medical University*, *23*.

Usama, J., Iram, A., Sania, M., Saddiqa, Q., Hafiz Muhammad Uzair, A., Aliza, T., Arifa, M., & Mudassar, I. (2022). Comparative Effect of Muscle Energy Techniques and Mulligan Mobilization on Pain & Range of Motion in patients with Mechanical Neck Pain. In (pp. 195-199): CrossLinks International Publishers.

Vijayan, K., Sivaraman, A., Kumaresan, P., & Palani, J. (2022). Short-term Effect of Mulligan SNAGs on Pain Intensity, Cervical Range of Motion and Craniovertebral Angle in Patients with Non Specific Neck Pain: A Quasi-experimental Study. *Journal of Clinical and Diagnostic Research*, *16(7)*, YC05-YC08. <https://doi.org/https://dx.doi.org/10.7860/JCDR/2022/55962.16547>

Waqas, S., Shah, S. H. A., Zafar, U., & Akhtar, M. F. (2017). Comparison of Mulligan Sustained Natural Apophyseal Glides Versus Mulligan Natural Apophyseal Glides in Mechanical Neck Pain. . *Annals of King Edward Medical University*, *23*. <https://doi.org/https://doi.org/10.21649/akemu.v23i3.2007>

Zemadanis, K. (2018). The short and mid-term effects of Mulligan concept in patients with chronic mechanical neck pain. *J Nov Physiother Rehabil*, *2*(2), 022-021.
