# Supplementary material for: The Effectiveness of Mulligan's Techniques in Non‐Specific Neck Pain: A Systematic Review and Meta‐Analysis
Source: Physiother Res Int. 2025 May 29;30(3):e70045. doi: 10.1002/pri.70045 (PMC12121345; doi:10.1002/pri.70045)
Supplement: Supplementary file 7 — Supporting Information S7 [file PRI-30-e70045-s004.docx]

**Appendix 7. Summary (matrix) of the results.**

| **Pain intensity** | | | | | | | | |
| --- | --- | --- | --- | --- | --- | --- | --- | --- |
| **Therapy of interest** | | **Comparator** | **Timepoint** | **Acute neck pain** | **Mixed chronicity neck pain** | **Chronic neck pain** | **Unclear chronicity neck pain** | **GRADE ID** |
| SNAG + CT+ exercises | | MET + CT + exercises | 2 weeks | 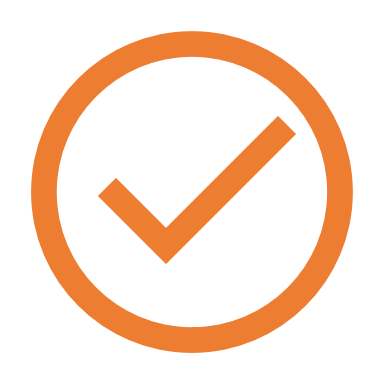🗹 (Tank et al., 2018) | 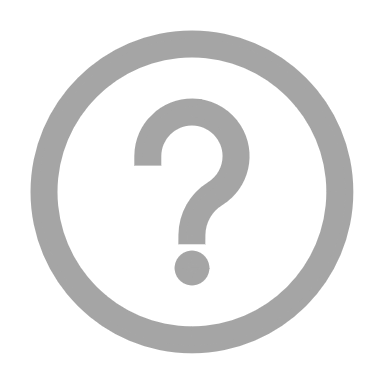 | 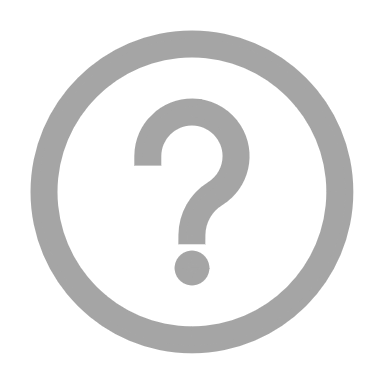 | 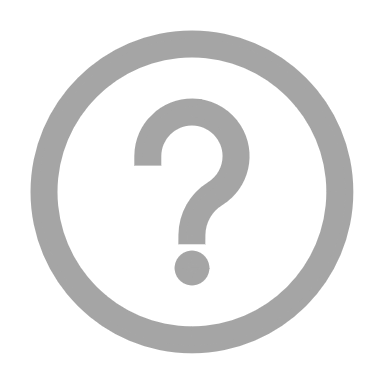 | 6.1 |
| SNAG + exercises | | Mobilization (PAIVMs) + exercises | 2 weeks | 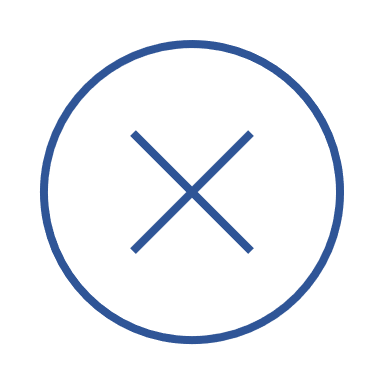(Ganesh et al., 2015) | 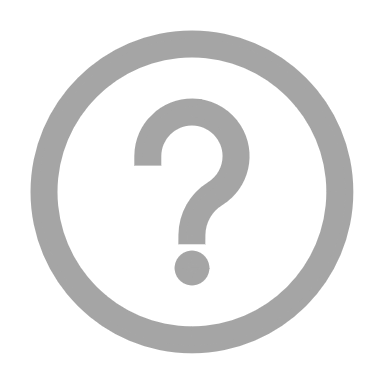 | 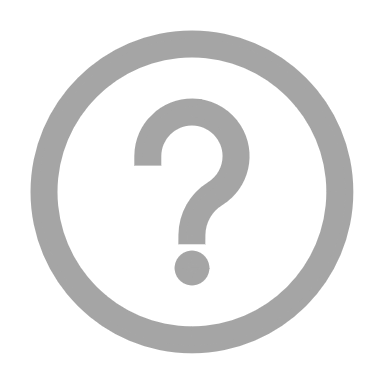 | 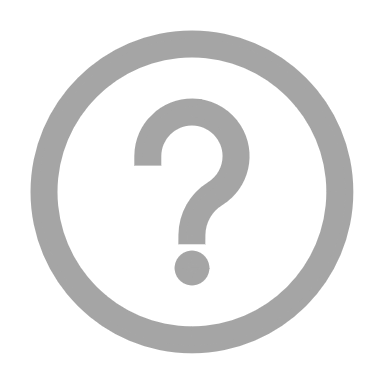 | 6.2 |
| SNAG + exercises | | Exercises | 2/4 weeks | 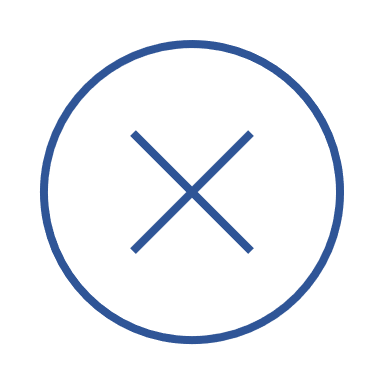(Ganesh et al., 2015) | 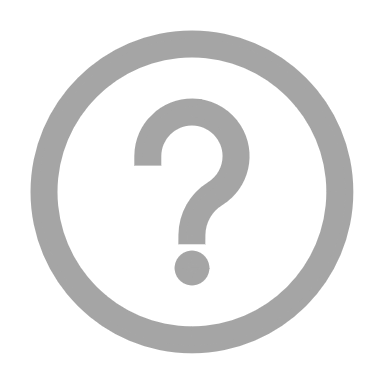 | 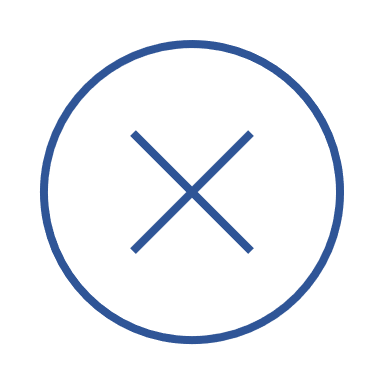🗹 (Duymaz & Yagci, 2018; Rezkallah & Abdullah, 2018) | 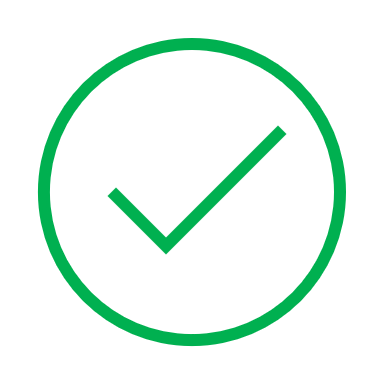🗹(Tanveer et al., 2017) | 6.3;  6.75; 6.230 |
|  | |  | 12 weeks | 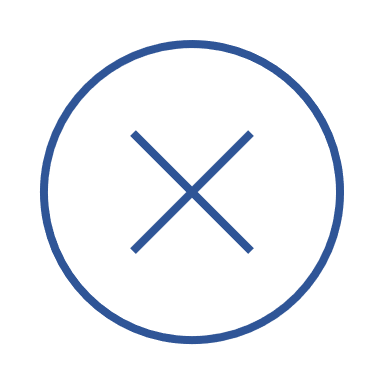(Ganesh et al., 2015) | 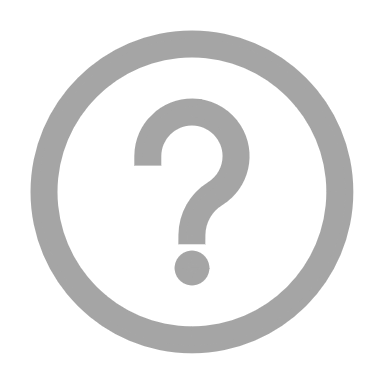 | 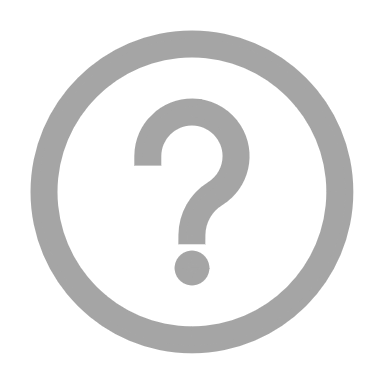 | 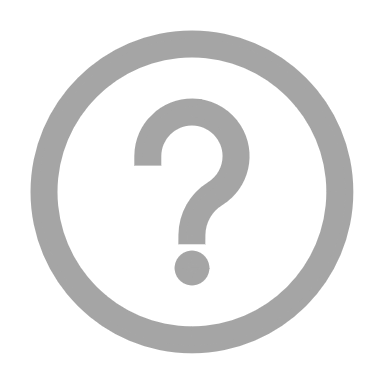 | 6.4 |
| SNAG + exercises | | Mobilization (PAIVMs) + exercises | 12 weeks | 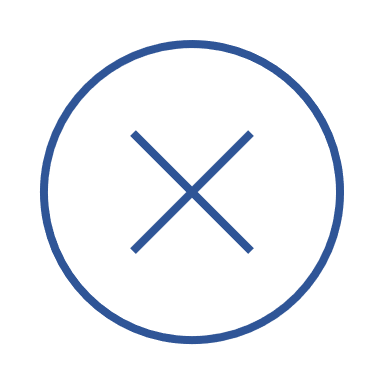(Ganesh et al., 2015) | 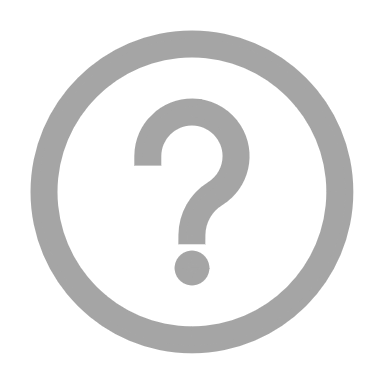 | 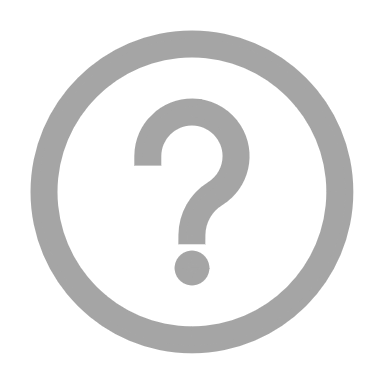 | 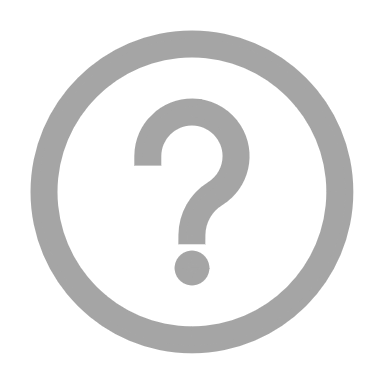 | 6.5 |
| SNAG | | Mobilization (PAIVMs) | 3 weeks | 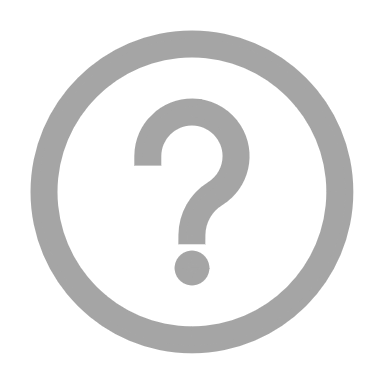 | 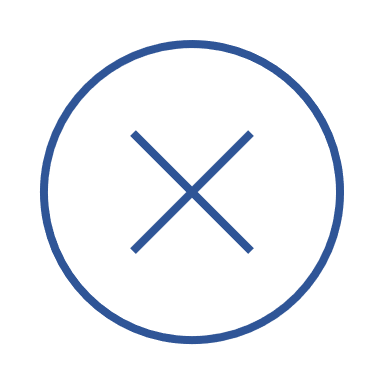(Alansari et al., 2021) | 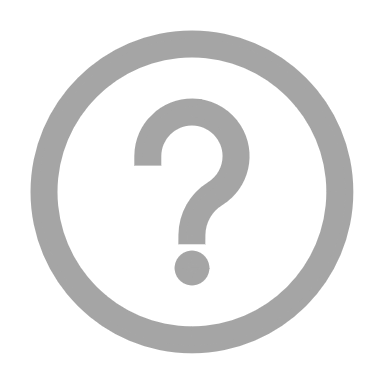 | 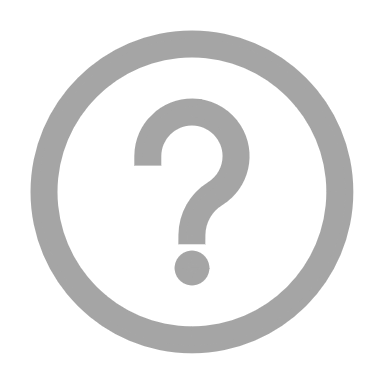 | 6.37 |
| SNAG | | CCFT | Single-session | 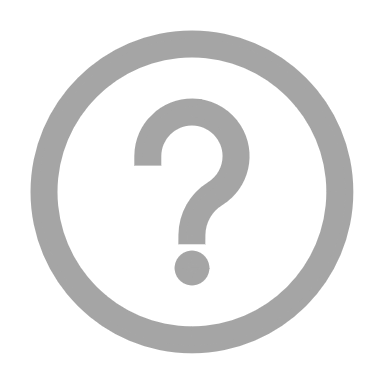 | 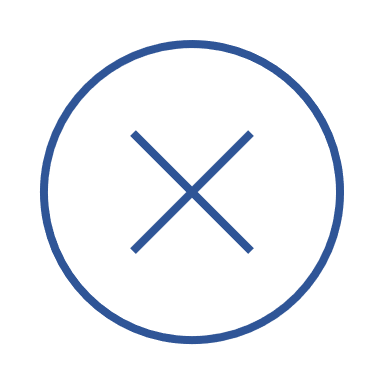(Shelke et al., 2023 | 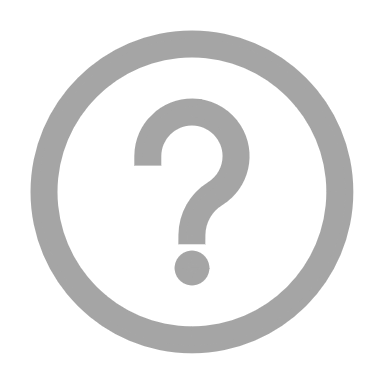 | 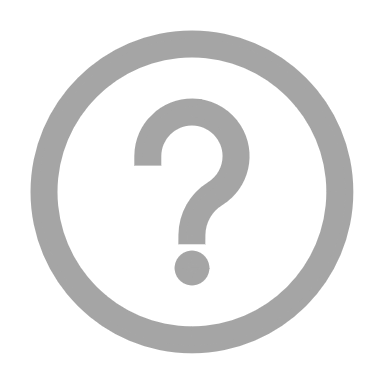 | 6.39 |
| SNAGs + Interferential therapy + Isometric neck exercises | | Interferential therapy + Isometric neck exercises | 2 weeks | 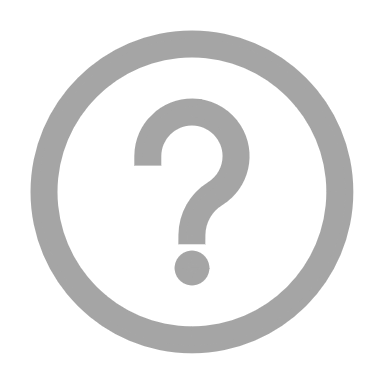 | 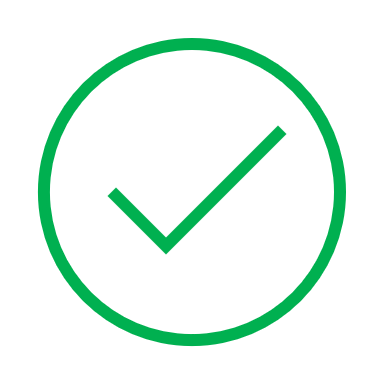🗹 (Vijayan et al., 2022) | 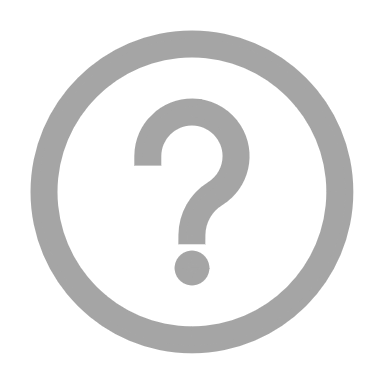 | 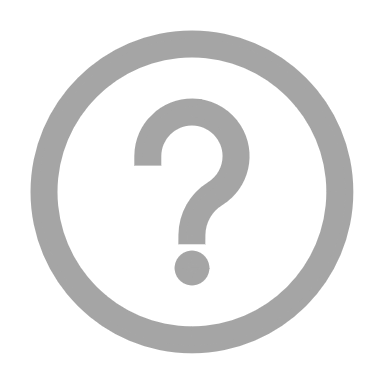 | 6.46 |
| NAGS + CT | | Maitland + CT | 2 weeks | 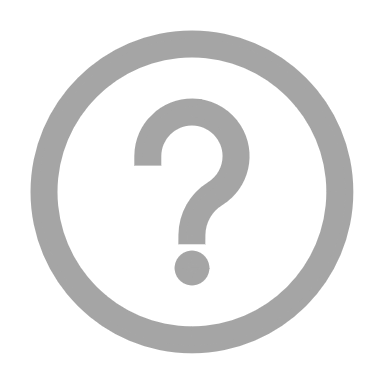 | 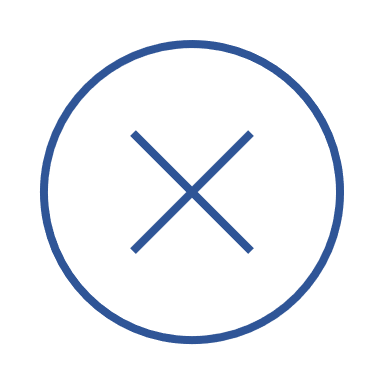 (Hussain et al., 2016) | 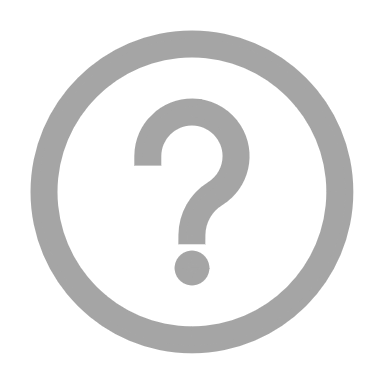 | 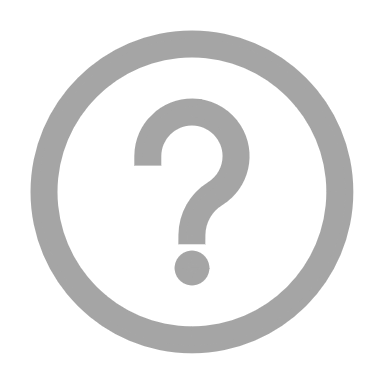 | 6.51 |
| NAGS + CT | | Maitland + CT | 4 weeks | 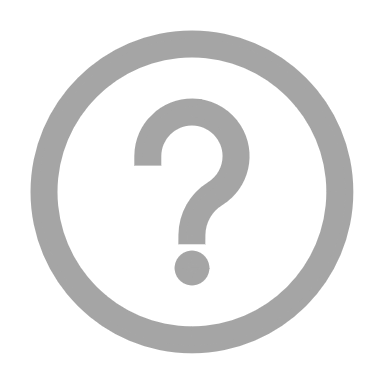 | 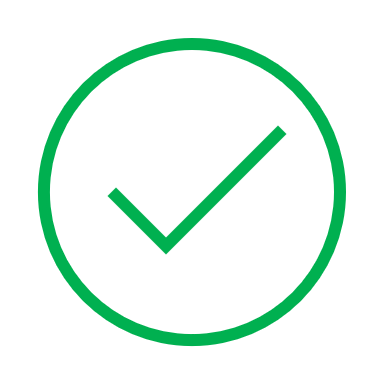 (Hussain et al., 2016) | 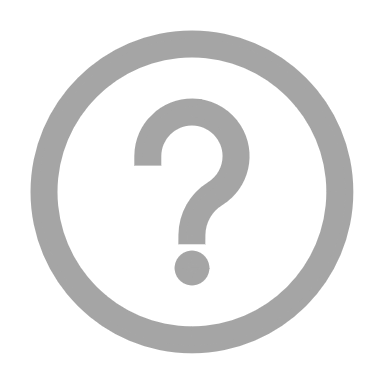 | 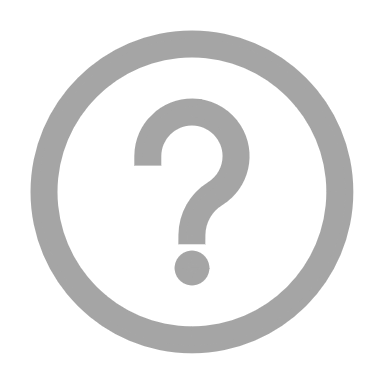 | 6.52 |
| SNAGs | | HVLA | Single-session | 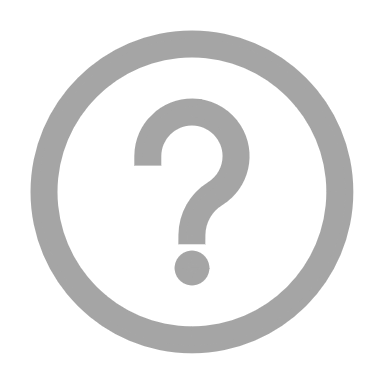 | 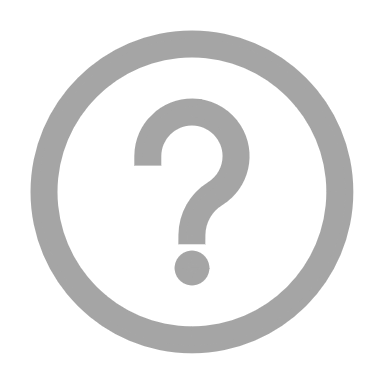 | 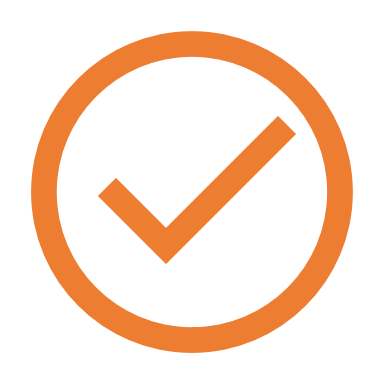🗹 (Izquierdo Perez et al., 2014; Lopez-Lopez et al., 2015) | 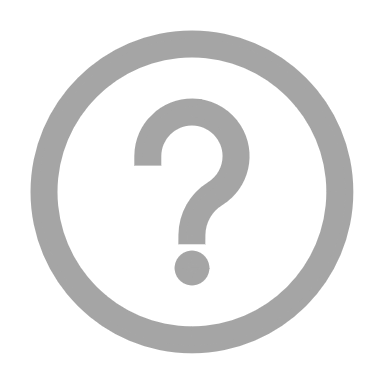 | 6.68 |
| SNAG | | Mobilization (PAIVMs) | Single-session | 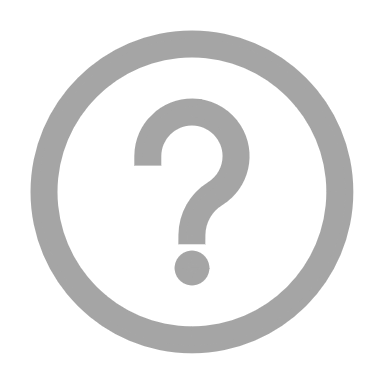 | 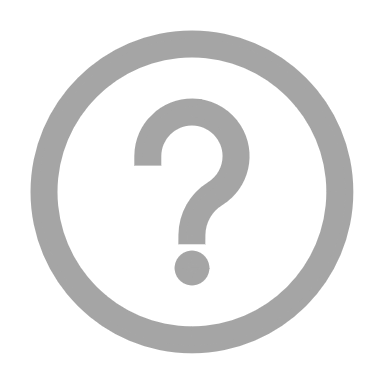 | 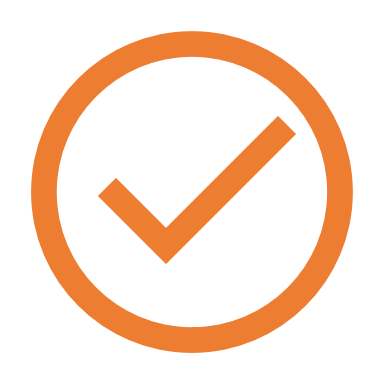🗹 (Izquierdo Perez et al., 2014; Lopez-Lopez et al., 2015) | 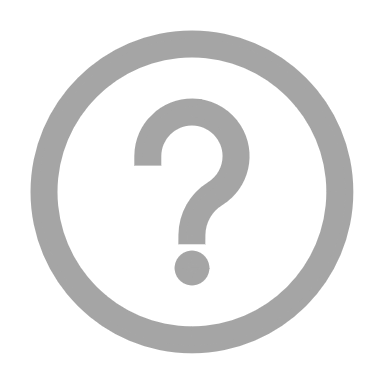 | 6.69 |
| SNAG + CT+ exercises | | CT+ exercises | 2-4 weeks | 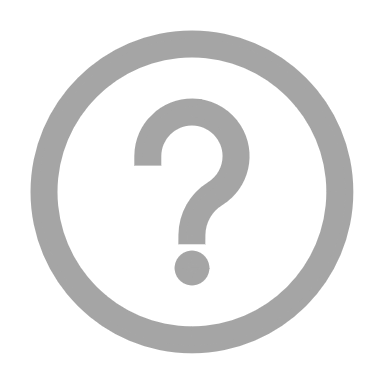 | 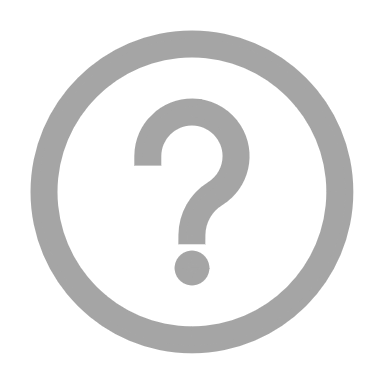 | 🗹 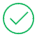 (Buyukturan et al., 2018; Shamsi et al., 2021; Tachii et al., 2015) | 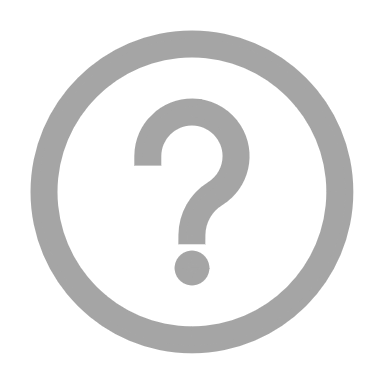 | 6.70 |
|  |  |  |  |  |  | 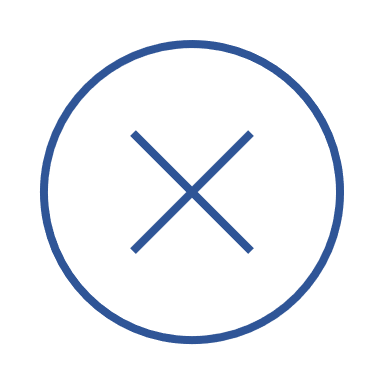🗹(Abd El-Azeim & Grase, 2023; Buyukturan et al., 2018; Shamsi et al., 2021; Tachii et al., 2015) |  |  |
| MWM for the scapula | | CT + taping | 2 weeks | 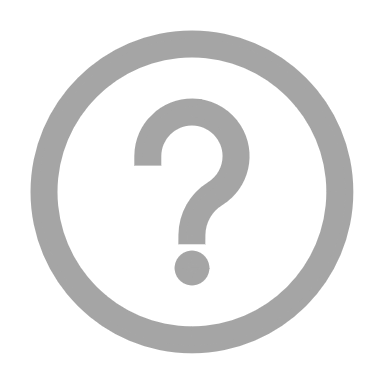 | 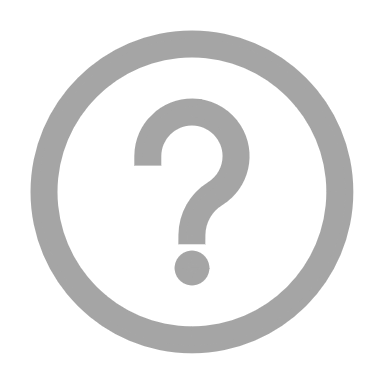 | 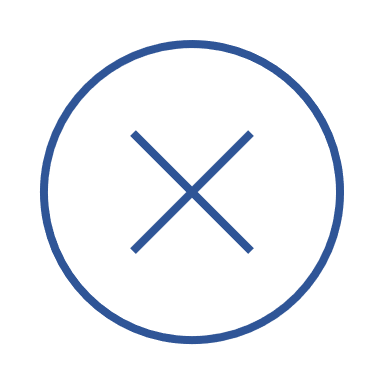(Alshami & AlSadiq, 2021) | 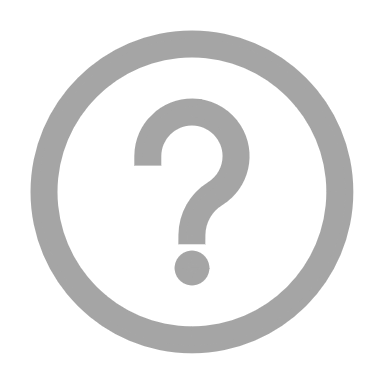 | 6.71 |
| MWM for the scapula | | CT + taping | 3 weeks | 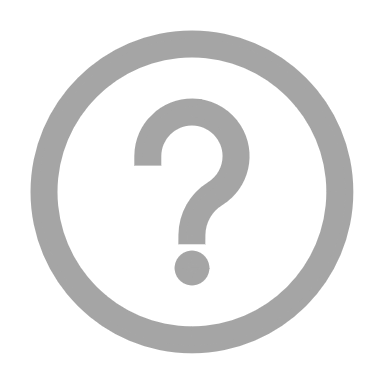 | 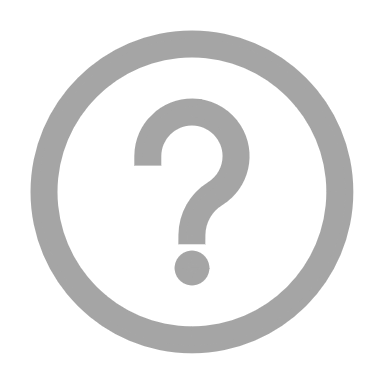 | 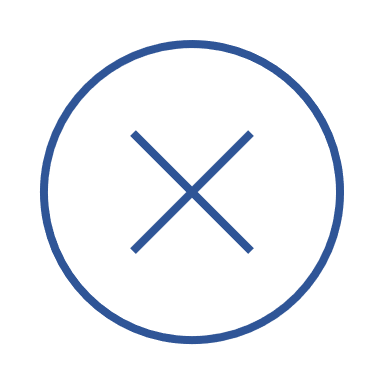(Alshami & AlSadiq, 2021) | 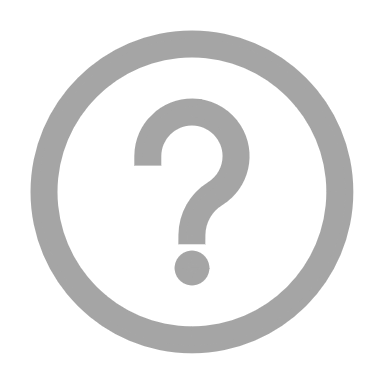 | 6.72 |
| SNAGs + NAGs | | MET | 3 weeks | 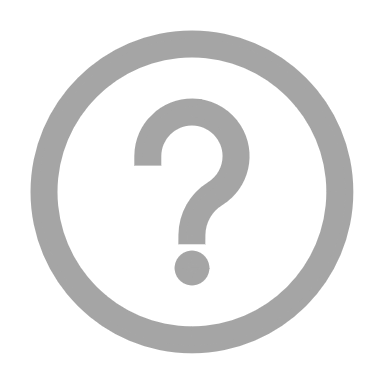 | 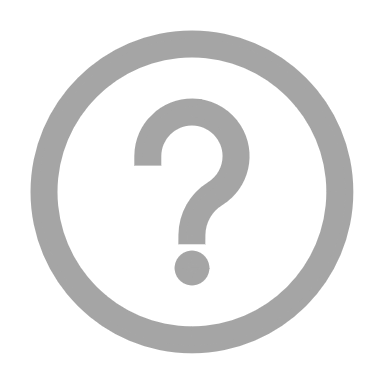 | 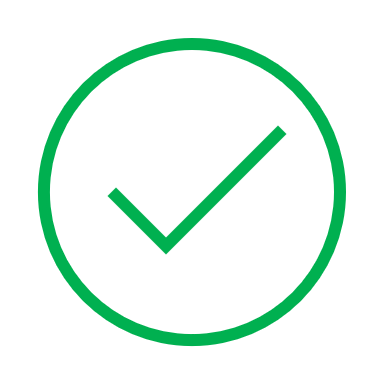**×** (Manzoor et al., 2021) | 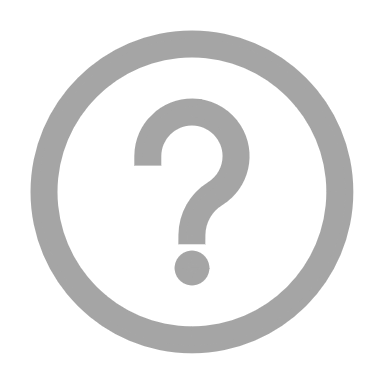 | 6.73 |
| SNAG+NAG+self-SNAG | | Sham | 3 weeks | 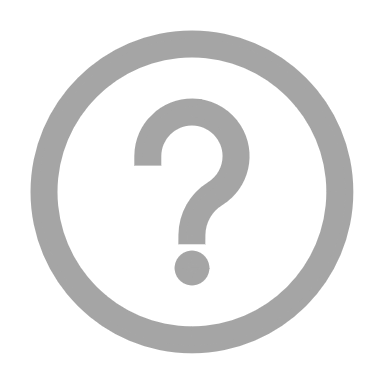 | 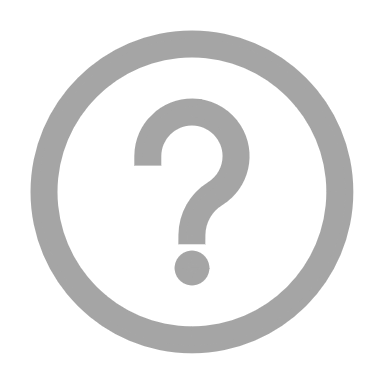 | 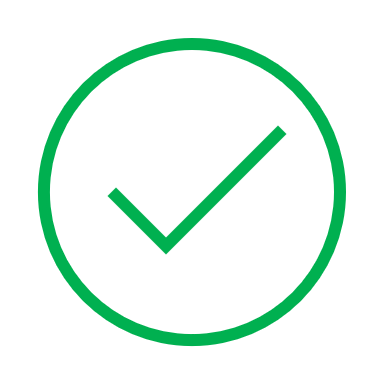🗹 (Zemadanis, 2018) | 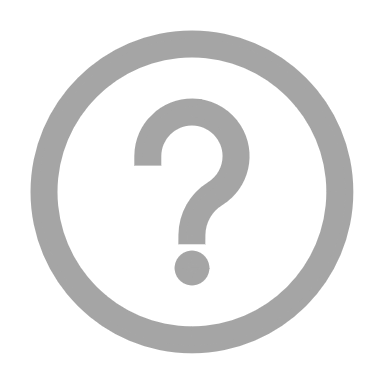 | 6.74 |
| SNAGs + exercise | | Exercises | 2-4 weeks | 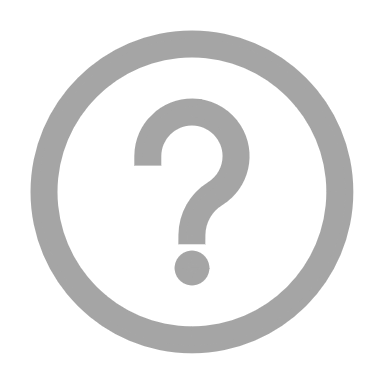 | 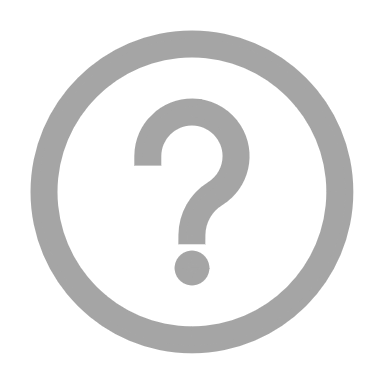 | 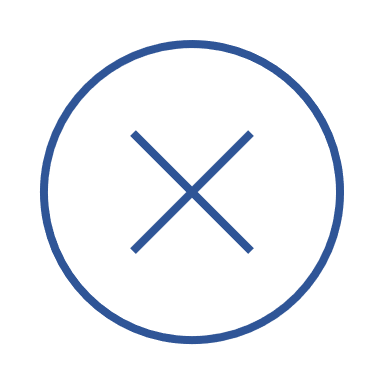🗹  (Duymaz & Yagci, 2018; Rezkallah & Abdullah, 2018) | 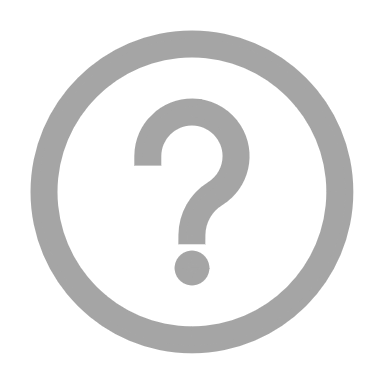 | 6.75 |
| SNAGs + exercise | | Myofascial release + exercise | 4 weeks | 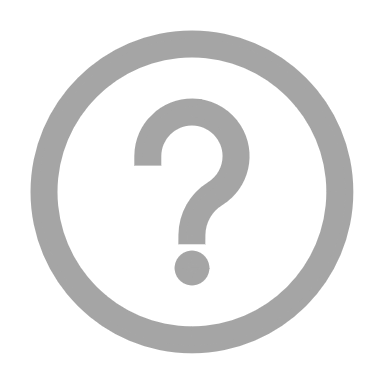 | 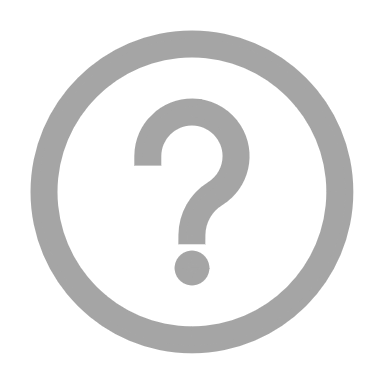 | 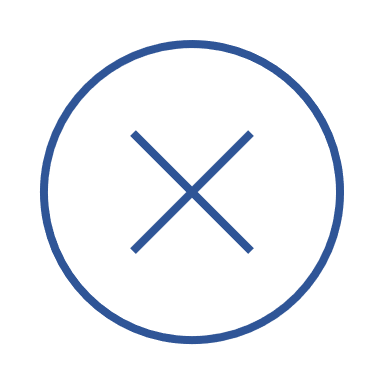(Alshami & AlSadiq, 2021) | 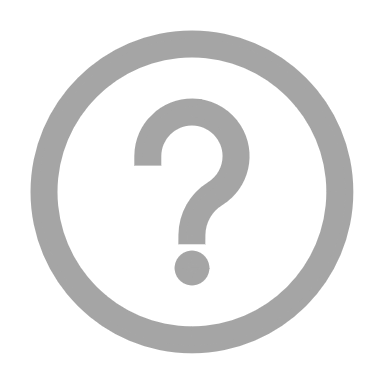 | 6.76 |
| SNAGs | | HVLA | 4 weeks | 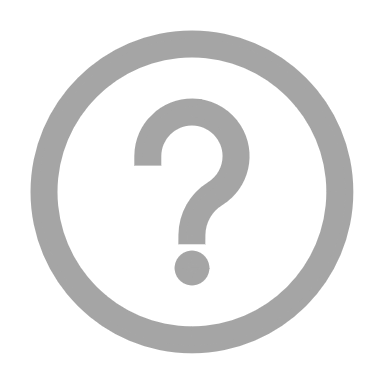 | 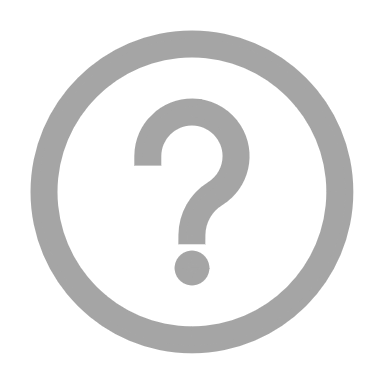 | 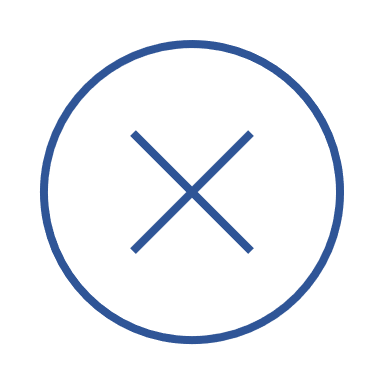 (Izquierdo Perez et al., 2014) | 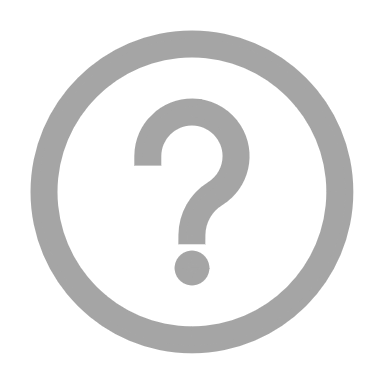 | 6.77 |
| SNAG | | Mobilization (PAIVMs) | 4 weeks | 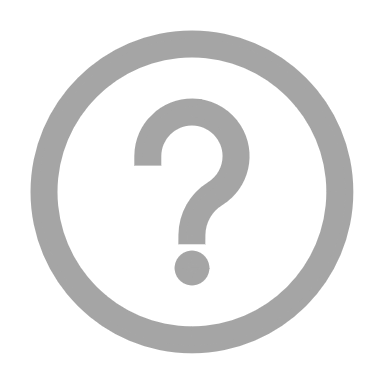 | 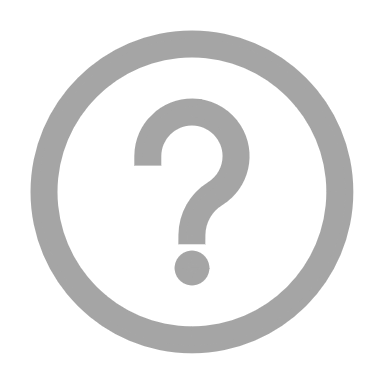 | 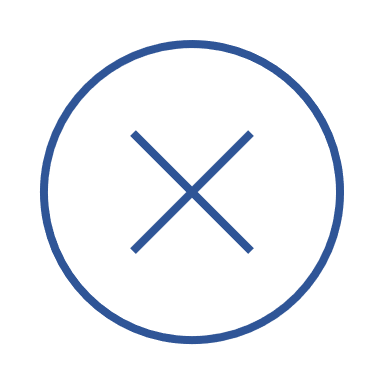 (Izquierdo Perez et al., 2014) | 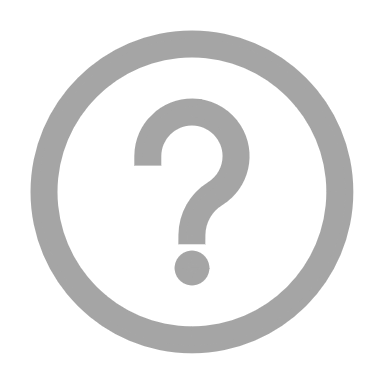 | 6.78 |
| Self-SNAGs + CT | | CT | 2-4 weeks | 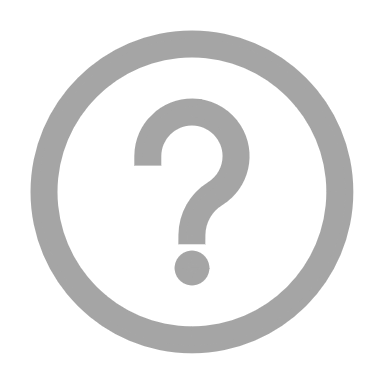 | 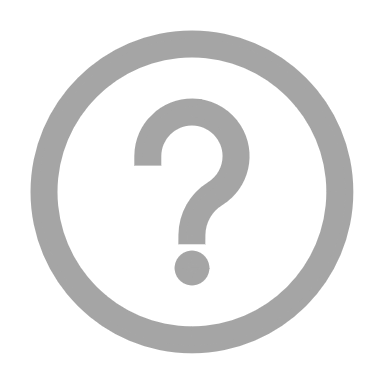 | 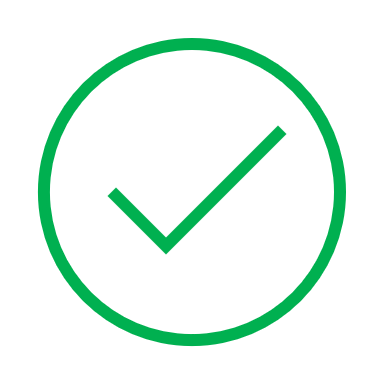🗹 (Said et al., 2017) | 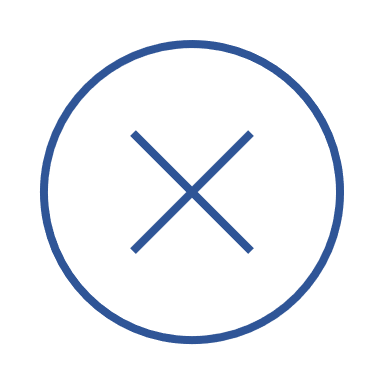 (Aggarwal & Verma, 2018) | 6.79; 6.225 |
| SNAGs + CT | | CT | 2-4 weeks | 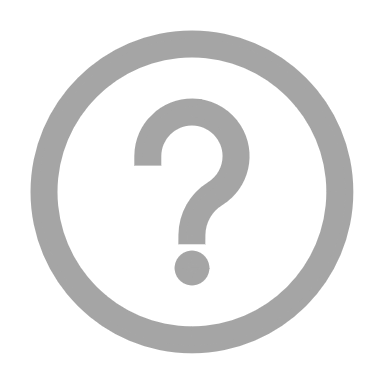 | 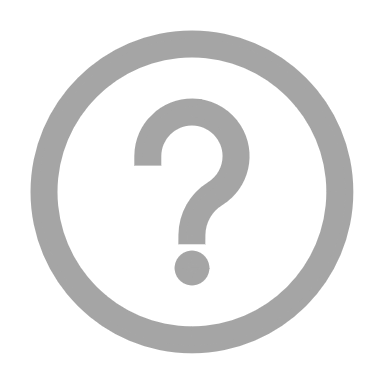 | 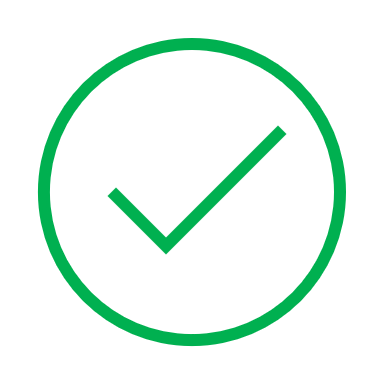🗹 (Said et al., 2017) | 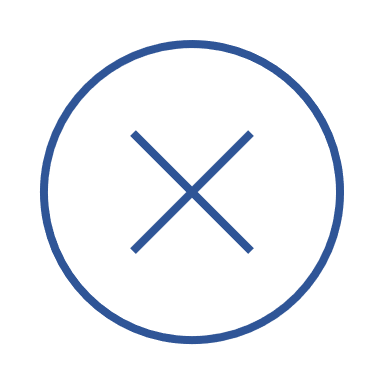🗹 (Aggarwal & Verma, 2018; Ozlu & Sahin, 2024) | 6.80; 6.223 |
| SNAG+NAG+self-SNAG | | Sham | 4 weeks | 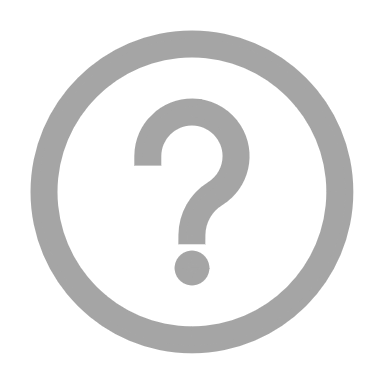 | 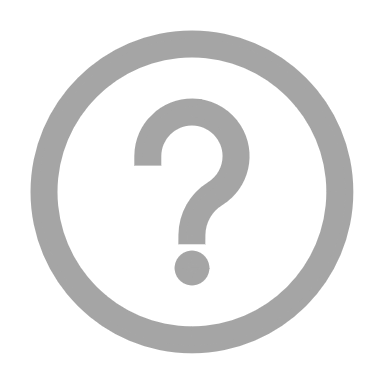 | 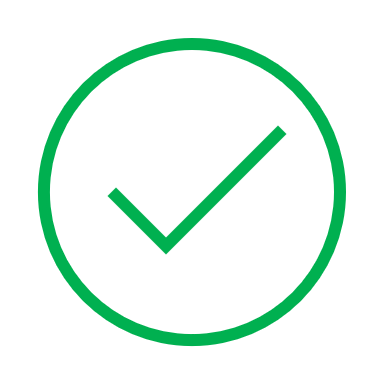🗹 (Zemadanis, 2018) | 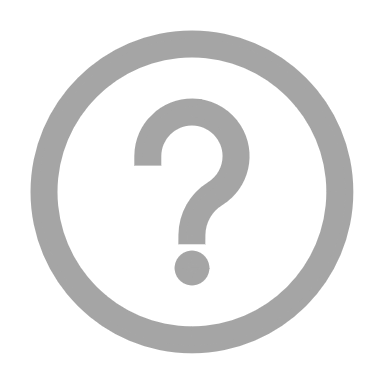 | 6.81 |
| Self-SNAGs + exercise/CCFT | | Cervicothoracic mobilization + exercise/CCFT | 6 weeks | 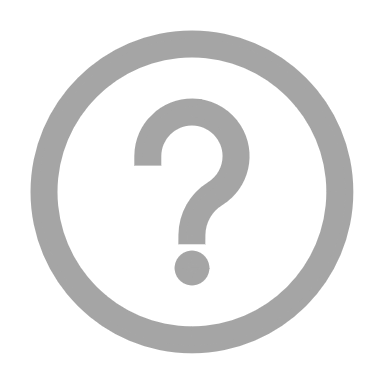 | 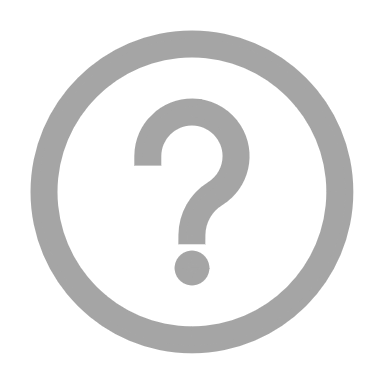 | 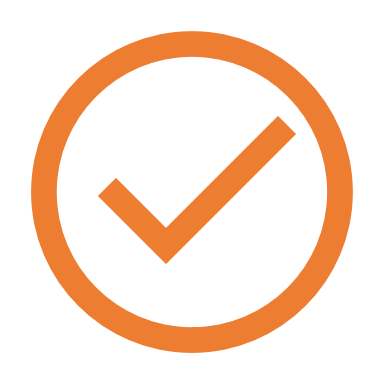🗹 (Sun et al., 2024) |  | 6.83 |
| Self-SNAGs + exercise/CCFT | | exercise/CCFT | 6 weeks |  |  | (Sun et al., 2024) |  | 6.84 |
| SNAG + CT | | PRT + CT + exercise | 8 weeks |  |  | 🗹 (Mohamed & Elrazik, 2020) |  | 6.85 |
| SNAGs | | MFR | 8 weeks |  |  | (Morsi et al., 2023) |  | 6.86 |
| SNAGs | | SNAGs + MFR | 8 weeks |  |  | (Morsi et al., 2023 |  | 6.87 |
| SNAGs + MFR | | MFR | 8 weeks |  |  | (Morsi et al., 2023) |  | 6.88 |
| SNAGs | | HVLA | 8 weeks |  |  | (Izquierdo Perez et al., 2014) |  | 6.89 |
| SNAG | | Mobilization (PAIVMs) | 8 weeks |  |  | (Izquierdo Perez et al., 2014; Lopez-Lopez et al., 2015) |  | 6.90 |
| SNAGs | | HVLA | 12 weeks |  |  | (Izquierdo Perez et al., 2014) |  | 6.91 |
| SNAG | | Mobilization (PAIVMs) | 8 weeks |  |  | (Izquierdo Perez et al., 2014; Lopez-Lopez et al., 2015) |  | 6.92 |
| SNAGs + CT | | NAGs + CT | 2 weeks |  |  |  | 🗹 (Waqas et al., 2017) | 6.224 |
| Muscle energy technique (post-isometric relaxation) | | NAGs | 4 weeks |  |  |  | 🗹 (Usama et al., 2022) | 6.226 |
| SNAGs + CT | | CT | 4 weeks |  |  |  | (Aggarwal & Verma, 2018) | 6.227 |
| SNAG + CT + ex | | Maitland +CT + ex | 4 weeks |  |  |  | 🗹 (Usama et al., 2022) | 6.228 |
| SNAG + ex | | Maitland + ex | 4 weeks |  |  |  | 🗹 (Tanveer et al., 2017) | 6.229 |
| SNAG + ex | | Exercise | 4 weeks |  |  |  | 🗹 (Tanveer et al., 2017) | 6.230 |
| Self-SNAG + CT | | CT | 4 weeks |  |  |  | (Aggarwal & Verma, 2018) | 6.231 |
| SNAGs + MET + CT | | SNAGs + CT | 8 weeks |  |  |  | 🗹 (Sultan et al., 2021) | 6.232 |
| **Cervical range of motion – Flexion** | | | | | | | | |
| **Therapy of interest** | | **Comparator** | **Timepoint** | **Acute neck pain** | **Mixed chronicity neck pain** | **Chronic neck pain** | **Unclear chronicity neck pain** | **GRADE comparison** |
| SNAG | | MET + CT | 2 weeks | (Tank et al., 2018) |  |  |  | 6.6 |
| SNAG | | CCFT | Single-session |  | (Shelke et al., 2023 |  |  | 6.40 |
| SNAGs + Interferential therapy + Isometric neck exercises | | Interferential therapy + Isometric neck exercises | 2 weeks |  | 🗹 (Vijayan et al., 2022) |  |  | 6.47 |
| SNAGs | | HVLA | Single-session |  |  | 🗹 (Izquierdo Perez et al., 2014; Lopez-Lopez et al., 2015) |  | 6.94 |
| SNAG | | Mobilization (PAIVMs) | Single-session |  |  | (Izquierdo Perez et al., 2014; Lopez-Lopez et al., 2015) |  | 6.95 |
| SNAG + CT+ exercises | | CT+ exercises | 2 weeks |  |  | 🗹 (Buyukturan et al., 2018; Shamsi et al., 2021) |  | 6.96 |
| SNAGs + NAGs | | MET | 3 weeks |  |  | 🗹 (Manzoor et al., 2021) |  | 6.97 |
| MWM for the scapula | | CT + taping | 2 weeks |  |  | (Alshami & AlSadiq, 2021) |  | 6.98 |
| MWM for the scapula | | CT + taping | 3 weeks |  |  | (Alshami & AlSadiq, 2021) |  | 6.99 |
| SNAGs + Exercise | | Exercises | 2/4 weeks |  |  | 🗹 (Duymaz & Yagci, 2018; Rezkallah & Abdullah, 2018) |  | 6.100 |
| SNAGs + exercise | | Myofascial release + exercise | 4 weeks |  |  | (Rezkallah & Abdullah, 2018) |  | 6.101 |
| SNAGs | | HVLA | 4 weeks |  |  | (Izquierdo Perez et al., 2014) |  | 6.102 |
| SNAGs | | Mobilization (PAIVMs) | 4 weeks |  |  | (Izquierdo Perez et al., 2014) |  | 6.103 |
| Self-SNAGs + exercise/CCFT | | Cervicothoracic mobilization + exercise/CCFT | 6 weeks |  |  | (Sun et al., 2024) |  | 6.104 |
| Self-SNAGs + exercise/CCFT | | exercise/CCFT | 6 weeks |  |  | (Sun et al., 2024) |  | 6.105 |
| SNAGs | | MFR | 8 weeks |  |  | (Morsi et al., 2023) |  | 6.106 |
| SNAGs | | SNAGs + MFR | 8 weeks |  |  | (Morsi et al., 2023 |  | 6.107 |
| SNAGs + MFR | | MFR | 8 weeks |  |  | (Morsi et al., 2023) |  | 6.108 |
| SNAG + CT | | PRT + CT + exercise | 8 weeks |  |  | 🗹 (Mohamed & Elrazik, 2020) |  | 6.109 |
| SNAGs | | HVLA | 8 weeks |  |  | (Izquierdo Perez et al., 2014) |  | 6.110 |
| SNAGs | | Mobilization (PAIVMs) | 8 weeks |  |  | (Izquierdo Perez et al., 2014) |  | 6.111 |
| SNAGS + exercise | | Exercise | 12 weeks |  |  | 🗹 (Duymaz & Yagci, 2018) |  | 6.112 |
| SNAGs | | HVLA | 12 weeks |  |  | (Izquierdo Perez et al., 2014) |  | 6.113 |
| SNAGs | | Mobilization (PAIVMs) | 12 weeks |  |  | (Izquierdo Perez et al., 2014) |  | 6.114 |
| SNAGs + CT | | CT | 2 weeks |  |  |  | 🗹 (Ozlu & Sahin, 2024) | 6.233 |
| NAGs | | Muscle energy technique (post-isometric relaxation) | 4 weeks |  |  |  | 🗹 (Usama et al., 2022) | 6.234 |
| SNAGs+NAGs+CT +ex | | CT+ex | 4 weeks |  |  |  | (Gautam et al., 2014) | 6.235 |
| SNAGs + MET + CT | | SNAGs + CT | 8 weeks |  |  |  | 🗹 (Sultan et al., 2021) | 6.236 |
| **Cervical range of motion – Extension** | | | | | | | | |
| SNAG + exercises | | Mobilization (PAIVMs) + exercises | 2 weeks | (Ganesh et al., 2015) |  |  |  | 6.7 |
| SNAG + exercises | | Exercises | 2 weeks | (Ganesh et al., 2015) |  |  |  | 6.8 |
| SNAG | | MET + CT | 2 weeks | (Tank et al., 2018) |  |  |  | 6.9 |
| SNAG + exercises | | Mobilization (PAIVMs) + exercises | 12 weeks | (Ganesh et al., 2015) |  |  |  | 6.10 |
| SNAG + exercises | | Exercises | 12 weeks | (Ganesh et al., 2015) |  |  |  | 6.11 |
| SNAG | | CCFT | Single-session |  | (Shelke et al., 2023 |  |  | 6.41 |
| SNAGs + Interferential therapy + Isometric neck exercises | | Interferential therapy + Isometric neck exercises | 2 weeks |  | 🗹 (Vijayan et al., 2022) |  |  | 6.48 |
| SNAGs | | HVLA | Single-session |  |  | 🗹 (Izquierdo Perez et al., 2014; Lopez-Lopez et al., 2015) |  | 6.115 |
| SNAG | | Mobilization (PAIVMs) | Single-session |  |  | (Izquierdo Perez et al., 2014; Lopez-Lopez et al., 2015) |  | 6.116 |
| SNAG + CT+ exercises | | MET + CT + exercises | 2 weeks |  |  | 🗹 (Buyukturan et al., 2018; Shamsi et al., 2021) |  | 6.117 |
| MWM for the scapula | | CT + taping | 2 weeks |  |  | (Alshami & AlSadiq, 2021) |  | 6.118 |
| SNAGs + Exercise | | Exercises | 2/4 weeks |  |  | 🗹 (Duymaz & Yagci, 2018; Rezkallah & Abdullah, 2018) |  | 6.119 |
| SNAGs + NAGs | | MET | 3 weeks |  |  | 🗹 (Manzoor et al., 2021) |  | 6.120 |
| MWM for the scapula | | CT + taping | 3 weeks |  |  | (Alshami & AlSadiq, 2021) |  | 6.121 |
| SNAGs + exercise | | Myofascial release + exercise | 4 weeks |  |  | (Alshami & AlSadiq, 2021) |  | 6.122 |
| SNAGs | | HVLA | 4 weeks |  |  | (Izquierdo Perez et al., 2014) |  | 6.123 |
| SNAGs | | Mobilization (PAIVMs) | 4 weeks |  |  | (Izquierdo Perez et al., 2014) |  | 6.124 |
| Self-SNAGs + exercise/CCFT | | Cervicothoracic mobilization + exercise/CCFT | 6 weeks |  |  | (Sun et al., 2024) |  | 6.125 |
| Self-SNAGs + exercise/CCFT | | Exercise/CCFT | 6 weeks |  |  | 🗹 (Sun et al., 2024) |  | 6.126 |
| SNAGs | | MFR | 8 weeks |  |  | (Morsi et al., 2023) |  | 6.127 |
| SNAGs | | SNAGs + MFR | 8 weeks |  |  | (Morsi et al., 2023 |  | 6.128 |
| SNAGs + MFR | | MFR | 8 weeks |  |  | (Morsi et al., 2023) |  | 6.129 |
| SNAG + CT | | PRT + CT + exercise | 8 weeks |  |  | 🗹 (Mohamed & Elrazik, 2020) |  | 6.130 |
| SNAGs | | HVLA | 8 weeks |  |  | (Izquierdo Perez et al., 2014) |  | 6.131 |
| SNAGs | | Mobilization (PAIVMs) | 8 weeks |  |  | (Izquierdo Perez et al., 2014) |  | 6.132 |
| SNAGS + exercise | | Exercise | 12 weeks |  |  | 🗹 (Duymaz & Yagci, 2018) |  | 6.133 |
| SNAGs | | HVLA | 12 weeks |  |  | (Izquierdo Perez et al., 2014) |  | 6.134 |
| SNAGs | | Mobilization (PAIVMs) | 12 weeks |  |  | (Izquierdo Perez et al., 2014) |  | 6.135 |
| SNAGs + CT | | CT | 2 weeks |  |  |  | (Aggarwal & Verma, 2018; Ozlu & Sahin, 2024) | 6.237 |
| Self-SNAG + CT | | CT | 2 weeks |  |  |  | (Aggarwal & Verma, 2018) | 6.238 |
| NAGs | | Muscle energy technique (post-isometric relaxation) | 4 weeks |  |  |  | 🗹 (Usama et al., 2022) | 6.239 |
| Self-SNAG+ CT | | CT | 4 weeks |  |  |  | (Aggarwal & Verma, 2018) | 6.240 |
| SNAGs+NAGs+CT +ex | | CT+ex | 4 weeks |  |  |  | (Gautam et al., 2014) | 6.241 |
| SNAGs + MET + CT | | SNAGs + CT | 8 weeks |  |  |  | 🗹 (Sultan et al., 2021) | 6.242 |
| **Cervical range of motion – Lateral flexion** | | | | | | | | |
| SNAGs | | HVLA | Single-session |  |  | (Izquierdo Perez et al., 2014; Lopez-Lopez et al., 2015) |  | 6.136 |
| SNAG | | Mobilization (PAIVMs) | Single-session |  |  | 🗹 (Izquierdo Perez et al., 2014; Lopez-Lopez et al., 2015) |  | 6.137 |
| SNAGs + NAGs | | MET | 3 weeks |  |  | (Manzoor et al., 2021) |  | 6.138 |
| SNAGs | | HVLA | 4 weeks |  |  | (Izquierdo Perez et al., 2014) |  | 6.139 |
| SNAGs | | Mobilization (PAIVMs) | 4 weeks |  |  | (Izquierdo Perez et al., 2014) |  | 6.140 |
| SNAGs | | HVLA | 8 weeks |  |  | (Izquierdo Perez et al., 2014) |  | 6.141 |
| SNAGs | | Mobilization (PAIVMs) | 8 weeks |  |  | (Izquierdo Perez et al., 2014) |  | 6.142 |
| SNAGS + exercise | | Exercise | 12 weeks |  |  | 🗹 (Duymaz & Yagci, 2018) |  | 6.143 |
| SNAGs | | HVLA | 12 weeks |  |  | (Izquierdo Perez et al., 2014) |  | 6.144 |
| SNAGs | | Mobilization (PAIVMs) | 12 weeks |  |  | (Izquierdo Perez et al., 2014) |  | 6.145 |
| **Cervical range of motion – Left lateral flexion** | | | | | | | | |
| SNAG + exercises | | Mobilization (PAIVMs) and exercises | 2 weeks | (Ganesh et al., 2015) |  |  |  | 6.12 |
| SNAG + exercises | | Exercises | 2 weeks | (Ganesh et al., 2015) |  |  |  | 6.13 |
| SNAG | | MET + CT | 2 weeks | (Tank et al., 2018) |  |  |  | 6.14 |
| SNAG + exercises | | Mobilization (PAIVMs) and exercises | 12 weeks | (Ganesh et al., 2015) |  |  |  | 6.15 |
| SNAG + exercises | | Exercises | 12 weeks | (Ganesh et al., 2015) |  |  |  | 6.16 |
| SNAG | | CCFT | Single-session |  | (Shelke et al., 2023 |  |  | 6.42 |
| SNAGs + Interferential therapy + Isometric neck exercises | | Interferential therapy + Isometric neck exercises | 2 weeks |  | 🗹 (Vijayan et al., 2022) |  |  | 6.50 |
| SNAG + CT+ exercises | | CT+ exercises | 2 weeks |  |  | 🗹 (Buyukturan et al., 2018; Shamsi et al., 2021) |  | 6.146 |
| MWM for the scapula | | CT + taping | 2 weeks |  |  | (Alshami & AlSadiq, 2021) |  | 6.147 |
| MWM for the scapula | | CT + taping | 3 weeks |  |  | (Alshami & AlSadiq, 2021) |  | 6.148 |
| SNAGs + Exercise | | Exercises | 2/4 weeks |  |  | 🗹 (Duymaz & Yagci, 2018; Rezkallah & Abdullah, 2018) |  | 6.149 |
| SNAGs + exercise | | Myofascial release + exercise | 4 weeks |  |  | 🗹 (Alshami & AlSadiq, 2021) |  | 6.150 |
| Self-SNAGs + exercise/CCFT | | Cervicothoracic mobilization + exercise/CCFT | 6 weeks |  |  | (Sun et al., 2024) |  | 6.151 |
| Self-SNAGs + exercise/CCFT | | Exercise/CCFT | 6 weeks |  |  | (Sun et al., 2024) |  | 6.152 |
| SNAGs | | MFR | 8 weeks |  |  | (Morsi et al., 2023) |  | 6.153 |
| SNAGs | | SNAGs + MFR | 8 weeks |  |  | 🗹 (Morsi et al., 2023 |  | 6.154 |
| SNAGs + MFR | | MFR | 8 weeks |  |  | 🗹 (Morsi et al., 2023) |  | 6.155 |
| SNAG + CT | | PRT + CT + exercise | 8 weeks |  |  | 🗹 (Mohamed & Elrazik, 2020) |  | 6.156 |
| SNAGs + CT | | CT | 2 weeks |  |  |  | 🗹 (Aggarwal & Verma, 2018; Ozlu & Sahin, 2024) | 6.243 |
| Self-SNAG + CT | | CT | 2 weeks |  |  |  | (Aggarwal & Verma, 2018) | 6.244 |
| Self-SNAG + CT | | CT | 4 weeks |  |  |  | (Aggarwal & Verma, 2018) | 6.245 |
| SNAGs+NAGs+CT +ex | | CT+ex | 4 weeks |  |  |  | 🗹(Gautam et al., 2014) | 6.246 |
| SNAGs + MET + CT | | SNAGs + CT | 8 weeks |  |  |  | 🗹 (Sultan et al., 2021) | 6.247 |
| **Cervical range of motion – Right lateral flexion** | | | | | | | | |
| SNAG + exercises | | Mobilization (PAIVMs) and exercises | 2 weeks | (Ganesh et al., 2015) |  |  |  | 6.17 |
| SNAG + exercises | | Exercises | 2 weeks | (Ganesh et al., 2015) |  |  |  | 6.18 |
| SNAG | | MET + CT | 2 weeks | (Tank et al., 2018) |  |  |  | 6.19 |
| SNAG + exercises | | Mobilization (PAIVMs) and exercises | 12 weeks | (Ganesh et al., 2015) |  |  |  | 6.20 |
| SNAG + exercises | | Exercises | 12 weeks | (Ganesh et al., 2015) |  |  |  | 6.21 |
| SNAG | | CCFT | Single-session |  | (Shelke et al., 2023 |  |  | 6.43 |
| SNAG + CT+ exercises | | CT+ exercises | 2 weeks |  |  | 🗹 (Buyukturan et al., 2018; Shamsi et al., 2021) |  | 6.157 |
| MWM for the scapula | | CT + taping | 2 weeks |  |  | (Alshami & AlSadiq, 2021) |  | 6.158 |
| MWM for the scapula | | CT + taping | 3 weeks |  |  | (Alshami & AlSadiq, 2021) |  | 6.159 |
| SNAGs + Exercise | | Exercises | 4 weeks |  |  | 🗹 (Duymaz & Yagci, 2018; Rezkallah & Abdullah, 2018) |  | 6.160 |
| SNAGs + exercise | | Myofascial release + exercise | 4 weeks |  |  | **×** (Rezkallah & Abdullah, 2018) |  | 6.161 |
| Self-SNAGs + exercise/CCFT | | Cervicothoracic mobilization + exercise/CCFT | 6 weeks |  |  | 🗹 (Sun et al., 2024) |  | 6.162 |
| Self-SNAGs + exercise/CCFT | | Exercise/CCFT | 6 weeks |  |  | (Sun et al., 2024) |  | 6.163 |
| SNAGs | | MFR | 8 weeks |  |  | (Morsi et al., 2023) |  | 6.164 |
| SNAGs | | SNAGs + MFR | 8 weeks |  |  | 🗹 (Morsi et al., 2023 |  | 6.165 |
| SNAGs + MFR | | MFR | 8 weeks |  |  | 🗹 (Morsi et al., 2023) |  | 6.166 |
| SNAG + CT | | PRT + CT + exercise | 8 weeks |  |  | 🗹 (Mohamed & Elrazik, 2020) |  | 6.167 |
| SNAGs + CT | | CT | 2 weeks |  |  |  | (Aggarwal & Verma, 2018; Ozlu & Sahin, 2024) | 6.248 |
| Self-SNAG + CT | | CT | 2 weeks |  |  |  | (Aggarwal & Verma, 2018) | 6.249 |
| Self-SNAG + CT | | CT | 4 weeks |  |  |  | (Aggarwal & Verma, 2018) | 6.250 |
| SNAGs+NAGs+CT +ex | | CT+ex | 4 weeks |  |  |  | (Gautam et al., 2014) | 6.251 |
| SNAGs + MET + CT | | SNAGs + CT | 8 weeks |  |  |  | 🗹 (Sultan et al., 2021) | 6.252 |
| **Cervical range of motion – Rotation** | | | | | | | | |
| SNAGs | | HVLA | Single-session |  |  | (Izquierdo Perez et al., 2014; Lopez-Lopez et al., 2015) |  | 6.168 |
| SNAG | | Mobilization (PAIVMs) | Single-session |  |  | (Izquierdo Perez et al., 2014; Lopez-Lopez et al., 2015) |  | 6.169 |
| SNAGs + NAGs | | MET | 3 weeks |  |  | (Manzoor et al., 2021) |  | 6.170 |
| SNAGs | | HVLA | 4 weeks |  |  | (Izquierdo Perez et al., 2014) |  | 6.171 |
| SNAGs | | Mobilization (PAIVMs) | 4 weeks |  |  | (Izquierdo Perez et al., 2014) |  | 6.172 |
| SNAGS + exercise | | Exercise | 12 weeks |  |  | 🗹 (Duymaz & Yagci, 2018) |  | 6.173 |
| SNAGs | | HVLA | 12 weeks |  |  | (Izquierdo Perez et al., 2014) |  | 6.174 |
| SNAGs | | Mobilization (PAIVMs) | 12 weeks |  |  | (Izquierdo Perez et al., 2014) |  | 6.175 |
| **Cervical range of motion – Left rotation** | | | | | | | | |
| SNAG + exercises | | Mobilization (PAIVMs) and exercises | 2 weeks | (Ganesh et al., 2015) |  |  |  | 6.22 |
| SNAG + exercises | | Exercises | 2 weeks | (Ganesh et al., 2015) |  |  |  | 6.23 |
| SNAG | | MET + CT | 2 weeks | (Tank et al., 2018) |  |  |  | 6.24 |
| SNAG + exercises | | Mobilization (PAIVMs) and exercises | 12 weeks | (Ganesh et al., 2015) |  |  |  | 6.25 |
| SNAG + exercises | | Exercises | 12 weeks | (Ganesh et al., 2015) |  |  |  | 6.26 |
| SNAG | | CCFT | Single-session |  | (Shelke et al., 2023 |  |  | 6.44 |
| SNAGs + Interferential therapy + Isometric neck exercises | | Interferential therapy + Isometric neck exercises | 2 weeks |  | 🗹 (Vijayan et al., 2022) |  |  | 6.50 |
| SNAG + CT+ exercises | | CT+ exercises | 2 weeks |  |  | 🗹 (Buyukturan et al., 2018; Shamsi et al., 2021) |  | 6.176 |
| MWM for the scapula | | CT + taping | 2 weeks |  |  | (Alshami & AlSadiq, 2021) |  | 6.177 |
| MWM for the scapula | | CT + taping | 3 weeks |  |  | (Alshami & AlSadiq, 2021) |  | 6.178 |
| SNAGs + Exercise | | Exercises | 4 weeks |  |  | 🗹 (Duymaz & Yagci, 2018; Rezkallah & Abdullah, 2018) |  | 6.179 |
| SNAGs + exercise | | Myofascial release + exercise | 4 weeks |  |  | (Alshami & AlSadiq, 2021) |  | 6.180 |
| Self-SNAGs + exercise/CCFT | | Cervicothoracic mobilization + exercise/CCFT | 6 weeks |  |  | 🗹 (Sun et al., 2024) |  | 6.181 |
| Self-SNAGs + exercise/CCFT | | Exercise/CCFT | 6 weeks |  |  | (Sun et al., 2024) |  | 6.182 |
| SNAGs | | MFR | 8 weeks |  |  | (Morsi et al., 2023) |  | 6.183 |
| SNAGs | | SNAGs + MFR | 8 weeks |  |  | 🗹 (Morsi et al., 2023 |  | 6.184 |
| SNAGs + MFR | | MFR | 8 weeks |  |  | 🗹 (Morsi et al., 2023) |  | 6.185 |
| SNAG + CT | | PRT + CT + exercise | 8 weeks |  |  | 🗹 (Mohamed & Elrazik, 2020) |  | 6.186 |
| Self-SNAG+ CT | | CT | 2 weeks |  |  |  | (Aggarwal & Verma, 2018) | 6.253 |
| SNAGs + CT | | CT | 2 weeks |  |  |  | 🗹 (Aggarwal & Verma, 2018; Ozlu & Sahin, 2024) | 6.254 |
| Self-SNAG+ CT | | CT | 4 weeks |  |  |  | (Aggarwal & Verma, 2018) | 6.255 |
| SNAGs+NAGs+CT +ex | | CT+ex | 4 weeks |  |  |  | (Gautam et al., 2014) | 6.256 |
| SNAGs + MET + CT | | SNAGs + CT | 8 weeks |  |  |  | 🗹 (Sultan et al., 2021) | 6.257 |
| **Cervical range of motion – Right rotation** | | | | | | | | |
| SNAG + exercises | | Mobilization (PAIVMs) and exercises | 2 weeks | (Ganesh et al., 2015) |  |  |  | 6.27 |
| SNAG + exercises | | Exercises | 2 weeks | (Ganesh et al., 2015) |  |  |  | 6.28 |
| SNAG | | MET + CT | 2 weeks | (Tank et al., 2018) |  |  |  | 6.29 |
| SNAG + exercises | | Mobilization (PAIVMs) and exercises | 12 weeks | (Ganesh et al., 2015) |  |  |  | 6.30 |
| SNAG + exercises | | Exercises | 12 weeks | (Ganesh et al., 2015) |  |  |  | 6.31 |
| SNAG | | CCFT | Single-session |  | (Shelke et al., 2023 |  |  | 6.45 |
| SNAGs + Interferential therapy + Isometric neck exercises | | Interferential therapy + Isometric neck exercises | 2 weeks |  | 🗹 (Vijayan et al., 2022) |  |  | 6.49 |
| SNAG + CT+ exercises | | CT + exercises | 2 weeks |  |  | 🗹 (Buyukturan et al., 2018; Shamsi et al., 2021) |  | 6.187 |
| MWM for the scapula | | CT + taping | 2 weeks |  |  | (Alshami & AlSadiq, 2021) |  | 6.188 |
| MWM for the scapula | | CT + taping | 3 weeks |  |  | (Alshami & AlSadiq, 2021) |  | 6.189 |
| SNAGs + Exercise | | Exercises | 4 weeks |  |  | 🗹 (Duymaz & Yagci, 2018; Rezkallah & Abdullah, 2018) |  | 6.190 |
| SNAGs + exercise | | Myofascial release + exercise | 4 weeks |  |  | (Alshami & AlSadiq, 2021) |  | 6.191 |
| Self-SNAGs + exercise/CCFT | | Cervicothoracic mobilization + exercise/CCFT | 6 weeks |  |  | (Sun et al., 2024) |  | 6.192 |
| Self-SNAGs + exercise/CCFT | | Exercise/CCFT | 6 weeks |  |  | 🗹 (Sun et al., 2024) |  | 6.193 |
| SNAGs | | MFR | 8 weeks |  |  | (Morsi et al., 2023) |  | 6.194 |
| SNAGs | | SNAGs + MFR | 8 weeks |  |  | (Morsi et al., 2023 |  | 6.195 |
| SNAGs + MFR | | MFR | 8 weeks |  |  | 🗹 (Morsi et al., 2023) |  | 6.196 |
| SNAG + CT | | PRT + CT + exercise | 8 weeks |  |  | 🗹 (Mohamed & Elrazik, 2020) |  | 6.197 |
| Self-SNAG+ CT | CT | 2 weeks |  |  |  | (Aggarwal & Verma, 2018) | 6.258 |  |
| SNAGs + CT | CT | 2 weeks |  |  |  | 🗹 (Aggarwal & Verma, 2018; Ozlu & Sahin, 2024) | 6.259 |  |
| Self-SNAG+ CT | CT | 4 weeks |  |  |  | (Aggarwal & Verma, 2018) | 6.260 |  |
| SNAGs+NAGs+CT +ex | CT+ex | 4 weeks |  |  |  | 🗹 (Gautam et al., 2014) | 6.261 |  |
| SNAGs + MET + CT | SNAGs + CT | 8 weeks |  |  |  | 🗹 (Sultan et al., 2021) | 6.262 |  |
| **Disability** | | | | | | | | |
| SNAG + exercises | | Mobilization (PAIVMs) and exercises | 2 weeks | (Ganesh et al., 2015) |  |  |  | 6.32 |
| SNAG + exercises | | Exercises | 2 weeks | (Ganesh et al., 2015) |  |  |  | 6.33 |
| SNAG | | MET + CT | 2 weeks | (Tank et al., 2018) |  |  |  | 6.34 |
| SNAG + exercises | | Mobilization (PAIVMs) and exercises | 12 weeks | (Ganesh et al., 2015) |  |  |  | 6.35 |
| SNAG + exercises | | Exercises | 12 weeks | (Ganesh et al., 2015) |  |  |  | 6.36 |
| SNAG | | Mobilization (PAIVMs) | 3 weeks |  | (Alansari et al., 2021) |  |  | 6.38 |
| NAGS + CT | | Maitland + CT | 2 weeks |  | (Hussain et al., 2016) |  |  | 6.53 |
| NAGS + CT | | Maitland + CT | 4 weeks |  | (Hussain et al., 2016) |  |  | 6.54 |
| SNAGs | | HVLA | Single-session |  |  | (Izquierdo Perez et al., 2014; Lopez-Lopez et al., 2015) |  | 6.198 |
| SNAG | | Mobilization (PAIVMs) | Single-session |  |  | (Izquierdo Perez et al., 2014; Lopez-Lopez et al., 2015) |  | 6.199 |
| SNAG + CT+ exercises | | CT+ exercises | 2-4 weeks |  |  | (Shamsi et al., 2021; Tachii et al., 2015) |  | 6.200 |
|  |  |  |  |  |  | 🗹(Abd El-Azeim & Grase, 2023; Buyukturan et al., 2018; Shamsi et al., 2021; Tachii et al., 2015)  ***sensitivity analysis** |  |  |
| MWM for the scapula | | CT + taping | 2 weeks |  |  | (Alshami & AlSadiq, 2021) |  | 6.204 |
| MWM for the scapula | | CT + taping | 3 weeks |  |  | (Alshami & AlSadiq, 2021) |  | 6.205 |
| SNAGs + NAGs | | MET | 3 weeks |  |  | 🗹 (Manzoor et al., 2021) |  | 6.201 |
| SNAG+NAG+self-SNAG | | Sham | 3 weeks |  |  | 🗹 (Zemadanis, 2018) |  | 6.202 |
| SNAGs + exercises | | Exercises | 2-4 weeks |  |  | 🗹  (Duymaz & Yagci, 2018; Rezkallah & Abdullah, 2018) |  | 6.203 |
| SNAGs + exercise | | Myofascial release + exercise | 4 weeks |  |  | 🗹(Rezkallah & Abdullah, 2018) |  | 6.206 |
| SNAGs | | HVLA | 4 weeks |  |  | (Izquierdo Perez et al., 2014) |  | 6.207 |
| SNAG | | Mobilization (PAIVMs) | 4 weeks |  |  | (Izquierdo Perez et al., 2014) |  | 6.208 |
| Self/SNAGs + CT | | CT | 4 weeks |  |  | 🗹 (Said et al., 2017) |  | 6.209 |
| SNAGs + CT | | CT | 4 weeks |  |  | 🗹 (Said et al., 2017) |  | 6.210 |
| SNAG+NAG+self-SNAG | | Sham | 4 weeks |  |  | 🗹 (Zemadanis, 2018) |  | 6.211 |
| Self-SNAGs + exercise/CCFT | | Cervicothoracic mobilization + exercise/CCFT | 6 weeks |  |  | (Sun et al., 2024) |  | 6.213 |
| Self-SNAGs + exercise/CCFT | | Exercise/CCFT | 6 weeks |  |  | (Sun et al., 2024) |  | 6.214 |
| SNAG + CT | | PRT + CT + exercise | 8 weeks |  |  | 🗹 (Mohamed & Elrazik, 2020) |  | 6.215 |
| SNAGs | | MFR | 8 weeks |  |  | (Morsi et al., 2023) |  | 6.216 |
| SNAGs | | SNAGs + MFR | 8 weeks |  |  | 🗹 (Morsi et al., 2023 |  | 6.217 |
| SNAGs + MFR | | MFR | 8 weeks |  |  | 🗹 (Morsi et al., 2023) |  | 6.218 |
| SNAGs | | HVLA | 8 weeks |  |  | (Izquierdo Perez et al., 2014) |  | 6.219 |
| SNAG | | Mobilization (PAIVMs) | 8 weeks |  |  | (Izquierdo Perez et al., 2014; Lopez-Lopez et al., 2015) |  | 6.220 |
| SNAGs | | HVLA | 12 weeks |  |  | (Izquierdo Perez et al., 2014) |  | 6.221 |
| SNAG | | Mobilization (PAIVMs) | 8 weeks |  |  | (Izquierdo Perez et al., 2014; Lopez-Lopez et al., 2015) |  | 6.222 |
| SNAGs + CT | | CT | 2 weeks |  |  |  | 🗹 (Aggarwal & Verma, 2018; Ozlu & Sahin, 2024) | 6.263 |
| Self-SNAG + CT | | CT | 2 weeks |  |  |  | (Aggarwal & Verma, 2018) | 6.264 |
| NAGs | | Muscle energy technique (post-isometric relaxation) | 4 weeks |  |  |  | 🗹 (Usama et al., 2022) | 6.265 |
| SNAG + ex | | Maitland + ex | 4 weeks |  |  |  | 🗹 (Tanveer et al., 2017) | 6.267 |
| SNAG + ex | | Exercise | 4 weeks |  |  |  | (Tanveer et al., 2017) | 6.268 |
| Mulligan + CT + ex | | Maitland + CT + ex | 4 weeks |  |  |  | (Shehri et al., 2018) | 6.269 |

CCFT: neck motor control training using the craniocervical flexion test;

CT: conventional therapy;

ex: exercise;

HVLA: high velocity low amplitude;

MET: muscle energy techniques;

MRF: myofascial release;

NAGs: Natural Apophyseal Glides;

NAGs: Natural Apophyseal Glides;

PAIVMs: passive accessory intervertebral movements;

PRT: positional release therapy;

SNAG: Sustained Natural Apophyseal Glides;

: positive effect favoring therapy of interest; : positive effect favoring comparator; : no differences between therapies were identified; : not investigated.

🗹: potentially clinically relevant based on the SMD;

🗹: potentially clinically relevant based on the MD;

🗹: clinically relevant based on SMD and MD;

**×**: not clinically relevant;

*# some comparisons reported in GRADE are not presented in the matrix due the lack of information provided by the primary study ((El-Sodany et al., 2014; Ali et al., 2014 and Kumar et al., 2011)*
