# Supplementary material for: The Effectiveness of Mulligan's Techniques in Non‐Specific Neck Pain: A Systematic Review and Meta‐Analysis
Source: Physiother Res Int. 2025 May 29;30(3):e70045. doi: 10.1002/pri.70045 (PMC12121345; doi:10.1002/pri.70045)
Supplement: Supplementary file 8 — Supporting Information S8 [file PRI-30-e70045-s002.pdf]

Study or Subgroup	SNAGs+CT+Ex Mean	SNAGs+CT+Ex SD	SNAGs+CT+Ex Total	CT+Ex Mean	CT+Ex SD	CT+Ex Total	Weight	Std. Mean Difference IV, Random, 95% CI
**8.1.1 Pain Intensity (VAS/NPRS) at 2 weeks**								
Buyukturan, 2018	4	3.18	21	4	2.4	19	14.4%	0.00 [-0.62, 0.62]
Shamsi, 2021	0.44	0.6	50	0.68	0.85	50	14.9%	-0.32 [-0.72, 0.07]
Tachii, 2015	3.43	0.51	15	4.07	1.21	15	14.0%	-0.67 [-1.41, 0.07]
**Subtotal (95% CI)**			**86**			**84**	**43.2%**	**-0.30 [-0.61, 0.00]**
Heterogeneity: Tau² = 0.00; Chi² = 1.88, df = 2 (P = 0.39); I² = 0%								
Test for overall effect: Z = 1.97 (P = 0.05)								
**8.1.2 Pain Intensity (VAS/NPRS) at 2/4weeks**								
Buyukturan, 2018	4	3.18	21	4	2.4	19	14.4%	0.00 [-0.62, 0.62]
El-Azeim, 2023	1.6	0.25	45	5.7	1.08	45	13.5%	-5.19 [-6.06, -4.31]
Shamsi, 2021	0.44	0.6	50	0.68	0.85	50	14.9%	-0.32 [-0.72, 0.07]
Tachii, 2015	3.43	0.51	15	4.07	1.21	15	14.0%	-0.67 [-1.41, 0.07]
**Subtotal (95% CI)**			**131**			**129**	**56.8%**	**-1.52 [-3.38, 0.35]**
Heterogeneity: Tau² = 3.51; Chi² = 107.91, df = 3 (P < 0.00001); I² = 97%								
Test for overall effect: Z = 1.59 (P = 0.11)								

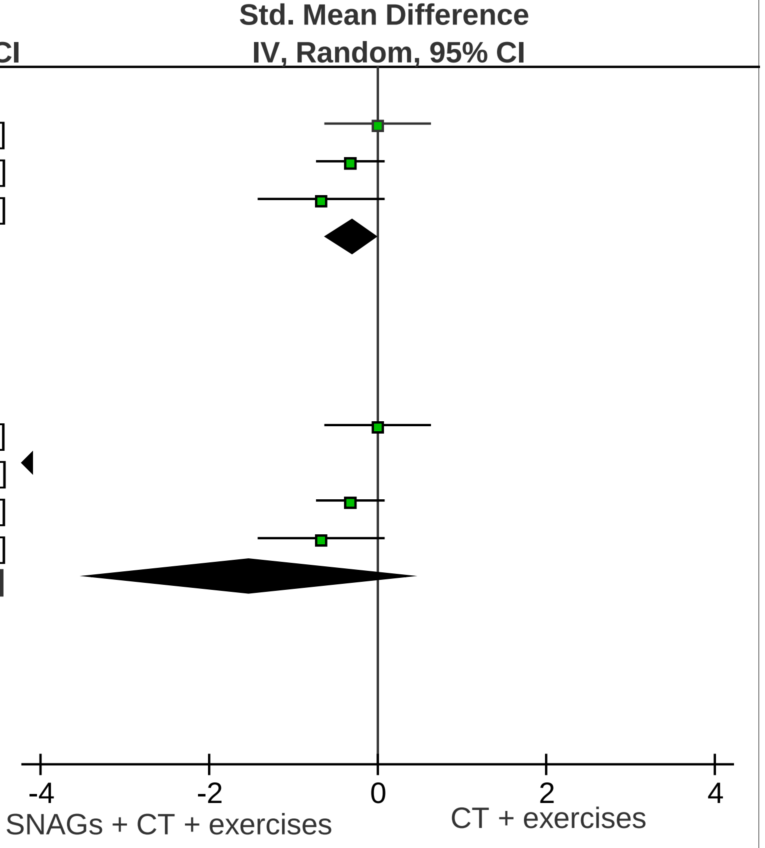

Figure 8.1 Pain intensity comparing SNAG + Conventional Therapy + Exercise vs. Conventional Therapy + Exercise at 2-4 weeks (end of the treatment) in patients with chronic neck pain.

All analyses are described as standardized mean differences (SMD) with random effects.

The effect sizes for comparisons were calculated using post treatment means.

Lower absolute values mean reduction in pain intensity after treatment.

SNAGs: Sustained Natural Apophyseal Glides; CT: conventional therapy; Ex: exercise

	SNAG + exercises			Exercises				Std. Mean Difference
**Study or Subgroup**	**Mean**	**SD**	**Total**	**Mean**	**SD**	**Total**	**Weight**	**IV, Random, 95% CI**
Duymaz, 2018	14.65	12.69	20	57.95	17.44	20	42.9%	-2.78 [-3.68, -1.89]
Rezkallah, 2018	2.69	0.97	25	5.14	1.35	22	57.1%	-2.07 [-2.79, -1.35]
**Total (95% CI)**			**45**			**42**	**100.0%**	**-2.38 [-3.07, -1.69]**

Heterogeneity: $Tau^2 = 0.08$; $Chi^2 = 1.48$, df = 1 (P = 0.22); $I^2 = 32\%$
Test for overall effect: Z = 6.75 (P < 0.00001)

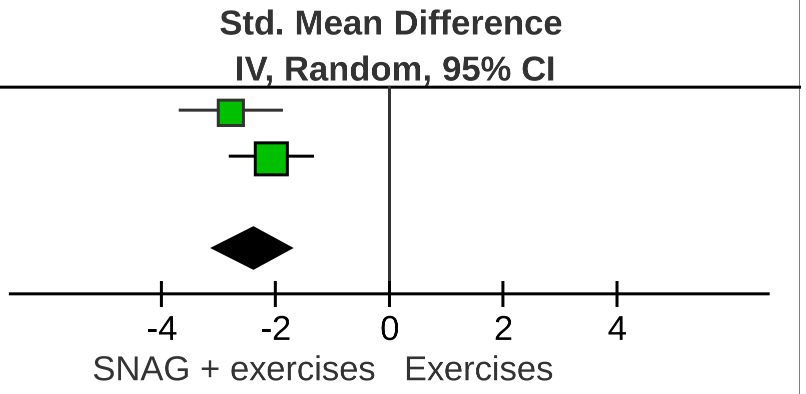

Figure 8.2 Pain intensity comparing SNAG + Exercises vs. Exercises at 2-4 weeks (end of the treatment) in patients with chronic neck pain.

All analyses are described as standardized mean differences (SMD) with random effects.

The effect sizes for comparisons were calculated using post treatment means.

Lower absolute values mean reduction in pain intensity after treatment.

SNAGs: Sustained Natural Apophyseal Glides;

	SNAGs+CT+Ex			CT+Ex				Std. Mean Difference
Study or Subgroup	Mean	SD	Total	Mean	SD	Total	Weight	IV, Random, 95% CI
Buyukturan, 2018	44.7	5.32	21	41.16	3.28	19	49.3%	0.78 [0.13, 1.42]
Shamsi, 2021	43.64	3.95	50	34.02	3.88	50	50.7%	2.44 [1.92, 2.96]
**Total (95% CI)**			**71**			**69**	**100.0%**	**1.62 [-0.01, 3.25]**

Heterogeneity: Tau² = 1.29; Chi² = 15.39, df = 1 (P < 0.0001); I² = 94%

Test for overall effect: Z = 1.95 (P = 0.05)

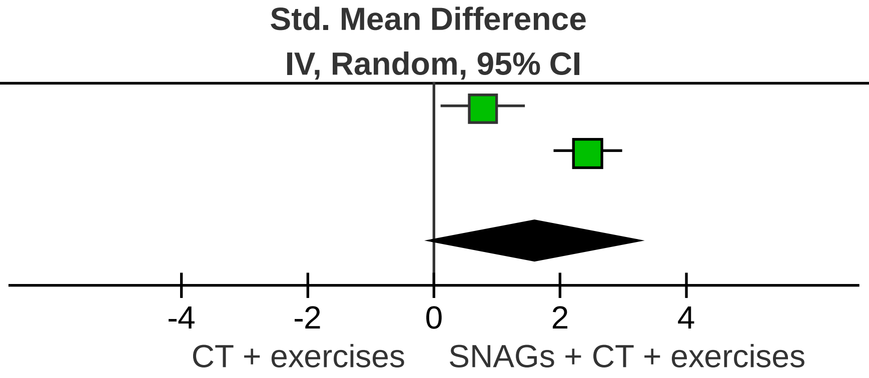

Figure 8.3 Neck flexion comparing SNAG + Conventional Therapy + Exercise vs. Conventional Therapy + Exercise at 2-4 weeks (end of the treatment) in patients with chronic neck pain.

All analyses are described as standardized mean differences with random effects.

The effect sizes for comparisons were calculated using post treatment means.

Higher absolute values mean cervical range of motion improvement after treatment.

SNAGs: Sustained Natural Apophyseal Glides; CT: Conventional therapy; Ex: exercise

Study or Subgroup	SNAG + exercises Mean	SD	Total	Exercises Mean	SD	Total	Weight	Std. Mean Difference IV, Random, 95% CI
Duymaz, 2018	49.6	1.98	20	43.2	7.4	20	51.1%	1.16 [0.48, 1.83]
Rezkallah, 2018	50.56	2.8	25	44.71	2.05	22	48.9%	2.32 [1.57, 3.07]
**Total (95% CI)**			**45**			**42**	**100.0%**	**1.73 [0.59, 2.87]**

Heterogeneity: $Tau^2 = 0.54$; $Chi^2 = 5.07$, df = 1 (P = 0.02); $I^2 = 80\%$

Test for overall effect: Z = 2.97 (P = 0.003)

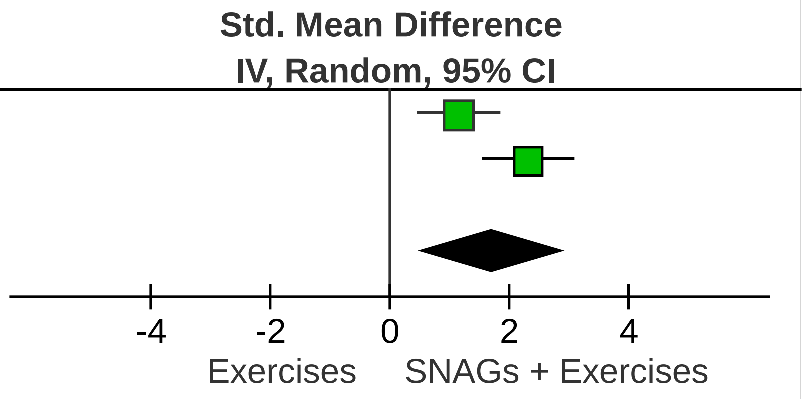

Figure 8.4 Neck flexion comparing SNAG + Exercise vs. Exercise at 2-4 weeks (end of the treatment) in patients with chronic neck pain.

All analyses are described as standardized mean differences (SMD) with random effects.

The effect sizes for comparisons were calculated using post treatment means.

Higher absolute values mean cervical range of motion improvement after treatment.

SNAGs: Sustained Natural Apophyseal Glides; Ex: exercise

	SNAGs+CT+Ex			CT+Ex				Std. Mean Difference
Study or Subgroup	Mean	SD	Total	Mean	SD	Total	Weight	IV, Random, 95% CI
Buyukturan, 2018	41.2	6.2	21	39.2	5.52	19	49.7%	0.33 [-0.29, 0.96]
Shamsi, 2021	42.9	4.42	50	33.32	3.02	50	50.3%	2.51 [1.98, 3.04]
**Total (95% CI)**			**71**			**69**	**100.0%**	**1.43 [-0.71, 3.56]**

Heterogeneity: $Tau^2 = 2.29$; $Chi^2 = 27.18$, df = 1 ($P < 0.00001$); $I^2 = 96\%$
Test for overall effect: Z = 1.31 (P = 0.19)

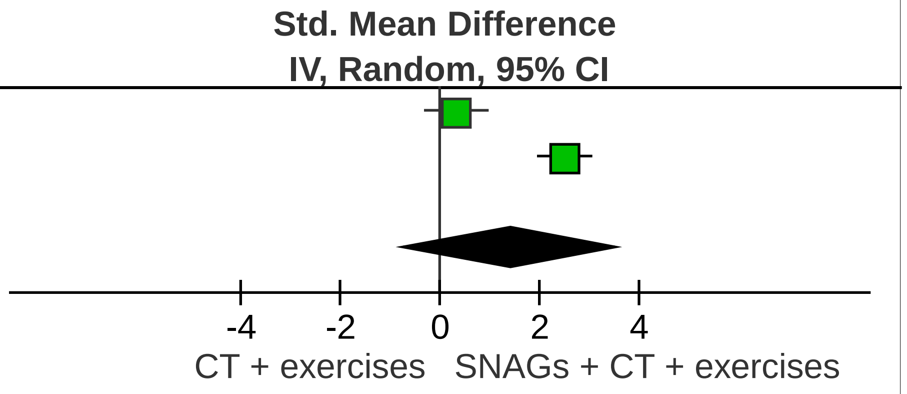

Figure 8.5 Neck extension comparing SNAG + Conventional Therapy + Exercise vs. Conventional Therapy + Exercise at 2-4 weeks (end of the treatment) in patients with chronic neck pain.
All analyses are described as standardized mean differences with random effects.
The effect sizes for comparisons were calculated using post treatment means.
Higher absolute values mean cervical range of motion improvement after treatment.
SNAGs: Sustained Natural Apophyseal Glides; CT: Conventional therapy; Ex: exercise

	SNAG + exercises			Exercises				Std. Mean Difference
**Study or Subgroup**	**Mean**	**SD**	**Total**	**Mean**	**SD**	**Total**	**Weight**	**IV, Random, 95% CI**
Duymaz, 2018	38.8	2.69	20	34.87	4.37	20	50.9%	1.06 [0.40, 1.73]
Rezkallah, 2018	69.17	2.28	25	55.42	3.57	22	49.1%	4.58 [3.45, 5.70]
**Total (95% CI)**			**45**			**42**	**100.0%**	**2.79 [-0.66, 6.24]**

Heterogeneity: $Tau^2 = 5.96$; $Chi^2 = 27.82$, df = 1 ($P < 0.00001$); $I^2 = 96\%$
Test for overall effect: Z = 1.59 (P = 0.11)

Std. Mean Difference
IV, Random, 95% CI

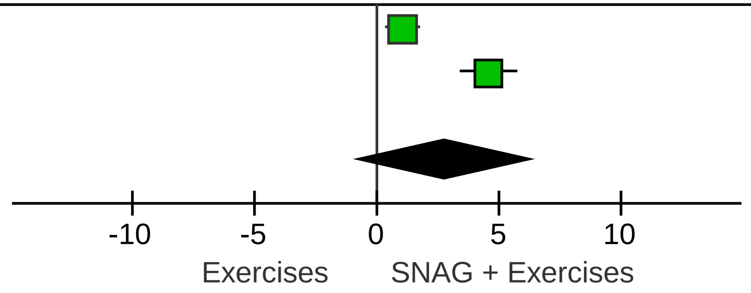

Figure 8.6 Neck extension comparing SNAG + Exercise vs. Exercise at 2-4 weeks (end of the treatment) in patients with chronic neck pain.
All analyses are described as standardized mean differences (SMD) with random effects.
The effect sizes for comparisons were calculated using post treatment means.
Higher absolute values mean cervical range of motion improvement after treatment.
SNAGs: Sustained Natural Apophyseal Glides; Ex: exercise

Study or Subgroup	SNAG+CT+Ex Mean	SNAG+CT+Ex SD	SNAG+CT+Ex Total	CT+Ex Mean	CT+Ex SD	CT+Ex Total	Weight	Std. Mean Difference IV, Random, 95% CI
Buyukturan, 2018	41.36	5.8	21	37.03	4.08	19	30.1%	0.84 [0.19, 1.49]
Shamsi, 2021	36.48	2.79	50	32.54	3.68	50	69.9%	1.20 [0.77, 1.62]
**Total (95% CI)**			**71**			**69**	**100.0%**	**1.09 [0.73, 1.45]**

Heterogeneity: $Tau^2 = 0.00$; $Chi^2 = 0.82$, df = 1 (P = 0.37); $I^2 = 0\%$
Test for overall effect: Z = 5.98 (P < 0.00001)

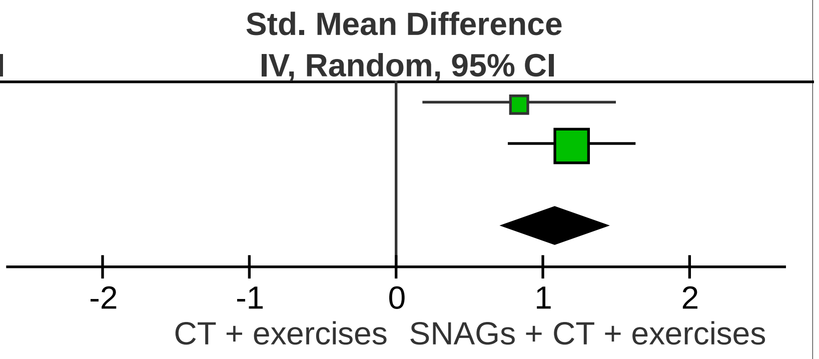

Figure 8.7 Left lateral flexion comparing SNAG + Conventional Therapy + Exercise vs. Conventional Therapy + Exercise at 2-4 weeks (end of the treatment) in patients with chronic neck pain.
All analyses are described as standardized mean differences with random effects.
The effect sizes for comparisons were calculated using post treatment means.
Higher absolute values mean cervical range of motion improvement after treatment.
SNAGs: Sustained Natural Apophyseal Glides; CT: Conventional therapy; Ex: exercise

	SNAG+CT+Ex			CT+Ex				Std. Mean Difference
Study or Subgroup	Mean	SD	Total	Mean	SD	Total	Weight	IV, Random, 95% CI
Buyukturan, 2018	43.56	6.59	21	37.8	3.2	19	27.3%	1.07 [0.40, 1.74]
Shamsi, 2021	36.46	2.53	50	33.66	3.97	50	72.7%	0.83 [0.43, 1.24]
**Total (95% CI)**			**71**			**69**	**100.0%**	**0.90 [0.55, 1.25]**

Heterogeneity: $Tau^2 = 0.00$; $Chi^2 = 0.35$, df = 1 (P = 0.55); $I^2 = 0\%$
Test for overall effect: Z = 5.05 (P < 0.00001)

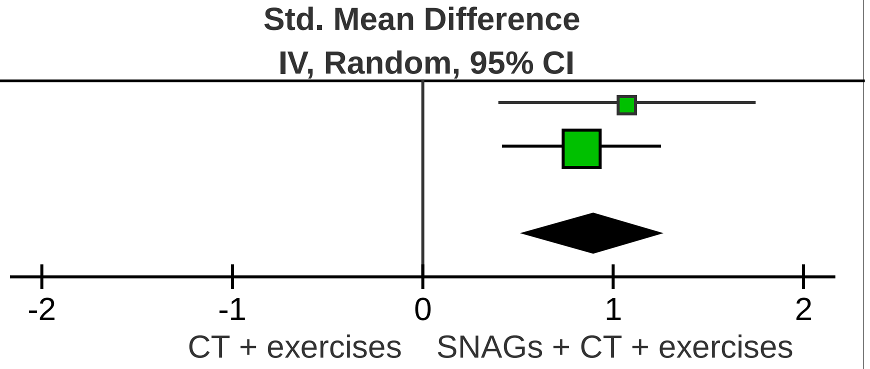

Figure 8.8 Right lateral flexion comparing SNAG + Conventional Therapy + Exercise vs. Conventional Therapy + Exercise at 2-4 weeks (end of the treatment) in patients with chronic neck pain.
All analyses are described as standardized mean differences with random effects.
The effect sizes for comparisons were calculated using post treatment means.
Higher absolute values mean cervical range of motion improvement after treatment.
SNAGs: Sustained Natural Apophyseal Glides; CT: Conventional therapy; Ex: exercise

	SNAG+CT+Ex			CT+Ex				Std. Mean Difference
**Study or Subgroup**	**Mean**	**SD**	**Total**	**Mean**	**SD**	**Total**	**Weight**	**IV, Random, 95% CI**
Buyukturan, 2018	48.63	5.48	21	41.7	3.86	19	44.3%	1.42 [0.72, 2.12]
Shamsi, 2021	66.1	4.91	50	53.94	6.14	50	55.7%	2.17 [1.67, 2.67]
**Total (95% CI)**			**71**			**69**	**100.0%**	**1.84 [1.11, 2.57]**

Heterogeneity: $Tau^2 = 0.18$; $Chi^2 = 2.92$, df = 1 (P = 0.09); $I^2 = 66\%$
Test for overall effect: Z = 4.93 (P < 0.00001)

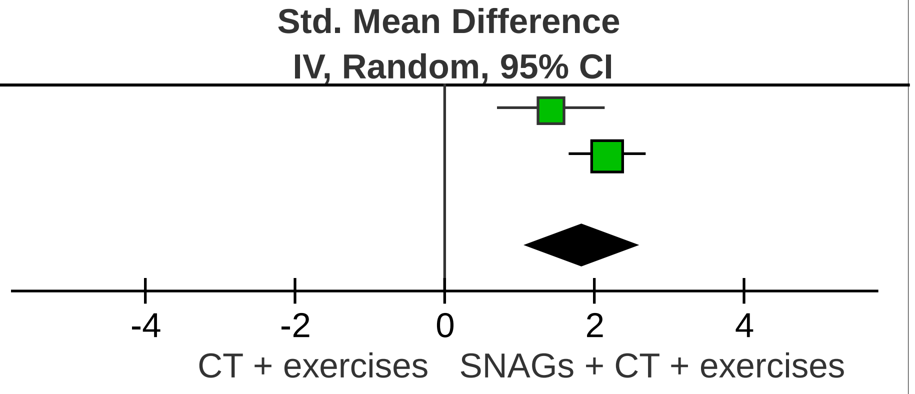

Figure 8.9 Left rotation comparing SNAG + Conventional Therapy + Exercise vs. Conventional Therapy + Exercise at 2-4 weeks (end of the treatment) in patients with chronic neck pain.
All analyses are described as standardized mean differences with random effects.
The effect sizes for comparisons were calculated using post treatment means.
Higher absolute values mean cervical range of motion improvement after treatment.
SNAGs: Sustained Natural Apophyseal Glides; CT: Conventional therapy; Ex: exercise

Study or Subgroup	SNAG+CT+Ex Mean	SNAG+CT+Ex SD	SNAG+CT+Ex Total	CT+Ex Mean	CT+Ex SD	CT+Ex Total	Weight	Std. Mean Difference IV, Random, 95% CI
Buyukturan, 2018	50.4	6.2	21	43.27	3.83	19	29.7%	1.34 [0.65, 2.03]
Shamsi, 2021	64.92	5.21	50	55.03	7.07	50	70.3%	1.58 [1.13, 2.03]
**Total (95% CI)**			**71**			**69**	**100.0%**	**1.51 [1.13, 1.89]**

Heterogeneity: $Tau^2 = 0.00$; $Chi^2 = 0.32$, $df = 1$ ($P = 0.57$); $I^2 = 0\%$
Test for overall effect: $Z = 7.82$ ($P < 0.00001$)

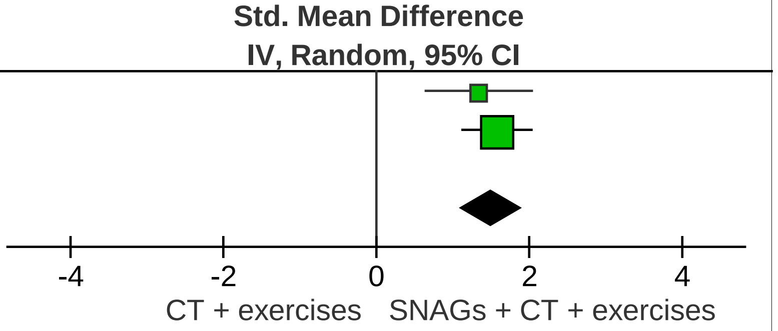

Figure 8.10 Right rotation comparing SNAG + Conventional Therapy + Exercise vs. Conventional Therapy + Exercise at 2-4 weeks (end of the treatment) in patients with chronic neck pain.
All analyses are described as standardized mean differences with random effects.
The effect sizes for comparisons were calculated using post treatment means.
Higher absolute values mean cervical range of motion improvement after treatment.
SNAGs: Sustained Natural Apophyseal Glides; CT: Conventional therapy; Ex: exercise

	SNAG+CT+Ex			CT+Ex				Std. Mean Difference
Study or Subgroup	Mean	SD	Total	Mean	SD	Total	Weight	IV, Random, 95% CI
**8.11.1 NDI at 2 weeks**								
Buyukturan, 2018	5	1.59	21	6.33	3.2	19	14.2%	-0.52 [-1.16, 0.11]
Shamsi, 2021	14.48	3.68	50	18.3	6.23	50	15.2%	-0.74 [-1.15, -0.34]
Tachii, 2015	13.07	4.03	15	16.14	4.91	15	13.6%	-0.67 [-1.40, 0.07]
**Subtotal (95% CI)**			**86**			**84**	**43.0%**	**-0.68 [-0.99, -0.37]**
Heterogeneity: Tau² = 0.00; Chi² = 0.32, df = 2 (P = 0.85); I² = 0%								
Test for overall effect: Z = 4.27 (P < 0.0001)								
**8.11.2. NDI at 2-4 weeks**								
Buyukturan, 2018	5	1.59	21	6.33	3.2	19	14.2%	-0.52 [-1.16, 0.11]
El-Azeim, 2023	9.55	1.53	45	16	2.1	45	14.0%	-3.48 [-4.14, -2.82]
Shamsi, 2021	14.48	3.68	50	18.3	6.23	50	15.2%	-0.74 [-1.15, -0.34]
Tachii, 2015	13.07	4.03	15	16.14	4.91	15	13.6%	-0.67 [-1.40, 0.07]
**Subtotal (95% CI)**			**131**			**129**	**57.0%**	**-1.35 [-2.63, -0.06]**
Heterogeneity: Tau² = 1.62; Chi² = 56.59, df = 3 (P < 0.00001); I² = 95%								
Test for overall effect: Z = 2.05 (P = 0.04)								

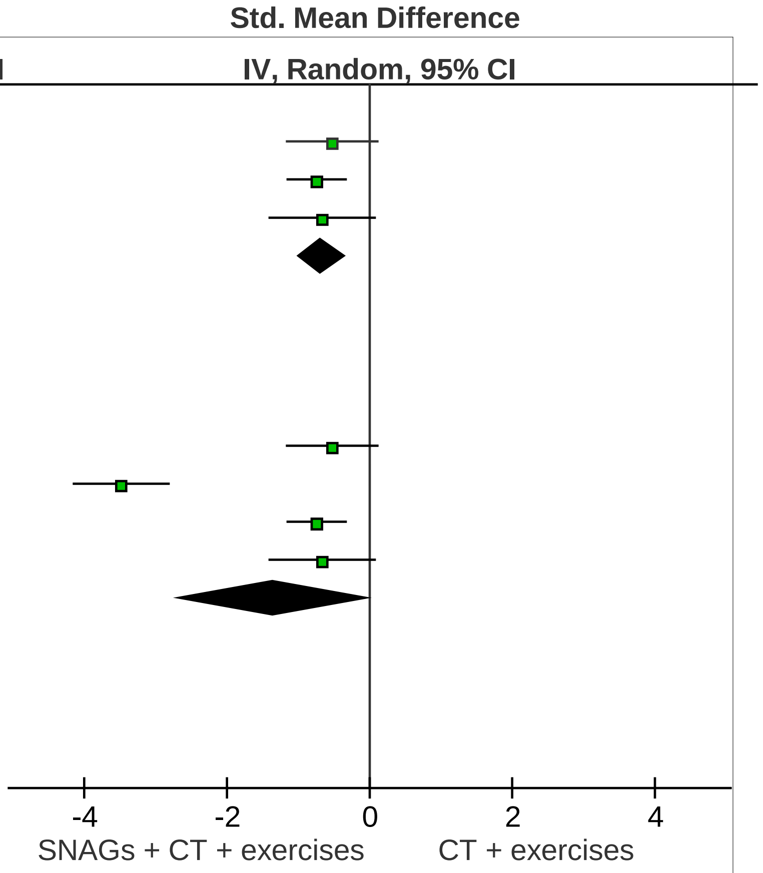

Figure 8.11 Neck disability comparing SNAG + Conventional Therapy + Exercise vs. Conventional Therapy + Exercise at 2/4 weeks (end of the treatment) in patients with chronic neck pain.

All analyses are described as standardized mean differences with random effects.

The effect sizes for comparisons were calculated using post treatment means.

Lower absolute values mean improvement on disability after treatment.

SNAGs: Sustained Natural Apophyseal Glides; CT: conventional therapy; Ex: exercise

	SNAG + Exercises			Exercises				Mean Difference
Study or Subgroup	Mean	SD	Total	Mean	SD	Total	Weight	IV, Random, 95% CI
Duymaz, 2018	2.9	3.12	20	11.5	5.18	20	12.1%	-8.60 [-11.25, -5.95]
Rezkallah, 2018	5.86	1.25	25	14.52	2.04	22	87.9%	-8.66 [-9.64, -7.68]
**Total (95% CI)**			**45**			**42**	**100.0%**	**-8.65 [-9.57, -7.73]**

Heterogeneity: Tau² = 0.00; Chi² = 0.00, df = 1 (P = 0.97); I² = 0%
Test for overall effect: Z = 18.40 (P < 0.00001)

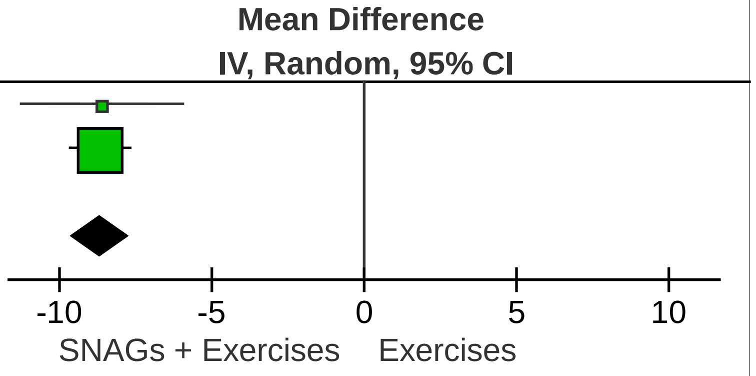

Figure 8.12 Neck disability comparing SNAG + Exercise vs. Exercises at 2/4 weeks (end of the treatment) in patients with chronic neck pain.

All analyses are described as standardized mean differences with random effects.
The effect sizes for comparisons were calculated using post treatment means.
Lower absolute values mean improvement on disability after treatment.
SNAGs: Sustained Natural Apophyseal Glides; CT: conventional therapy; Ex: exercise

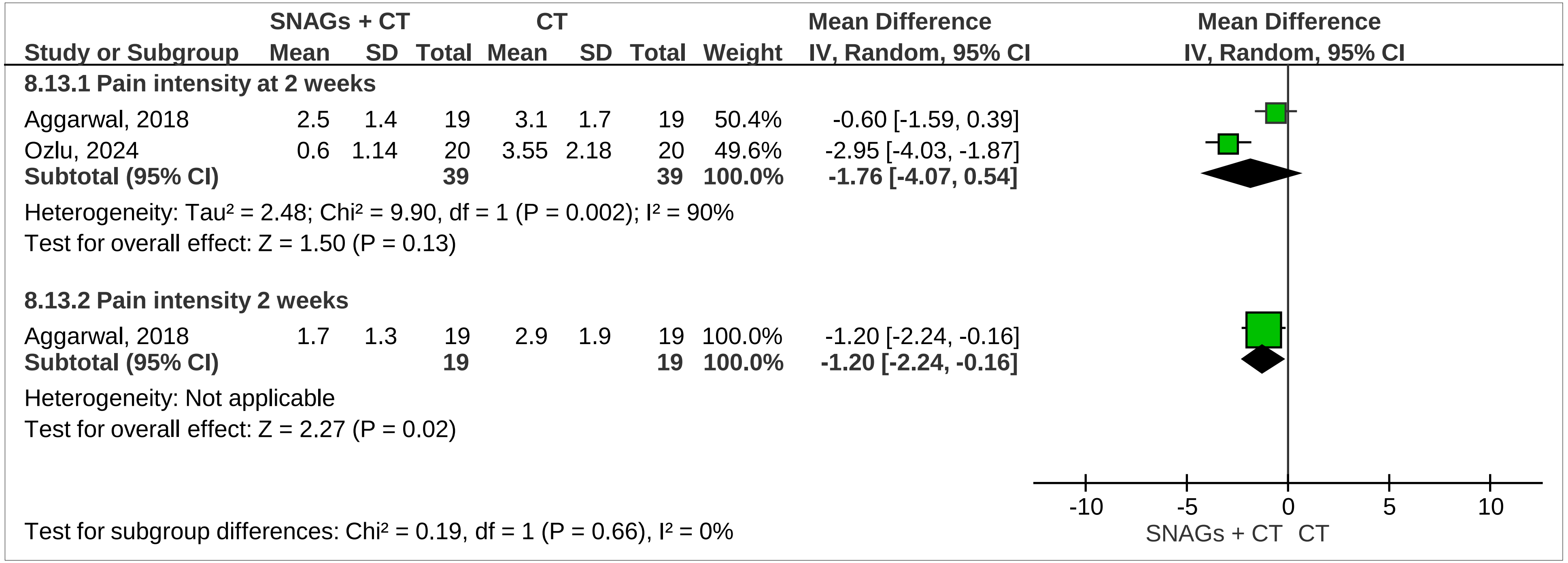

Figure 8.13 Pain intensity comparing SNAG + CT vs. CT at 2 weeks (end of the treatment), non-specified chronicity.

All analyses are described as standardized mean differences with random effects.

The effect sizes for comparisons were calculated using post treatment means.

Lower absolute values mean improvement on pain after treatment.

SNAGs: Sustained Natural Apophyseal Glides; CT: conventional therapy

Study or Subgroup	SNAG+CT Mean	SNAG+CT SD	SNAG+CT Total	CT Mean	CT SD	CT Total	Weight	Mean Difference IV, Random, 95% CI
**8.14.1 2 weeks**								
Aggarwal, 2018	60.8	8.5	19	63.3	6.7	19	53.9%	-2.50 [-7.37, 2.37]
Ozlu, 2024	60.85	15.66	20	49.35	13.59	20	46.1%	11.50 [2.41, 20.59]
**Subtotal (95% CI)**			**39**			**39**	**100.0%**	**3.95 [-9.73, 17.63]**
Heterogeneity: Tau² = 84.17; Chi² = 7.09, df = 1 (P = 0.008); I² = 86%								
Test for overall effect: Z = 0.57 (P = 0.57)								
**8.14.2 4 weeks**								
Aggarwal, 2018	61.2	8.3	19	63.4	6.7	19	100.0%	-2.20 [-7.00, 2.60]
**Subtotal (95% CI)**			**19**			**19**	**100.0%**	**-2.20 [-7.00, 2.60]**
Heterogeneity: Not applicable								
Test for overall effect: Z = 0.90 (P = 0.37)								
Test for subgroup differences: Chi² = 0.69, df = 1 (P = 0.41), I² = 0%								

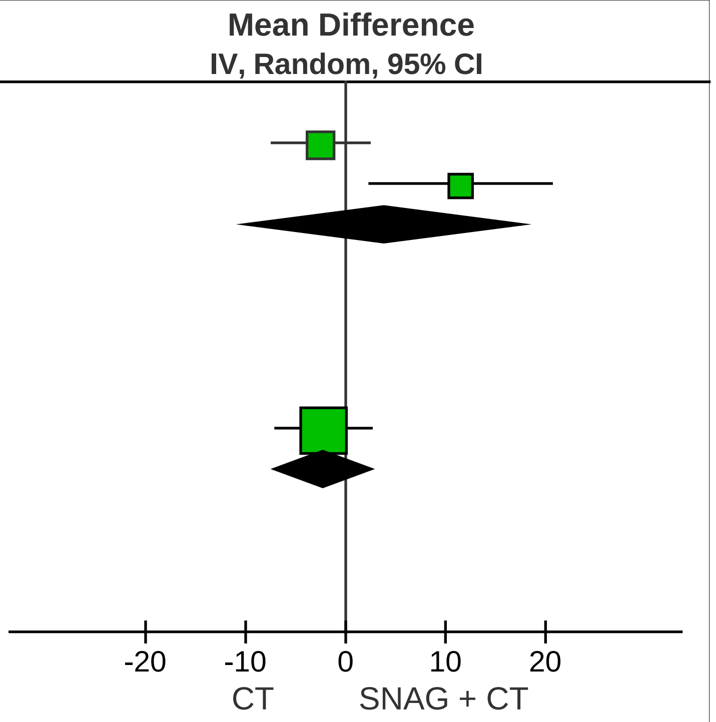

Figure 8.14 Extension comparing SNAG + Conventional Therapy vs. Conventional Therapy at 2/4 weeks (end of the treatment), non-specified chronicity.
All analyses are described as mean differences in degrees with random effects.
The effect sizes for comparisons were calculated using post treatment means.
Higher absolute values mean cervical range of motion improvement after treatment.
SNAGs: Sustained Natural Apophyseal Glides; CT: conventional therapy; Ex: exercise

Study or Subgroup	SNAG+CT Mean	SNAG+CT SD	SNAG+CT Total	CT Mean	CT SD	CT Total	Weight	Mean Difference IV, Random, 95% CI
**8.15.1 2 weeks**								
Aggarwal, 2018	38	6.4	19	37	6.7	19	51.1%	1.00 [-3.17, 5.17]
Ozlu, 2024	32.85	7.13	20	22.65	8.7	20	48.9%	10.20 [5.27, 15.13]
**Subtotal (95% CI)**			**39**			**39**	**100.0%**	**5.50 [-3.51, 14.52]**
Heterogeneity: Tau² = 36.90; Chi² = 7.80, df = 1 (P = 0.005); I² = 87%								
Test for overall effect: Z = 1.20 (P = 0.23)								
**8.15.2 4 weeks**								
Aggarwal, 2018	38.3	6.7	19	37.3	6.7	19	100.0%	1.00 [-3.26, 5.26]
**Subtotal (95% CI)**			**19**			**19**	**100.0%**	**1.00 [-3.26, 5.26]**
Heterogeneity: Not applicable								
Test for overall effect: Z = 0.46 (P = 0.65)								
Test for subgroup differences: Chi² = 0.78, df = 1 (P = 0.38), I² = 0%								

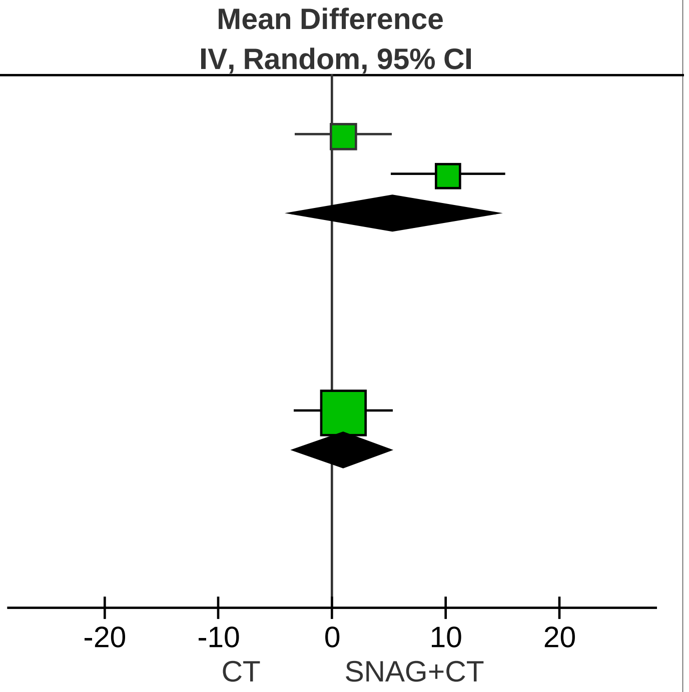

Figure 8.15 Left lateral flexion comparing SNAG + Conventional Therapy vs. Conventional Therapy at 2/4 weeks (end of the treatment), non-specified chronicity.

All analyses are described as mean differences in degrees with random effects.

The effect sizes for comparisons were calculated using post treatment means.

Higher absolute values mean cervical range of motion improvement after treatment.

SNAGs: Sustained Natural Apophyseal Glides; CT: conventional therapy; Ex: exercise

Study or Subgroup	SNAG+CT Mean	SNAG+CT SD	SNAG+CT Total	CT Mean	CT SD	CT Total	Weight	Mean Difference IV, Random, 95% CI
**8.16.1 2 weeks**								
Aggarwal, 2018	37.2	6.4	19	36.7	6.5	19	35.8%	0.50 [-3.60, 4.60]
Ozlu, 2024	31.1	6.19	20	23.25	10.42	20	29.2%	7.85 [2.54, 13.16]
**Subtotal (95% CI)**			**39**			**39**	**65.0%**	**3.97 [-3.22, 11.17]**
Heterogeneity: Tau² = 21.15; Chi² = 4.61, df = 1 (P = 0.03); I² = 78%								
Test for overall effect: Z = 1.08 (P = 0.28)								
**8.16.2 4 weeks**								
Aggarwal, 2018	37.6	6.8	19	36.7	6.5	19	35.0%	0.90 [-3.33, 5.13]
**Subtotal (95% CI)**			**19**			**19**	**35.0%**	**0.90 [-3.33, 5.13]**
Heterogeneity: Not applicable								
Test for overall effect: Z = 0.42 (P = 0.68)								

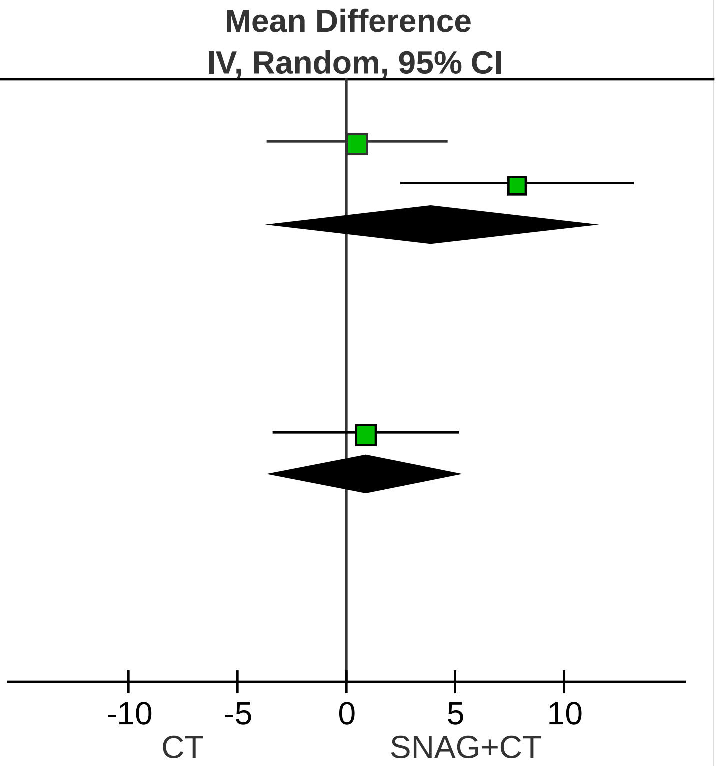

Figure 8.16 Right lateral flexion comparing SNAG + Conventional Therapy vs. Conventional Therapy at 2/4 weeks (end of the treatment), non-specified chronicity.

All analyses are described as mean differences in degrees with random effects.

The effect sizes for comparisons were calculated using post treatment means.

Higher absolute values mean cervical range of motion improvement after treatment.

SNAGs: Sustained Natural Apophyseal Glides; CT: conventional therapy; Ex: exercise

Study or Subgroup	SNAG+CT Mean	SNAG+CT SD	SNAG+CT Total	CT Mean	CT SD	CT Total	Weight	Mean Difference IV, Random, 95% CI
**8.17.1 2 weeks**								
Aggarwal, 2018	69.9	9.2	19	64.6	9.8	19	49.0%	5.30 [-0.74, 11.34]
Ozlu, 2024	79.1	5.99	20	54.6	1.36	20	51.0%	24.50 [21.81, 27.19]
**Subtotal (95% CI)**			**39**			**39**	**100.0%**	**15.10 [-3.71, 33.91]**
Heterogeneity: Tau² = 178.62; Chi² = 32.35, df = 1 (P < 0.00001); I² = 97%								
Test for overall effect: Z = 1.57 (P = 0.12)								
**8.17.2 4 weeks**								
Aggarwal, 2018	70.2	9.1	19	64.6	9.8	19	100.0%	5.60 [-0.41, 11.61]
**Subtotal (95% CI)**			**19**			**19**	**100.0%**	**5.60 [-0.41, 11.61]**
Heterogeneity: Not applicable								
Test for overall effect: Z = 1.83 (P = 0.07)								

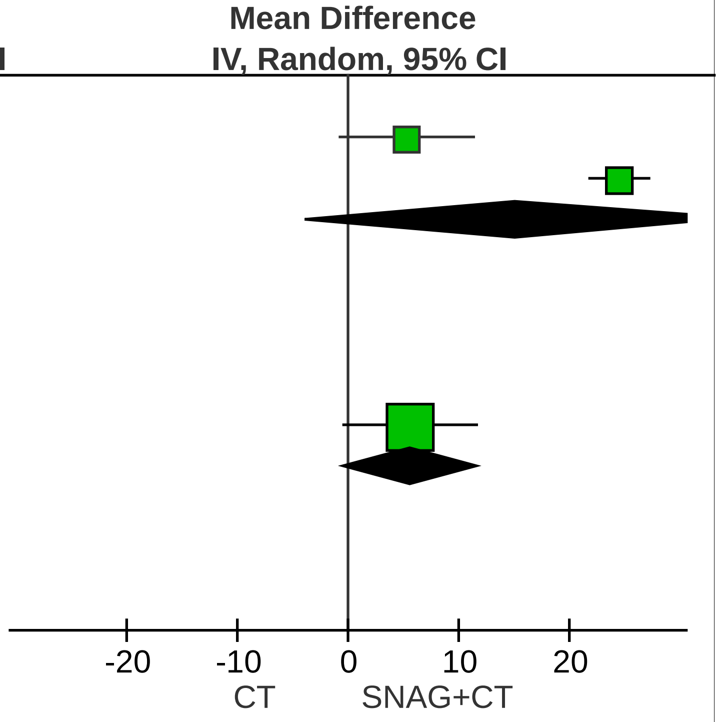

Test for subgroup differences: Chi² = 0.89, df = 1 (P = 0.35), I² = 0%

Figure 8.17 Left rotation comparing SNAG + Conventional Therapy vs. Conventional Therapy at 2/4 weeks (end of the treatment).

All analyses are described as mean differences in degrees with random effects.

The effect sizes for comparisons were calculated using post treatment means.

Higher absolute values mean cervical range of motion improvement after treatment.

SNAGs: Sustained Natural Apophyseal Glides; CT: conventional therapy; Ex: exercise

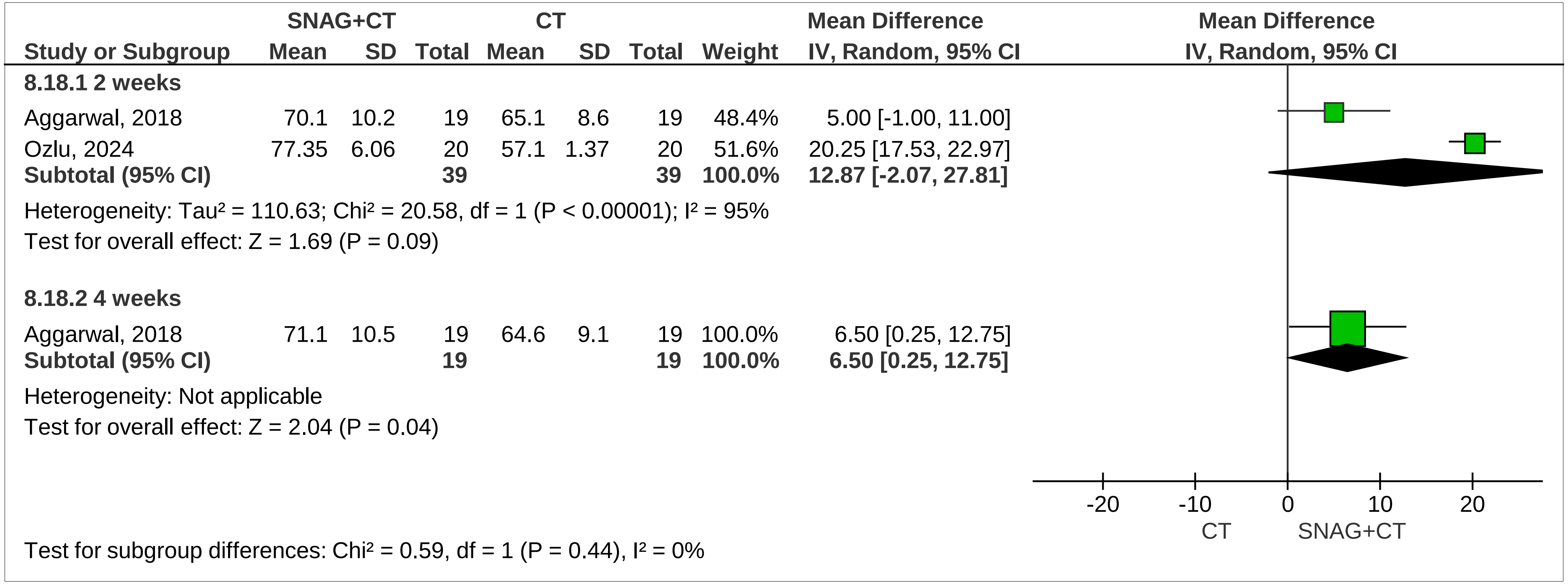

Figure 8.18 Right rotation comparing SNAG + Conventional Therapy vs. Conventional Therapy at 2/4 weeks (end of the treatment).

All analyses are described as mean differences in degrees with random effects.

The effect sizes for comparisons were calculated using post treatment means.

Higher absolute values mean cervical range of motion improvement after treatment.

SNAGs: Sustained Natural Apophyseal Glides; CT: conventional therapy; Ex: exercise

Study or Subgroup	SNAG+CT Mean	SNAG+CT SD	SNAG+CT Total	CT Mean	CT SD	CT Total	Weight	Mean Difference IV, Random, 95% CI
**8.19.1 2 weeks**								
Aggarwal, 2018	13.1	8.2	19	17.5	10.2	19	48.4%	-4.40 [-10.28, 1.48]
Ozlu, 2024	18.18	1.09	20	39.35	1.89	20	51.6%	-21.17 [-22.13, -20.21]
**Subtotal (95% CI)**			**39**			**39**	**100.0%**	**-13.05 [-29.47, 3.38]**
Heterogeneity: Tau² = 135.99; Chi² = 30.39, df = 1 (P < 0.00001); I² = 97%								
Test for overall effect: Z = 1.56 (P = 0.12)								
**8.19.2 4 weeks**								
Aggarwal, 2018	0	0	0	0	0	0		Not estimable
**Subtotal (95% CI)**			**0**			**0**		**Not estimable**
Heterogeneity: Not applicable								
Test for overall effect: Not applicable								
Test for subgroup differences: Not applicable								

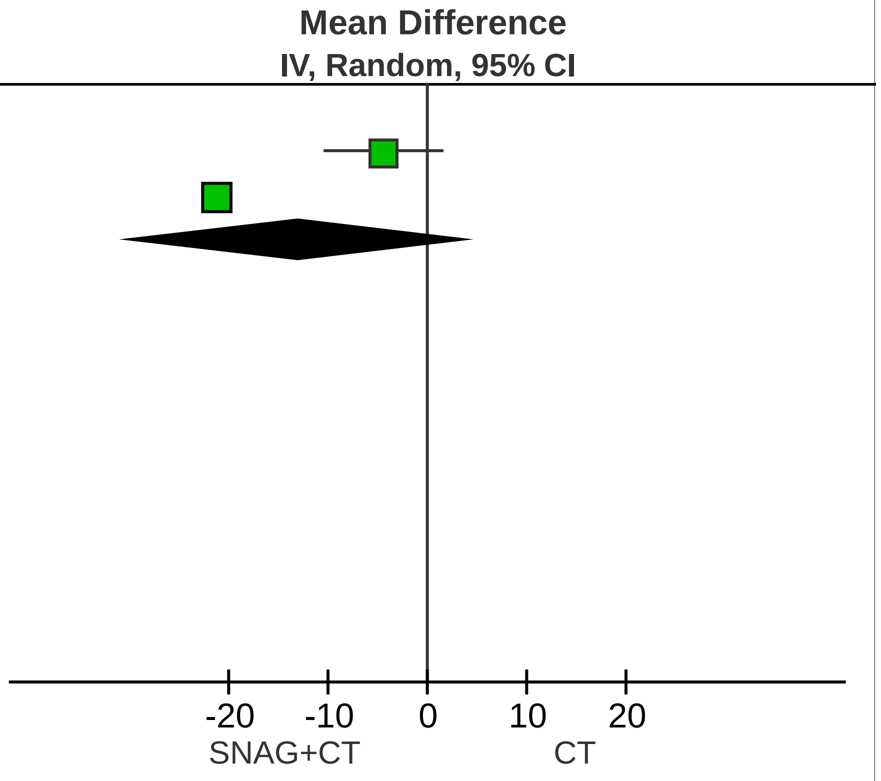

Figure 8.19 Disability comparing SNAG + Conventional Therapy vs. Conventional Therapy at 2 weeks (end of the treatment).

All analyses are described as mean differences in degrees with random effects.
The effect sizes for comparisons were calculated using post treatment means.
Lower absolute values mean improvement on disability after treatment.
SNAGs: Sustained Natural Apophyseal Glides; CT: conventional therapy; Ex: exercise
